# Supplementary material for: Inability to switch from ARID1A-BAF to ARID1B-BAF impairs exit from pluripotency and commitment towards neural crest formation in ARID1B-related neurodevelopmental disorders
Source: Nat Commun. 2021 Nov 9;12:6469. doi: 10.1038/s41467-021-26810-x (PMC8578637; doi:10.1038/s41467-021-26810-x)
Supplement: Supplementary file 1 — Supplementary Information [file 41467_2021_26810_MOESM1_ESM.pdf]

Supplementary information file

Inability to switch from ARID1A-BAF to ARID1B-BAF impairs exit from pluripotency and commitment towards neural crest formation in *ARID1B*-related neurodevelopmental disorders

Luca Pagliaroli[1,#], Patrizia Porazzi[2,#], Alyxandra T. Curtis[1], Chiara Scopa[1], Harald M.M. Mikkers[3], Christian Freund[4], Lucia Daxinger[5], Sandra Deliard[6], Sarah A. Welsh[6], Sarah Offley[6], Connor A. Ott[1], Bruno Calabretta[2], Samantha A. Brugmann[7], Gijs W.E. Santen*[,8], and Marco Trizzino*[,1,9]

a

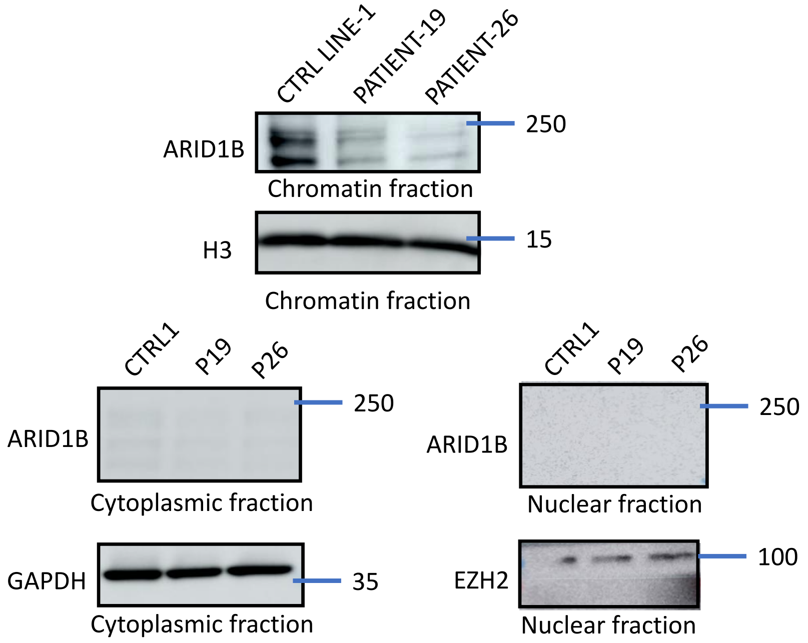

Supplementary Figure S1 – (a) ARID1B Immunoblot performed on the chromatin, nuclear and cytoplasmic fraction performed at the iPSC stage in an *ARID1B*-wt Control Line and in the two patient lines. Experiment repeated twice. Marker represents kDa.

A Pluripotency Analysis (Day 0)

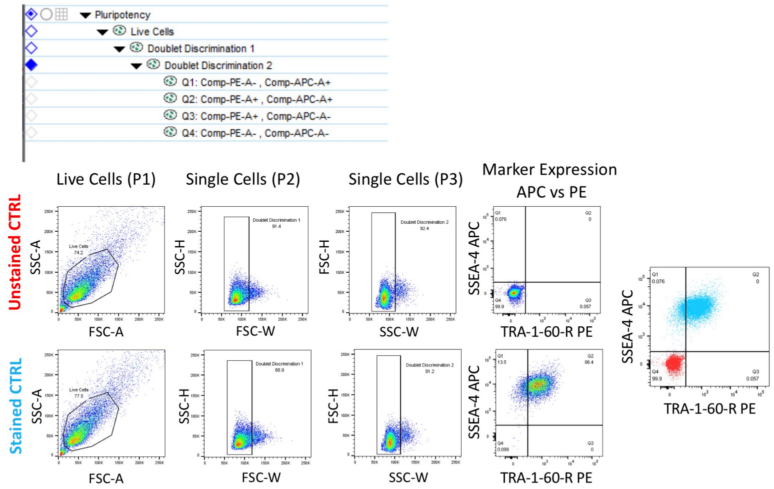

B Differentiation Analysis (Day 14)

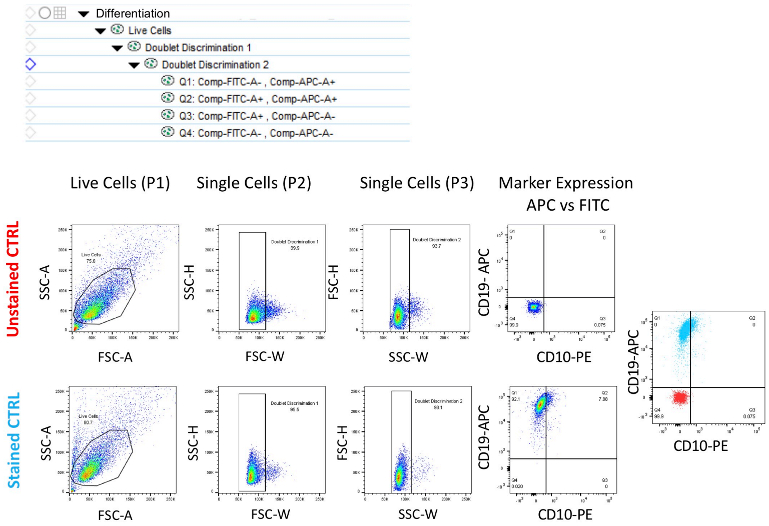

Supplementary Fig. S2: Gating Strategy for Analysis of Pluripotency (A) and Differentiation (B): representation of hierarchical gating for control sample. Unstained control sample, top row; stained control sample, bottom row; Forward Scatter Area (FSC-A) vs Side Scatter Area (SSC-A) gating has been used to identify P1 “Live Cells” on the base of cell size and granularity, and also to remove dead cells and debris. Sequential gating has been used to identify single cells (SSC-H vs FSC-W and FSC-H vs SSC-W, “Doublet Discrimination” 1 and 2) by exclusion of doublets/clumps. Pluripotent cells were then identified by surface biomarker expression of SSEA-4 APC vs TRA-1-60-R PE within the population (Marker Expression APC vs PE). Differentiation cells were identified by surface marker expression of CD19-APC vs CD10-FITC within the population (Marker Expression APC vs FITC). Separation between negative (red population) and positive (blue population) cells is presented to the right.

a

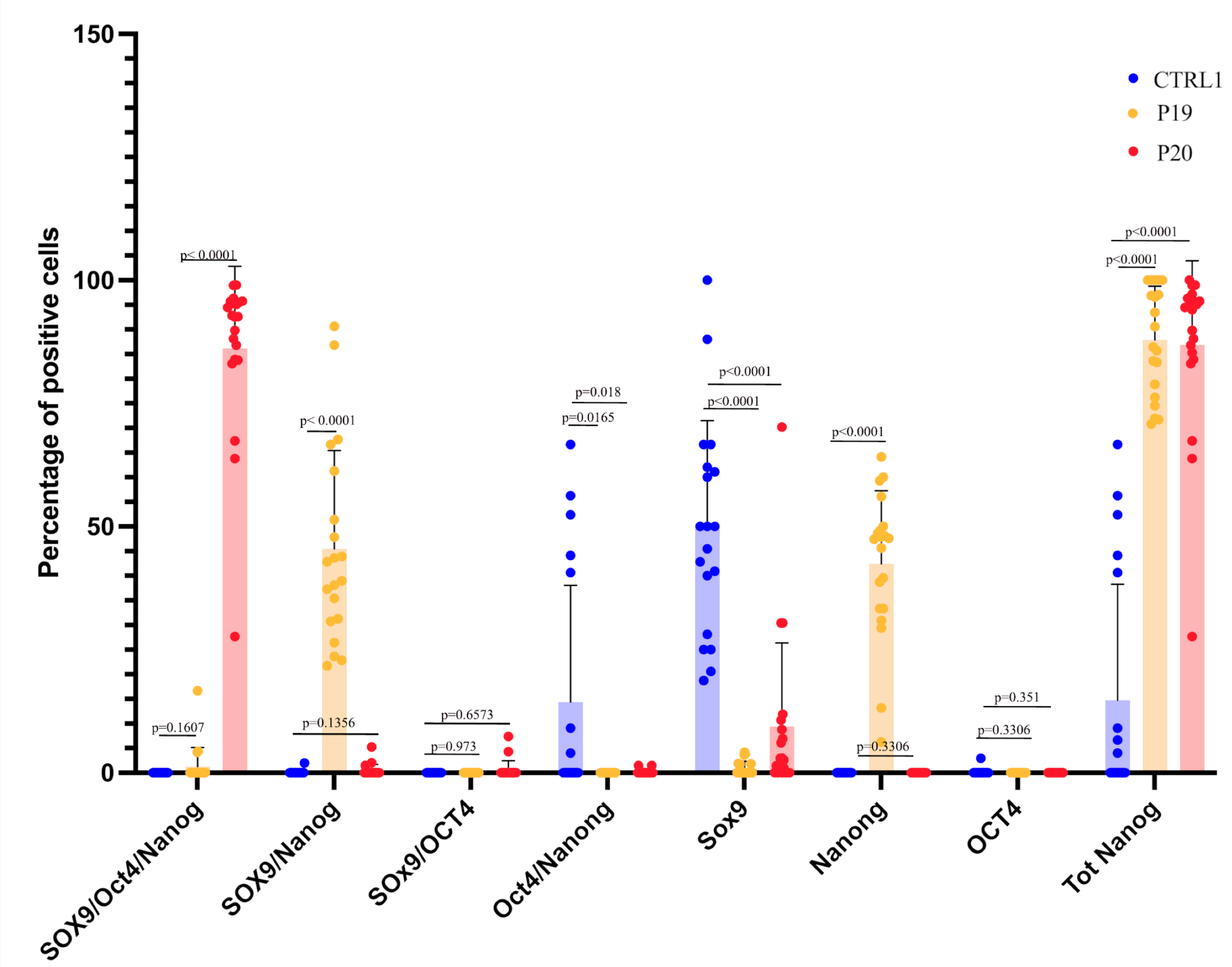

Supplementary Figure S3 – (a) Quantification of immunofluorescence experiment (day-14) showed in Figure 3. P-values computed with two-sided Student's T-Test.

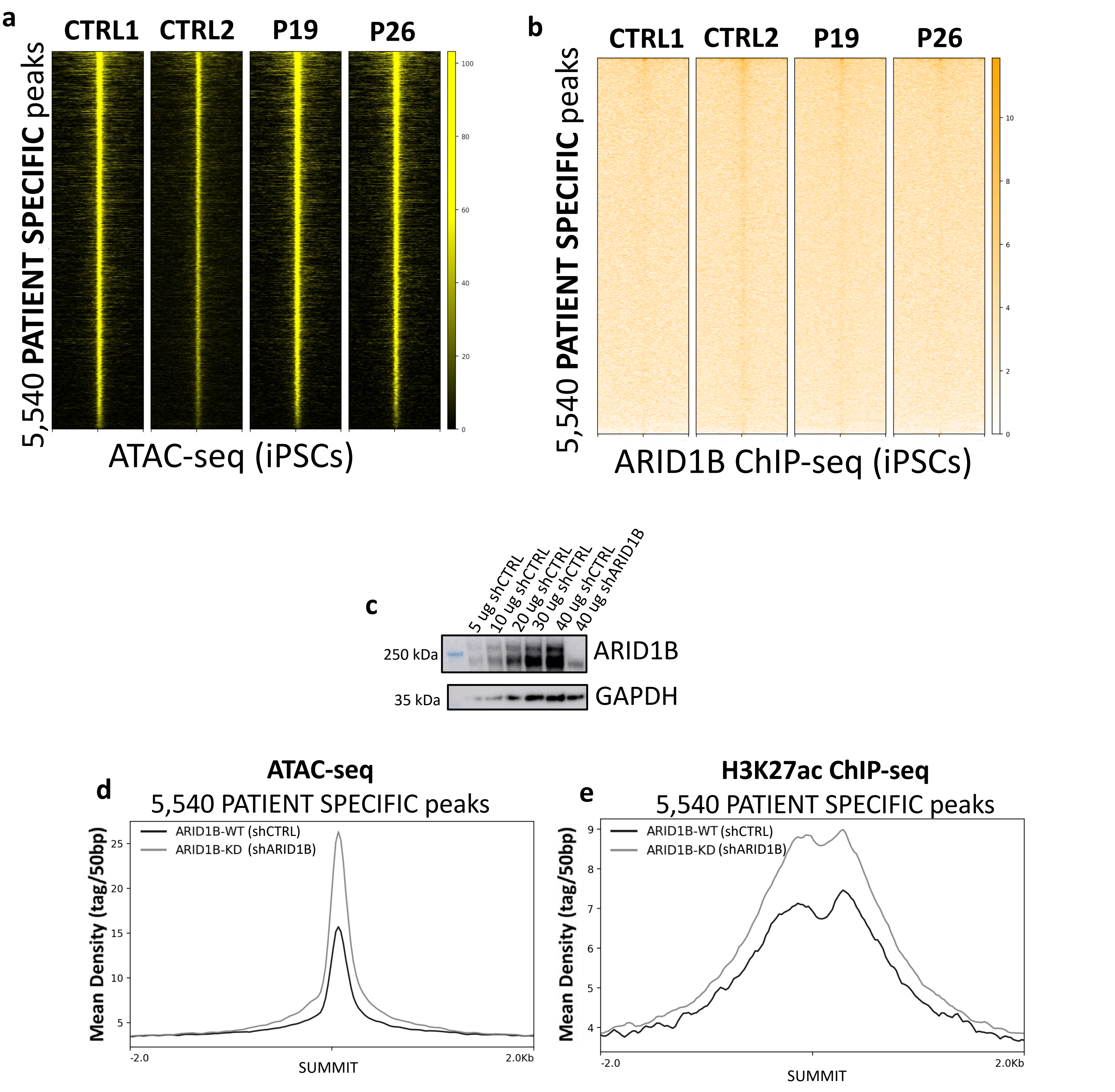

Supplementary Figure S4 – **(a)** Heatmap displaying ATAC-seq signal at the iPSC stage on the 5,540 regions detected as “patient-specific” at CNCC day-5 (peak summit +/-2kb). **(b)** Heatmap displaying ARID1B ChIP-seq signal at the 5,540 PATIENT-SPECIFIC ATAC-seq regions in two control lines and two patient lines at the iPSC stage (peak summit +/-2kb) **(c)** Quantitative immunoblot quantifying ARID1B-KD in Control line-1 at the iPSC stage. Western Blot repeated twice. **(d)** Average profile measuring ATAC-seq signal at the 5,540 PATIENT-SPECIFIC ATAC-seq regions in ARID1B-WT and ARID1B-KD cells (CNCC Day-5). **(e)** Average profile measuring ATAC-seq signal at the 5,540 PATIENT-SPECIFIC H3K27ac ChIP-seq regions in ARID1B-WT and ARID1B-KD cells (CNCC Day-5).

CNCC **Day-5**

a

IP-Mass Spectrometry Data – Control line			
**Protein**	**ARID1B IP rep1**	**ARID1B IP rep2**	**Negative Control**
**ARID1B**	30	27	0
SMARCA4	42	47	2
SMARCC1	40	46	2
ACTL6A	15	17	4
SMARCB1	16	14	2
SMARCE1	14	19	4
SMARCD1	22	24	0
DPF2	11	11	2
SMARCD2	13	11	0
SMARCC2	2	11	2
SS18	2	2	1

b

IP-Mass Spectrometry Data – Patients			
**Protein**	**ARID1A IP P19**	**ARID1A IP P26**	**Negative Control**
**ARID1A**	41	39	0
SMARCA4	52	44	0
SMARCC1	47	39	2
ACTL6A	19	19	0
SMARCB1	19	13	1
SMARCE1	24	17	0
SMARCD1	22	21	0
DPF2	15	10	1
SMARCD2	17	14	0
SMARCC2	15	10	0
SS18	2	4	0

Supplementary Figure S5 – (a) Table displaying all the BAF subunits that coeluted with ARID1B in the IP-MS performed at Day-5 of CNCC5 differentiation in Control Line-1. Values represent unique peptide numbers. **(b)** Table displaying all the BAF subunits that coeluted with ARID1A in the IP-MS performed at Day-5 of CNCC5 differentiation in the patient lines. Values represent unique peptide numbers.
