# Supplementary material for: Inability to switch from ARID1A-BAF to ARID1B-BAF impairs exit from pluripotency and commitment towards neural crest formation in ARID1B-related neurodevelopmental disorders
Source: Nat Commun. 2021 Nov 9;12:6469. doi: 10.1038/s41467-021-26810-x (PMC8578637; doi:10.1038/s41467-021-26810-x)
Supplement: Supplementary file 3 — Description for Additional Supplementary Files [file 41467_2021_26810_MOESM3_ESM.pdf]

Legends for Supplementary Data Files

File Name: Supplementary Data 1
Description: PATIENT-SPECIFIC ATAC-seq PEAKS (CONTROL-SPECIFIC PEAKS ARE IN THE SECOND SHEET)

File Name: Supplementary Data 2
Description: OUTPUT OF MEME-CHIP MOTIF ANALYSIS FOR PATIENT-SPECIFIC ATAC-SEQ PEAKS

File Name: Supplementary Data 3
Description: Differentially expressed genes between control and patient lines at Day-5. The DE genes that also represent the closest gene to a patient-specific ATAC-seq peak are listed in the second sheet. The P-value were adjusted using False Discovery Rate (Benjaminy Hochberg).

File Name: Supplementary Data 4
Description: Pathways enriched in the 598 differentially expressed genes that also represent the closest gene to a patient-specific ATAC-seq peak

File Name: Supplementary Data 5
Description: CONTROL-SPECIFIC SOX2 PEAKS.

File Name: Supplementary Data 6
Description: PATIENT-SPECIFIC NANOG_PEAKS

File Name: Supplementary Data 7
Description: LIST OF PRIMERS USED IN THIS STUDY
