# Supplementary material for: Inability to switch from ARID1A-BAF to ARID1B-BAF impairs exit from pluripotency and commitment towards neural crest formation in ARID1B-related neurodevelopmental disorders
Source: Nat Commun. 2021 Nov 9;12:6469. doi: 10.1038/s41467-021-26810-x (PMC8578637; doi:10.1038/s41467-021-26810-x)
Supplement: Supplementary file 5 — Supplementary Data 2 [file 41467_2021_26810_MOESM5_ESM.pdf]

MEME-ChIP

Motif Analysis of Large Nucleotide Datasets

For further information on how to interpret these results please access http://meme-suite.org//doc/meme-chip-output-format.html.
To get a copy of the MEME software please access http://meme-suite.org.

If you use MEME-ChIP in your research, please cite the following paper:

Philip Machanick and Timothy L. Bailey, "MEME-ChIP: motif analysis of large DNA datasets", *Bioinformatics*, **27**12, 1696-1697, 2011. [full text]

Motifs | Programs | Input Files | Program information | Summary in TSV Format NEW | Motifs in MEME Text Format NEW

Description

patient specific long

Motifs

The significant motifs (E-value ≤ 0.05) found by the programs MEME, DREME and CentriMo; clustered by similarity and ordered by E-value.

Expand All Clusters Collapse All Clusters

Motif Found	Discovery/ Enrichment Program	E-value	Known or Similar Motifs	Distribution	SpaMo FIMO
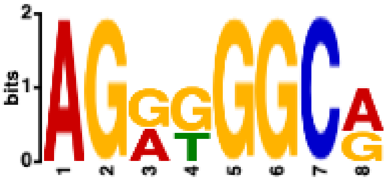	DREME	6.0e-103	CTCF_HUMAN.H11MO.0.A<br>INSM1_HUMAN.H11MO.0.C<br>CTCFL_HUMAN.H11MO.0.A	Not Centrally Enriched	• Mot Spa Ana<br>• Mot Site GFF

Reverse Complement ⇆ Show 1 More ↧

Motif Found	Discovery/ Enrichment Program	E-value	Known or Similar Motifs	Distribution	SpaMo & FIMO
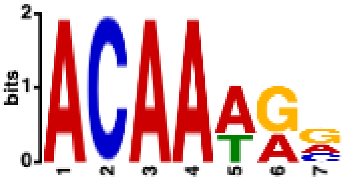	DREME	1.9e-091	SOX2_HUMAN.H11MO.0.A<br>SOX3_HUMAN.H11MO.0.B<br>SOX9_HUMAN.H11MO.0.B	Not Centrally Enriched	• Motif Spacing Analysis<br>• Motif Sites in GFF3

Reverse Complement ⇆

Motif Found	Discovery/ Enrichment Program	E-value	Known or Similar Motifs	Distribution	SpaMo & FIMO
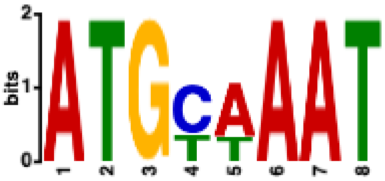	DREME	2.9e-043	NANOG_HUMAN.H11MO.0.A<br>PO5F1_HUMAN.H11MO.0.A<br>PO2F2_HUMAN.H11MO.0.A	Not Centrally Enriched	• Mo[illegible] Spa[illegible] Ana[illegible]<br>• Mo[illegible] Site[illegible] GF[illegible]

Reverse Complement ⇆ Show 1 More ↧

Motif Found	Discovery/ Enrichment Program	E-value	Known or Similar Motifs	Distribution	SpaMo & FIMO
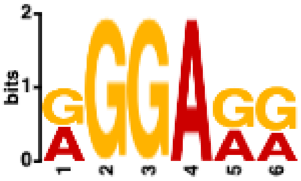	DREME	2.7e-039	VEZF1_HUMAN.H11MO.0.C<br>ZN436_HUMAN.H11MO.0.C<br>IKZF1_HUMAN.H11MO.0.C	Not Centrally Enriched	• Motif Spacing Analysis<br>• Motif Sites in GFF3

Reverse Complement ⇆

Motif Found	Discovery/ Enrichment Program	E-value	Known or Similar Motifs	Distribution	SpaMo & FIMO
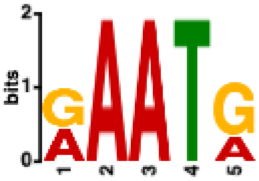	DREME	7.7e-021	PRGR_HUMAN.H11MO.0.A	Not Centrally Enriched	• Motif Spacing Analysis<br>• Motif Sites in GFF3

Reverse Complement ⇆

Motif Found	Discovery/ Enrichment Program	E-value	Known or Similar Motifs	Distribution	SpaMo & FIMO
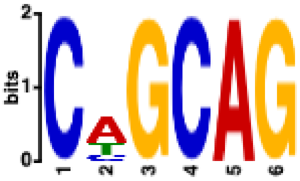	DREME	4.1e-016	ZSC31_HUMAN.H11MO.0.C<br>ZIC3_HUMAN.H11MO.0.B<br>OSR2_HUMAN.H11MO.0.C	Not Centrally Enriched	• Motif Spacing Analysis<br>• Motif Sites in GFF3

Reverse Complement ⇆

Motif Found	Discovery/ Enrichment Program	E-value	Known or Similar Motifs	Distribution	SpaMo & FIMO

Motif Found	Discovery/ Enrichment Program	E-value	Known or Similar Motifs	Distribution	SpaMo & FIMO
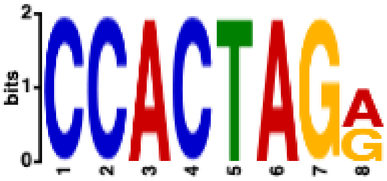	DREME	2.5e-015	CTCF_HUMAN.H11MO.0.A	Not Centrally Enriched	• Motif Spaci Analy<br>• Motif Sites GFF3

Reverse Complement ⇆

Motif Found	Discovery/ Enrichment Program	E-value	Known or Similar Motifs	Distribution
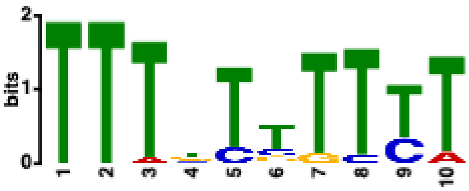	MEME	1.2e-013	PRDM6_HUMAN.H11MO.0.C<br>ANDR_HUMAN.H11MO.0.A<br>FOXO4_HUMAN.H11MO.0.C	Not Centrally Enriched

Reverse Complement ⇆ Show 1 More ↧

Motif Found	Discovery/ Enrichment Program	E-value	Known or Similar Motifs	Distribution	SpaMo & FIMO
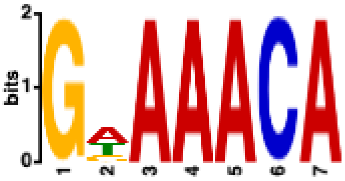	DREME	1.7e-012	FOXO1_HUMAN.H11MO.0.A<br>FOXK1_HUMAN.H11MO.0.A<br>FOXO3_HUMAN.H11MO.0.B	Not Centrally Enriched	• Motif Spacin Analysi<br>• Motif Sites in GFF3

Reverse Complement ⇆

Motif Found	Discovery/ Enrichment Program	E-value	Known or Similar Motifs	Distribution	SpaMo & FIMO
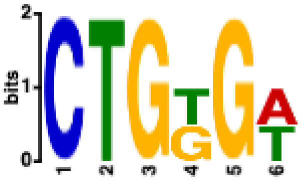	DREME	4.4e-011		Not Centrally Enriched	• Motif Spacing Analysis<br>• Motif Sites in GFF3

Reverse Complement ⇆

Motif Found	Discovery/ Enrichment Program	E-value	Known or Similar Motifs	Distribution	SpaMo & FIMO

Motif Found	Discovery/ Enrichment Program	E-value	Known or Similar Motifs	Distribution	SpaMo & FIMO
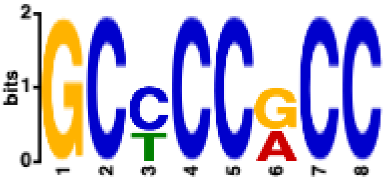	DREME	4.7e-009	KLF3_HUMAN.H11MO.0.B<br>SP4_HUMAN.H11MO.0.A<br>KLF12_HUMAN.H11MO.0.C	Not Centrally Enriched	• Moti Spac Anal<br>• Moti Sites GFF

Reverse Complement ⇆

Motif Found	Discovery/ Enrichment Program	E-value	Known or Similar Motifs	Distribution	SpaMo & FIMO
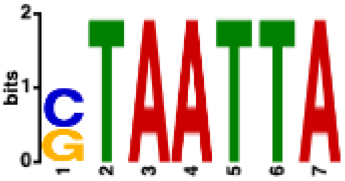	DREME	9.0e-009	DLX3_HUMAN.H11MO.0.C<br>LHX2_HUMAN.H11MO.0.A<br>NOBOX_HUMAN.H11MO.0.C	Not Centrally Enriched	• Motif Spacin Analys<br>• Motif Sites ir GFF3

Reverse Complement ⇆

Motif Found	Discovery/ Enrichment Program	E-value	Known or Similar Motifs	Distribution	SpaMo & FIMO
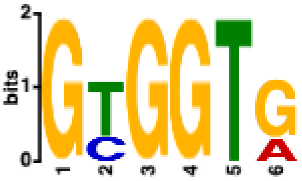	DREME	9.1e-007		Not Centrally Enriched	• Motif Spacing Analysis<br>• Motif Sites in GFF3

Reverse Complement ⇆

Motif Found	Discovery/ Enrichment Program	E-value	Known or Similar Motifs	Distribution	SpaMo & FIMO
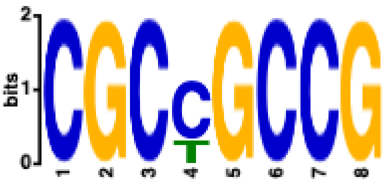	DREME	6.1e-006	TAF1_HUMAN.H11MO.0.A	Not Centrally Enriched	• Motif Spaci Analy<br>• Motif Sites GFF3

Reverse Complement ⇆

Motif Found	Discovery/ Enrichment Program	E-value	Known or Similar Motifs	Distribution	SpaMo & FIMO

Motif Found	Discovery/ Program	E-value	Known or Similar Motifs	Distribution	SpaMo & FIMO
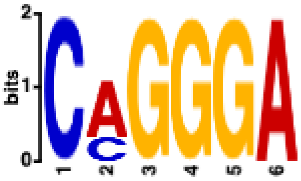	DREME	2.5e-002	ZN436_HUMAN.H11MO.0.C<br>ZN528_HUMAN.H11MO.0.C<br>COE1_HUMAN.H11MO.0.A	Not Centrally Enriched	• Motif Spacing Analysis<br>• Motif Sites in GFF3

Reverse Complement ⇆

Motif Found	Discovery/ Program	E-value	Known or Similar Motifs	Distribution	SpaMo & FIMO
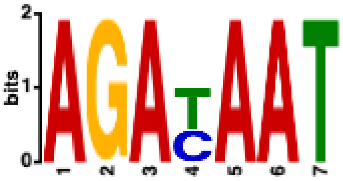	DREME	2.9e-002	EVI1_HUMAN.H11MO.0.B	Not Centrally Enriched	• Motif Spacing Analysis<br>• Motif Sites in GFF3

Reverse Complement ⇆

Motif Found	Discovery/ Program	E-value	Known or Similar Motifs	Distribution	SpaMo & FIMO
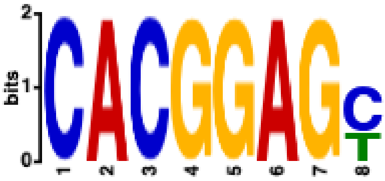	DREME	4.0e-002	NFIA_HUMAN.H11MO.0.C	Not Centrally Enriched	• Motif Spaci<br>Analy<br>• Motif Sites<br>GFF3

Reverse Complement ⇆

Programs

Command	Running Time	Status	Outputs
**getsize** ./ATAC_CNCC5_PATIENT_SPECIFIC_rep2_LONG_VERSION.fasta 1> $metrics	0.05s	Success	
**fasta-most** -min 50 < ./ATAC_CNCC5_PATIENT_SPECIFIC_rep2_LONG_VERSION.fasta 1> $metrics	0.12s	Success	
**fasta-center** -dna -len 100 < ./ATAC_CNCC5_PATIENT_SPECIFIC_rep2_LONG_VERSION.fasta 1> ./seqs-centered	0.19s	Success	• seqs-centered

Command	Running Time	Status	Outputs
**fasta-shuffle-letters** ./seqs-centered ./seqs-shuffled -kmer 2 -tag -dinuc -dna -seed 1	0.08s	Success	• seqs-shuffled
**fasta-get-markov** -nostatus -nosummary -dna -m 1 ./ATAC_CNCC5_PATIENT_SPECIFIC_rep2_LONG_VERSION.fasta ./background	0.03s	Success	• Background
**meme** ./seqs-centered -oc meme_out -mod zoops -nmotifs 12 -minw 6 -maxw 10 -bfile ./background -dna -searchsize 100000 -time 5082 -revcomp -nostatus	1h 1m 45.79s	Success	• MEME HTML<br>• MEME text<br>• MEME XML
**dreme** -verbosity 1 -oc dreme_out -png -dna -p ./seqs-centered -n ./seqs-shuffled -t 5657 -e 0.05	18m 6.85s	Success	• DREME HTML<br>• DREME text<br>• DREME XML
**centrimo** -seqlen 73 -verbosity 1 -oc centrimo_out -bfile ./background -score 5.0 -ethresh 10.0 ./ATAC_CNCC5_PATIENT_SPECIFIC_rep2_LONG_VERSION.fasta meme_out/meme.xml dreme_out/dreme.xml db/HUMAN/HOCOMOCOv11_core_HUMAN_mono_meme_format.meme	2.14s	Warnings	• CentriMo HTML<br>• Site Counts
**tomtom** -verbosity 1 -oc meme_tomtom_out -min-overlap 5 -dist pearson -evalue -thresh 1 -no-ssc meme_out/meme.xml db/HUMAN/HOCOMOCOv11_core_HUMAN_mono_meme_format.meme	4.58s	Success	• Tomtom HTML<br>• Tomtom TSV<br>• Tomtom XML
**tomtom** -verbosity 1 -oc dreme_tomtom_out -min-overlap 5 -dist pearson -evalue -thresh 1 -no-ssc dreme_out/dreme.xml db/HUMAN/HOCOMOCOv11_core_HUMAN_mono_meme_format.meme	3.84s	Success	• Tomtom HTML<br>• Tomtom TSV<br>• Tomtom XML
**tomtom** -verbosity 1 -text -thresh 0.1 ./combined.meme ./combined.meme 1> ./motif_alignment.txt	0.14s	Success	• Motif Alignment
**spamo** -verbosity 1 -oc spamo_out_1 -bgfile ./background -keepprimary -primary AGRKGGCR ./ATAC_CNCC5_PATIENT_SPECIFIC_rep2_LONG_VERSION.fasta dreme_out/dreme.xml meme_out/meme.xml dreme_out/dreme.xml db/HUMAN/HOCOMOCOv11_core_HUMAN_mono_meme_format.meme	3.50s	Warnings	• SpaMo HTML
**spamo** -verbosity 1 -oc spamo_out_2 -bgfile ./background -keepprimary -primary ACAAWRV ./ATAC_CNCC5_PATIENT_SPECIFIC_rep2_LONG_VERSION.fasta dreme_out/dreme.xml meme_out/meme.xml dreme_out/dreme.xml db/HUMAN/HOCOMOCOv11_core_HUMAN_mono_meme_format.meme	5.14s	Warnings	• SpaMo HTML
**spamo** -verbosity 1 -oc spamo_out_3 -bgfile ./background -keepprimary -primary ATGYWAAT ./ATAC_CNCC5_PATIENT_SPECIFIC_rep2_LONG_VERSION.fasta dreme_out/dreme.xml meme_out/meme.xml dreme_out/dreme.xml db/HUMAN/HOCOMOCOv11_core_HUMAN_mono_meme_format.meme	2.58s	Warnings	• SpaMo HTML

Command	Running Time	Status	Outputs
**spamo** -verbosity 1 -oc spamo_out_4 -bgfile ./background -keepprimary -primary RGGARR ./ATAC_CNCC5_PATIENT_SPECIFIC_rep2_LONG_VERSION.fasta dreme_out/dreme.xml meme_out/meme.xml dreme_out/dreme.xml db/HUMAN/HOCOMOCOv11_core_HUMAN_mono_meme_format.meme	12.96s	Warnings	• SpaMo HTML
**spamo** -verbosity 1 -oc spamo_out_5 -bgfile ./background -keepprimary -primary RAATR ./ATAC_CNCC5_PATIENT_SPECIFIC_rep2_LONG_VERSION.fasta dreme_out/dreme.xml meme_out/meme.xml dreme_out/dreme.xml db/HUMAN/HOCOMOCOv11_core_HUMAN_mono_meme_format.meme	9.41s	Warnings	• SpaMo HTML
**spamo** -verbosity 1 -oc spamo_out_6 -bgfile ./background -keepprimary -primary CHGCAG ./ATAC_CNCC5_PATIENT_SPECIFIC_rep2_LONG_VERSION.fasta dreme_out/dreme.xml meme_out/meme.xml dreme_out/dreme.xml db/HUMAN/HOCOMOCOv11_core_HUMAN_mono_meme_format.meme	6.43s	Warnings	• SpaMo HTML
**spamo** -verbosity 1 -oc spamo_out_7 -bgfile ./background -keepprimary -primary CCACTAGR ./ATAC_CNCC5_PATIENT_SPECIFIC_rep2_LONG_VERSION.fasta dreme_out/dreme.xml meme_out/meme.xml dreme_out/dreme.xml db/HUMAN/HOCOMOCOv11_core_HUMAN_mono_meme_format.meme	1.88s	Warnings	• SpaMo HTML
**spamo** -verbosity 1 -oc spamo_out_8 -bgfile ./background -keepprimary -primary TTTKTTTTTT ./ATAC_CNCC5_PATIENT_SPECIFIC_rep2_LONG_VERSION.fasta meme_out/meme.xml meme_out/meme.xml dreme_out/dreme.xml db/HUMAN/HOCOMOCOv11_core_HUMAN_mono_meme_format.meme	4.86s	Warnings	• SpaMo HTML
**spamo** -verbosity 1 -oc spamo_out_9 -bgfile ./background -keepprimary -primary GDAAACA ./ATAC_CNCC5_PATIENT_SPECIFIC_rep2_LONG_VERSION.fasta dreme_out/dreme.xml meme_out/meme.xml dreme_out/dreme.xml db/HUMAN/HOCOMOCOv11_core_HUMAN_mono_meme_format.meme	3.01s	Warnings	• SpaMo HTML
**spamo** -verbosity 1 -oc spamo_out_10 -bgfile ./background -keepprimary -primary CTGKGW ./ATAC_CNCC5_PATIENT_SPECIFIC_rep2_LONG_VERSION.fasta dreme_out/dreme.xml meme_out/meme.xml dreme_out/dreme.xml db/HUMAN/HOCOMOCOv11_core_HUMAN_mono_meme_format.meme	6.34s	Warnings	• SpaMo HTML
**spamo** -verbosity 1 -oc spamo_out_11 -bgfile ./background -keepprimary -primary GCYCCRCC ./ATAC_CNCC5_PATIENT_SPECIFIC_rep2_LONG_VERSION.fasta dreme_out/dreme.xml meme_out/meme.xml dreme_out/dreme.xml db/HUMAN/HOCOMOCOv11_core_HUMAN_mono_meme_format.meme	3.44s	Warnings	• SpaMo HTML

Command	Running Time	Status	Outputs
**spamo** -verbosity 1 -oc spamo_out_12 -bgfile ./background -keepprimary -primary STAATTA ./ATAC_CNCC5_PATIENT_SPECIFIC_rep2_LONG_VERSION.fasta dreme_out/dreme.xml meme_out/meme.xml dreme_out/dreme.xml db/HUMAN/HOCOMOCOv11_core_HUMAN_mono_meme_format.meme	2.66s	Warnings	• SpaMo HTML
**spamo** -verbosity 1 -oc spamo_out_13 -bgfile ./background -keepprimary -primary GYGGTR ./ATAC_CNCC5_PATIENT_SPECIFIC_rep2_LONG_VERSION.fasta dreme_out/dreme.xml meme_out/meme.xml dreme_out/dreme.xml db/HUMAN/HOCOMOCOv11_core_HUMAN_mono_meme_format.meme	4.25s	Warnings	• SpaMo HTML
**spamo** -verbosity 1 -oc spamo_out_14 -bgfile ./background -keepprimary -primary CGCYGCCG ./ATAC_CNCC5_PATIENT_SPECIFIC_rep2_LONG_VERSION.fasta dreme_out/dreme.xml meme_out/meme.xml dreme_out/dreme.xml db/HUMAN/HOCOMOCOv11_core_HUMAN_mono_meme_format.meme	2.65s	Warnings	• SpaMo HTML
**spamo** -verbosity 1 -oc spamo_out_15 -bgfile ./background -keepprimary -primary CMGGGA ./ATAC_CNCC5_PATIENT_SPECIFIC_rep2_LONG_VERSION.fasta dreme_out/dreme.xml meme_out/meme.xml dreme_out/dreme.xml db/HUMAN/HOCOMOCOv11_core_HUMAN_mono_meme_format.meme	5.97s	Warnings	• SpaMo HTML
**spamo** -verbosity 1 -oc spamo_out_16 -bgfile ./background -keepprimary -primary AGAYAAT ./ATAC_CNCC5_PATIENT_SPECIFIC_rep2_LONG_VERSION.fasta dreme_out/dreme.xml meme_out/meme.xml dreme_out/dreme.xml db/HUMAN/HOCOMOCOv11_core_HUMAN_mono_meme_format.meme	2.53s	Warnings	• SpaMo HTML
**spamo** -verbosity 1 -oc spamo_out_17 -bgfile ./background -keepprimary -primary CACGGAGY ./ATAC_CNCC5_PATIENT_SPECIFIC_rep2_LONG_VERSION.fasta dreme_out/dreme.xml meme_out/meme.xml dreme_out/dreme.xml db/HUMAN/HOCOMOCOv11_core_HUMAN_mono_meme_format.meme	1.80s	Warnings	• SpaMo HTML
**fimo** --parse-genomic-coord --verbosity 1 --oc fimo_out_1 --bgfile ./background --motif AGRKGGCR dreme_out/dreme.xml ./ATAC_CNCC5_PATIENT_SPECIFIC_rep2_LONG_VERSION.fasta	0.83s	Success	• FIMO GFF<br>• FIMO HTML<br>• FIMO TSV
**fimo** --parse-genomic-coord --verbosity 1 --oc fimo_out_2 --bgfile ./background --motif ACAAWRV dreme_out/dreme.xml ./ATAC_CNCC5_PATIENT_SPECIFIC_rep2_LONG_VERSION.fasta	0.83s	Success	• FIMO GFF<br>• FIMO HTML<br>• FIMO TSV
**fimo** --parse-genomic-coord --verbosity 1 --oc fimo_out_3 --bgfile ./background --motif ATGYWAAT dreme_out/dreme.xml ./ATAC_CNCC5_PATIENT_SPECIFIC_rep2_LONG_VERSION.fasta	0.83s	Success	• FIMO GFF<br>• FIMO HTML<br>• FIMO TSV

Command	Running Time	Status	Outputs
**fimo** --parse-genomic-coord --verbosity 1 --oc fimo_out_4 --bgfile ./background --motif RGGARR dreme_out/dreme.xml ./ATAC_CNCC5_PATIENT_SPECIFIC_rep2_LONG_VERSION.fasta	0.83s	Success	• FIMO GFF<br>• FIMO HTML<br>• FIMO TSV
**fimo** --parse-genomic-coord --verbosity 1 --oc fimo_out_5 --bgfile ./background --motif RAATR dreme_out/dreme.xml ./ATAC_CNCC5_PATIENT_SPECIFIC_rep2_LONG_VERSION.fasta	0.78s	Success	• FIMO GFF<br>• FIMO HTML<br>• FIMO TSV
**fimo** --parse-genomic-coord --verbosity 1 --oc fimo_out_6 --bgfile ./background --motif CHGCAG dreme_out/dreme.xml ./ATAC_CNCC5_PATIENT_SPECIFIC_rep2_LONG_VERSION.fasta	0.79s	Success	• FIMO GFF<br>• FIMO HTML<br>• FIMO TSV
**fimo** --parse-genomic-coord --verbosity 1 --oc fimo_out_7 --bgfile ./background --motif CCACTAGR dreme_out/dreme.xml ./ATAC_CNCC5_PATIENT_SPECIFIC_rep2_LONG_VERSION.fasta	0.82s	Success	• FIMO GFF<br>• FIMO HTML<br>• FIMO TSV
**fimo** --parse-genomic-coord --verbosity 1 --oc fimo_out_8 --bgfile ./background --motif TTTKTTTTTT meme_out/meme.xml ./ATAC_CNCC5_PATIENT_SPECIFIC_rep2_LONG_VERSION.fasta	0.84s	Success	• FIMO GFF<br>• FIMO HTML<br>• FIMO TSV
**fimo** --parse-genomic-coord --verbosity 1 --oc fimo_out_9 --bgfile ./background --motif GDAAACA dreme_out/dreme.xml ./ATAC_CNCC5_PATIENT_SPECIFIC_rep2_LONG_VERSION.fasta	0.82s	Success	• FIMO GFF<br>• FIMO HTML<br>• FIMO TSV
**fimo** --parse-genomic-coord --verbosity 1 --oc fimo_out_10 --bgfile ./background --motif CTGKGW dreme_out/dreme.xml ./ATAC_CNCC5_PATIENT_SPECIFIC_rep2_LONG_VERSION.fasta	0.79s	Success	• FIMO GFF<br>• FIMO HTML<br>• FIMO TSV
**fimo** --parse-genomic-coord --verbosity 1 --oc fimo_out_11 --bgfile ./background --motif GCYCCRCC dreme_out/dreme.xml ./ATAC_CNCC5_PATIENT_SPECIFIC_rep2_LONG_VERSION.fasta	0.82s	Success	• FIMO GFF<br>• FIMO HTML<br>• FIMO TSV
**fimo** --parse-genomic-coord --verbosity 1 --oc fimo_out_12 --bgfile ./background --motif STAATTA dreme_out/dreme.xml ./ATAC_CNCC5_PATIENT_SPECIFIC_rep2_LONG_VERSION.fasta	0.82s	Success	• FIMO GFF<br>• FIMO HTML<br>• FIMO TSV
**fimo** --parse-genomic-coord --verbosity 1 --oc fimo_out_13 --bgfile ./background --motif GYGGTR dreme_out/dreme.xml ./ATAC_CNCC5_PATIENT_SPECIFIC_rep2_LONG_VERSION.fasta	0.79s	Success	• FIMO GFF<br>• FIMO HTML<br>• FIMO TSV
**fimo** --parse-genomic-coord --verbosity 1 --oc fimo_out_14 --bgfile ./background --motif CGCYGCCG dreme_out/dreme.xml ./ATAC_CNCC5_PATIENT_SPECIFIC_rep2_LONG_VERSION.fasta	0.81s	Success	• FIMO GFF<br>• FIMO HTML<br>• FIMO TSV
**fimo** --parse-genomic-coord --verbosity 1 --oc fimo_out_15 --bgfile ./background --motif CMGGGA dreme_out/dreme.xml ./ATAC_CNCC5_PATIENT_SPECIFIC_rep2_LONG_VERSION.fasta	0.79s	Success	• FIMO GFF<br>• FIMO HTML<br>• FIMO TSV
**fimo** --parse-genomic-coord --verbosity 1 --oc fimo_out_16 --bgfile ./background --motif AGAYAAT dreme_out/dreme.xml ./ATAC_CNCC5_PATIENT_SPECIFIC_rep2_LONG_VERSION.fasta	0.81s	Success	• FIMO GFF<br>• FIMO HTML<br>• FIMO TSV

Command	Running Time	Status	Outputs
**fimo** --parse-genomic-coord --verbosity 1 --oc fimo_out_17 --bgfile ./background --motif CACGGAGY dreme_out/dreme.xml ./ATAC_CNCC5_PATIENT_SPECIFIC_rep2_LONG_VERSION.fasta	1.08s	Success	• FIMO GFF<br>• FIMO HTML<br>• FIMO TSV

Input Files

Alphabet

Background source: built from the (primary) sequences

Background source: built from the (primary) sequences

Name	Bg.				Bg.	Name
Adenine	0.2304	A	~	T	0.2304	Thymine
Cytosine	0.2696	C	~	G	0.2696	Guanine

Name	Bg.				Bg.	Name
Adenine	0.2304	A	~	T	0.2304	Thymine
Cytosine	0.2696	C	~	G	0.2696	Guanine

Primary Sequences

Database	Source	Sequence Count
ATAC CNCC5 PATIENT SPECIFIC rep2 LONG VERSION	ATAC_CNCC5_PATIENT_SPECIFIC_rep2_LONG_VERSION.fasta	8144

Motifs

Database	Source	Motif Count
HOCOMOCOv11 core HUMAN mono meme format	db/HUMAN/HOCOMOCOv11_core_HUMAN_mono_meme_format.meme	401

MEME-ChIP version
5.2.0 (Release date: Wed Oct 14 12:02:54 2020 -0700)

Reference
Philip Machanick and Timothy L. Bailey, "MEME-ChIP: motif analysis of large DNA datasets", *Bioinformatics*, **27**12, 1696-1697, 2011.

Command line summary

```
meme-chip -oc . -time 300 -ccut 100 -fdesc description -order 1 -db
db/HUMAN/HOCOMOCOv11_core_HUMAN_mono_meme_format.meme -meme-mod zoops -meme-minw 6 -
meme-maxw 10 -meme-nmotifs 12 -meme-searchsize 100000 -dreme-e 0.05 -centrimo-score 5.0
-centrimo-ethresh 10.0 ATAC_CNCC5_PATIENT_SPECIFIC_rep2_LONG_VERSION.fasta
```
