# Supplementary material for: Inability to switch from ARID1A-BAF to ARID1B-BAF impairs exit from pluripotency and commitment towards neural crest formation in ARID1B-related neurodevelopmental disorders
Source: Nat Commun. 2021 Nov 9;12:6469. doi: 10.1038/s41467-021-26810-x (PMC8578637; doi:10.1038/s41467-021-26810-x)
Supplement: Supplementary file 7 — Supplementary Data 4 [file 41467_2021_26810_MOESM7_ESM.pdf]

#Supplementary File S4 - Pathways enriched in the 598 differentially expressed genes that also represent the closest gene to a patient-specific ATAC-seq peak

© 2000-2021 QIAGEN. All rights reserved.

Ingenuity Canonical Pathways -log(p-value) Ratio z-score Molecules
Hepatic Fibrosis Signaling Pathway 8.29E00 8.47E-02 NaN
ACVR1,ACVR2A,BCL2,CCND1,CEBPB,COL1A1,COL1A2,COL2A1,CREB3L3,CREBBP,FGFR1,FNBP1,FZD7,GLI2,IRS2,LRP5,NGFR,NRAS,PDGFA,PDGFD,PDK1,PIK3CB,PIK3R3,PRKD1,PRKD3,RHOH,SMAD3,TCF7L1,TGFBR2,TNFRSF1A,WNT7B,WNT8B
Molecular Mechanisms of Cancer 6.14E00 7.25E-02 NaN
ABL1,APH1B,ARHGEF17,ARHGEF18,BCL2,CASP8,CCND1,CREBBP,E2F7,FNBP1,FZD7,GAB2,HIPK2,LRP5,MAX,NRAS,PIK3CB,PIK3R3,PLCB4,PRKD1,PRKD3,RAPGEF1,RASGRF2,RHOH,SMAD3,SRC,TGFBR2,WNT7B,WNT8B
Glioblastoma Multiforme Signaling 5.19E00 9.7E-02 NaN
CCND1,E2F7,FNBP1,FZD7,ITPR3,NRAS,PDGFA,PDGFD,PIK3CB,PIK3R3,PLCB4,PLCL1,RHOH,SRC,WNT7B,WNT8B
Factors Promoting Cardiogenesis in Vertebrates 5.06E00 1E-01 NaN
ACVR1,ACVR2A,CCND1,CREB3L3,CREBBP,FZD7,LRP5,PLCB4,PLCL1,PRKD1,PRKD3,TCF7L1,TGFBR2,WNT7B,WNT8B
Human Embryonic Stem Cell Pluripotency 4.95E00 1.04E-01 NaN
ACVR1,FGFR1,FGFR2,FZD7,PDGFA,PDGFD,PIK3CB,PIK3R3,SALL4,SMAD3,TCF7L1,TGFBR2,WNT7B,WNT8B
Wnt/β-catenin Signaling 4.93E00 9.25E-02 NaN
ACVR1,ACVR2A,CCND1,CREBBP,FZD7,KREMEN1,LRP5,PPP2R2B,RARG,SOX21,SRC,TCF7L1,TGFBR2,TLE3,WNT7B,WNT8B
Role of Macrophages, Fibroblasts and Endothelial Cells in Rheumatoid Arthritis 4.58E00 7.01E-02 NaN
CCND1,CEBPB,CREB3L3,CREBBP,FZD7,LRP5,MAPKAPK2,NGFR,NRAS,PDGFA,PDGFD,PIK3CB,PIK3R3,PLCB4,PLCL1,PRKD1,PRKD3,SRC,TCF7L1,TNFRSF1A,WNT7B,WNT8B
Hepatic Fibrosis / Hepatic Stellate Cell Activation 4.53E00 8.6E-02 NaN
BCL2,COL1A1,COL1A2,COL2A1,COL5A1,COL6A3,FGFR1,FGFR2,KLF6,MYH9,NGFR,PDGFA,PDGFD,SMAD3,TGFBR2,TNFRSF1A
PPARα/RXRα Activation 4.39E00 8.38E-02 NaN
ABCA1,ACVR1,ACVR2A,ADIPOR1,CREBBP,GPD2,HSP90AA1,LPL,MAP4K4,NCOR2,NRAS,PLCB4,PLCL1,SLC27A1,SMAD3,TGFBR2
Type II Diabetes Mellitus Signaling 4.07E00 9.15E-02 NaN
ABCC8,ADIPOR1,CACNA1F,CACNB4,CEBPB,IRS2,NGFR,PIK3CB,PIK3R3,PRKD1,PRKD3,SLC27A1,TNFRSF1A
Senescence Pathway 3.95E00 6.91E-02 NaN
ACVR1,ACVR2A,ATF3,CAPN2,CCND1,CEBPB,CREBBP,E2F7,HIPK2,ITPR3,MAPKAPK2,NRAS,PDK1,PIK3CB,PIK3R3,PPP2R2B,SIRT1,SMAD3,TGFBR2
Glioma Signaling 3.87E00 1E-01 NaN
ABL1,CAMK1D,CCND1,E2F7,NRAS,PDGFA,PDGFD,PIK3CB,PIK3R3,PRKD1,PRKD3
White Adipose Tissue Browning Pathway 3.87E00 9.3E-02 NaN
CACNA1F,CACNB4,CEBPB,CREB3L3,CREBBP,FGFR1,FGFR2,FOXC2,GUCY1A2,PRKG2,RA

RG,SIRT1
Macropinocytosis Signaling 3.82E00 1.18E-01 NaN
NRAS,PDGFA,PDGFD,PIK3CB,PIK3R3,PRKD1,PRKD3,SRC,USP6NL
Regulation of the Epithelial-Mesenchymal Transition Pathway 3.82E00
7.81E-02 NaN
APH1B,FGFR1,FGFR2,FOXC2,FZD7,NOTCH2,NRAS,PDGFD,PIK3CB,PIK3R3,SMAD3,TCF
7L1,TGFBR2,WNT7B,WNT8B
Sperm Motility 3.61E00 7.17E-02 NaN
ABL1,EPHA3,FGFR1,FGFR2,GUCY1A2,ITPR3,PDE4B,PLA2R1,PLAAT5,PLCB4,PLCL1,P
RKD1,PRKD3,PRKG2,PTK7,SRC
PPAR Signaling 3.4E00 9.52E-02 NaN
CREBBP,HSP90AA1,MAP4K4,NCOR2,NGFR,NRAS,NRIP1,PDGFA,PDGFD,TNFRSF1A
GP6 Signaling Pathway 3.38E00 8.8E-02 NaN
COL1A1,COL1A2,COL2A1,COL5A1,COL6A3,LAMB2,NCK1,PIK3CB,PIK3R3,PRKD1,PRKD
3
Integrin Signaling 3.33E00 7.04E-02 NaN
ABL1,ASAP1,BCAR1,BCAR3,CAPN2,FNBP1,GSN,NCK1,NRAS,PIK3CB,PIK3R3,PXN,RAP
GEF1,RHOH,SRC
Axonal Guidance Signaling 3.25E00 5.26E-02 NaN
ABL1,BCAR1,EPHA3,FZD7,GLI2,MMP25,NCK1,NGFR,NRAS,NTN5,PAPPA,PDGFA,PDGFD
,PIK3CB,PIK3R3,PLCB4,PLCL1,PLXNC1,PRKD1,PRKD3,PXN,SHANK2,SRGAP1,UNC5C,
WNT7B,WNT8B
Prostate Cancer Signaling 3.23E00 9.89E-02 NaN
ABL1,BCL2,CCND1,CREB3L3,CREBBP,HSP90AA1,NRAS,PIK3CB,PIK3R3
Osteoarthritis Pathway 3.19E00 6.82E-02 NaN
CASP8,CEBPB,COL2A1,CREB3L3,CREBBP,FGFR1,FZD7,GLI2,SIK3,SIRT1,SMAD3,TCF
7L1,TGFBR2,TNFRSF1A,WNT8B
Estrogen-Dependent Breast Cancer Signaling 3.15E00 1.07E-01 NaN
CCND1,CREB3L3,CREBBP,HSD17B14,NRAS,PIK3CB,PIK3R3,SRC
PTEN Signaling 3.07E00 8.09E-02 NaN
BCAR1,BCL2,CCND1,FGFR1,FGFR2,FOXG1,NGFR,NRAS,PIK3CB,PIK3R3,TGFBR2
Aldosterone Signaling in Epithelial Cells 3.06E00 7.59E-02 NaN
DNAJB12,DNAJC15,HSP90AA1,ITPR3,PIK3CB,PIK3R3,PIP5K1C,PIP5KL1,PLCB4,PLC
L1,PRKD1,PRKD3
Colorectal Cancer Metastasis Signaling 3.01E00 6.32E-02 NaN
CCND1,FNBP1,FZD7,LRP5,MMP25,NRAS,PIK3CB,PIK3R3,RHOH,SMAD3,SRC,TCF7L1,T
GFBR2,TNFRSF1A,WNT7B,WNT8B
p53 Signaling 3E00 9.18E-02 NaN
BCL2,CCND1,HIPK2,MDM4,PIK3CB,PIK3R3,PLAGL1,RPRM,SIRT1
Ovarian Cancer Signaling 3E00 7.91E-02 NaN
ABL1,BCL2,CCND1,FZD7,NRAS,PIK3CB,PIK3R3,SRC,TCF7L1,WNT7B,WNT8B
Cholecystokinin/Gastrin-mediated Signaling 2.97E00 8.4E-02 NaN
BCAR1,FNBP1,ITPR3,NRAS,PLCB4,PRKD1,PRKD3,PXN,RHOH,SRC
FAT10 Cancer Signaling Pathway 2.94E00 1.3E-01 NaN
ACVR1,ACVR2A,NGFR,SMAD3,TGFBR2,TNFRSF1A
Prolactin Signaling 2.93E00 9.88E-02 NaN
CEBPB,CREBBP,NRAS,PIK3CB,PIK3R3,PRKD1,PRKD3,PRLR
Thrombopoietin Signaling 2.92E00 1.11E-01 NaN
GAB2,IRS2,NRAS,PIK3CB,PIK3R3,PRKD1,PRKD3
Neuropathic Pain Signaling In Dorsal Horn Neurons 2.9E00 8.91E-02 NaN

CAMK1D,ITPR3,PIK3CB,PIK3R3,PLCB4,PLCL1,PRKD1,PRKD3,SRC
Endothelin-1 Signaling 2.89E00 6.91E-02 NaN
CASP8,GUCY1A2,ITPR3,NRAS,PIK3CB,PIK3R3,PLA2R1,PLAAT5,PLCB4,PLCL1,PRKD1,PRKD3,SRC
Synaptic Long Term Depression 2.87E00 6.88E-02 NaN
CACNA1F,CACNB4,GUCY1A2,ITPR3,NRAS,PLA2R1,PLAAT5,PLCB4,PLCL1,PPP2R2B,PRKD1,PRKD3,PRKG2
Ephrin Receptor Signaling 2.87E00 6.88E-02 NaN
ABL1,BCAR1,CREB3L3,CREBBP,EPHA3,MAP4K4,NCK1,NRAS,PDGFA,PDGFD,PXN,RAPGEF1,SRC
CXCR4 Signaling 2.85E00 7.19E-02 NaN
BCAR1,FNBP1,ITPR3,NRAS,PIK3CB,PIK3R3,PLCB4,PRKD1,PRKD3,PXN,RHOH,SRC
ILK Signaling 2.85E00 6.84E-02 NaN
CCND1,CREB3L3,CREBBP,FLNC,FNBP1,IRS2,MYH9,PIK3CB,PIK3R3,PPP2R2B,PXN,RHOH,TNFRSF1A
Xenobiotic Metabolism Signaling 2.85E00 5.92E-02 NaN
ALDH9A1,CAMK1D,CREBBP,FMO4,HSP90AA1,MGMT,MGST1,MGST2,NCOR2,NRAS,NRIP1,PIK3CB,PIK3R3,PPP2R2B,PRKD1,PRKD3,SOD3
Chronic Myeloid Leukemia Signaling 2.84E00 8.74E-02 NaN
ABL1,CCND1,E2F7,GAB2,NRAS,PIK3CB,PIK3R3,SMAD3,TGFBR2
Germ Cell-Sertoli Cell Junction Signaling 2.76E00 7.02E-02 NaN
BCAR1,FNBP1,GSN,NRAS,PIK3CB,PIK3R3,PXN,RHOH,SRC,TGFBR2,TJP1,TNFRSF1A

Myc Mediated Apoptosis Signaling 2.75E00 1.2E-01 NaN
BCL2,CASP8,CREBBP,MAX,NGFR,TNFRSF1A
Insulin Secretion Signaling Pathway 2.74E00 6.15E-02 NaN
ABCC8,CREB3L3,CREBBP,GLP1R,ITPR3,PCSK1,PIK3CB,PIK3R3,PLCB4,PLCL1,PRKD1,PRKD3,PRLR,SRC,SSR2
p70S6K Signaling 2.71E00 7.75E-02 NaN
F2RL2,NRAS,PIK3CB,PIK3R3,PLCB4,PLCL1,PPP2R2B,PRKD1,PRKD3,SRC
Gap Junction Signaling 2.69E00 6.57E-02 NaN
DBN1,GUCY1A2,ITPR3,NRAS,PIK3CB,PIK3R3,PLCB4,PLCL1,PRKD1,PRKD3,PRKG2,SRC,TJP1
Tumor Microenvironment Pathway 2.66E00 6.82E-02 NaN
BCL2,CCND1,COL1A1,COL1A2,FOXG1,MMP25,NRAS,PDGFA,PDGFD,PIK3CB,PIK3R3,TNFRSF1A
Estrogen Receptor Signaling 2.62E00 5.49E-02 NaN
BCL2,CCND1,CREB3L3,CREBBP,FOXG1,HSP90AA1,MED15,MMP25,NCOR2,NRAS,NRIP1,PIK3CB,PIK3R3,PLCB4,PLCL1,PRKD1,PRKD3,SRC
Adipogenesis pathway 2.59E00 7.46E-02 NaN
CEBPB,FGFR1,FGFR2,FOXC2,FZD7,KLF3,LPL,SIRT1,SMAD3,TNFRSF1A
Melanocyte Development and Pigmentation Signaling 2.51E00 8.51E-02 NaN
BCL2,CREB3L3,CREBBP,KITLG,NRAS,PIK3CB,PIK3R3,SRC
HOTAIR Regulatory Pathway 2.51E00 6.88E-02 NaN
AEBP2,COL1A1,COL1A2,CREBBP,MMP25,PIK3CB,PIK3R3,RCOR1,TCF7L1,WNT7B,WNT8B
Thrombin Signaling 2.5E00 6.25E-02 NaN
CAMK1D,F2RL2,FNBP1,ITPR3,NRAS,PIK3CB,PIK3R3,PLCB4,PLCL1,PRKD1,PRKD3,RHOH,SRC
Sphingosine-1-phosphate Signaling 2.46E00 7.69E-02 NaN

CASP8,FNBP1,PDGFA,PDGFD,PIK3CB,PIK3R3,PLCB4,PLCL1,RHOH
TGF-β Signaling 2.46E00 8.33E-02 NaN
ACVR1,ACVR2A,BCL2,CREBBP,NRAS,PITX2,SMAD3,TGFBR2
GDNF Family Ligand-Receptor Interactions 2.45E00 9.21E-02 NaN
DOK5,IRS2,ITPR3,NCK1,NRAS,PIK3CB,PIK3R3
D-myo-inositol (1,4,5)-Trisphosphate Biosynthesis 2.44E00 1.6E-01 NaN
PI4K2A,PIP5K1C,PIP5KL1,PLCB4
Regulation Of The Epithelial Mesenchymal Transition By Growth Factors Pathway 2.42E00 6.38E-02 NaN
FGFR1,FGFR2,FOXC2,NGFR,NRAS,PDGFA,PDGFD,PIK3CB,PIK3R3,SMAD3,TGFBR2,TNFRSF1A
Role of NANOG in Mammalian Embryonic Stem Cell Pluripotency 2.42E00 7.56E-02 NaN CDX2,FZD7,NRAS,PIK3CB,PIK3R3,SALL4,TCF7L1,WNT7B,WNT8B

NRF2-mediated Oxidative Stress Response 2.41E00 6.35E-02 NaN
CREBBP,DNAJB12,DNAJC15,HERPUD1,MGST1,MGST2,NRAS,PIK3CB,PIK3R3,PRKD1,PRKD3,SOD3
Xenobiotic Metabolism CAR Signaling Pathway 2.41E00 6.35E-02 NaN

ALDH9A1,CREBBP,FMO4,HSP90AA1,MGST1,MGST2,NRIP1,PPP2R2B,PRKD1,PRKD3,SOD3,SRC
Role of NFAT in Cardiac Hypertrophy 2.4E00 6.07E-02 NaN
CACNA1F,CACNB4,CAMK1D,ITPR3,NRAS,PIK3CB,PIK3R3,PLCB4,PLCL1,PRKD1,PRKD3,SRC,TGFBR2
Nitric Oxide Signaling in the Cardiovascular System 2.38E00 8.08E-02 NaN
GUCY1A2,HSP90AA1,ITPR3,PIK3CB,PIK3R3,PRKD1,PRKD3,PRKG2
IL-3 Signaling 2.36E00 8.86E-02 NaN
GAB2,NRAS,PIK3CB,PIK3R3,PRKD1,PRKD3,RAPGEF1
Thyroid Cancer Signaling 2.36E00 8.86E-02 NaN
CCND1,IRS2,NRAS,PDK1,PIK3CB,PIK3R3,TCF7L1
Role of Osteoblasts, Osteoclasts and Chondrocytes in Rheumatoid Arthritis 2.33E00 5.96E-02 NaN
BCL2,COL1A1,FZD7,GSN,LRP5,NGFR,PIK3CB,PIK3R3,SRC,TCF7L1,TNFRSF1A,WNT7B,WNT8B
RAR Activation 2.32E00 6.19E-02 NaN
CRABP2,CREBBP,MAPKAPK2,NCOR2,NRIP1,PIK3CB,PIK3R3,PRKD1,PRKD3,RARG,SMAD3,SRC
Virus Entry via Endocytic Pathways 2.3E00 7.84E-02 NaN
ABL1,FLNC,NRAS,PIK3CB,PIK3R3,PRKD1,PRKD3,SRC
PEDF Signaling 2.27E00 8.54E-02 NaN
BCL2,CASP8,NRAS,PIK3CB,PIK3R3,TCF7L1,WASF2
GNRH Signaling 2.25E00 6.36E-02 NaN
CACNA1F,CACNB4,CREB3L3,CREBBP,ITPR3,NRAS,PLCB4,PRKD1,PRKD3,PXN,SRC
IL-6 Signaling 2.25E00 7.14E-02 NaN
CEBPB,COL1A1,MAP4K4,MAPKAPK2,NGFR,NRAS,PIK3CB,PIK3R3,TNFRSF1A
FAK Signaling 2.25E00 7.69E-02 NaN
ASAP1,BCAR1,CAPN2,NRAS,PIK3CB,PIK3R3,PXN,SRC
14-3-3-mediated Signaling 2.23E00 7.09E-02 NaN
NRAS,PIK3CB,PIK3R3,PLCB4,PLCL1,PRKD1,PRKD3,SRC,TNFRSF1A

Atherosclerosis Signaling 2.23E00 7.09E-02 NaN
COL1A1,COL1A2,COL2A1,GLG1,LPL,PDGFA,PDGFD,PLA2R1,PLAAT5
P2Y Purigenic Receptor Signaling Pathway 2.23E00 7.09E-02 NaN
CREB3L3,CREBBP,NRAS,PIK3CB,PIK3R3,PLCB4,PLCL1,PRKD1,PRKD3
TR/RXR Activation 2.21E00 8.33E-02 NaN
COL6A3,ENO1,NCOR2,NXPH2,PIK3CB,PIK3R3,RAB3B
FGF Signaling 2.21E00 8.33E-02 NaN
CREB3L3,CREBBP,FGFR1,FGFR2,MAPKAPK2,PIK3CB,PIK3R3
PAK Signaling 2.2E00 7.55E-02 NaN
EPHA3,NCK1,NRAS,PDGFA,PDGFD,PIK3CB,PIK3R3,PXN
Epithelial Adherens Junction Signaling 2.2E00 6.58E-02 NaN
ACVR1,ACVR2A,FGFR1,MYH9,NOTCH2,NRAS,RAPGEF1,SRC,TCF7L1,TGFBR2
PDGF Signaling 2.16E00 8.14E-02 NaN
ABL1,NRAS,PDGFA,PDGFD,PIK3CB,PIK3R3,SRC
NF-κB Signaling 2.15E00 6.15E-02 NaN
CASP8,CREBBP,FGFR1,FGFR2,MAP4K4,NGFR,NRAS,PIK3CB,PIK3R3,TGFBR2,TNFRSF1
A
Ephrin A Signaling 2.14E00 1.06E-01 NaN
BCAR1,EPHA3,NGFR,PIK3CB,PIK3R3
Pancreatic Adenocarcinoma Signaling 2.13E00 7.34E-02 NaN
ABL1,BCL2,CCND1,E2F7,PIK3CB,PIK3R3,SMAD3,TGFBR2
ErbB4 Signaling 2.11E00 8.96E-02 NaN
APH1B,NRAS,PIK3CB,PIK3R3,PRKD1,PRKD3
Ceramide Signaling 2.1E00 7.95E-02 NaN
BCL2,NGFR,NRAS,PIK3CB,PIK3R3,PPP2R2B,TNFRSF1A
MSP-RON Signaling In Cancer Cells Pathway 2.08E00 6.72E-02 NaN
CCND1,CREB3L3,CREBBP,KLK10,NRAS,PIK3CB,PIK3R3,SRC,TCF7L1
Acute Myeloid Leukemia Signaling 2.08E00 7.87E-02 NaN
CCND1,KITLG,NRAS,PIK3CB,PIK3R3,PIM1,TCF7L1
Dendritic Cell Maturation 2.06E00 5.98E-02 NaN
COL1A1,COL1A2,COL2A1,CREB3L3,CREBBP,NGFR,PIK3CB,PIK3R3,PLCB4,PLCL1,TNF
RSF1A
Androgen Signaling 2.04E00 6.62E-02 NaN
CACNA1F,CACNB4,CCND1,CREBBP,HSP90AA1,PRKD1,PRKD3,SMAD3,SRC
AMPK Signaling 2.01E00 5.63E-02 NaN
ADRA2A,AK4,AK7,CCND1,CREB3L3,CREBBP,FOXG1,IRS2,PIK3CB,PIK3R3,PPP2R2B,S
IRT1
Small Cell Lung Cancer Signaling 1.99E00 8.45E-02 NaN
ABL1,BCL2,CCND1,MAX,PIK3CB,PIK3R3
Dopamine-DARPP32 Feedback in cAMP Signaling 1.99E00 6.13E-02 NaN

CREB3L3,CREBBP,GUCY1A2,ITPR3,PLCB4,PLCL1,PPP2R2B,PRKD1,PRKD3,PRKG2
Cardiac Hypertrophy Signaling (Enhanced) 1.96E00 4.43E-02 NaN
ACVR1,ACVR2A,ADRA2A,FGFR1,FGFR2,FZD7,ITPR3,MAPKAPK2,NGFR,NRAS,PDE4B,PD
K1,PIK3CB,PIK3R3,PLCB4,PLCL1,PRKD1,PRKD3,TGFBR2,TNFRSF1A,WNT7B,WNT8B

Fcγ Receptor-mediated Phagocytosis in Macrophages and Monocytes
1.95E00 7.45E-02 NaN GAB2,NCK1,PIK3R3,PRKD1,PRKD3,PXN,SRC
Non-Small Cell Lung Cancer Signaling 1.94E00 8.22E-02 NaN
ABL1,CCND1,ITPR3,NRAS,PIK3CB,PIK3R3

Role of PKR in Interferon Induction and Antiviral Response 1.93E00
6.78E-02 NaN ATF3,CASP8,HSP90AA1,IFIH1,METAP2,PDGFA,PDGFD,TNFRSF1A

Neuroinflammation Signaling Pathway 1.92E00 5E-02 NaN
ACVR1,ACVR2A,APH1B,BCL2,CASP8,CREB3L3,CREBBP,GABRA2,GABRG1,GLUL,MFGE8,
PIK3CB,PIK3R3,TGFBR2,TNFRSF1A
Leukocyte Extravasation Signaling 1.91E00 5.7E-02 NaN
ABL1,ARHGAP12,BCAR1,MMP25,PIK3CB,PIK3R3,PRKD1,PRKD3,PXN,RHOH,SRC
Aryl Hydrocarbon Receptor Signaling 1.91E00 6.29E-02 NaN
ALDH9A1,CCND1,HSP90AA1,MGST1,MGST2,NCOR2,NRIP1,RARG,SRC
Endocannabinoid Cancer Inhibition Pathway 1.91E00 6.29E-02 NaN
ATF3,CASP8,CCND1,CREB3L3,CREBBP,PIK3CB,PIK3R3,SRC,TCF7L1
HGF Signaling 1.89E00 6.67E-02 NaN
CCND1,NRAS,PIK3CB,PIK3R3,PRKD1,PRKD3,PXN,RAPGEF1
ATM Signaling 1.88E00 7.22E-02 NaN
ABL1,CREB3L3,CREBBP,MDM4,PPP2R2B,RBBP8,SMC2
Corticotropin Releasing Hormone Signaling 1.87E00 6.21E-02 NaN
CACNA1F,CACNB4,CREB3L3,CREBBP,GLI2,GUCY1A2,ITPR3,PRKD1,PRKD3
Neurotrophin/TRK Signaling 1.86E00 7.89E-02 NaN
CREB3L3,CREBBP,NGFR,NRAS,PIK3CB,PIK3R3
Adrenomedullin signaling pathway 1.85E00 5.58E-02 NaN
BCL2,CEBPB,GUCY1A2,ITPR3,MAX,NRAS,PIK3CB,PIK3R3,PLCB4,PLCL1,PRKG2
Cell Cycle Regulation by BTG Family Proteins 1.82E00 1.08E-01 NaN
BTG2,CCND1,E2F7,PPP2R2B
Notch Signaling 1.82E00 1.08E-01 NaN APH1B,DTX4,NOTCH2,NUMBL
IL-8 Signaling 1.81E00 5.5E-02 NaN
BCL2,CCND1,FNBP1,MAP4K4,NRAS,PIK3CB,PIK3R3,PRKD1,PRKD3,RHOH,SRC
Actin Cytoskeleton Signaling 1.8E00 5.29E-02 NaN
BCAR1,GSN,MYH9,NRAS,PDGFA,PDGFD,PIK3CB,PIK3R3,PIP5K1C,PXN,TRIO,WASF2

VDR/RXR Activation 1.8E00 7.69E-02 NaN
CEBPB,LRP5,NCOR2,PDGFA,PRKD1,PRKD3
T Cell Exhaustion Signaling Pathway 1.79E00 5.71E-02 NaN
ACVR1,ACVR2A,MGAT5,NRAS,PDK1,PIK3CB,PIK3R3,PPP2R2B,SMAD3,TGFBR2
BEX2 Signaling Pathway 1.78E00 7.59E-02 NaN
BCL2,CCND1,NGFR,PLXNC1,PPP2R2B,TCF7L1
Cancer Drug Resistance By Drug Efflux 1.77E00 8.62E-02 NaN
FOXG1,NRAS,PDK1,PIK3CB,PIK3R3
Protein Kinase A Signaling 1.76E00 4.5E-02 NaN
AKAP1,CREB3L3,CREBBP,FLNC,ITPR3,NGFR,PDE4B,PLCB4,PLCL1,PRKD1,PRKD3,PTP
RG,PTPRO,PTPRS,PXN,SMAD3,TCF7L1,TGFBR2
FLT3 Signaling in Hematopoietic Progenitor Cells 1.75E00 7.5E-02 NaN
CREB3L3,CREBBP,GAB2,NRAS,PIK3CB,PIK3R3
Renal Cell Carcinoma Signaling 1.75E00 7.5E-02 NaN
CREBBP,EGLN3,NRAS,PIK3CB,PIK3R3,RAPGEF1
Pyrimidine Deoxyribonucleotides De Novo Biosynthesis I 1.73E00
1.36E-01 NaN AK4,AK7,NME2
Synaptic Long Term Potentiation 1.72E00 6.2E-02 NaN
CREB3L3,CREBBP,ITPR3,NRAS,PLCB4,PLCL1,PRKD1,PRKD3
Neuregulin Signaling 1.71E00 6.67E-02 NaN

HSP90AA1,NRAS,PIK3R3,PRKD1,PRKD3,RNF41,SRC
nNOS Signaling in Skeletal Muscle Cells 1.67E00 9.76E-02 NaN
CACNA1F,CACNB4,DAG1,SNTB1
Regulation Of The Epithelial Mesenchymal Transition In Development Pathway 1.66E00 7.14E-02 NaN APH1B,FZD7,GLI2,TCF7L1,WNT7B,WNT8B

Wnt/Ca+ pathway 1.66E00 8.06E-02 NaN
CREB3L3,CREBBP,FZD7,PLCB4,PLCL1
Paxillin Signaling 1.65E00 6.48E-02 NaN
BCAR1,NCK1,NRAS,PIK3CB,PIK3R3,PXN,SRC
Huntington's Disease Signaling 1.65E00 5.02E-02 NaN
CAPN2,CASP8,CREB3L3,CREBBP,NCOR2,PIK3CB,PIK3R3,PLCB4,PRKD1,PRKD3,RCOR1,STX1A
Phagosome Formation 1.65E00 6.02E-02 NaN
FNBP1,PIK3CB,PIK3R3,PLCB4,PLCL1,PRKD1,PRKD3,RHOH
Intrinsic Prothrombin Activation Pathway 1.64E00 9.52E-02 NaN
COL1A1,COL1A2,COL2A1,KLK10
eNOS Signaling 1.64E00 5.66E-02 NaN
CASP8,GUCY1A2,HSP90AA1,ITPR3,LPAR3,PIK3CB,PIK3R3,PRKD1,PRKD3
Xenobiotic Metabolism AHR Signaling Pathway 1.64E00 7.06E-02 NaN
ALDH9A1,CREBBP,HSP90AA1,MGST1,MGST2,NRIP1
STAT3 Pathway 1.61E00 5.93E-02 NaN
BCL2,FGFR1,FGFR2,NGFR,NRAS,PIM1,SRC,TGFBR2
Th2 Pathway 1.59E00 5.88E-02 NaN
ACVR1,ACVR2A,APH1B,BHLHE41,NOTCH2,PIK3CB,PIK3R3,TGFBR2
Production of Nitric Oxide and Reactive Oxygen Species in Macrophages 1.59E00 5.29E-02 NaN
CREBBP,FNBP1,NGFR,PIK3CB,PIK3R3,PPP2R2B,PRKD1,PRKD3,RHOH,TNFRSF1A
RhoGDI Signaling 1.59E00 5.29E-02 NaN
ARHGAP12,ARHGEF17,ARHGEF18,CREBBP,FNBP1,PIP5K1C,PIP5KL1,RHOH,SRC,WASF2

Glutamine Biosynthesis I 1.59E00 1E00 NaN GLUL
Netrin Signaling 1.58E00 7.69E-02 NaN
CACNA1F,CACNB4,ENAH,NCK1,UNC5C
Phospholipases 1.58E00 7.69E-02 NaN
LPL,PLA2R1,PLAAT5,PLCB4,PLCL1
GPCR-Mediated Nutrient Sensing in Enteroendocrine Cells 1.58E00 6.25E-02 NaN CACNA1F,CACNB4,ITPR3,PLCB4,PLCL1,PRKD1,PRKD3
PI3K Signaling in B Lymphocytes 1.56E00 5.8E-02 NaN
ABL1,ATF3,IRS2,ITPR3,NRAS,PIK3CB,PLCB4,PLCL1
MSP-RON Signaling In Macrophages Pathway 1.56E00 6.19E-02 NaN
CREB3L3,CREBBP,GAB2,KLK10,NRAS,PIK3CB,PIK3R3
Opioid Signaling Pathway 1.55E00 4.86E-02 NaN
CACNA1F,CACNB4,CAMK1D,CREB3L3,CREBBP,ITPR3,NRAS,PDK1,PRKD1,PRKD3,RGS5,SRC
Semaphorin Neuronal Repulsive Signaling Pathway 1.55E00 5.76E-02 NaN
CSPG5,FARP1,GUCY1A2,PDE4B,PIK3CB,PIK3R3,PIP5K1C,PRKG2
NGF Signaling 1.54E00 6.14E-02 NaN
CREB3L3,CREBBP,NGFR,NRAS,PIK3CB,PIK3R3,TRIO
Apelin Liver Signaling Pathway 1.54E00 1.15E-01 NaN

COL1A1,COL1A2,COL2A1
Cell Cycle: G1/S Checkpoint Regulation 1.53E00 7.46E-02 NaN
ABL1,CCND1,E2F7,MAX,SMAD3
Endocannabinoid Developing Neuron Pathway 1.52E00 6.09E-02 NaN
CCND1,CREB3L3,CREBBP,NRAS,PIK3CB,PIK3R3,SRC
Apelin Endothelial Signaling Pathway 1.52E00 6.09E-02 NaN
NRAS,PIK3CB,PIK3R3,PLCB4,PRKD1,PRKD3,SMAD3
fMLP Signaling in Neutrophils 1.5E00 6.03E-02 NaN
ITPR3,NRAS,PIK3CB,PIK3R3,PLCB4,PRKD1,PRKD3
Xenobiotic Metabolism General Signaling Pathway 1.48E00 5.59E-02 NaN
CREBBP,MGST1,MGST2,NRAS,PIK3CB,PIK3R3,PRKD1,PRKD3
GM-CSF Signaling 1.46E00 7.14E-02 NaN
CCND1,NRAS,PIK3CB,PIK3R3,PIM1
ErbB Signaling 1.45E00 6.38E-02 NaN
NCK1,NRAS,PIK3CB,PIK3R3,PRKD1,PRKD3
Erythropoietin Signaling Pathway 1.44E00 5.2E-02 NaN
CCND1,IRS2,ITPR3,NRAS,PIK3CB,PIK3R3,PRKD1,PRKD3,SRC
Cell Cycle: G2/M DNA Damage Checkpoint Regulation 1.42E00 8.16E-02 NaN
ABL1,HIPK2,MDM4,RPRM
ERK/MAPK Signaling 1.42E00 4.95E-02 NaN
BCAR1,CREB3L3,CREBBP,NRAS,PIK3CB,PIK3R3,PPP2R2B,PXN,RAPGEF1,SRC
Melatonin Signaling 1.41E00 6.94E-02 NaN
PLCB4,PLCL1,PRKD1,PRKD3,RORB
Basal Cell Carcinoma Signaling 1.41E00 6.94E-02 NaN
FZD7,GLI2,TCF7L1,WNT7B,WNT8B
Melanoma Signaling 1.4E00 8E-02 NaN
CCND1,NRAS,PIK3CB,PIK3R3
Amyotrophic Lateral Sclerosis Signaling 1.39E00 6.19E-02 NaN
BCL2,CAPN2,GLUL,NEFM,PIK3CB,PIK3R3
Glioma Invasiveness Signaling 1.39E00 6.85E-02 NaN
FNBP1,NRAS,PIK3CB,PIK3R3,RHOH
HIF1α Signaling 1.38E00 4.88E-02 NaN
CAMK1D,CREBBP,EGLN3,HSP90AA1,MMP25,NRAS,PIK3CB,PIK3R3,PRKD1,PRKD3
UVA-Induced MAPK Signaling 1.38E00 6.12E-02 NaN
NRAS,PARP8,PIK3CB,PIK3R3,PLCB4,PLCL1
CCR3 Signaling in Eosinophils 1.37E00 5.65E-02 NaN
ITPR3,NRAS,PIK3CB,PIK3R3,PLCB4,PRKD1,PRKD3
UVC-Induced MAPK Signaling 1.37E00 7.84E-02 NaN
NRAS,PRKD1,PRKD3,SRC
Regulation of Cellular Mechanics by Calpain Protease 1.37E00
6.76E-02 NaN CAPN2,CCND1,NRAS,PXN,SRC
VEGF Signaling 1.36E00 6.06E-02 NaN
BCL2,NRAS,PIK3CB,PIK3R3,PXN,SRC
Apelin Cardiomyocyte Signaling Pathway 1.36E00 6.06E-02 NaN
PIK3CB,PIK3R3,PLCB4,PLCL1,PRKD1,PRKD3
FcγRIIB Signaling in B Lymphocytes 1.35E00 6.67E-02 NaN
CACNA1F,CACNB4,NRAS,PIK3CB,PIK3R3
Phospholipase C Signaling 1.34E00 4.51E-02 NaN
ARHGEF17,ARHGEF18,CREB3L3,CREBBP,FNBP1,ITPR3,NRAS,PLCB4,PRKD1,PRKD3,RH
OH,SRC

UVB-Induced MAPK Signaling	1.34E00	7.69E-02	NaN	PIK3CB,PIK3R3,PRKD1,PRKD3
Apoptosis Signaling	1.34E00	6E-02	NaN	BCL2,CAPN2,CASP8,MAP4K4,NRAS,TNFRSF1A
NF-κB Activation by Viruses	1.33E00	6.58E-02	NaN	NRAS,PIK3CB,PIK3R3,PRKD1,PRKD3
Cardiac Hypertrophy Signaling	1.31E00	4.58E-02	NaN	ADRA2A,CREBBP,FNBP1,MAPKAPK2,NRAS,PIK3CB,PIK3R3,PLCB4,PLCL1,RHOH,TGFBR2
Antiproliferative Role of Somatostatin Receptor 2	1.31E00	6.49E-02	NaN	GUCY1A2,NRAS,PIK3CB,PIK3R3,SRC
Leukotriene Biosynthesis	1.3E00	1.43E-01	NaN	GGT1,MGST2
Cardiolipin Biosynthesis II	1.29E00	5E-01	NaN	PGS1
Mouse Embryonic Stem Cell Pluripotency	1.29E00	5.83E-02	NaN	CREBBP,FZD7,NRAS,PIK3CB,PIK3R3,TCF7L1
IL-7 Signaling Pathway	1.29E00	6.41E-02	NaN	BCL2,CCND1,FOXG1,PIK3CB,PIK3R3
Gαq Signaling	1.28E00	5.1E-02	NaN	FNBP1,ITPR3,PIK3CB,PIK3R3,PLCB4,PRKD1,PRKD3,RHOH
Circadian Rhythm Signaling	1.27E00	9.09E-02	NaN	BHLHE41,CREB3L3,CREBBP
IL-9 Signaling	1.27E00	9.09E-02	NaN	IRS2,PIK3CB,PIK3R3
Pyrimidine Ribonucleotides Interconversion	1.27E00	9.09E-02	NaN	AK4,AK7,NME2
EGF Signaling	1.27E00	7.27E-02	NaN	ITPR3,PIK3CB,PIK3R3,SRC
Cellular Effects of Sildenafil (Viagra)	1.26E00	5.34E-02	NaN	GUCY1A2,ITPR3,MYH9,PDE4B,PLCB4,PLCL1,PRKG2
Gα12/13 Signaling	1.26E00	5.34E-02	NaN	F2RL2,LPAR3,NRAS,PIK3CB,PIK3R3,PXN,SRC
Inhibition of Angiogenesis by TSP1	1.24E00	8.82E-02	NaN	GUCY1A2,HSPG2,TGFBR2
Telomerase Signaling	1.22E00	5.61E-02	NaN	ABL1,HSP90AA1,NRAS,PIK3CB,PIK3R3,PPP2R2B
Pyrimidine Ribonucleotides De Novo Biosynthesis	1.21E00	8.57E-02	NaN	AK4,AK7,NME2
CREB Signaling in Neurons	1.21E00	3.69E-02	NaN	ADRA2A,CACNA1F,CACNB4,CREB3L3,CREBBP,F2RL2,FGFR1,FGFR2,FZD7,GLP1R,HTR1B,ITPR3,LPAR3,NGFR,NRAS,PIK3CB,PIK3R3,PLCB4,PLCL1,PRKD1,PRKD3,TGFBR2
Xenobiotic Metabolism PXR Signaling Pathway	1.2E00	4.69E-02	NaN	ALDH9A1,CREBBP,HSP90AA1,MGST1,MGST2,NCOR2,NRIP1,PRKD1,PRKD3
LPS-stimulated MAPK Signaling	1.19E00	6.02E-02	NaN	NRAS,PIK3CB,PIK3R3,PRKD1,PRKD3
HER-2 Signaling in Breast Cancer	1.18E00	4.64E-02	NaN	CCND1,NRAS,PDK1,PIK3CB,PIK3R3,PRKD1,PRKD3,SMAD3,SRC
VEGF Family Ligand-Receptor Interactions	1.17E00	5.95E-02	NaN	NRAS,PIK3CB,PIK3R3,PRKD1,PRKD3
3-phosphoinositide Biosynthesis	1.17E00	4.82E-02	NaN	PI4K2A,PIK3CB,PIK3R3,PIP5K1C,PIP5KL1,PPFIBP2,PTPRO,RNGTT
LPS/IL-1 Mediated Inhibition of RXR Function	1.16E00	4.44E-02	NaN	

ABCA1,ALDH9A1,FMO4,MGMT,MGST1,MGST2,NGFR,SLC27A1,SOD3,TNFRSF1A

BMP signaling pathway 1.16E00 5.88E-02 NaN
CREBBP,MAGED1,NRAS,PITX2,SOSTDC1
Endometrial Cancer Signaling 1.16E00 6.67E-02 NaN
CCND1,NRAS,PIK3CB,PIK3R3
Retinoic acid Mediated Apoptosis Signaling 1.16E00 6.67E-02 NaN
CASP8,CRABP2,PARP8,RARG
Induction of Apoptosis by HIV1 1.13E00 6.56E-02 NaN
BCL2,CASP8,NGFR,TNFRSF1A
Docosahexaenoic Acid (DHA) Signaling 1.13E00 7.89E-02 NaN
BCL2,PIK3CB,PIK3R3
Superpathway of Inositol Phosphate Compounds 1.12E00 4.52E-02 NaN
PI4K2A,PIK3CB,PIK3R3,PIP5K1C,PIP5KL1,PLCB4,PPFIBP2,PTPRO,RNGTT

L-carnitine Biosynthesis 1.12E00 3.33E-01 NaN ALDH9A1
1,25-dihydroxyvitamin D3 Biosynthesis 1.12E00 3.33E-01 NaN POR

Anandamide Degradation 1.12E00 3.33E-01 NaN NAAA
Glycerol-3-phosphate Shuttle 1.12E00 3.33E-01 NaN GPD2
Th1 and Th2 Activation Pathway 1.11E00 4.68E-02 NaN
ACVR1,ACVR2A,APH1B,BHLHE41,NOTCH2,PIK3CB,PIK3R3,TGFBR2
Renin-Angiotensin Signaling 1.06E00 5.08E-02 NaN
ITPR3,NRAS,PIK3CB,PIK3R3,PRKD1,PRKD3
p38 MAPK Signaling 1.06E00 5.08E-02 NaN
CREB3L3,CREBBP,MAPKAPK2,MAX,TGFBR2,TNFRSF1A
ErbB2-ErbB3 Signaling 1.06E00 6.15E-02 NaN
CCND1,NRAS,PIK3CB,PIK3R3
Calcium Signaling 1.05E00 4.37E-02 NaN
CACNA1F,CACNB4,CAMK1D,CREB3L3,CREBBP,ITPR3,MICU1,MYH9,TRPC4
Breast Cancer Regulation by Stathmin1 1.05E00 3.56E-02 NaN
ADRA2A,ARHGEF17,ARHGEF18,CAMK1D,CCND1,CREB3L3,CREBBP,E2F7,F2RL2,FZD7,G
LP1R,HTR1B,LPAR3,NRAS,PIK3CB,PIK3R3,PLCB4,PPP2R2B,PRKD1,PRKD3,UHMK1
Death Receptor Signaling 1.04E00 5.43E-02 NaN
BCL2,CASP8,MAP4K4,PARP8,TNFRSF1A
Calcium-induced T Lymphocyte Apoptosis 1.04E00 6.06E-02 NaN
CAPN2,ITPR3,PRKD1,PRKD3
Estrogen Biosynthesis 1.02E00 7.14E-02 NaN CYP2S1,HSD17B14,POR

LXR/RXR Activation 1.02E00 4.96E-02 NaN
ABCA1,LPL,NCOR2,NGFR,SCD,TNFRSF1A
IL-15 Production 1.02E00 4.96E-02 NaN
ABL1,EPHA3,FGFR1,FGFR2,PTK7,SRC
Coronavirus Pathogenesis Pathway 1.02E00 4.67E-02 NaN
ABL1,BCL2,CASP8,CCND1,E2F7,SMAD3,TGFBR2
mTOR Signaling 1.01E00 4.29E-02 NaN
EIF3D,FNBP1,NRAS,PIK3CB,PIK3R3,PPP2R2B,PRKD1,PRKD3,RHOH
Reelin Signaling in Neurons 1.01E00 4.92E-02 NaN
MAP1B,PDK1,PIK3CB,PIK3R3,RAPGEF1,SRC
G Beta Gamma Signaling 1.01E00 4.92E-02 NaN

CACNA1F,CACNB4,NRAS,PRKD1,PRKD3,SRC
Nur77 Signaling in T Lymphocytes 1E00 5.88E-02 NaN
BCL2,PDK1,PRKD1,PRKD3
α-Adrenergic Signaling 9.96E-01 5.26E-02 NaN
ADRA2A,ITPR3,NRAS,PRKD1,PRKD3
GABA Receptor Signaling 9.96E-01 5.26E-02 NaN
ALDH9A1,CACNA1F,CACNB4,GABRA2,GABRG1
G-Protein Coupled Receptor Signaling 9.96E-01 4.01E-02 NaN
ADRA2A,CREB3L3,CREBBP,GLP1R,HTR1B,NRAS,PDE4B,PIK3CB,PIK3R3,PLCB4,SRC

Systemic Lupus Erythematosus In B Cell Signaling Pathway 9.87E-01
4E-02 NaN
BCL2,CCND1,FOXG1,IFIH1,MAP4K4,NRAS,PIK3CB,PIK3R3,PRKD1,PRKD3,SRC
Role of JAK1 and JAK3 in γc Cytokine Signaling 9.83E-01 5.8E-02 NaN
IRS2,NRAS,PIK3CB,PIK3R3
Role of RIG1-like Receptors in Antiviral Innate Immunity 9.79E-01
6.82E-02 NaN CASP8,CREBBP,IFIH1
PI3K/AKT Signaling 9.75E-01 4.35E-02 NaN
BCL2,CCND1,GAB2,HSP90AA1,NRAS,PIK3CB,PIK3R3,PPP2R2B
Agrin Interactions at Neuromuscular Junction 9.67E-01 5.71E-02 NaN
DAG1,NRAS,PXN,SRC
Salvage Pathways of Pyrimidine Ribonucleotides 9.55E-01 5.1E-02 NaN
ACVR2A,AK4,AK7,NME2,PIM1
Growth Hormone Signaling 9.51E-01 5.63E-02 NaN
PIK3CB,PIK3R3,PRKD1,PRKD3
Synaptogenesis Signaling Pathway 9.43E-01 3.85E-02 NaN
CACNB4,CREB3L3,CREBBP,EPHA3,FARP1,MAP1B,NRAS,PIK3CB,PIK3R3,RAPGEF1,SRC
,STX1A
Role of Oct4 in Mammalian Embryonic Stem Cell Pluripotency 9.36E-01
6.52E-02 NaN CDX2,IGF2BP1,SALL4
PFKFB4 Signaling Pathway 9.36E-01 6.52E-02 NaN CREB3L3,CREBBP,XDH

ERK5 Signaling 9.32E-01 5.56E-02 NaN CREB3L3,CREBBP,NRAS,SRC
Tryptophan Degradation III (Eukaryotic) 9.24E-01 8.7E-02 NaN
CYP2S1,HAAO
GPCR-Mediated Integration of Enteroendocrine Signaling Exemplified by
an L Cell 9.17E-01 5.48E-02 NaN GLP1R,ITPR3,PLCB4,PLCL1
nNOS Signaling in Neurons 9.14E-01 6.38E-02 NaN CAPN2,PRKD1,PRKD3

Protein Citrullination 9.1E-01 2E-01 NaN PADI2
Creatine-phosphate Biosynthesis 9.1E-01 2E-01 NaN MAP4K4
Myo-inositol Biosynthesis 9.1E-01 2E-01 NaN ISYNA1
Lysine Degradation V 9.1E-01 2E-01 NaN PIPOX
Leptin Signaling in Obesity 9.03E-01 5.41E-02 NaN
PIK3CB,PIK3R3,PLCB4,PLCL1
Hypoxia Signaling in the Cardiovascular System 9.03E-01 5.41E-02 NaN
CREB3L3,CREBBP,HSP90AA1,UBE2H
SAPK/JNK Signaling 9E-01 4.9E-02 NaN
MAP4K2,MAP4K4,NRAS,PIK3CB,PIK3R3
Glutathione Redox Reactions I 8.96E-01 8.33E-02 NaN MGST1,MGST2

Phosphatidylglycerol Biosynthesis II (Non-plastidic) 8.96E-01
8.33E-02 NaN AGPAT5,PGS1
Regulation of Actin-based Motility by Rho 8.89E-01 4.85E-02 NaN
FNBP1,GSN,PIP5K1C,PIP5KL1,RHOH
Sumoylation Pathway 8.89E-01 4.85E-02 NaN
CREBBP,FNBP1,RCOR1,RHOH,SIRT1
Clathrin-mediated Endocytosis Signaling 8.86E-01 4.15E-02 NaN
PDGFA,PDGFD,PIK3CB,PIK3R3,PIP5K1C,SH3BP4,SRC,STON2
Angiopoietin Signaling 8.86E-01 5.33E-02 NaN
NCK1,NRAS,PIK3CB,PIK3R3
IGF-1 Signaling 8.76E-01 4.81E-02 NaN IRS2,NRAS,PIK3CB,PIK3R3,PXN

Bupropion Degradation 8.66E-01 8E-02 NaN CYP2S1,POR
HMGB1 Signaling 8.6E-01 4.24E-02 NaN
FNBP1,NGFR,NRAS,PIK3CB,PIK3R3,RHOH,TNFRSF1A
TNFR1 Signaling 8.57E-01 6E-02 NaN CASP8,MAP4K2,TNFRSF1A
Natural Killer Cell Signaling 8.51E-01 4.06E-02 NaN
COL1A1,COL1A2,COL2A1,NCK1,NRAS,PIK3CB,PIK3R3,PXN
PD-1, PD-L1 cancer immunotherapy pathway 8.51E-01 4.72E-02 NaN
NGFR,PIK3CB,PIK3R3,SMAD3,TNFRSF1A
cAMP-mediated signaling 8.48E-01 3.93E-02 NaN
ADRA2A,AKAP1,CAMK1D,CREB3L3,CREBBP,GLP1R,HTR1B,PDE4B,SRC
Role of Wnt/GSK-3β Signaling in the Pathogenesis of Influenza
8.42E-01 5.13E-02 NaN FZD7,TCF7L1,WNT7B,WNT8B
Estrogen-mediated S-phase Entry 8.39E-01 7.69E-02 NaN CCND1,E2F7

Glycerol Degradation I 8.36E-01 1.67E-01 NaN GPD2
Tryptophan Degradation to 2-amino-3-carboxymuconate Semialdehyde
8.36E-01 1.67E-01 NaN HAAO
Tight Junction Signaling 8.33E-01 4.17E-02 NaN
F2RL2,MYH9,NGFR,PPP2R2B,TGFBR2,TJP1,TNFRSF1A
Insulin Receptor Signaling 8.15E-01 4.32E-02 NaN
IRS2,NCK1,NRAS,PIK3CB,PIK3R3,RAPGEF1
Role of MAPK Signaling in the Pathogenesis of Influenza 8.12E-01
5E-02 NaN BCL2,NRAS,PLA2R1,PLAAT5
JAK/Stat Signaling 8.12E-01 5E-02 NaN
CEBPB,NRAS,PIK3CB,PIK3R3
Chemokine Signaling 8.12E-01 5E-02 NaN
CAMK1D,NRAS,PLCB4,SRC
Role of MAPK Signaling in Promoting the Pathogenesis of Influenza
8.04E-01 4.55E-02 NaN ATP6V1H,BCL2,NRAS,PLA2R1,PLAAT5
Lymphotoxin β Receptor Signaling 8.01E-01 5.66E-02 NaN
CREBBP,PIK3CB,PIK3R3
Cyclins and Cell Cycle Regulation 8.01E-01 4.94E-02 NaN
ABL1,CCND1,E2F7,PPP2R2B
Actin Nucleation by ARP-WASP Complex 8.01E-01 4.94E-02 NaN
FNBP1,NCK1,NRAS,RHOH
Tec Kinase Signaling 7.88E-01 4.05E-02 NaN
FNBP1,PIK3CB,PIK3R3,PRKD1,PRKD3,RHOH,SRC

Transcriptional Regulatory Network in Embryonic Stem Cells	7.85E-01	5.56E-02	NaN	CDX2,TCF7L1,ZFHX3
Glycoaminoglycan-protein Linkage Region Biosynthesis	7.75E-01	1.43E-01	NaN	XYLT1
Role of IL-17A in Arthritis	7.7E-01	5.45E-02	NaN	MAPKAPK2,PIK3CB,PIK3R3
Role of p14/p19ARF in Tumor Suppression	7.62E-01	6.9E-02	NaN	PIK3CB,PIK3R3
CNTF Signaling	7.38E-01	5.26E-02	NaN	NRAS,PIK3CB,PIK3R3
Fc Epsilon RI Signaling	7.28E-01	4.27E-02	NaN	NRAS,PIK3CB,PIK3R3,PRKD1,PRKD3
Acute Phase Response Signaling	7.28E-01	3.89E-02	NaN	CEBPB,CRABP2,NGFR,NRAS,PIK3CB,PIK3R3,TNFRSF1A
Superoxide Radicals Degradation	7.24E-01	1.25E-01	NaN	SOD3
MSP-RON Signaling Pathway	7.21E-01	5.17E-02	NaN	KLK10,PIK3CB,PIK3R3
G Protein Signaling Mediated by Tubby	7.19E-01	6.45E-02	NaN	ABL1,PLCB4
Acetone Degradation I (to Methylglyoxal)	7.19E-01	6.45E-02	NaN	CYP2S1,POR
RANK Signaling in Osteoclasts	6.99E-01	4.49E-02	NaN	GSN,PIK3CB,PIK3R3,SRC
Glutathione-mediated Detoxification	6.97E-01	6.25E-02	NaN	MGST1,MGST2
PCP pathway	6.93E-01	5E-02	NaN	FZD7,WNT7B,WNT8B
SPINK1 Pancreatic Cancer Pathway	6.93E-01	5E-02	NaN	KLK10,SMAD3,TGFBR2
Rac Signaling	6.88E-01	4.13E-02	NaN	NRAS,PIK3CB,PIK3R3,PIP5K1C,PIP5KL1
B Cell Receptor Signaling	6.8E-01	3.76E-02	NaN	ABL1,CREB3L3,CREBBP,GAB2,NRAS,PIK3CB,PIK3R3
Sucrose Degradation V (Mammalian)	6.78E-01	1.11E-01	NaN	KHK
Fatty Acid β-oxidation I	6.78E-01	6.06E-02	NaN	ECHDC3,SLC27A1
IL-2 Signaling	6.78E-01	4.92E-02	NaN	NRAS,PIK3CB,PIK3R3
Cytotoxic T Lymphocyte-mediated Apoptosis of Target Cells	6.58E-01	5.88E-02	NaN	BCL2,CASP8
Role of JAK2 in Hormone-like Cytokine Signaling	6.58E-01	5.88E-02	NaN	IRS2,PRLR
Necroptosis Signaling Pathway	6.52E-01	3.82E-02	NaN	CAPN2,CASP8,GLUL,NGFR,RBCK1,TNFRSF1A
CCR5 Signaling in Macrophages	6.44E-01	4.26E-02	NaN	CACNA1F,CACNB4,PRKD1,PRKD3
Embryonic Stem Cell Differentiation into Cardiac Lineages	6.36E-01	1E-01	NaN	SP4
Prostanoid Biosynthesis	6.36E-01	1E-01	NaN	CYP2S1
Glycine Betaine Degradation	6.36E-01	1E-01	NaN	PIPOX
CD40 Signaling	6.25E-01	4.62E-02	NaN	MAPKAPK2,PIK3CB,PIK3R3
Nicotine Degradation II	6.25E-01	4.62E-02	NaN	CYP2S1,FMO4,POR
Sertoli Cell-Sertoli Cell Junction Signaling	6.22E-01	3.61E-02	NaN	BCAR1,GUCY1A2,NRAS,PRKG2,SRC,TJP1,TNFRSF1A

Bladder Cancer Signaling	6.14E-01	4.12E-02	NaN	ABL1,CCND1,MMP25,NRAS
Mitotic Roles of Polo-Like Kinase	6.13E-01	4.55E-02	NaN	FBXO5,HSP90AA1,PPP2R2B
Eicosanoid Signaling	6.13E-01	4.55E-02	NaN	GGT1,PLA2R1,PLAAT5
γ-glutamyl Cycle	6E-01	9.09E-02	NaN	GGT1
IL-12 Signaling and Production in Macrophages	5.82E-01	3.76E-02	NaN	CEBPB,PIK3CB,PIK3R3,PRKD1,PRKD3
SPINK1 General Cancer Pathway	5.77E-01	4.35E-02	NaN	NRAS,PIK3CB,PIK3R3
Antiproliferative Role of TOB in T Cell Signaling	5.72E-01	5.13E-02	NaN	SMAD3,TGFBR2
Inhibition of Matrix Metalloproteases	5.72E-01	5.13E-02	NaN	HSPG2,MMP25
Hematopoiesis from Multipotent Stem Cells	5.69E-01	8.33E-02	NaN	KITLG
NAD biosynthesis II (from tryptophan)	5.69E-01	8.33E-02	NaN	HAAO
Iron homeostasis signaling pathway	5.5E-01	3.65E-02	NaN	ATP6V1H,CREB3L3,PDGFA,SLC25A37,SMAD3
Mitochondrial Dysfunction	5.48E-01	3.51E-02	NaN	APH1B,BCL2,CASP8,GPD2,NDUFB4,XDH
Mechanisms of Viral Exit from Host Cells	5.42E-01	4.88E-02	NaN	PRKD1,PRKD3
Assembly of RNA Polymerase III Complex	5.39E-01	7.69E-02	NaN	GTF3A
Fatty Acid Activation	5.39E-01	7.69E-02	NaN	SLC27A1
Oleate Biosynthesis II (Animals)	5.39E-01	7.69E-02	NaN	SCD
Guanosine Nucleotides Degradation III	5.39E-01	7.69E-02	NaN	XDH
Caveolar-mediated Endocytosis Signaling	5.33E-01	4.11E-02	NaN	ABL1,FLNC,SRC
T Helper Cell Differentiation	5.33E-01	4.11E-02	NaN	NGFR,TGFBR2,TNFRSF1A
Hereditary Breast Cancer Signaling	5.27E-01	3.57E-02	NaN	CCND1,CREBBP,NRAS,PIK3CB,PIK3R3
Gαs Signaling	5.21E-01	3.74E-02	NaN	CREB3L3,CREBBP,GLP1R,SRC
CDK5 Signaling	5.13E-01	3.7E-02	NaN	ABL1,NGFR,NRAS,PPP2R2B
Role of IL-17F in Allergic Inflammatory Airway Diseases	5.13E-01	4.65E-02	NaN	CREB3L3,CREBBP
BAG2 Signaling Pathway	5.13E-01	4.65E-02	NaN	HSP90AA1,MAPKAPK2
IL-15 Signaling	5.11E-01	4E-02	NaN	NRAS,PIK3CB,PIK3R3
DNA Double-Strand Break Repair by Homologous Recombination	5.11E-01	7.14E-02	NaN	ABL1
Glycogen Degradation III	5.11E-01	7.14E-02	NaN	GAA
Urate Biosynthesis/Inosine 5'-phosphate Degradation	5.11E-01	7.14E-02	NaN	XDH
Antioxidant Action of Vitamin C	5.04E-01	3.67E-02	NaN	

PLA2R1,PLAAT5,PLCB4,PLCL1
Apelin Pancreas Signaling Pathway 5E-01 4.55E-02 NaN
PIK3CB,PIK3R3
IL-23 Signaling Pathway 5E-01 4.55E-02 NaN PIK3CB,PIK3R3
iCOS-iCOSL Signaling in T Helper Cells 4.89E-01 3.6E-02 NaN
GAB2,ITPR3,PIK3CB,PIK3R3
Type I Diabetes Mellitus Signaling 4.89E-01 3.6E-02 NaN
BCL2,CASP8,NGFR,TNFRSF1A
Signaling by Rho Family GTPases 4.76E-01 3.16E-02 NaN
ARHGEF17,ARHGEF18,FNBP1,PIK3CB,PIK3R3,PIP5K1C,PIP5KL1,RHOH
Heparan Sulfate Biosynthesis 4.74E-01 3.8E-02 NaN
EXT1,EXTL3,XYLT1
Granzyme B Signaling 4.65E-01 6.25E-02 NaN CASP8
Glutaryl-CoA Degradation 4.65E-01 6.25E-02 NaN CYP2S1
Androgen Biosynthesis 4.65E-01 6.25E-02 NaN HSD17B14
Adenosine Nucleotides Degradation II 4.65E-01 6.25E-02 NaN XDH

Role of Tissue Factor in Cancer 4.51E-01 3.45E-02 NaN
NRAS,PIK3CB,PIK3R3,SRC
Isoleucine Degradation I 4.44E-01 5.88E-02 NaN ECHDC3
γ-linolenate Biosynthesis II (Animals) 4.44E-01 5.88E-02 NaN
SLC27A1
Dermatan Sulfate Degradation (Metazoa) 4.44E-01 5.88E-02 NaN IDS

Mitochondrial L-carnitine Shuttle Pathway 4.44E-01 5.88E-02 NaN
SLC27A1
Histamine Degradation 4.44E-01 5.88E-02 NaN ALDH9A1
EIF2 Signaling 4.42E-01 3.12E-02 NaN
ATF3,BCL2,CCND1,EIF3D,NRAS,PIK3CB,PIK3R3
Systemic Lupus Erythematosus In T Cell Signaling Pathway 4.37E-01
2.99E-02 NaN
CASP8,CREB3L3,CREBBP,FNBP1,NRAS,PDK1,PIK3CB,PIK3R3,PPP2R2B,RHOH
Role of Pattern Recognition Receptors in Recognition of Bacteria and
Viruses 4.33E-01 3.25E-02 NaN IFIH1,PIK3CB,PIK3R3,PRKD1,PRKD3
Amyloid Processing 4.28E-01 4E-02 NaN APH1B,CAPN2
PKCθ Signaling in T Lymphocytes 4.27E-01 3.23E-02 NaN
CACNA1F,CACNB4,NRAS,PIK3CB,PIK3R3
IL-4 Signaling 4.21E-01 3.53E-02 NaN NRAS,PIK3CB,PIK3R3
Th1 Pathway 4.17E-01 3.31E-02 NaN APH1B,NOTCH2,PIK3CB,PIK3R3

Inhibition of ARE-Mediated mRNA Degradation Pathway 4.1E-01
3.28E-02 NaN MAPKAPK2,NGFR,PPP2R2B,TNFRSF1A
Granzyme A Signaling 4.06E-01 5.26E-02 NaN CREBBP
GADD45 Signaling 4.06E-01 5.26E-02 NaN CCND1
Purine Nucleotides Degradation II (Aerobic) 4.06E-01 5.26E-02 NaN
XDH
Oxidative Ethanol Degradation III 4.06E-01 5.26E-02 NaN ALDH9A1
RhoA Signaling 4.03E-01 3.25E-02 NaN
ARHGAP12,LPAR3,PIP5K1C,PIP5KL1
CTLA4 Signaling in Cytotoxic T Lymphocytes 3.9E-01 3.37E-02 NaN

PIK3CB,PIK3R3,PPP2R2B
Gαi Signaling 3.9E-01 3.2E-02 NaN ADRA2A,HTR1B,NRAS,SRC
Valine Degradation I 3.89E-01 5E-02 NaN ECHDC3
Fatty Acid α-oxidation 3.89E-01 5E-02 NaN ALDH9A1
Inflammasome pathway 3.89E-01 5E-02 NaN CASP8
Protein Ubiquitination Pathway 3.85E-01 2.93E-02 NaN
DNAJB12,DNAJC15,HSP90AA1,UBE2H,UBE3B,USP13,USP2,USP46
Regulation of IL-2 Expression in Activated and Anergic T Lymphocytes
3.83E-01 3.33E-02 NaN NRAS,SMAD3,TGFBR2
Putrescine Degradation III 3.73E-01 4.76E-02 NaN ALDH9A1
Endocannabinoid Neuronal Synapse Pathway 3.73E-01 3.12E-02 NaN
CACNA1F,CACNB4,PLCB4,PLCL1
Unfolded protein response 3.69E-01 3.57E-02 NaN BCL2,CEBPB
Regulation of eIF4 and p70S6K Signaling 3.67E-01 3.01E-02 NaN
EIF3D,NRAS,PIK3CB,PIK3R3,PPP2R2B
Role of CHK Proteins in Cell Cycle Checkpoint Control 3.6E-01
3.51E-02 NaN E2F7,PPP2R2B
Nicotine Degradation III 3.6E-01 3.51E-02 NaN CYP2S1,POR
CDP-diacylglycerol Biosynthesis I 3.58E-01 4.55E-02 NaN AGPAT5
Methionine Degradation I (to Homocysteine) 3.58E-01 4.55E-02 NaN
MGMT
Polyamine Regulation in Colon Cancer 3.44E-01 4.35E-02 NaN MAX

Ethanol Degradation IV 3.44E-01 4.35E-02 NaN ALDH9A1
Semaphorin Signaling in Neurons 3.34E-01 3.33E-02 NaN FNBP1,RHOH

Maturity Onset Diabetes of Young (MODY) Signaling 3.34E-01 3.33E-02 NaN
ABCC8,CREBBP
Melatonin Degradation I 3.34E-01 3.33E-02 NaN CYP2S1,POR
Tumoricidal Function of Hepatic Natural Killer Cells 3.31E-01
4.17E-02 NaN CASP8
Cysteine Biosynthesis III (mammalia) 3.31E-01 4.17E-02 NaN
MGMT
Vitamin-C Transport 3.31E-01 4.17E-02 NaN LRRC8D
Tryptophan Degradation X (Mammalian, via Tryptamine) 3.18E-01
4E-02 NaN ALDH9A1
Activation of IRF by Cytosolic Pattern Recognition Receptors
3.12E-01 3.17E-02 NaN CREBBP,IFIH1
Kinetochore Metaphase Signaling Pathway 3.12E-01 2.97E-02 NaN
CENPE,CENPN,ZWILCH
Glycolysis I 3.05E-01 3.85E-02 NaN ENO1
Gluconeogenesis I 3.05E-01 3.85E-02 NaN ENO1
Role of PI3K/AKT Signaling in the Pathogenesis of Influenza 3.04E-01
3.12E-02 NaN PIK3CB,PIK3R3
Role of NFAT in Regulation of the Immune Response 2.97E-01 2.76E-02 NaN
ITPR3,NRAS,PIK3CB,PIK3R3,PLCB4
IL-17A Signaling in Airway Cells 2.97E-01 3.08E-02 NaN
PIK3CB,PIK3R3
Superpathway of Melatonin Degradation 2.97E-01 3.08E-02 NaN
CYP2S1,POR

Pyridoxal 5'-phosphate Salvage Pathway	2.9E-01	3.03E-02	NaN	ACVR2A,PIM1
T Cell Receptor Signaling	2.84E-01	2.83E-02	NaN	NRAS,PIK3CB,PIK3R3
Remodeling of Epithelial Adherens Junctions	2.77E-01	2.94E-02	NaN	EXOC2,SRC
Hepatic Cholestasis	2.76E-01	2.69E-02	NaN	MAP4K2,NGFR,PRKD1,PRKD3,TNFRSF1A
Sonic Hedgehog Signaling	2.73E-01	3.45E-02	NaN	GLI2
IL-17 Signaling	2.72E-01	2.67E-02	NaN	CEBPB,HSP90AA1,NRAS,PIK3CB,PIK3R3
Dopamine Degradation	2.64E-01	3.33E-02	NaN	ALDH9A1
Relaxin Signaling	2.58E-01	2.65E-02	NaN	GUCY1A2,PDE4B,PIK3CB,PIK3R3
Heparan Sulfate Biosynthesis (Late Stages)	2.52E-01	2.78E-02	NaN	EXT1,EXTL3
Ethanol Degradation II	2.46E-01	3.12E-02	NaN	ALDH9A1
Role of MAPK Signaling in Inhibiting the Pathogenesis of Influenza	2.35E-01	2.67E-02	NaN	PLA2R1,PLAAT5
TWEAK Signaling	2.21E-01	2.86E-02	NaN	CASP8
Noradrenaline and Adrenaline Degradation	2.21E-01	2.86E-02	NaN	ALDH9A1
IL-17A Signaling in Fibroblasts	2.13E-01	2.78E-02	NaN	CEBPB
Interferon Signaling	2.13E-01	2.78E-02	NaN	BCL2
Superpathway of Methionine Degradation	2.06E-01	2.7E-02	NaN	MGMT
Glucocorticoid Receptor Signaling	-0E00	2.38E-02	NaN	BCL2,CREBBP,HSP90AA1,NCOR2,NRAS,NRIP1,PIK3CB,PIK3R3,SMAD3,SRC,TGFBR2
IL-10 Signaling	-0E00	1.43E-02	NaN	MAP4K4
FXR/RXR Activation	-0E00	1.59E-02	NaN	CREBBP,LPL
PXR/RXR Activation	-0E00	1.54E-02	NaN	SCD
Role of BRCA1 in DNA Damage Response	-0E00	2.5E-02	NaN	E2F7,RBBP8
CD27 Signaling in Lymphocytes	-0E00	1.89E-02	NaN	CASP8
Oncostatin M Signaling	-0E00	2.33E-02	NaN	NRAS
CD28 Signaling in T Helper Cells	-0E00	2.48E-02	NaN	ITPR3,PIK3CB,PIK3R3
Systemic Lupus Erythematosus Signaling	-0E00	1.75E-02	NaN	NRAS,PIK3CB,PIK3R3,PRPF18
Cdc42 Signaling	-0E00	1.14E-02	NaN	EXOC2,SRC
Neuroprotective Role of THOP1 in Alzheimer's Disease	-0E00	8.62E-03	NaN	KLK10
OX40 Signaling Pathway	-0E00	1.11E-02	NaN	BCL2
Cell Cycle Control of Chromosomal Replication	-0E00	1.79E-02	NaN	PRIM2
Spliceosomal Cycle	-0E00	2.08E-02	NaN	PRPF18
Hematopoiesis from Pluripotent Stem Cells	-0E00	2.04E-02	NaN	KITLG
iNOS Signaling	-0E00	2.22E-02	NaN	CREBBP

Ephrin B Signaling	-0E00	1.39E-02	NaN	PXN
tRNA Splicing	-0E00	2.27E-02	NaN	PDE4B
D-myo-inositol-5-phosphate Metabolism	-0E00	2.55E-02	NaN	PLCB4,PPFIBP2,PTPRO,RNGTT
Triacylglycerol Degradation	-0E00	2E-02	NaN	LPL
Chondroitin Sulfate Biosynthesis	-0E00	1.79E-02	NaN	XYLT1
Dermatan Sulfate Biosynthesis	-0E00	1.69E-02	NaN	XYLT1
D-myo-inositol (1,4,5,6)-Tetrakisphosphate Biosynthesis	-0E00	2.11E-02	NaN	PPFIBP2,PTPRO,RNGTT
Serotonin Degradation	-0E00	1.49E-02	NaN	ALDH9A1
D-myo-inositol (3,4,5,6)-tetrakisphosphate Biosynthesis	-0E00	2.11E-02	NaN	PPFIBP2,PTPRO,RNGTT
3-phosphoinositide Degradation	-0E00	1.92E-02	NaN	PPFIBP2,PTPRO,RNGTT
Retinol Biosynthesis	-0E00	2.27E-02	NaN	LPL
Triacylglycerol Biosynthesis	-0E00	2.27E-02	NaN	AGPAT5
Stearate Biosynthesis I (Animals)	-0E00	2.04E-02	NaN	SLC27A1
Agranulocyte Adhesion and Diapedesis	-0E00	2.07E-02	NaN	GLG1,MMP25,MYH9,TNFRSF1A
Granulocyte Adhesion and Diapedesis	-0E00	2.31E-02	NaN	GLG1,MMP25,NGFR,TNFRSF1A
Oxidative Phosphorylation	-0E00	9.17E-03	NaN	NDUFB4
HIPPO signaling	-0E00	2.35E-02	NaN	PPP2R2B,SMAD3
Toll-like Receptor Signaling	-0E00	1.32E-02	NaN	MAP4K4
Cardiac β-adrenergic Signaling	-0E00	2.11E-02	NaN	AKAP1,PDE4B,PPP2R2B
Serotonin Receptor Signaling	-0E00	2.33E-02	NaN	HTR1B
Phototransduction Pathway	-0E00	1.92E-02	NaN	GUCY1A2
Dopamine Receptor Signaling	-0E00	1.3E-02	NaN	PPP2R2B
Glutamate Receptor Signaling	-0E00	1.75E-02	NaN	GLUL
Gustation Pathway	-0E00	2.58E-02	NaN	CACNA1F,CACNB4,ITPR3,PDE4B
Phagosome Maturation	-0E00	1.99E-02	NaN	ATP6V1H,DYNC1H1,DYNC2H1
Autophagy	-0E00	1.64E-02	NaN	BCL2
Sirtuin Signaling Pathway	-0E00	2.06E-02	NaN	ABCA1,NDUFB4,PDK1,RBBP8,SIRT1,SOD3
Th17 Activation Pathway	-0E00	1.1E-02	NaN	HSP90AA1
NER Pathway	-0E00	9.71E-03	NaN	PRIM2
Apelin Adipocyte Signaling Pathway	-0E00	2.44E-02	NaN	MGST1,MGST2
Ferroptosis Signaling Pathway	-0E00	1.59E-02	NaN	ABCA1,NRAS
