# Supplementary material for: Inability to switch from ARID1A-BAF to ARID1B-BAF impairs exit from pluripotency and commitment towards neural crest formation in ARID1B-related neurodevelopmental disorders
Source: Nat Commun. 2021 Nov 9;12:6469. doi: 10.1038/s41467-021-26810-x (PMC8578637; doi:10.1038/s41467-021-26810-x)
Supplement: Supplementary file 8 — Supplementary Data 5 [file 41467_2021_26810_MOESM8_ESM.pdf]

#Supplemental_File_S5: CONTROL-SPECIFIC SOX2 PEAKS

CHR	START	END	CLOSEST_GENE	DISTANCE_FROM_TSS
chr1	1711630	1711864	NADK	122
chr1	3022116	3022356	MIR4251	22182
chr1	5285722	5286222	MIR4417	337908
chr1	5327565	5328009	MIR4417	296121
chr1	6555655	6555888	PLEKHG5	1268
chr1	8923805	8924056	ENO1	7579
chr1	9111533	9111799	SLC2A5	18088
chr1	11969638	11969898	KIAA2013	16587
chr1	14919884	14920214	KAZN	4998
chr1	16840337	16840789	CROCCP3	21141
chr1	18745560	18745854	KLHDC7A	61569
chr1	18929834	18930107	PAX7	27392
chr1	20775180	20775567	LOC339505	19893
chr1	21262242	21262506	HP1BP3	149061
chr1	22552322	22552719	WNT4	82803
chr1	23924944	23925263	MDS2	28560
chr1	25051906	25052184	CLIC4	19575
chr1	28975060	28975595	RNU11	0
chr1	30447229	30447533	MATN1-AS1	744085
chr1	32800024	32800265	MARCKSL1	1575
chr1	34337993	34338263	LOC402779	3437
chr1	37138823	37139106	CSF3R	189908
chr1	37231454	37231696	GRIK3	268148
chr1	37236768	37237112	GRIK3	262732
chr1	38888660	38889031	LOC339442	208221
chr1	41177995	41178225	LOC100130557	20062
chr1	44585325	44585559	KLF17	804
chr1	49038372	49038632	SPATA6	100496
chr1	55996434	55996746	MIR4422	305121
chr1	56919085	56919315	PPAP2B	125942
chr1	57670632	57670964	C8B	238944
chr1	63765082	63765476	LINC00466	17425
chr1	65352656	65352886	JAK1	79301
chr1	67924515	67924928	SERBP1	28392
chr1	71053465	71053817	CTH	176565
chr1	71928007	71928295	NEGR1-IT1	374400
chr1	73013163	73013416	NEGR1	264886
chr1	75548998	75549246	LHX8	44872
chr1	76968855	76969128	ST6GALNAC5	364057
chr1	80647068	80647562	ELTD1	1174573
chr1	85037642	85038009	CTBS	2154
chr1	91003572	91003912	BARHL2	178882
chr1	91392545	91392861	ZNF644	94204
chr1	95391244	95391474	CNN3	1261
chr1	96940927	96941239	PTBP2	245935
chr1	99534735	99535087	LPPR5	64286

chr1	100770496	100770738	MIR553	23700
chr1	100927965	100928250	GPR88	75477
chr1	108305562	108305890	VAV3	74436
chr1	108410054	108410420	VAV3-AS1	96644
chr1	113570224	113570529	LRIG2	45301
chr1	116575875	116576153	SLC22A15	56757
chr1	119883511	119883805	HAO2	27596
chr1	147349725	147349981	GJA8	24964
chr1	149858181	149858634	HIST2H2BE	0
chr1	150228794	150229052	CA14	1165
chr1	150400922	150401173	TARS2	58746
chr1	157039506	157039805	ARHGEF11	24344
chr1	161720124	161720422	DUSP12	544
chr1	163437080	163437434	NUF2	145358
chr1	165911432	165911722	MIR3658	34275
chr1	166035428	166035708	MIR921	88327
chr1	167175465	167175739	POU2F1	14326
chr1	172247682	172248042	DNM3OS	133707
chr1	172507089	172507353	SUCO	4773
chr1	181034834	181035108	IER5	22529
chr1	193009114	193009513	UCHL5	19010
chr1	201605349	201605653	NAV1	11796
chr1	202070924	202071165	GPR37L1	20863
chr1	203626508	203626823	ATP2B4	30594
chr1	204116618	204116890	ETNK2	4417
chr1	204389537	204389767	PPP1R15B	8593
chr1	205414018	205414284	MIR135B	3242
chr1	205639732	205640122	SLC45A3	9508
chr1	208375690	208376015	PLXNA2	41650
chr1	212483414	212483665	PPP2R5A	8267
chr1	214489954	214490254	SMYD2	35390
chr1	214490651	214491009	SMYD2	36087
chr1	216913721	216913998	ESRRG	16907
chr1	217517326	217517645	ESRRG	206229
chr1	218197703	218197962	LINC00210	131462
chr1	218207822	218208161	LINC00210	141581
chr1	218667797	218668291	LOC728463	148777
chr1	220006912	220007187	RNU5F-1	39431
chr1	223493302	223493541	SUSD4	44003
chr1	225696219	225696509	LBR	79662
chr1	226592635	226592893	PARP1	2908
chr1	232608526	232608789	SIPA1L2	42454
chr1	232981364	232981706	KIAA1383	40727
chr1	235049519	235049800	LOC100506810	189731
chr1	238540650	238540927	LOC339535	108390
chr1	241211868	241212252	MIR3123	83319
chr1	242099655	242099943	MAP1LC3C	62442
chr1	242992189	242992514	LOC731275	272532
chr10	2756863	2757093	PFKP	352618
chr10	5511098	5511407	NET1	22553

chr10	7677414	7677737	ITIH5	15778
chr10	7932185	7932528	TAF3	71513
chr10	9552717	9552947	SFTA1P	1283930
chr10	11378871	11379110	CELF2	171879
chr10	12753238	12753493	LOC283070	121639
chr10	16237846	16238185	PTER	240756
chr10	16971103	16971427	RSU1	111650
chr10	17261164	17261394	VIM	8863
chr10	18383762	18384105	CACNB2	45500
chr10	20253618	20253973	PLXDC2	148247
chr10	20480379	20480612	MIR4675	360286
chr10	21677603	21677833	MIR1915	107737
chr10	32555905	32556199	EPC1	79914
chr10	33986984	33987311	LOC100505583	74297
chr10	35292937	35293202	CUL2	70056
chr10	36238664	36238896	FZD8	308302
chr10	36977734	36978008	ANKRD30A	436776
chr10	43850493	43850741	FXYD4	16350
chr10	43868559	43868871	FXYD4	1468
chr10	52617322	52617556	A1CF	27879
chr10	54727876	54728208	MBL2	196416
chr10	57279126	57279414	MTRNR2L5	79335
chr10	62239113	62239385	ANK3	89479
chr10	68685616	68685865	LRRTM3	0
chr10	71712561	71712918	H2AFY2	99438
chr10	79637125	79637462	DLG5	48886
chr10	80311107	80311338	LOC100132987	302726
chr10	87651292	87651566	GRID1-AS1	313805
chr10	89733468	89733745	PTEN	110274
chr10	90846539	90846776	MIR4679-2	23371
chr10	92154167	92154404	HTR7	463267
chr10	93504926	93505156	TNKS2	52994
chr10	96383302	96383547	CYP2C18	59703
chr10	96955783	96956051	C10orf129	1827
chr10	98210680	98210938	TLL2	62745
chr10	98604480	98604710	LCOR	11769
chr10	99239591	99239973	MMS19	18393
chr10	104409285	104409515	TRIM8	5034
chr10	105826900	105827147	COL17A1	18491
chr10	108468334	108468605	SORCS1	455861
chr10	111153247	111153484	RNU6-53	222833
chr10	111694773	111695129	XPNPEP1	11462
chr10	114571787	114572100	TCF7L2	137908
chr10	114813123	114813356	TCF7L2	103115
chr10	118561918	118562217	ENO4	46805
chr10	122264827	122265057	PPAPDC1A	48362
chr10	124108673	124108963	PLEKHA1	25130
chr10	125557379	125557635	CPXM2	93865
chr10	126843829	126844079	CTBP2	5024
chr10	130860212	130860458	MGMT	404995

chr10	134560538	134560803	NKX6-2	38734
chr11	2905393	2905739	CDKN1C	1256
chr11	3085167	3085421	CARS	6486
chr11	5278588	5278925	HBG2	2577
chr11	6303189	6303445	CCKBR	22286
chr11	6864923	6865220	OR10A5	1693
chr11	9482111	9482343	LOC644656	0
chr11	9648458	9648703	SWAP70	36924
chr11	11713431	11713661	MIR4299	35162
chr11	13136479	13136731	RASSF10	105510
chr11	15866757	15867172	SOX6	557241
chr11	16805673	16805966	C11orf58	45526
chr11	17668002	17668237	MYOD1	72872
chr11	18428731	18429115	LDHC	4737
chr11	19830440	19830709	MIR4694	48811
chr11	20230501	20230930	DBX1	48631
chr11	24701527	24702027	LUZP2	183012
chr11	31443344	31443749	DNAJC24	51968
chr11	31892659	31893007	PAX6	53150
chr11	33209658	33210019	LOC338739	26456
chr11	35565129	35565377	PAMR1	17953
chr11	41742428	41742763	LRRC4C	261242
chr11	41943493	41943806	LOC100507205	331434
chr11	43312340	43312712	API5	20792
chr11	44692723	44693023	TSPAN18	92952
chr11	44884889	44885154	TP53I11	86605
chr11	46223846	46224091	CREB3L1	75136
chr11	47996097	47996375	PTPRJ	5734
chr11	57568123	57568420	CTNND1	38890
chr11	61648923	61649158	FADS3	9848
chr11	65264874	65265243	MALAT1	0
chr11	71751790	71752095	MIR3165	31253
chr11	75374857	75375127	MAP6	4352
chr11	76504432	76504806	TSKU	10148
chr11	79088548	79088978	MIR708	24175
chr11	79184156	79184470	ODZ4	32461
chr11	81358399	81358728	MIR4300	243150
chr11	82525466	82525820	FAM181B	80560
chr11	83733406	83733670	DLG2	250653
chr11	85060103	85060333	DLG2	277981
chr11	86040551	86040874	C11orf73	26154
chr11	86299537	86299767	ME3	83473
chr11	100534347	100534665	ARHGAP42	23741
chr11	100661902	100662210	ARHGAP42	103496
chr11	100698846	100699156	ARHGAP42	140440
chr11	101394662	101394933	MIR3920	4026
chr11	112467019	112467295	C11orf34	335436
chr11	113863450	113863798	HTR3A	15229
chr11	114644279	114644639	NXPE2	95080
chr11	115622097	115622377	LOC283143	8541

chr11	115773887	115774172	LOC283143	142969
chr11	121713042	121713272	MIR125B1	257280
chr11	123278449	123278722	MIR4493	26229
chr11	123645993	123646370	OR6X1	20767
chr11	130790603	130790885	SNX19	4221
chr12	3771615	3771851	EFCAB4B	90515
chr12	6432417	6432663	PLEKHG6	10601
chr12	6505518	6505759	LTBR	12162
chr12	6646632	6647121	GAPDH	2224
chr12	6647231	6647496	GAPDH	2823
chr12	7243295	7243635	C1R	1408
chr12	8298608	8298864	POU5F1P3	11160
chr12	12813898	12814312	GPR19	34809
chr12	13880828	13881072	GRIN2B	251950
chr12	14412984	14413392	ATF7IP	105218
chr12	18834733	18835062	PLCZ1	55856
chr12	19157066	19157296	PLEKHA5	125329
chr12	19178774	19179075	PLEKHA5	103550
chr12	23738709	23739025	SOX5	1163
chr12	24368907	24369148	MIR920	3553
chr12	24409810	24410059	MIR920	44456
chr12	25216775	25217055	LRMP	11595
chr12	26040240	26040614	MIR4302	13228
chr12	27311273	27311647	C12orf71	75818
chr12	27429205	27429565	STK38L	32128
chr12	28613475	28613734	CCDC91	203343
chr12	30037888	30038190	TMTC1	100196
chr12	31523830	31524060	FAM60A	44671
chr12	32292909	32293156	BICD1	32725
chr12	33740794	33741302	SYT10	148040
chr12	39139462	39139719	CPNE8	159701
chr12	46713744	46714094	SLC38A1	50536
chr12	47063591	47063933	SLC38A4	155847
chr12	51714273	51714680	BIN2	3258
chr12	52890453	52890746	KRT6A	3272
chr12	53892367	53892621	MAP3K12	823
chr12	54695214	54695538	NFE2	393
chr12	56140721	56140975	GDF11	3658
chr12	61228622	61228914	SLC16A7	1145497
chr12	61915061	61915411	FAM19A2	671209
chr12	62997506	62997791	MIRLET7I	41
chr12	63046640	63046985	MIRLET7I	49175
chr12	63455868	63456098	AVPR1A	90492
chr12	69979002	69979235	CCT2	0
chr12	70290503	70290810	RAB3IP	117750
chr12	70327031	70327301	RAB3IP	154278
chr12	72341594	72341946	TPH2	8969
chr12	74024223	74024650	LOC100507377	661761
chr12	76352952	76353213	PHLDA1	72343
chr12	77266146	77266380	CSRP2	6419

chr12	77668784	77669139	E2F7	209424
chr12	79775084	79775326	MIR1252	37710
chr12	79941200	79941511	MIR5692B	90550
chr12	80714157	80714387	OTOGL	110925
chr12	81078026	81078327	MYF6	23080
chr12	81602331	81602619	MIR4699	50165
chr12	85199261	85199562	SLC6A15	107046
chr12	86343195	86343442	NTS	75123
chr12	89481645	89481931	LOC728084	68176
chr12	93977804	93978034	SOCS2	11503
chr12	94093115	94093477	CRADD	21965
chr12	96343507	96343766	AMDHD1	6437
chr12	98178796	98179135	MIR4303	210091
chr12	99420493	99420809	ANKS1B	127768
chr12	106975350	106975649	RFX4	1035
chr12	107162076	107162335	RIC8B	6063
chr12	107259450	107259723	C12orf23	89820
chr12	111372355	111372585	LOC100131138	1820
chr12	114646013	114646289	TBX5	197679
chr12	119776790	119777082	CCDC60	4274
chr12	123376338	123376568	VPS37B	4144
chr12	123886591	123886884	SETD8	17888
chr12	127095344	127095591	LOC100507206	125961
chr12	128782775	128783029	MIR3612	4139
chr12	128867893	128868417	MIR3612	89257
chr12	128943694	128943994	MIR3612	165058
chr12	130356613	130357263	TMEM132D	30949
chr12	130529717	130529957	LOC100190940	2830
chr12	130646805	130647083	FZD10	0
chr12	131597877	131598240	LOC116437	51315
chr12	131825470	131825765	LOC116437	175915
chr13	26052463	26052813	ATP8A2	106255
chr13	26545253	26545596	SHISA2	79602
chr13	27049653	27049901	WASF3	81938
chr13	27471407	27471710	GPR12	136485
chr13	28396830	28397117	GSX1	30051
chr13	29029820	29030157	FLT1	39108
chr13	30538261	30538593	LOC440131	27594
chr13	31039953	31040303	HMGB1	0
chr13	36147862	36148092	NBEA	96977
chr13	38221486	38221716	POSTN	48505
chr13	39101813	39102070	LINC00366	39790
chr13	39599605	39599914	PROSER1	12338
chr13	40064251	40064587	LHFP	112769
chr13	43167704	43167993	TNFSF11	19414
chr13	47521600	47521982	HTR2A	50389
chr13	49595302	49595537	FNDC3A	45255
chr13	53850349	53850790	OLFM4	247474
chr13	60053887	60054224	DIAPH3-AS1	532660
chr13	67525604	67525912	PCDH9-AS3	25608

chr13	68310970	68311314	PCDH9	506502
chr13	79862454	79862824	RBM26	117099
chr13	80871488	80871790	SPRY2	43296
chr13	83321580	83321879	SLITRK1	1134649
chr13	83473935	83474165	SLITRK1	982363
chr13	86142076	86142421	LINC00351	204339
chr13	94870824	94871169	DCT	260767
chr13	95477879	95478126	SOX21	113490
chr13	96606512	96606905	UGGT2	98831
chr13	107638438	107638777	LINC00443	332211
chr13	110919326	110919591	COL4A1	39905
chr13	111118747	111118992	COL4A2-AS1	41534
chr13	112758198	112758579	SOX1	36286
chr13	114031231	114031478	GRTP1	12768
chr13	114805447	114805747	RASA3	92348
chr14	21046458	21046809	RNASE11	11608
chr14	34400640	34400990	EGLN3	19294
chr14	39261088	39261440	LOC283547	124646
chr14	39656735	39657065	PNN	12349
chr14	44224074	44224367	FSCB	752132
chr14	46874055	46874499	RPL10L	246529
chr14	48444832	48445119	LOC100506433	180615
chr14	49343866	49344208	RPS29	708886
chr14	50605932	50606168	METTL21D	22635
chr14	52718562	52718792	PTGDR	15638
chr14	55508759	55509018	MAPK1IP1L	9343
chr14	56358465	56358814	LINC00520	95073
chr14	56991653	56991893	C14orf101	54617
chr14	58181228	58181562	SLC35F4	151030
chr14	61990854	61991389	FLJ22447	45868
chr14	65833901	65834203	MIR4708	32000
chr14	68839174	68839414	ZFP36L1	421217
chr14	70706361	70706668	ADAM21P1	7850
chr14	72710009	72710359	RGS6	310224
chr14	73088051	73088380	DPF3	272429
chr14	73271118	73271348	DPF3	89461
chr14	74896005	74896321	SYNDIG1L	3200
chr14	75946582	75946883	BATF	41900
chr14	77434907	77435354	IRF2BPL	59688
chr14	78834241	78834471	NRXN3	35621
chr14	81747772	81748003	GTF2A1	60197
chr14	84427287	84427775	FLRT2	1568712
chr14	88263382	88263664	GALC	195951
chr14	89759978	89760218	FOXN3	123236
chr14	91071875	91072111	LOC400238	150302
chr14	91580598	91580847	C14orf159	131
chr14	92036394	92036855	SMEK1	59750
chr14	96445922	96446291	C14orf132	59369
chr14	100137499	100137741	MIR5698	2477
chr14	102411488	102411860	DYNC1H1	19004

chr14	104606191	104606521	KIF26A	1132
chr15	25068714	25069117	SNRPN	0
chr15	27657687	27657987	GABRG3	441259
chr15	28012914	28013278	OCA2	331180
chr15	37428378	37428651	MEIS2	34878
chr15	38364940	38365302	TMCO5A	137484
chr15	39505701	39505994	C15orf54	36890
chr15	39637393	39637630	C15orf54	94509
chr15	39984735	39985175	FSIP1	89864
chr15	42676904	42677164	CAPN3	17423
chr15	43182782	43183035	TTBK2	29972
chr15	48591154	48591492	DUT	32128
chr15	49621243	49621483	FGF7	93891
chr15	49902775	49903163	FAM227B	9955
chr15	50188036	50188333	ATP8B4	223086
chr15	58043646	58043908	POLR2M	44746
chr15	59221988	59222461	SLTM	3391
chr15	59453479	59453739	MIR2116	9722
chr15	59837832	59838101	GCNT3	65880
chr15	60935835	60936075	RORA	16106
chr15	62587452	62587682	C2CD4B	129970
chr15	63688695	63688925	CA12	14620
chr15	65074475	65074759	RBPMS2	6705
chr15	70925400	70925819	UACA	68801
chr15	71927161	71927428	NR2E3	175465
chr15	72535019	72535288	PKM	11292
chr15	74592387	74592762	CCDC33	18137
chr15	75016342	75016718	CYP1A1	1159
chr15	77422947	77423200	TSPAN3	59377
chr15	80766108	80766338	ARNT2	69417
chr15	89527136	89527496	MFGE8	70473
chr15	89878150	89878531	POLG	124
chr15	91127202	91127443	CRTC3	54085
chr15	92043015	92043305	SLCO3A1	353632
chr15	93447302	93447568	MIR3175	60
chr15	94773310	94773660	MCTP2	67769
chr15	101175962	101176304	ASB7	33208
chr16	533682	533912	RAB11FIP3	8833
chr16	2368758	2369011	ABCA3	21736
chr16	4764200	4764521	ANKS3	19642
chr16	6174321	6174581	RBFOX1	105190
chr16	6391957	6392218	RBFOX1	322826
chr16	7355270	7355517	RBFOX1	27233
chr16	8916466	8916841	PMM2	24797
chr16	13688689	13689002	ERCC4	325011
chr16	19959622	19959855	GPRC5B	63471
chr16	22308402	22308632	POLR3E	63
chr16	24150728	24151008	CACNG3	115865
chr16	25586807	25587077	HS3ST4	116269
chr16	25961937	25962524	HS3ST4	258591

chr16	26067161	26067489	HS3ST4	363815
chr16	30381910	30382271	TBC1D10B	388
chr16	30934026	30934261	FBXL19-AS1	329
chr16	31084487	31084749	ZNF668	74
chr16	47400309	47400555	ITFG1	94460
chr16	49366872	49367294	C16orf78	40513
chr16	50644510	50644771	NKD1	62270
chr16	51393054	51393344	SALL1	207871
chr16	51426217	51426475	SALL1	241034
chr16	51648990	51649317	LOC388276	458530
chr16	51704375	51704723	LOC388276	403124
chr16	53949439	53949737	FTO	211565
chr16	54464480	54464782	IRX3	144102
chr16	54780422	54780787	CRNDE	181903
chr16	57333817	57334062	PLLP	15233
chr16	59203990	59204308	GOT2	435744
chr16	61870807	61871062	CDH8	199677
chr16	67627139	67627407	CTCF	30830
chr16	67840425	67840812	RANBP10	0
chr16	78940140	78940428	MAF	694194
chr16	79631889	79632139	MAF	2483
chr16	80062891	80063200	MAF	428269
chr16	80700628	80700858	DYNLRB2	125775
chr16	83636395	83636743	MIR3182	94445
chr16	89357245	89357660	LOC100287036	29880
chr17	880512	880742	NXN	2256
chr17	2777367	2777713	RAP1GAP2	77636
chr17	5800583	5800916	LOC339166	125030
chr17	10985939	10986183	SHISA6	158556
chr17	26321663	26321904	NLK	47783
chr17	29884287	29884565	MIR193A	2449
chr17	35901158	35901444	DUSP14	51208
chr17	36765693	36766011	SRCIN1	3510
chr17	40164480	40164722	DNAJC7	4461
chr17	41179563	41179801	RND2	2306
chr17	41188226	41188591	RND2	10969
chr17	41561161	41561458	DHX8	0
chr17	42589019	42589372	GPATCH8	8062
chr17	45142878	45143195	RPRML	86264
chr17	46604375	46604686	HOXB1	3586
chr17	49124421	49124736	SPAG9	92
chr17	49998889	49999168	CA10	236964
chr17	52333961	52334215	KIF2B	433723
chr17	66420426	66420700	MIR635	0
chr17	67444668	67444964	MAP2K6	33831
chr17	70103179	70103421	SOX9	13739
chr17	71563108	71563339	SDK2	76888
chr17	73775862	73776123	H3F3B	2
chr17	74102239	74102573	EXOC7	2371
chr17	75806515	75806840	FLJ45079	73329

chr17	78500326	78500597	RPTOR	18027
chr17	78801519	78801814	CHMP6	163826
chr17	78833349	78833825	CHMP6	131815
chr17	79477093	79477436	ACTG1	2456
chr17_gl000205_random	87029	87655	-1	-1
chr18	955087	955423	ADCYAP1	49791
chr18	1090803	1091215	ADCYAP1	185507
chr18	2059406	2059636	METTL4	511853
chr18	3665864	3666109	FLJ35776	71753
chr18	5941951	5942331	TMEM200C	49848
chr18	9106885	9107257	NDUFV2	4258
chr18	9434405	9434668	RALBP1	40861
chr18	19643805	19644035	GATA6	105368
chr18	21958625	21958905	OSBPL1A	18928
chr18	22529890	22530140	LOC729950	321745
chr18	22864980	22865380	ZNF521	66834
chr18	23323524	23323900	SS18	346711
chr18	28367435	28367702	DSC3	255079
chr18	34061146	34061412	FHOD3	183445
chr18	35134304	35134595	CELF4	11405
chr18	36132970	36133215	MIR4318	895873
chr18	37171677	37171948	MIR5583-1	84736
chr18	38891683	38891953	KC6	208608
chr18	45833293	45833585	ZBTB7C	169613
chr18	56179399	56179676	MIR3591	61015
chr18	59304353	59304647	CDH20	146579
chr18	68972077	68972365	LOC100505776	273827
chr18	69774912	69775193	CBLN2	436530
chr18	70111841	70112187	CBLN2	99536
chr18	76741643	76742007	SALL3	1369
chr19	1249373	1249951	MIDN	822
chr19	2028758	2028991	BTBD2	13056
chr19	2042649	2042948	MKNK2	8295
chr19	3977963	3978267	SNORD37	4303
chr19	12075722	12075979	ZNF763	0
chr19	13130906	13131178	NFIX	24323
chr19	18455462	18455754	PGPEP1	4055
chr19	21888138	21888545	LOC641367	45001
chr19	30788492	30788776	ZNF536	74551
chr19	30864543	30864830	ZNF536	1216
chr19	45393508	45393850	TOMM40	626
chr19	48353001	48353252	CRX	27903
chr19	54286006	54286247	MIR371A	4681
chr19	55860407	55860752	COX6B2	5430
chr2	2791877	2792174	MYT1L	456832
chr2	5135733	5135968	LOC727982	431921
chr2	5625570	5625932	SOX11	206866
chr2	6780532	6780762	LINC00487	129680
chr2	6819598	6819890	LINC00487	90552
chr2	8357834	8358064	LINC00299	110485

chr2	12223151	12223395	MIR4262	246039
chr2	15399438	15399776	NBAS	301696
chr2	16120620	16120968	MYCNOS	38775
chr2	16386112	16386420	MYCNOS	304267
chr2	17319643	17319881	RAD51AP2	379825
chr2	17738861	17739171	VSNL1	17055
chr2	17910436	17910769	SMC6	24327
chr2	19171844	19172101	MIR4757	376088
chr2	21417852	21418099	APOB	150907
chr2	23101064	23101336	KLHL29	506961
chr2	24800637	24800900	NCOA1	6445
chr2	29513501	29513735	CLIP4	175194
chr2	33612745	33613057	RASGRP3	48358
chr2	34591071	34591334	MYADML	637787
chr2	34672328	34672723	MYADML	719044
chr2	38452005	38452332	CYP1B1-AS1	93759
chr2	44288956	44289226	LRPPRC	65812
chr2	44365124	44365415	PPM1B	30584
chr2	44466345	44466677	SLC3A1	35919
chr2	47572058	47572516	EPCAM	23770
chr2	47906055	47906358	MSH6	103862
chr2	50424287	50424584	NRXN1	150310
chr2	55646894	55647146	CCDC88A	0
chr2	62693061	62693291	TMEM17	40313
chr2	63805994	63806288	MDH1	9454
chr2	65502458	65502721	ACTR2	47630
chr2	69945078	69945515	ANXA4	23611
chr2	70475616	70475957	TIA1	0
chr2	70847188	70847506	TGFA	66041
chr2	71688194	71688529	DYSF	5302
chr2	79601265	79601556	CTNNA2	138503
chr2	86668083	86668350	KDM3A	0
chr2	100735722	100736117	AFF3	13677
chr2	101220422	101220779	PDCL3	41005
chr2	102225836	102226174	MAP4K4	87990
chr2	105783192	105783496	GPR45	74703
chr2	106578155	106578395	C2orf40	103717
chr2	115467603	115468016	DPP10	248424
chr2	119954016	119954368	STEAP3	27015
chr2	121345364	121345663	LOC84931	121439
chr2	123418959	123419210	TSN	905839
chr2	134891389	134891644	MIR3679	6694
chr2	139481953	139482303	NXPH2	55508
chr2	147575313	147575543	PABPC1P2	230689
chr2	149280885	149281126	EPC2	121433
chr2	150920426	150920811	RND3	423398
chr2	151968405	151968656	RBM43	149733
chr2	158695624	158695857	ACVR1	35766
chr2	164415263	164415521	FIGN	176992
chr2	165191695	165191984	GRB14	286376

chr2	165640994	165641228	COBLL1	56700
chr2	168701142	168701384	B3GALT1	25961
chr2	171536678	171537073	LOC440925	34004
chr2	174662560	174662850	SP3	166097
chr2	177190423	177190823	MTX2	56301
chr2	180045136	180045381	SESTD1	83969
chr2	180414823	180415279	ZNF385B	12036
chr2	205069889	205070229	ICOS	268419
chr2	206515073	206515409	NRP2	31814
chr2	206795002	206795339	INO80D	155567
chr2	207193710	207193987	ZDBF2	54188
chr2	208702999	208703267	FZD5	68856
chr2	210621940	210622396	UNC80	14320
chr2	211724061	211724364	CPS1-IT1	241767
chr2	218486899	218487144	DIRC3	134172
chr2	218700892	218701219	DIRC3	79576
chr2	220918572	220918930	MIR4268	147286
chr2	221813306	221813672	EPHA4	623338
chr2	222434737	222435029	EPHA4	1981
chr2	222776108	222776379	EPHA4	339098
chr2	222776641	222776940	EPHA4	339631
chr2	222890569	222890922	CCDC140	271943
chr2	224817009	224817353	MRPL44	4767
chr2	225773598	225773888	MIR4439	101369
chr2	226532300	226532541	MIR548AR	95619
chr2	232044522	232044908	ARMC9	18433
chr2	235869488	235869732	SH3BP4	8861
chr20	3430001	3430232	ATRN	21432
chr20	15201985	15202220	MACROD2	24482
chr20	16008378	16008620	KIF16B	545459
chr20	16217099	16217350	KIF16B	336729
chr20	20444572	20444865	INSM1	95808
chr20	22547944	22548186	LINC00261	11094
chr20	23454004	23454239	CST8	17526
chr20	24165647	24165877	FLJ33581	14525
chr20	30192005	30192235	ID1	850
chr20	30300505	30300745	BCL2L1	9911
chr20	30493724	30494053	TTLL9	35220
chr20	36981651	36982057	LBP	6838
chr20	40767315	40767624	CHD6	520182
chr20	40903698	40903992	CHD6	656565
chr20	47139660	47139928	LINC00494	151007
chr20	48384755	48385000	SLC9A8	44249
chr20	49922342	49922631	MIR3194	146883
chr20	50620523	50620813	ZFP64	187711
chr20	52484102	52484415	SUMO1P1	7833
chr20	53782758	53783073	DOK5	690493
chr20	54039717	54040020	CBLN4	539992
chr20	56098043	56098333	CTCFL	1590
chr20	61546333	61546638	DIDO1	11265

chr20	61809873	61810113	MIR124-3	22	
chr21	16288933	16289174	NRIP1	147952	
chr21	17125365	17125672	USP25	22870	
chr21	18827038	18827280	C21orf37	15831	
chr21	28419892	28420185	ADAMTS5	80453	
chr21	29963164	29963423	LINC00161		51525
chr21	33102898	33103348	SCAF4	1083	
chr21	33933611	33933987	TCP10L	23858	
chr21	34126576	34126829	GCFC1	17340	
chr21	34340465	34340743	OLIG2	57472	
chr21	39041208	39041513	KCNJ6	247228	
chr21	40351243	40351501	ETS2	173489	
chr22	17916273	17916562	CECR2	40065	
chr22	19879131	19879426	GNB1L	36669	
chr22	28583643	28583962	MIR3199-1		267043
chr22	35531203	35531509	ISX	69074	
chr22	35772905	35773207	HMOX1	3852	
chr22	39447944	39448233	APOBEC3F	11272	
chr22	40405420	40405670	FAM83F	14468	
chr22	42461435	42461692	NAGA	5154	
chr22	42761330	42761560	NFAM1	66841	
chr3	1172969	1173231	CNTN6	38341	
chr3	2346357	2346674	CNTN4	65845	
chr3	3840869	3841120	LRRN1	0	
chr3	8152950	8153196	LOC100288428		390148
chr3	8536032	8536405	LOC100288428		6939
chr3	10436944	10437190	MIR885	698	
chr3	10595375	10595727	ATP2B2	48107	
chr3	12826654	12826911	CAND2	11259	
chr3	20292331	20292621	SGOL1	64607	
chr3	22747017	22747295	UBE2E2	497488	
chr3	24518708	24519067	LOC644990		16510
chr3	24560806	24561144	MIR4792	1782	
chr3	30792368	30792645	GADL1	143508	
chr3	32362505	32362762	CMTM7	70400	
chr3	32885514	32885864	TRIM71	26005	
chr3	33830640	33831033	PDCD6IP	9029	
chr3	34295853	34296132	PDCD6IP	455791	
chr3	45353490	45353838	LARS2	76236	
chr3	46615869	46616172	TDGF1	0	
chr3	48318425	48318721	NME6	24127	
chr3	50855552	50855846	DOCK3	142881	
chr3	57193316	57193605	IL17RD	5798	
chr3	58567177	58567412	FAM107A	3686	
chr3	59079357	59079655	C3orf67	43642	
chr3	60557288	60557527	FHIT	679606	
chr3	71353502	71353849	FOXP1	62	
chr3	72816068	72816309	SHQ1	81289	
chr3	75303896	75304150	MIR4444-1		40270
chr3	79117877	79118166	ROBO1	49268	

chr3	83617221	83617486	LOC440970	1301240
chr3	86940194	86940565	VGLL3	99692
chr3	87776976	87777350	HTR1F	254375
chr3	100280671	100280929	GPR128	47503
chr3	101705573	101705817	LOC152225	45871
chr3	102406898	102407317	ZPLD1	253040
chr3	103374898	103375128	MIR548A3	570977
chr3	104875879	104876458	ALCAM	209098
chr3	106028179	106028489	CBLB	440292
chr3	108298767	108299073	DZIP3	9263
chr3	110302172	110302462	PVRL3-AS1	486344
chr3	112110017	112110266	CD200	58102
chr3	113829875	113830105	QTRTD1	54294
chr3	114261339	114261588	ZBTB20	81465
chr3	115578218	115578593	GAP43	236068
chr3	117126302	117126532	LSAMP-AS3	697668
chr3	119068372	119068670	ARHGAP31	55153
chr3	122187974	122188204	KPNA1	45582
chr3	122458109	122458368	HSPBAP1	54298
chr3	130682842	130683101	ASTE1	62545
chr3	131515579	131515839	MIR5704	188936
chr3	134413486	134413716	KY	43622
chr3	136913237	136913480	IL20RB	236531
chr3	140017414	140017652	CLSTN2	363388
chr3	140432789	140433048	TRIM42	35924
chr3	144001221	144001531	C3orf58	309055
chr3	144976936	144977249	PLOD2	902033
chr3	145437060	145437332	PLOD2	441950
chr3	147119247	147119647	ZIC4	2424
chr3	155463721	155464000	PLCH1	41724
chr3	157155538	157155844	PTX3	959
chr3	158867399	158867730	IQCJ	80359
chr3	171101931	171102205	TNIK	75992
chr3	173517942	173518246	NLGN1	401699
chr3	179419273	179419591	USP13	48341
chr3	179598761	179599012	PEX5L	92996
chr3	179600120	179600363	PEX5L	91645
chr3	179667299	179667657	PEX5L	24351
chr3	181473857	181474118	SOX2	44146
chr3	181658516	181658815	SOX2	228805
chr3	182218203	182218513	FLJ46066	14053
chr3	183152876	183153296	MCF2L2	7021
chr3	185508016	185508343	IGF2BP2	34484
chr3	187079632	187080010	RTP4	6157
chr3	193493373	193493747	OPA1-AS1	148252
chr3	193693036	193693296	LOC100128023	18731
chr3	193772418	193772788	LOC647323	50970
chr3	194103975	194104294	LRRC15	13503
chr3	194916208	194916445	XXYLT1	75450
chr4	4577090	4577346	LOC100507266	33233

chr4	4644177	4644424	LOC100507266	100320
chr4	15257239	15257514	C1QTNF7	84045
chr4	16010611	16010857	FGFBP2	45752
chr4	25487824	25488107	ANAPC4	108977
chr4	29377309	29377598	MIR4275	556106
chr4	30602570	30602924	PCDH7	119105
chr4	40720689	40721004	NSUN7	30909
chr4	41329770	41330030	LIMCH1	32773
chr4	41622872	41623121	LIMCH1	7954
chr4	44058921	44059185	KCTD8	391639
chr4	55144430	55144718	PDGFRA	49167
chr4	55998982	55999213	KDR	7220
chr4	57291682	57291971	PPAT	9831
chr4	62075980	62076218	LPHN3	286620
chr4	62589364	62589594	LPHN3	226526
chr4	62636243	62636489	LPHN3	273405
chr4	67159595	67159952	LOC100144602	623917
chr4	74850878	74851171	PPBP	2736
chr4	80970569	80970832	ANTXR2	23645
chr4	87857078	87857363	AFF1	925
chr4	88856706	88856967	SPP1	39834
chr4	91649088	91649387	FAM190A	492907
chr4	96759490	96759741	PDHA2	1497
chr4	101022185	101022461	DDIT4L	89194
chr4	105422515	105422769	CXXC4	6457
chr4	108812017	108812361	SGMS2	2058
chr4	109115537	109115779	LEF1-AS1	22262
chr4	111564242	111564490	PITX2	963
chr4	113568006	113568291	MIR367	806
chr4	113668265	113668590	ANK2	70648
chr4	114805869	114806133	ARSJ	94745
chr4	115648613	115648906	MIR577	70699
chr4	121387569	121387881	MAD2L1	399556
chr4	128424323	128424553	INTU	129533
chr4	131106281	131106662	C4orf33	1089000
chr4	134514940	134515228	PCDH10	444471
chr4	135857189	135857451	PABPC4L	734286
chr4	136107304	136107535	PABPC4L	984401
chr4	147932620	147932903	TTC29	65586
chr4	149803442	149803721	NR3C2	439770
chr4	150061176	150061449	NR3C2	697504
chr4	151467496	151467771	MAB21L2	35305
chr4	154794963	154795226	SFRP2	84735
chr4	159883600	159883886	C4orf45	72447
chr4	162110963	162111232	FSTL5	973954
chr4	165787975	165788276	LOC100506013	9879
chr4	169093270	169093513	ANXA10	79583
chr4	169706148	169706490	PALLD	46665
chr4	169762249	169762524	PALLD	9094
chr4	171943987	171944286	LOC100506122	17466

chr4	174013145	174013408	GALNT7	76495
chr4	178480096	178480468	AGA	116439
chr4	179674209	179674478	LOC285501	1024299
chr4	182087982	182088322	LINC00290	7680
chr4	184799764	184800164	STOX2	26344
chr4	187645361	187645625	FAT1	374
chr4	189029402	189029669	TRIML2	2994
chr4	190587025	190587260	HSP90AA4P	192727
chr5	4213055	4213676	IRX1	616888
chr5	4549358	4549798	LOC340094	484673
chr5	5015063	5015328	LOC340094	19143
chr5	5695226	5695459	KIAA0947	272441
chr5	6302414	6302644	FLJ33360	34761
chr5	9601225	9601499	TAS2R1	28964
chr5	12174944	12175221	CTNND2	270834
chr5	15553863	15554093	FBXL7	53559
chr5	15919182	15919418	11-Mar	260479
chr5	16061423	16061820	11-Mar	118077
chr5	17147410	17147640	LOC285696	69891
chr5	18394430	18394664	LOC401177	1007011
chr5	32952485	32952797	LOC340113	4937
chr5	41708274	41708584	OXCT1	162207
chr5	42507811	42508118	GHR	40026
chr5	43145235	43145467	ZNF131	23594
chr5	52070044	52070274	PELO	13499
chr5	52714233	52714552	FST	61711
chr5	54326015	54326338	GZMK	5909
chr5	54921464	54921694	SLC38A9	67187
chr5	58221355	58221749	PDE4D	74010
chr5	59096454	59096686	PDE4D	32016
chr5	61339493	61339750	KIF2A	262238
chr5	61865394	61865625	LRRC70	8936
chr5	63254625	63254855	HTR1A	3264
chr5	65277567	65277798	LOC100303749	36166
chr5	66125571	66125920	MAST4	968
chr5	67489689	67489999	PIK3R1	21584
chr5	73839542	73839830	ENC1	97419
chr5	74884544	74884896	COL4A3BP	76738
chr5	85936864	85937140	MIR3607	20551
chr5	87966949	87967183	LINC00461	1963
chr5	90391843	90392124	ARRDC3-AS1	284039
chr5	90634930	90635164	ARRDC3-AS1	40999
chr5	107414105	107414355	FBXL17	303444
chr5	108022449	108022693	HP07349	41269
chr5	109872185	109872504	TMEM232	189946
chr5	112023143	112023402	APC	19799
chr5	113472791	113473045	KCNN2	224970
chr5	116354120	116354396	LOC728342	396811
chr5	118806148	118806445	HSD17B4	17947
chr5	119952035	119952272	PRR16	152017

chr5	123804404	123804764	ZNF608	276041
chr5	123812705	123813111	ZNF608	267694
chr5	124316241	124316610	ZNF608	235436
chr5	124393412	124393654	ZNF608	312607
chr5	125393012	125393355	GRAMD3	302432
chr5	125684987	125685254	GRAMD3	10533
chr5	126592758	126593100	MEGF10	33355
chr5	134085155	134085385	DDX46	9075
chr5	137805504	137805781	EGR1	4324
chr5	137805904	137806298	EGR1	4724
chr5	138755565	138755890	SPATA24	15789
chr5	142261365	142261600	ARHGAP26-AS1	12890
chr5	142416670	142417018	ARHGAP26-AS1	168195
chr5	143697630	143697916	KCTD16	147194
chr5	145745295	145745530	POU4F3	26709
chr5	146712243	146712503	STK32A	97665
chr5	146901941	146902324	DPYSL3	12322
chr5	148331275	148331529	SH3TC2	111208
chr5	150862802	150863058	SLC36A1	35640
chr5	152590585	152590986	GRIA1	279097
chr5	153457803	153458084	MFAP3	39285
chr5	153465649	153465912	MFAP3	47131
chr5	158379141	158379371	EBF1	147417
chr5	159593401	159593675	FABP6	20698
chr5	167219677	167219920	WWC1	499144
chr5	168261144	168261374	MIR218-2	65884
chr5	169835294	169835547	KCNMB1	18656
chr5	173097089	173097477	BOD1	53423
chr5	174906485	174906860	SFXN1	972
chr5	175031341	175031590	HRH2	53449
chr5	176817565	176817795	SLC34A1	6134
chr6	1104545	1104803	LOC285768	2978
chr6	3356698	3356928	SLC22A23	88290
chr6	4654910	4655145	CDYL	51247
chr6	5378725	5379009	FARS2	117142
chr6	5412181	5412427	FARS2	150598
chr6	7087349	7087668	RREB1	20161
chr6	10417437	10417946	TFAP2A	1851
chr6	14009806	14010066	RNF182	84383
chr6	15895192	15895522	DTNBP1	231903
chr6	18303380	18303610	DEK	38581
chr6	19804831	19805151	ID4	32449
chr6	20484208	20484442	CDKAL1	50245
chr6	22897038	22897300	HDGFL1	327361
chr6	25636036	25636312	SCGN	16116
chr6	26158008	26158454	HIST1H2BD	0
chr6	26533102	26533344	HMGN4	5227
chr6	27819343	27819685	HIST1H2BN	12904
chr6	34213388	34213629	C6orf1	3275
chr6	34992570	34992896	TCP11	116291

chr6	35064445	35064743	TCP11	44444
chr6	36634826	36635118	CDKN1A	9118
chr6	37293542	37293797	RNF8	27950
chr6	37787092	37787322	ZFAND3	0
chr6	38445498	38445778	BTBD9	118065
chr6	39172590	39173005	KCNK5	24246
chr6	39311683	39311966	KCNK16	21353
chr6	41933392	41933806	CCND3	23840
chr6	42104341	42104575	C6orf132	6140
chr6	42209679	42210032	MRPS10	24046
chr6	42225449	42225727	MRPS10	39816
chr6	43826307	43826646	LOC100132354	32118
chr6	44221193	44221544	MIR4647	478
chr6	45866712	45867016	CLIC5	38318
chr6	48732714	48733054	PTCHD4	696289
chr6	49110186	49110584	MUT	320457
chr6	49998653	49999049	DEFB110	8959
chr6	51428681	51428950	PKHD1	523473
chr6	55142422	55142756	GFRAL	49510
chr6	68849117	68849438	BAI3	496193
chr6	76000416	76000710	LOC100506804	5687
chr6	80441201	80441504	RNY4	10131
chr6	86379716	86379965	SNORD50A	7121
chr6	89283655	89284179	RNGTT	389169
chr6	90211003	90211250	ANKRD6	60786
chr6	91145279	91145537	MIR4464	122819
chr6	95977675	95978053	MANEA	47319
chr6	96133899	96134141	MANEA	108527
chr6	100615549	100615832	MCHR2	173435
chr6	102148685	102148931	GRIK2	301825
chr6	108191065	108191313	SCML4	45544
chr6	108501539	108501895	NR2E1	14325
chr6	108960324	108960615	FOXO3	78256
chr6	108988085	108988345	LINC00222	84511
chr6	110418666	110418940	WASF1	82267
chr6	115736532	115736918	TPI1P3	622975
chr6	118273014	118273337	SLC35F1	44326
chr6	121426731	121427116	C6orf170	228528
chr6	124815124	124815441	STL	468732
chr6	129639505	129639758	ARHGAP18	391612
chr6	131483320	131483628	AKAP7	26495
chr6	133151662	133151942	SNORA33	13305
chr6	134237761	134238102	TCF21	27502
chr6	134633319	134633551	SGK1	5645
chr6	137868339	137868693	OLIG3	52808
chr6	138388609	138388878	PERP	39782
chr6	139299056	139299357	REPS1	10041
chr6	139717284	139717608	CITED2	21497
chr6	139801934	139802286	LOC645434	6201
chr6	139932670	139932986	LOC645434	136937

chr6	143610053	143610361	ADAT2	161480
chr6	144965932	144966282	UTRN	353060
chr6	148921216	148921464	UST	146806
chr6	149204003	149204321	LOC100128176	81499
chr6	152128585	152128891	ESR1	0
chr6	153225770	153226042	FBXO5	78161
chr6	155470064	155470367	TIAM2	58642
chr6	158889033	158889321	TMEM181	68146
chr6	159101966	159102329	SYTL3	30921
chr6	159274477	159274800	OSTCP1	3864
chr6	161713178	161713517	AGPAT4	18071
chr6	163278040	163278300	PACRG	129039
chr6	168572488	168572719	FRMD1	92649
chr6	169499829	169500127	THBS2	154010
chr6	169670173	169670412	THBS2	16036
chr6	170019231	170019488	WDR27	82671
chr7	2861006	2861244	GNA12	22715
chr7	3439585	3439835	SDK1	98506
chr7	5566897	5567308	ACTB	2924
chr7	6941703	6942104	CCZ1B	75777
chr7	12463963	12464249	VWDE	20111
chr7	16678313	16678584	ANKMY2	6858
chr7	17352243	17352473	AHR	13968
chr7	22135535	22135765	CDCA7L	149993
chr7	24659665	24659928	MPP6	46581
chr7	24725428	24725724	DFNA5	71359
chr7	26141341	26141571	NFE2L3	50275
chr7	26231557	26232043	HNRNPA2B1	8370
chr7	27949995	27950248	TAX1BP1	170282
chr7	31014523	31014756	GHRHR	10888
chr7	31601629	31602005	CCDC129	9014
chr7	31807475	31807798	PPP1R17	80845
chr7	40787547	40787889	C7orf10	612973
chr7	41854574	41854804	INHBA	111868
chr7	42933558	42933793	C7orf25	17896
chr7	47720278	47720560	C7orf65	25437
chr7	48078542	48078812	C7orf57	3435
chr7	51148624	51148855	COBL	235660
chr7	51378822	51379057	COBL	5458
chr7	52129321	52129713	COBL	744806
chr7	52182229	52182485	COBL	797714
chr7	52547770	52548012	POM121L12	555336
chr7	53394396	53394680	POM121L12	291048
chr7	70938126	70938359	MIR3914-1	165370
chr7	76302760	76303019	POMZP3	46140
chr7	80874742	80875029	SEMA3C	326075
chr7	82389493	82389818	CACNA2D1	316462
chr7	86758735	86759121	DMTF1	22555
chr7	90581972	90582202	CDK14	243261
chr7	90997535	90997805	FZD1	103753

chr7	96622531	96622922	DLX6	12367
chr7	105279650	105280011	ATXN7L1	39598
chr7	107599930	107600268	LAMB1	43536
chr7	111053744	111054106	IMMP2L	148241
chr7	115456409	115456841	TFEC	151526
chr7	116256876	116257175	MET	55283
chr7	120358415	120358784	TSPAN12	139393
chr7	122078008	122078423	FEZF1	133443
chr7	122161198	122161473	RNF133	177735
chr7	123080252	123080551	IQUB	94167
chr7	123634558	123634827	TMEM229A	38696
chr7	130080951	130081201	CEP41	0
chr7	132303298	132303542	PLXNA4	29905
chr7	135348647	135348878	C7orf73	1427
chr7	136661259	136661489	MIR490	73346
chr7	137432304	137432580	DGKI	99029
chr7	138143518	138143806	TRIM24	1272
chr7	141244696	141245050	AGK	6027
chr7	147685285	147685544	MIR548T	58601
chr7	151326283	151326654	PRKAG2	2690
chr7	155019590	155019952	INSIG1	69533
chr7	156377856	156378487	LINC00244	44672
chr7	158262454	158262765	MIR595	62740
chr7	158818932	158819353	LOC154822	17888
chr7_gl000195_random	30623	30985	-1	-1
chr7_gl000195_random	31237	31513	-1	-1
chr7_gl000195_random	31768	32177	-1	-1
chr8	1267065	1267317	LOC286083	16238
chr8	1750918	1751257	MIR596	14139
chr8	5023674	5023943	CSMD1	171346
chr8	8601417	8601763	CLDN23	41752
chr8	13099098	13099353	DLC1	108289
chr8	16060223	16060474	MSR1	9923
chr8	16141755	16142163	MSR1	91455
chr8	16438661	16438907	MSR1	388361
chr8	19717912	19718142	INTS10	42995
chr8	20126298	20126554	LZTS1-AS1	6729
chr8	22814833	22815213	PEBP4	29412
chr8	23719470	23719731	STC1	7150
chr8	26386411	26386654	DPYSL2	14703
chr8	32315070	32315378	NRG1-IT3	16809
chr8	33276760	33277169	FUT10	53495
chr8	34704393	34704726	UNC5D	388248
chr8	37330421	37330698	ZNF703	222602
chr8	39955515	39955748	C8orf4	55238
chr8	41165279	41165526	SFRP1	1464
chr8	41249533	41249813	SFRP1	82543
chr8	41997737	41998037	AP3M2	12426
chr8	52282274	52282644	PXDNL	439361
chr8	54817030	54817265	RGS20	23589

chr8	59461555	59461828	SDCBP	3899
chr8	59942170	59942438	TOX	89329
chr8	62752218	62752565	MIR4470	124872
chr8	66131700	66131937	LINC00251	39125
chr8	71061217	71061469	PRDM14	77655
chr8	72628506	72628810	LOC100132891	126547
chr8	73687024	73687319	TERF1	233777
chr8	74659048	74659301	STAU2	0
chr8	80035258	80035604	IL7	317500
chr8	83976427	83976784	RALYL	1118668
chr8	87715687	87715969	CNGB3	39934
chr8	89124529	89124876	MMP16	214841
chr8	89230026	89230339	MMP16	109378
chr8	89604347	89604661	MMP16	264630
chr8	94229793	94230105	C8orf87	50714
chr8	97136204	97136470	GDF6	36550
chr8	100084527	100084898	VPS13B	59034
chr8	102472915	102473146	GRHL2	31521
chr8	103266617	103266875	RRM2B	15271
chr8	104118082	104118327	BAALC	34593
chr8	117050902	117051169	LINC00536	286128
chr8	118382257	118382658	MED30	150306
chr8	121060430	121060683	COL14A1	76668
chr8	127908628	127908972	PCAT1	116426
chr8	128101377	128101698	PCAT1	75979
chr8	129867231	129867475	LOC728724	386011
chr8	129994936	129995229	LOC728724	258257
chr8	140277552	140277820	COL22A1	351316
chr8	143101884	143102141	MIR4472-1	155558
chr8	146064530	146064781	ZNF7	11628
chr9	2812846	2813142	KIAA0020	30988
chr9	13492069	13492330	FLJ41200	60741
chr9	14547337	14547593	ZDHHC21	145887
chr9	15354940	15355170	TTC39B	47582
chr9	18379311	18379541	ADAMTSL1	94537
chr9	20664612	20664875	FOCAD	6304
chr9	22863864	22864119	FLJ35282	217666
chr9	26742910	26743195	CAAP1	149631
chr9	37339830	37340199	GRHPR	82507
chr9	38084365	38084709	SHB	15155
chr9	71487248	71487478	FAM122A	92285
chr9	74367886	74368163	TMEM2	15637
chr9	74600436	74600745	C9orf85	74014
chr9	75342028	75342321	TMC1	205312
chr9	76462613	76462846	RORB	649405
chr9	79209705	79210020	GCNT1	94157
chr9	82577752	82578072	TLE4	390875
chr9	84264429	84264687	TLE1	38909
chr9	84265862	84266145	TLE1	37451
chr9	86766237	86766467	RMI1	170601

chr9	87461989	87462219	NTRK2	177364
chr9	90230769	90231058	CTSL1	109915
chr9	90640966	90641345	CDK20	51271
chr9	90776114	90776429	FAM75C2	26214
chr9	92050001	92050439	SEMA4D	44172
chr9	94334457	94334730	MIR3910-1	63802
chr9	95674339	95674650	ZNF484	34019
chr9	95715217	95715526	FGD3	5617
chr9	95751752	95752228	FGD3	15035
chr9	112789981	112790295	AKAP2	20582
chr9	115073122	115073362	PTBP3	21944
chr9	120031181	120031477	SNORA70C	87701
chr9	120131240	120131508	ASTN2	45809
chr9	120311860	120312177	ASTN2	134543
chr9	124148639	124149045	STOM	16094
chr9	129874374	129874636	ANGPTL2	10408
chr9	131171219	131171455	CERCAM	11303
chr9	131941212	131941517	IER5L	672
chr9	132948074	132948304	NCS1	13218
chr9	137867039	137867416	FCN1	57233
chr9	139440689	139441068	MIR4674	0
chr9	140222674	140222988	NRARP	25971
chrX	3038712	3038995	ARSF	53873
chrX	13149780	13150065	FAM9C	86863
chrX	13220004	13220322	LOC100093698	108448
chrX	17555878	17556113	NHS	97299
chrX	20581223	20581691	RPS6KA3	296473
chrX	21765725	21766118	SMPX	10160
chrX	22464046	22464381	ZNF645	173017
chrX	23523692	23523959	PRDX4	161685
chrX	23971067	23971369	CXorf58	44945
chrX	28145144	28145407	DCAF8L1	145578
chrX	28176639	28176926	DCAF8L1	177073
chrX	28516913	28517154	IL1RAPL1	88526
chrX	28938793	28939125	IL1RAPL1	333113
chrX	29326922	29327307	IL1RAPL1	721242
chrX	29327437	29327700	IL1RAPL1	721757
chrX	30759747	30760071	GK	88272
chrX	31252118	31252354	DMD	32670
chrX	34621032	34621503	TMEM47	53902
chrX	37336044	37336423	LANCL3	94398
chrX	37439337	37439601	LANCL3	8516
chrX	38924730	38925012	LOC286442	261604
chrX	40016721	40016978	BCOR	19604
chrX	43477759	43478071	MAOA	36083
chrX	46187248	46187496	ZNF673	119127
chrX	46226217	46226672	ZNF673	79951
chrX	46348237	46348524	ZNF673	41614
chrX	47175652	47175937	ZNF157	54061
chrX	48754386	48754698	PQBP1	496

chrX	48897618	48897856	TFE3	3134
chrX	50454937	50455200	SHROOM4	101844
chrX	54830694	54830924	MAGED2	3246
chrX	68073727	68074029	EFNB1	24888
chrX	68123040	68123288	EFNB1	74201
chrX	68369613	68370005	PJA1	15360
chrX	68441487	68441722	PJA1	56122
chrX	68441839	68442120	PJA1	56474
chrX	68616700	68616936	FAM155B	108141
chrX	71401202	71401628	PIN4	0
chrX	73199572	73199858	JPX	35414
chrX	73545388	73545798	ZCCHC13	21364
chrX	82768864	82769280	POU3F4	5596
chrX	85518620	85518850	DACH2	650
chrX	92807952	92808319	NAP1L3	120363
chrX	95738677	95739083	LOC643486	145776
chrX	96101902	96102232	RPA4	36674
chrX	96894570	96894808	RPA4	755664
chrX	97861000	97861288	LOC442459	1333553
chrX	104543991	104544255	TEX13A	78633
chrX	107609917	107610206	COL4A6	71452
chrX	107669503	107669861	COL4A6	11797
chrX	110208060	110208290	PAK3	20548
chrX	116461295	116461563	KLHL13	646138
chrX	119274231	119274470	RHOXF2B	17996
chrX	119466276	119466507	FAM70A	20885
chrX	119947941	119948221	CT47B1	61558
chrX	123363226	123363469	SH2D1A	116662
chrX	128322379	128322644	SMARCA1	334816
chrX	129273782	129274085	AIFM1	1767
chrX	129872676	129873015	ENOX2	164193
chrX	131546388	131546628	MBNL3	975
chrX	132399293	132399545	TFDP3	46917
chrX	132441185	132441488	TFDP3	88809
chrX	132441867	132442099	TFDP3	89491
chrX	132690049	132690507	GPC4	140844
chrX	133047781	133048213	GPC3	71460
chrX	134407178	134407443	ZNF75D	22522
chrX	134589761	134589991	LINC00086	33894
chrX	135876359	135876619	ARHGEF6	12856
chrX	136831157	136831411	ZIC3	182812
chrX	137083445	137083798	ZIC3	435100
chrX	138307114	138307418	FGF13	19929
chrX	138936094	138936326	ATP11C	21647
chrX	148763786	148764081	MAGEA11	5821
chrX	148956866	148957184	MAGEA8	52756
chrX	149364531	149364839	MIR2114	31399
chrX	149735224	149735539	MTM1	1507
chrX	151809734	151810054	GABRQ	3098
chrY	21154426	21154857	CD24	0
