# Supplementary material for: Inability to switch from ARID1A-BAF to ARID1B-BAF impairs exit from pluripotency and commitment towards neural crest formation in ARID1B-related neurodevelopmental disorders
Source: Nat Commun. 2021 Nov 9;12:6469. doi: 10.1038/s41467-021-26810-x (PMC8578637; doi:10.1038/s41467-021-26810-x)
Supplement: Supplementary file 9 — Supplementary Data 6 [file 41467_2021_26810_MOESM9_ESM.pdf]

#Supplemental_File_S6: PATIENT-SPECIFIC NANOG_PEAKS

| CHR | START | END | PEAK_NAME |
|---|---|---|---|
| chr1 | 781045 | 781329 | P19_NANOG_CNCC_D5_mem_q10_srt_rmdup_peaks_peak_2 |
| chr1 | 1503327 | 1503697 | P19_NANOG_CNCC_D5_mem_q10_srt_rmdup_peaks_peak_11 |
| chr1 | 1790728 | 1791087 | P19_NANOG_CNCC_D5_mem_q10_srt_rmdup_peaks_peak_14 |
| chr1 | 1891489 | 1892055 | P19_NANOG_CNCC_D5_mem_q10_srt_rmdup_peaks_peak_17 |
| chr1 | 3594801 | 3595097 | P19_NANOG_CNCC_D5_mem_q10_srt_rmdup_peaks_peak_30 |
| chr1 | 6112598 | 6112902 | P19_NANOG_CNCC_D5_mem_q10_srt_rmdup_peaks_peak_37 |
| chr1 | 6208748 | 6209095 | P19_NANOG_CNCC_D5_mem_q10_srt_rmdup_peaks_peak_38 |
| chr1 | 6265164 | 6265577 | P19_NANOG_CNCC_D5_mem_q10_srt_rmdup_peaks_peak_40 |
| chr1 | 7039255 | 7039707 | P19_NANOG_CNCC_D5_mem_q10_srt_rmdup_peaks_peak_53 |
| chr1 | 7237985 | 7238346 | P19_NANOG_CNCC_D5_mem_q10_srt_rmdup_peaks_peak_58 |
| chr1 | 8215648 | 8215977 | P19_NANOG_CNCC_D5_mem_q10_srt_rmdup_peaks_peak_72 |
| chr1 | 8230262 | 8230567 | P19_NANOG_CNCC_D5_mem_q10_srt_rmdup_peaks_peak_74 |
| chr1 | 8467955 | 8468226 | P19_NANOG_CNCC_D5_mem_q10_srt_rmdup_peaks_peak_78 |
| chr1 | 8623978 | 8624460 | P19_NANOG_CNCC_D5_mem_q10_srt_rmdup_peaks_peak_84 |
| chr1 | 10859930 | 10860221 | P19_NANOG_CNCC_D5_mem_q10_srt_rmdup_peaks_peak_123 |
| chr1 | 12652532 | 12652860 | P19_NANOG_CNCC_D5_mem_q10_srt_rmdup_peaks_peak_155 |
| chr1 | 13882817 | 13883196 | P19_NANOG_CNCC_D5_mem_q10_srt_rmdup_peaks_peak_162 |
| chr1 | 14928232 | 14928623 | P19_NANOG_CNCC_D5_mem_q10_srt_rmdup_peaks_peak_174 |
| chr1 | 15214349 | 15214666 | P19_NANOG_CNCC_D5_mem_q10_srt_rmdup_peaks_peak_179 |
| chr1 | 15598886 | 15599194 | P19_NANOG_CNCC_D5_mem_q10_srt_rmdup_peaks_peak_187 |
| chr1 | 16177713 | 16178004 | P19_NANOG_CNCC_D5_mem_q10_srt_rmdup_peaks_peak_197 |
| chr1 | 16280473 | 16280878 | P19_NANOG_CNCC_D5_mem_q10_srt_rmdup_peaks_peak_198 |
| chr1 | 16678783 | 16679257 | |

P19_NANOG_CNCC_D5_mem_q10_srt_rmdup_peaks_peak_208
chr1 16825211 16825668
P19_NANOG_CNCC_D5_mem_q10_srt_rmdup_peaks_peak_210
chr1 17215998 17216269
P19_NANOG_CNCC_D5_mem_q10_srt_rmdup_peaks_peak_218
chr1 17339440 17339713
P19_NANOG_CNCC_D5_mem_q10_srt_rmdup_peaks_peak_225
chr1 17454791 17455219
P19_NANOG_CNCC_D5_mem_q10_srt_rmdup_peaks_peak_227
chr1 17914147 17914528
P19_NANOG_CNCC_D5_mem_q10_srt_rmdup_peaks_peak_238
chr1 18564008 18564316
P19_NANOG_CNCC_D5_mem_q10_srt_rmdup_peaks_peak_247
chr1 18979427 18979796
P19_NANOG_CNCC_D5_mem_q10_srt_rmdup_peaks_peak_268
chr1 19111381 19111799
P19_NANOG_CNCC_D5_mem_q10_srt_rmdup_peaks_peak_270
chr1 19283235 19283601
P19_NANOG_CNCC_D5_mem_q10_srt_rmdup_peaks_peak_274
chr1 19453378 19453886
P19_NANOG_CNCC_D5_mem_q10_srt_rmdup_peaks_peak_277
chr1 20617077 20617525
P19_NANOG_CNCC_D5_mem_q10_srt_rmdup_peaks_peak_296
chr1 21023498 21023922
P19_NANOG_CNCC_D5_mem_q10_srt_rmdup_peaks_peak_305
chr1 21974342 21974637
P19_NANOG_CNCC_D5_mem_q10_srt_rmdup_peaks_peak_319
chr1 21985641 21986096
P19_NANOG_CNCC_D5_mem_q10_srt_rmdup_peaks_peak_320
chr1 22052501 22052865
P19_NANOG_CNCC_D5_mem_q10_srt_rmdup_peaks_peak_322
chr1 22720806 22721158
P19_NANOG_CNCC_D5_mem_q10_srt_rmdup_peaks_peak_330
chr1 23939228 23939851
P19_NANOG_CNCC_D5_mem_q10_srt_rmdup_peaks_peak_358
chr1 24477016 24477956
P19_NANOG_CNCC_D5_mem_q10_srt_rmdup_peaks_peak_363
chr1 24648077 24648569
P19_NANOG_CNCC_D5_mem_q10_srt_rmdup_peaks_peak_368
chr1 24882159 24882600
P19_NANOG_CNCC_D5_mem_q10_srt_rmdup_peaks_peak_375
chr1 25087163 25087695
P19_NANOG_CNCC_D5_mem_q10_srt_rmdup_peaks_peak_381
chr1 25828216 25828496
P19_NANOG_CNCC_D5_mem_q10_srt_rmdup_peaks_peak_394
chr1 26758397 26758833
P19_NANOG_CNCC_D5_mem_q10_srt_rmdup_peaks_peak_411
chr1 27324509 27324829
P19_NANOG_CNCC_D5_mem_q10_srt_rmdup_peaks_peak_423
chr1 27989666 27989937

P19_NANOG_CNCC_D5_mem_q10_srt_rmdup_peaks_peak_446
chr1 28051841 28052317
P19_NANOG_CNCC_D5_mem_q10_srt_rmdup_peaks_peak_448
chr1 29179760 29180107
P19_NANOG_CNCC_D5_mem_q10_srt_rmdup_peaks_peak_467
chr1 29208689 29209012
P19_NANOG_CNCC_D5_mem_q10_srt_rmdup_peaks_peak_468
chr1 29292082 29292439
P19_NANOG_CNCC_D5_mem_q10_srt_rmdup_peaks_peak_471
chr1 29329881 29330194
P19_NANOG_CNCC_D5_mem_q10_srt_rmdup_peaks_peak_472
chr1 29545119 29545621
P19_NANOG_CNCC_D5_mem_q10_srt_rmdup_peaks_peak_477
chr1 30819456 30819777
P19_NANOG_CNCC_D5_mem_q10_srt_rmdup_peaks_peak_490
chr1 31839719 31840005
P19_NANOG_CNCC_D5_mem_q10_srt_rmdup_peaks_peak_502
chr1 32909868 32910232
P19_NANOG_CNCC_D5_mem_q10_srt_rmdup_peaks_peak_513
chr1 33098511 33098785
P19_NANOG_CNCC_D5_mem_q10_srt_rmdup_peaks_peak_516
chr1 33530915 33531211
P19_NANOG_CNCC_D5_mem_q10_srt_rmdup_peaks_peak_528
chr1 33592674 33593039
P19_NANOG_CNCC_D5_mem_q10_srt_rmdup_peaks_peak_529
chr1 33814057 33814379
P19_NANOG_CNCC_D5_mem_q10_srt_rmdup_peaks_peak_536
chr1 33894990 33895360
P19_NANOG_CNCC_D5_mem_q10_srt_rmdup_peaks_peak_538
chr1 34333410 34333853
P19_NANOG_CNCC_D5_mem_q10_srt_rmdup_peaks_peak_542
chr1 35064199 35064486
P19_NANOG_CNCC_D5_mem_q10_srt_rmdup_peaks_peak_552
chr1 35122385 35122762
P19_NANOG_CNCC_D5_mem_q10_srt_rmdup_peaks_peak_553
chr1 35936746 35937277
P19_NANOG_CNCC_D5_mem_q10_srt_rmdup_peaks_peak_565
chr1 36851496 36851826
P19_NANOG_CNCC_D5_mem_q10_srt_rmdup_peaks_peak_574
chr1 36852450 36852721
P19_NANOG_CNCC_D5_mem_q10_srt_rmdup_peaks_peak_575
chr1 36989356 36989836
P19_NANOG_CNCC_D5_mem_q10_srt_rmdup_peaks_peak_577
chr1 37389875 37390238
P19_NANOG_CNCC_D5_mem_q10_srt_rmdup_peaks_peak_587
chr1 37700411 37700904
P19_NANOG_CNCC_D5_mem_q10_srt_rmdup_peaks_peak_593
chr1 38619160 38619446
P19_NANOG_CNCC_D5_mem_q10_srt_rmdup_peaks_peak_608
chr1 38709885 38710235

P19_NANOG_CNCC_D5_mem_q10_srt_rmdup_peaks_peak_609
chr1 39110697 39111125
P19_NANOG_CNCC_D5_mem_q10_srt_rmdup_peaks_peak_622
chr1 39564148 39564520
P19_NANOG_CNCC_D5_mem_q10_srt_rmdup_peaks_peak_633
chr1 39835118 39835403
P19_NANOG_CNCC_D5_mem_q10_srt_rmdup_peaks_peak_646
chr1 39864397 39864716
P19_NANOG_CNCC_D5_mem_q10_srt_rmdup_peaks_peak_647
chr1 40303495 40303883
P19_NANOG_CNCC_D5_mem_q10_srt_rmdup_peaks_peak_656
chr1 40562762 40563196
P19_NANOG_CNCC_D5_mem_q10_srt_rmdup_peaks_peak_666
chr1 41134489 41134876
P19_NANOG_CNCC_D5_mem_q10_srt_rmdup_peaks_peak_668
chr1 41853327 41853607
P19_NANOG_CNCC_D5_mem_q10_srt_rmdup_peaks_peak_677
chr1 43124010 43124281
P19_NANOG_CNCC_D5_mem_q10_srt_rmdup_peaks_peak_685
chr1 43599841 43600125
P19_NANOG_CNCC_D5_mem_q10_srt_rmdup_peaks_peak_694
chr1 43751060 43751331
P19_NANOG_CNCC_D5_mem_q10_srt_rmdup_peaks_peak_697
chr1 44572400 44572835
P19_NANOG_CNCC_D5_mem_q10_srt_rmdup_peaks_peak_716
chr1 45049778 45050327
P19_NANOG_CNCC_D5_mem_q10_srt_rmdup_peaks_peak_732
chr1 45265722 45266100
P19_NANOG_CNCC_D5_mem_q10_srt_rmdup_peaks_peak_737
chr1 45769617 45769956
P19_NANOG_CNCC_D5_mem_q10_srt_rmdup_peaks_peak_742
chr1 46347201 46347547
P19_NANOG_CNCC_D5_mem_q10_srt_rmdup_peaks_peak_753
chr1 46751738 46752026
P19_NANOG_CNCC_D5_mem_q10_srt_rmdup_peaks_peak_760
chr1 46795677 46796105
P19_NANOG_CNCC_D5_mem_q10_srt_rmdup_peaks_peak_761
chr1 46930667 46931000
P19_NANOG_CNCC_D5_mem_q10_srt_rmdup_peaks_peak_764
chr1 46959920 46960385
P19_NANOG_CNCC_D5_mem_q10_srt_rmdup_peaks_peak_767
chr1 46985711 46986022
P19_NANOG_CNCC_D5_mem_q10_srt_rmdup_peaks_peak_768
chr1 47134082 47134397
P19_NANOG_CNCC_D5_mem_q10_srt_rmdup_peaks_peak_772
chr1 47223084 47223470
P19_NANOG_CNCC_D5_mem_q10_srt_rmdup_peaks_peak_775
chr1 47899670 47900010
P19_NANOG_CNCC_D5_mem_q10_srt_rmdup_peaks_peak_784
chr1 48490667 48491217

P19_NANOG_CNCC_D5_mem_q10_srt_rmdup_peaks_peak_798
chr1 48937548 48937875
P19_NANOG_CNCC_D5_mem_q10_srt_rmdup_peaks_peak_804
chr1 49350269 49350832
P19_NANOG_CNCC_D5_mem_q10_srt_rmdup_peaks_peak_807
chr1 49358198 49358481
P19_NANOG_CNCC_D5_mem_q10_srt_rmdup_peaks_peak_808
chr1 50882326 50882661
P19_NANOG_CNCC_D5_mem_q10_srt_rmdup_peaks_peak_812
chr1 51099073 51099388
P19_NANOG_CNCC_D5_mem_q10_srt_rmdup_peaks_peak_822
chr1 51116320 51116714
P19_NANOG_CNCC_D5_mem_q10_srt_rmdup_peaks_peak_823
chr1 52039695 52040054
P19_NANOG_CNCC_D5_mem_q10_srt_rmdup_peaks_peak_832
chr1 52799009 52799309
P19_NANOG_CNCC_D5_mem_q10_srt_rmdup_peaks_peak_836
chr1 53298174 53298458
P19_NANOG_CNCC_D5_mem_q10_srt_rmdup_peaks_peak_842
chr1 53863966 53864237
P19_NANOG_CNCC_D5_mem_q10_srt_rmdup_peaks_peak_849
chr1 54379025 54379328
P19_NANOG_CNCC_D5_mem_q10_srt_rmdup_peaks_peak_858
chr1 54738132 54738403
P19_NANOG_CNCC_D5_mem_q10_srt_rmdup_peaks_peak_860
chr1 54844238 54844640
P19_NANOG_CNCC_D5_mem_q10_srt_rmdup_peaks_peak_866
chr1 54968131 54968470
P19_NANOG_CNCC_D5_mem_q10_srt_rmdup_peaks_peak_869
chr1 55352714 55353167
P19_NANOG_CNCC_D5_mem_q10_srt_rmdup_peaks_peak_876
chr1 56145247 56145930
P19_NANOG_CNCC_D5_mem_q10_srt_rmdup_peaks_peak_882
chr1 57154044 57154618
P19_NANOG_CNCC_D5_mem_q10_srt_rmdup_peaks_peak_901
chr1 57637833 57638253
P19_NANOG_CNCC_D5_mem_q10_srt_rmdup_peaks_peak_911
chr1 57735542 57735857
P19_NANOG_CNCC_D5_mem_q10_srt_rmdup_peaks_peak_915
chr1 58501158 58501593
P19_NANOG_CNCC_D5_mem_q10_srt_rmdup_peaks_peak_928
chr1 60144520 60144815
P19_NANOG_CNCC_D5_mem_q10_srt_rmdup_peaks_peak_949
chr1 62647941 62648233
P19_NANOG_CNCC_D5_mem_q10_srt_rmdup_peaks_peak_990
chr1 62661468 62662007
P19_NANOG_CNCC_D5_mem_q10_srt_rmdup_peaks_peak_992
chr1 63002623 63002962
P19_NANOG_CNCC_D5_mem_q10_srt_rmdup_peaks_peak_1000
chr1 63582916 63583365

P19_NANOG_CNCC_D5_mem_q10_srt_rmdup_peaks_peak_1008
chr1 63833002 63833345
P19_NANOG_CNCC_D5_mem_q10_srt_rmdup_peaks_peak_1018
chr1 64323773 64324241
P19_NANOG_CNCC_D5_mem_q10_srt_rmdup_peaks_peak_1032
chr1 64620890 64621353
P19_NANOG_CNCC_D5_mem_q10_srt_rmdup_peaks_peak_1039
chr1 64642360 64642722
P19_NANOG_CNCC_D5_mem_q10_srt_rmdup_peaks_peak_1040
chr1 64809058 64809358
P19_NANOG_CNCC_D5_mem_q10_srt_rmdup_peaks_peak_1043
chr1 65802578 65803010
P19_NANOG_CNCC_D5_mem_q10_srt_rmdup_peaks_peak_1063
chr1 66016098 66016495
P19_NANOG_CNCC_D5_mem_q10_srt_rmdup_peaks_peak_1067
chr1 66620121 66620392
P19_NANOG_CNCC_D5_mem_q10_srt_rmdup_peaks_peak_1070
chr1 66815665 66816281
P19_NANOG_CNCC_D5_mem_q10_srt_rmdup_peaks_peak_1071
chr1 66820243 66820535
P19_NANOG_CNCC_D5_mem_q10_srt_rmdup_peaks_peak_1072
chr1 67484807 67485117
P19_NANOG_CNCC_D5_mem_q10_srt_rmdup_peaks_peak_1077
chr1 68112079 68112591
P19_NANOG_CNCC_D5_mem_q10_srt_rmdup_peaks_peak_1092
chr1 69700084 69700366
P19_NANOG_CNCC_D5_mem_q10_srt_rmdup_peaks_peak_1107
chr1 70606555 70606958
P19_NANOG_CNCC_D5_mem_q10_srt_rmdup_peaks_peak_1112
chr1 70908222 70908605
P19_NANOG_CNCC_D5_mem_q10_srt_rmdup_peaks_peak_1115
chr1 71650069 71650340
P19_NANOG_CNCC_D5_mem_q10_srt_rmdup_peaks_peak_1117
chr1 72078588 72078869
P19_NANOG_CNCC_D5_mem_q10_srt_rmdup_peaks_peak_1119
chr1 73653293 73653593
P19_NANOG_CNCC_D5_mem_q10_srt_rmdup_peaks_peak_1128
chr1 74445297 74446032
P19_NANOG_CNCC_D5_mem_q10_srt_rmdup_peaks_peak_1131
chr1 74974898 74975175
P19_NANOG_CNCC_D5_mem_q10_srt_rmdup_peaks_peak_1135
chr1 75140322 75140761
P19_NANOG_CNCC_D5_mem_q10_srt_rmdup_peaks_peak_1138
chr1 75195829 75196395
P19_NANOG_CNCC_D5_mem_q10_srt_rmdup_peaks_peak_1139
chr1 75549758 75550150
P19_NANOG_CNCC_D5_mem_q10_srt_rmdup_peaks_peak_1142
chr1 75595694 75596017
P19_NANOG_CNCC_D5_mem_q10_srt_rmdup_peaks_peak_1143
chr1 75607872 75608212

P19_NANOG_CNCC_D5_mem_q10_srt_rmdup_peaks_peak_1145
chr1 76169340 76169717
P19_NANOG_CNCC_D5_mem_q10_srt_rmdup_peaks_peak_1148
chr1 76540063 76540610
P19_NANOG_CNCC_D5_mem_q10_srt_rmdup_peaks_peak_1153
chr1 78729250 78729631
P19_NANOG_CNCC_D5_mem_q10_srt_rmdup_peaks_peak_1170
chr1 80923159 80923499
P19_NANOG_CNCC_D5_mem_q10_srt_rmdup_peaks_peak_1184
chr1 81609793 81610262
P19_NANOG_CNCC_D5_mem_q10_srt_rmdup_peaks_peak_1189
chr1 81622619 81622973
P19_NANOG_CNCC_D5_mem_q10_srt_rmdup_peaks_peak_1190
chr1 81704052 81704405
P19_NANOG_CNCC_D5_mem_q10_srt_rmdup_peaks_peak_1191
chr1 81710728 81711574
P19_NANOG_CNCC_D5_mem_q10_srt_rmdup_peaks_peak_1192
chr1 82044763 82045085
P19_NANOG_CNCC_D5_mem_q10_srt_rmdup_peaks_peak_1200
chr1 82164924 82165281
P19_NANOG_CNCC_D5_mem_q10_srt_rmdup_peaks_peak_1204
chr1 82402708 82402979
P19_NANOG_CNCC_D5_mem_q10_srt_rmdup_peaks_peak_1211
chr1 82686430 82686701
P19_NANOG_CNCC_D5_mem_q10_srt_rmdup_peaks_peak_1215
chr1 83325472 83325795
P19_NANOG_CNCC_D5_mem_q10_srt_rmdup_peaks_peak_1219
chr1 84420240 84420512
P19_NANOG_CNCC_D5_mem_q10_srt_rmdup_peaks_peak_1225
chr1 84501382 84501670
P19_NANOG_CNCC_D5_mem_q10_srt_rmdup_peaks_peak_1226
chr1 84570154 84570452
P19_NANOG_CNCC_D5_mem_q10_srt_rmdup_peaks_peak_1227
chr1 84768542 84768870
P19_NANOG_CNCC_D5_mem_q10_srt_rmdup_peaks_peak_1231
chr1 85077360 85077707
P19_NANOG_CNCC_D5_mem_q10_srt_rmdup_peaks_peak_1237
chr1 85705945 85706349
P19_NANOG_CNCC_D5_mem_q10_srt_rmdup_peaks_peak_1249
chr1 87169727 87170003
P19_NANOG_CNCC_D5_mem_q10_srt_rmdup_peaks_peak_1266
chr1 87289590 87289993
P19_NANOG_CNCC_D5_mem_q10_srt_rmdup_peaks_peak_1268
chr1 87325700 87326027
P19_NANOG_CNCC_D5_mem_q10_srt_rmdup_peaks_peak_1270
chr1 87457527 87457801
P19_NANOG_CNCC_D5_mem_q10_srt_rmdup_peaks_peak_1273
chr1 87617642 87617923
P19_NANOG_CNCC_D5_mem_q10_srt_rmdup_peaks_peak_1278
chr1 89032194 89032476

P19_NANOG_CNCC_D5_mem_q10_srt_rmdup_peaks_peak_1299
chr1 89113118 89113419
P19_NANOG_CNCC_D5_mem_q10_srt_rmdup_peaks_peak_1300
chr1 89203847 89204208
P19_NANOG_CNCC_D5_mem_q10_srt_rmdup_peaks_peak_1302
chr1 89487873 89488183
P19_NANOG_CNCC_D5_mem_q10_srt_rmdup_peaks_peak_1305
chr1 90377738 90378009
P19_NANOG_CNCC_D5_mem_q10_srt_rmdup_peaks_peak_1317
chr1 90421190 90421542
P19_NANOG_CNCC_D5_mem_q10_srt_rmdup_peaks_peak_1321
chr1 91081949 91082229
P19_NANOG_CNCC_D5_mem_q10_srt_rmdup_peaks_peak_1329
chr1 92258420 92258709
P19_NANOG_CNCC_D5_mem_q10_srt_rmdup_peaks_peak_1362
chr1 92545753 92546350
P19_NANOG_CNCC_D5_mem_q10_srt_rmdup_peaks_peak_1365
chr1 92655753 92656237
P19_NANOG_CNCC_D5_mem_q10_srt_rmdup_peaks_peak_1366
chr1 93703968 93704454
P19_NANOG_CNCC_D5_mem_q10_srt_rmdup_peaks_peak_1377
chr1 95089382 95089767
P19_NANOG_CNCC_D5_mem_q10_srt_rmdup_peaks_peak_1401
chr1 95134148 95134447
P19_NANOG_CNCC_D5_mem_q10_srt_rmdup_peaks_peak_1404
chr1 95348060 95348376
P19_NANOG_CNCC_D5_mem_q10_srt_rmdup_peaks_peak_1406
chr1 95583345 95583616
P19_NANOG_CNCC_D5_mem_q10_srt_rmdup_peaks_peak_1414
chr1 97025566 97025837
P19_NANOG_CNCC_D5_mem_q10_srt_rmdup_peaks_peak_1433
chr1 98457795 98458066
P19_NANOG_CNCC_D5_mem_q10_srt_rmdup_peaks_peak_1445
chr1 100352314 100352585
P19_NANOG_CNCC_D5_mem_q10_srt_rmdup_peaks_peak_1460
chr1 100778988 100779265
P19_NANOG_CNCC_D5_mem_q10_srt_rmdup_peaks_peak_1466
chr1 100865919 100866258
P19_NANOG_CNCC_D5_mem_q10_srt_rmdup_peaks_peak_1470
chr1 100889402 100889779
P19_NANOG_CNCC_D5_mem_q10_srt_rmdup_peaks_peak_1471
chr1 101326042 101326338
P19_NANOG_CNCC_D5_mem_q10_srt_rmdup_peaks_peak_1475
chr1 102598362 102598652
P19_NANOG_CNCC_D5_mem_q10_srt_rmdup_peaks_peak_1486
chr1 104430833 104431236
P19_NANOG_CNCC_D5_mem_q10_srt_rmdup_peaks_peak_1490
chr1 104724865 104725222
P19_NANOG_CNCC_D5_mem_q10_srt_rmdup_peaks_peak_1491
chr1 107406856 107407184

P19_NANOG_CNCC_D5_mem_q10_srt_rmdup_peaks_peak_1496
chr1	107682407	107682768
P19_NANOG_CNCC_D5_mem_q10_srt_rmdup_peaks_peak_1497
chr1	107723946	107724306
P19_NANOG_CNCC_D5_mem_q10_srt_rmdup_peaks_peak_1499
chr1	107963320	107963639
P19_NANOG_CNCC_D5_mem_q10_srt_rmdup_peaks_peak_1500
chr1	107975821	107976162
P19_NANOG_CNCC_D5_mem_q10_srt_rmdup_peaks_peak_1502
chr1	108067605	108067943
P19_NANOG_CNCC_D5_mem_q10_srt_rmdup_peaks_peak_1504
chr1	108337368	108337639
P19_NANOG_CNCC_D5_mem_q10_srt_rmdup_peaks_peak_1506
chr1	108507039	108507349
P19_NANOG_CNCC_D5_mem_q10_srt_rmdup_peaks_peak_1510
chr1	108593914	108594300
P19_NANOG_CNCC_D5_mem_q10_srt_rmdup_peaks_peak_1511
chr1	109323589	109324009
P19_NANOG_CNCC_D5_mem_q10_srt_rmdup_peaks_peak_1513
chr1	109925780	109926106
P19_NANOG_CNCC_D5_mem_q10_srt_rmdup_peaks_peak_1521
chr1	110325188	110325566
P19_NANOG_CNCC_D5_mem_q10_srt_rmdup_peaks_peak_1526
chr1	110546437	110546900
P19_NANOG_CNCC_D5_mem_q10_srt_rmdup_peaks_peak_1532
chr1	110691776	110692105
P19_NANOG_CNCC_D5_mem_q10_srt_rmdup_peaks_peak_1535
chr1	110814697	110815151
P19_NANOG_CNCC_D5_mem_q10_srt_rmdup_peaks_peak_1536
chr1	111263785	111264161
P19_NANOG_CNCC_D5_mem_q10_srt_rmdup_peaks_peak_1543
chr1	111294977	111295259
P19_NANOG_CNCC_D5_mem_q10_srt_rmdup_peaks_peak_1544
chr1	111802383	111802677
P19_NANOG_CNCC_D5_mem_q10_srt_rmdup_peaks_peak_1548
chr1	113067690	113068001
P19_NANOG_CNCC_D5_mem_q10_srt_rmdup_peaks_peak_1566
chr1	113452520	113452935
P19_NANOG_CNCC_D5_mem_q10_srt_rmdup_peaks_peak_1576
chr1	114489375	114489734
P19_NANOG_CNCC_D5_mem_q10_srt_rmdup_peaks_peak_1593
chr1	115295839	115296186
P19_NANOG_CNCC_D5_mem_q10_srt_rmdup_peaks_peak_1607
chr1	116069630	116070107
P19_NANOG_CNCC_D5_mem_q10_srt_rmdup_peaks_peak_1616
chr1	116088297	116088653
P19_NANOG_CNCC_D5_mem_q10_srt_rmdup_peaks_peak_1617
chr1	116088816	116089099
P19_NANOG_CNCC_D5_mem_q10_srt_rmdup_peaks_peak_1618
chr1	116100217	116100745

P19_NANOG_CNCC_D5_mem_q10_srt_rmdup_peaks_peak_1619
chr1 116407498 116407857
P19_NANOG_CNCC_D5_mem_q10_srt_rmdup_peaks_peak_1624
chr1 116467344 116467672
P19_NANOG_CNCC_D5_mem_q10_srt_rmdup_peaks_peak_1625
chr1 117403549 117404105
P19_NANOG_CNCC_D5_mem_q10_srt_rmdup_peaks_peak_1639
chr1 117632523 117632990
P19_NANOG_CNCC_D5_mem_q10_srt_rmdup_peaks_peak_1643
chr1 118050223 118050654
P19_NANOG_CNCC_D5_mem_q10_srt_rmdup_peaks_peak_1649
chr1 119025953 119026333
P19_NANOG_CNCC_D5_mem_q10_srt_rmdup_peaks_peak_1657
chr1 119623217 119623553
P19_NANOG_CNCC_D5_mem_q10_srt_rmdup_peaks_peak_1669
chr1 144689655 144689945
P19_NANOG_CNCC_D5_mem_q10_srt_rmdup_peaks_peak_1691
chr1 145382058 145382403
P19_NANOG_CNCC_D5_mem_q10_srt_rmdup_peaks_peak_1700
chr1 145575529 145575905
P19_NANOG_CNCC_D5_mem_q10_srt_rmdup_peaks_peak_1708
chr1 147737199 147737470
P19_NANOG_CNCC_D5_mem_q10_srt_rmdup_peaks_peak_1725
chr1 148660592 148661033
P19_NANOG_CNCC_D5_mem_q10_srt_rmdup_peaks_peak_1729
chr1 149294491 149294774
P19_NANOG_CNCC_D5_mem_q10_srt_rmdup_peaks_peak_1732
chr1 149719257 149719566
P19_NANOG_CNCC_D5_mem_q10_srt_rmdup_peaks_peak_1735
chr1 149747103 149747506
P19_NANOG_CNCC_D5_mem_q10_srt_rmdup_peaks_peak_1737
chr1 149783895 149784166
P19_NANOG_CNCC_D5_mem_q10_srt_rmdup_peaks_peak_1740
chr1 149989407 149989703
P19_NANOG_CNCC_D5_mem_q10_srt_rmdup_peaks_peak_1743
chr1 150884982 150885414
P19_NANOG_CNCC_D5_mem_q10_srt_rmdup_peaks_peak_1769
chr1 151584451 151584880
P19_NANOG_CNCC_D5_mem_q10_srt_rmdup_peaks_peak_1779
chr1 151945551 151945822
P19_NANOG_CNCC_D5_mem_q10_srt_rmdup_peaks_peak_1784
chr1 153490579 153490856
P19_NANOG_CNCC_D5_mem_q10_srt_rmdup_peaks_peak_1793
chr1 154909464 154909735
P19_NANOG_CNCC_D5_mem_q10_srt_rmdup_peaks_peak_1808
chr1 155294820 155295198
P19_NANOG_CNCC_D5_mem_q10_srt_rmdup_peaks_peak_1827
chr1 155568726 155569223
P19_NANOG_CNCC_D5_mem_q10_srt_rmdup_peaks_peak_1828
chr1 155569980 155570351

P19_NANOG_CNCC_D5_mem_q10_srt_rmdup_peaks_peak_1829
chr1 155948277 155948548
P19_NANOG_CNCC_D5_mem_q10_srt_rmdup_peaks_peak_1833
chr1 156470993 156471384
P19_NANOG_CNCC_D5_mem_q10_srt_rmdup_peaks_peak_1854
chr1 156611556 156611915
P19_NANOG_CNCC_D5_mem_q10_srt_rmdup_peaks_peak_1856
chr1 156902970 156903381
P19_NANOG_CNCC_D5_mem_q10_srt_rmdup_peaks_peak_1869
chr1 157166592 157166869
P19_NANOG_CNCC_D5_mem_q10_srt_rmdup_peaks_peak_1876
chr1 157838533 157838858
P19_NANOG_CNCC_D5_mem_q10_srt_rmdup_peaks_peak_1883
chr1 158184718 158185332
P19_NANOG_CNCC_D5_mem_q10_srt_rmdup_peaks_peak_1891
chr1 158975467 158975803
P19_NANOG_CNCC_D5_mem_q10_srt_rmdup_peaks_peak_1896
chr1 159645267 159645628
P19_NANOG_CNCC_D5_mem_q10_srt_rmdup_peaks_peak_1902
chr1 159976176 159976447
P19_NANOG_CNCC_D5_mem_q10_srt_rmdup_peaks_peak_1906
chr1 161015693 161016139
P19_NANOG_CNCC_D5_mem_q10_srt_rmdup_peaks_peak_1923
chr1 161335041 161335415
P19_NANOG_CNCC_D5_mem_q10_srt_rmdup_peaks_peak_1931
chr1 161391630 161391967
P19_NANOG_CNCC_D5_mem_q10_srt_rmdup_peaks_peak_1933
chr1 161886679 161887187
P19_NANOG_CNCC_D5_mem_q10_srt_rmdup_peaks_peak_1939
chr1 162125857 162126215
P19_NANOG_CNCC_D5_mem_q10_srt_rmdup_peaks_peak_1947
chr1 162283910 162284234
P19_NANOG_CNCC_D5_mem_q10_srt_rmdup_peaks_peak_1953
chr1 162370693 162371169
P19_NANOG_CNCC_D5_mem_q10_srt_rmdup_peaks_peak_1955
chr1 163268245 163268673
P19_NANOG_CNCC_D5_mem_q10_srt_rmdup_peaks_peak_1964
chr1 163716384 163716913
P19_NANOG_CNCC_D5_mem_q10_srt_rmdup_peaks_peak_1971
chr1 164004726 164005101
P19_NANOG_CNCC_D5_mem_q10_srt_rmdup_peaks_peak_1975
chr1 164023556 164023964
P19_NANOG_CNCC_D5_mem_q10_srt_rmdup_peaks_peak_1976
chr1 164527617 164528357
P19_NANOG_CNCC_D5_mem_q10_srt_rmdup_peaks_peak_1979
chr1 164607710 164608055
P19_NANOG_CNCC_D5_mem_q10_srt_rmdup_peaks_peak_1989
chr1 164652442 164652752
P19_NANOG_CNCC_D5_mem_q10_srt_rmdup_peaks_peak_1990
chr1 165042504 165042882

P19_NANOG_CNCC_D5_mem_q10_srt_rmdup_peaks_peak_1998
chr1 165098739 165099056
P19_NANOG_CNCC_D5_mem_q10_srt_rmdup_peaks_peak_1999
chr1 165530194 165530650
P19_NANOG_CNCC_D5_mem_q10_srt_rmdup_peaks_peak_2008
chr1 165555682 165556004
P19_NANOG_CNCC_D5_mem_q10_srt_rmdup_peaks_peak_2009
chr1 166305640 166305975
P19_NANOG_CNCC_D5_mem_q10_srt_rmdup_peaks_peak_2016
chr1 166686970 166687260
P19_NANOG_CNCC_D5_mem_q10_srt_rmdup_peaks_peak_2019
chr1 167436616 167436963
P19_NANOG_CNCC_D5_mem_q10_srt_rmdup_peaks_peak_2034
chr1 167604411 167604834
P19_NANOG_CNCC_D5_mem_q10_srt_rmdup_peaks_peak_2037
chr1 167684507 167685105
P19_NANOG_CNCC_D5_mem_q10_srt_rmdup_peaks_peak_2039
chr1 168240924 168241217
P19_NANOG_CNCC_D5_mem_q10_srt_rmdup_peaks_peak_2044
chr1 168409106 168409379
P19_NANOG_CNCC_D5_mem_q10_srt_rmdup_peaks_peak_2046
chr1 170862879 170863452
P19_NANOG_CNCC_D5_mem_q10_srt_rmdup_peaks_peak_2067
chr1 172386769 172387090
P19_NANOG_CNCC_D5_mem_q10_srt_rmdup_peaks_peak_2081
chr1 172387208 172387602
P19_NANOG_CNCC_D5_mem_q10_srt_rmdup_peaks_peak_2082
chr1 173417603 173418054
P19_NANOG_CNCC_D5_mem_q10_srt_rmdup_peaks_peak_2089
chr1 173822002 173822580
P19_NANOG_CNCC_D5_mem_q10_srt_rmdup_peaks_peak_2093
chr1 173903504 173903961
P19_NANOG_CNCC_D5_mem_q10_srt_rmdup_peaks_peak_2095
chr1 174992453 174992737
P19_NANOG_CNCC_D5_mem_q10_srt_rmdup_peaks_peak_2103
chr1 175307379 175307763
P19_NANOG_CNCC_D5_mem_q10_srt_rmdup_peaks_peak_2107
chr1 175803718 175804173
P19_NANOG_CNCC_D5_mem_q10_srt_rmdup_peaks_peak_2116
chr1 176027978 176028392
P19_NANOG_CNCC_D5_mem_q10_srt_rmdup_peaks_peak_2120
chr1 176196744 176197027
P19_NANOG_CNCC_D5_mem_q10_srt_rmdup_peaks_peak_2123
chr1 176652204 176652629
P19_NANOG_CNCC_D5_mem_q10_srt_rmdup_peaks_peak_2126
chr1 176702698 176703087
P19_NANOG_CNCC_D5_mem_q10_srt_rmdup_peaks_peak_2127
chr1 176911617 176911932
P19_NANOG_CNCC_D5_mem_q10_srt_rmdup_peaks_peak_2128
chr1 178252335 178252873

P19_NANOG_CNCC_D5_mem_q10_srt_rmdup_peaks_peak_2143
chr1 178314114 178314422
P19_NANOG_CNCC_D5_mem_q10_srt_rmdup_peaks_peak_2144
chr1 178512112 178512416
P19_NANOG_CNCC_D5_mem_q10_srt_rmdup_peaks_peak_2145
chr1 178994992 178995669
P19_NANOG_CNCC_D5_mem_q10_srt_rmdup_peaks_peak_2151
chr1 179110746 179111246
P19_NANOG_CNCC_D5_mem_q10_srt_rmdup_peaks_peak_2152
chr1 180123779 180124067
P19_NANOG_CNCC_D5_mem_q10_srt_rmdup_peaks_peak_2159
chr1 181069142 181069750
P19_NANOG_CNCC_D5_mem_q10_srt_rmdup_peaks_peak_2178
chr1 182571302 182571644
P19_NANOG_CNCC_D5_mem_q10_srt_rmdup_peaks_peak_2200
chr1 182573545 182573897
P19_NANOG_CNCC_D5_mem_q10_srt_rmdup_peaks_peak_2201
chr1 183263187 183263467
P19_NANOG_CNCC_D5_mem_q10_srt_rmdup_peaks_peak_2212
chr1 183318328 183318752
P19_NANOG_CNCC_D5_mem_q10_srt_rmdup_peaks_peak_2217
chr1 184395507 184395890
P19_NANOG_CNCC_D5_mem_q10_srt_rmdup_peaks_peak_2234
chr1 184665279 184665974
P19_NANOG_CNCC_D5_mem_q10_srt_rmdup_peaks_peak_2241
chr1 185448608 185449438
P19_NANOG_CNCC_D5_mem_q10_srt_rmdup_peaks_peak_2249
chr1 187574927 187575240
P19_NANOG_CNCC_D5_mem_q10_srt_rmdup_peaks_peak_2264
chr1 189868606 189869426
P19_NANOG_CNCC_D5_mem_q10_srt_rmdup_peaks_peak_2272
chr1 189890786 189891111
P19_NANOG_CNCC_D5_mem_q10_srt_rmdup_peaks_peak_2273
chr1 191143187 191143597
P19_NANOG_CNCC_D5_mem_q10_srt_rmdup_peaks_peak_2278
chr1 191350194 191350725
P19_NANOG_CNCC_D5_mem_q10_srt_rmdup_peaks_peak_2279
chr1 191741232 191741583
P19_NANOG_CNCC_D5_mem_q10_srt_rmdup_peaks_peak_2281
chr1 192602442 192602730
P19_NANOG_CNCC_D5_mem_q10_srt_rmdup_peaks_peak_2282
chr1 193649287 193649683
P19_NANOG_CNCC_D5_mem_q10_srt_rmdup_peaks_peak_2291
chr1 194988571 194988920
P19_NANOG_CNCC_D5_mem_q10_srt_rmdup_peaks_peak_2294
chr1 195730306 195730588
P19_NANOG_CNCC_D5_mem_q10_srt_rmdup_peaks_peak_2296
chr1 196306380 196306987
P19_NANOG_CNCC_D5_mem_q10_srt_rmdup_peaks_peak_2305
chr1 196546471 196546924

P19_NANOG_CNCC_D5_mem_q10_srt_rmdup_peaks_peak_2310
chr1 198392757 198393312
P19_NANOG_CNCC_D5_mem_q10_srt_rmdup_peaks_peak_2332
chr1 199481166 199481569
P19_NANOG_CNCC_D5_mem_q10_srt_rmdup_peaks_peak_2339
chr1 199660613 199661037
P19_NANOG_CNCC_D5_mem_q10_srt_rmdup_peaks_peak_2341
chr1 200193485 200193836
P19_NANOG_CNCC_D5_mem_q10_srt_rmdup_peaks_peak_2351
chr1 200334740 200335284
P19_NANOG_CNCC_D5_mem_q10_srt_rmdup_peaks_peak_2355
chr1 201417334 201417630
P19_NANOG_CNCC_D5_mem_q10_srt_rmdup_peaks_peak_2367
chr1 201619403 201619674
P19_NANOG_CNCC_D5_mem_q10_srt_rmdup_peaks_peak_2375
chr1 201708823 201709096
P19_NANOG_CNCC_D5_mem_q10_srt_rmdup_peaks_peak_2378
chr1 201711287 201711619
P19_NANOG_CNCC_D5_mem_q10_srt_rmdup_peaks_peak_2379
chr1 201721058 201721741
P19_NANOG_CNCC_D5_mem_q10_srt_rmdup_peaks_peak_2380
chr1 202607078 202607349
P19_NANOG_CNCC_D5_mem_q10_srt_rmdup_peaks_peak_2401
chr1 202662027 202662314
P19_NANOG_CNCC_D5_mem_q10_srt_rmdup_peaks_peak_2403
chr1 202682334 202682629
P19_NANOG_CNCC_D5_mem_q10_srt_rmdup_peaks_peak_2404
chr1 203023823 203024217
P19_NANOG_CNCC_D5_mem_q10_srt_rmdup_peaks_peak_2408
chr1 203189227 203189507
P19_NANOG_CNCC_D5_mem_q10_srt_rmdup_peaks_peak_2414
chr1 204406978 204407369
P19_NANOG_CNCC_D5_mem_q10_srt_rmdup_peaks_peak_2444
chr1 204616678 204616949
P19_NANOG_CNCC_D5_mem_q10_srt_rmdup_peaks_peak_2450
chr1 205425999 205426678
P19_NANOG_CNCC_D5_mem_q10_srt_rmdup_peaks_peak_2478
chr1 205457186 205457473
P19_NANOG_CNCC_D5_mem_q10_srt_rmdup_peaks_peak_2479
chr1 205630839 205631135
P19_NANOG_CNCC_D5_mem_q10_srt_rmdup_peaks_peak_2481
chr1 206223412 206223683
P19_NANOG_CNCC_D5_mem_q10_srt_rmdup_peaks_peak_2486
chr1 206819765 206820073
P19_NANOG_CNCC_D5_mem_q10_srt_rmdup_peaks_peak_2488
chr1 207773755 207774228
P19_NANOG_CNCC_D5_mem_q10_srt_rmdup_peaks_peak_2498
chr1 207811899 207812566
P19_NANOG_CNCC_D5_mem_q10_srt_rmdup_peaks_peak_2501
chr1 207822585 207823256

P19_NANOG_CNCC_D5_mem_q10_srt_rmdup_peaks_peak_2502
chr1 208371531 208371856
P19_NANOG_CNCC_D5_mem_q10_srt_rmdup_peaks_peak_2508
chr1 208523029 208523479
P19_NANOG_CNCC_D5_mem_q10_srt_rmdup_peaks_peak_2513
chr1 209066689 209067209
P19_NANOG_CNCC_D5_mem_q10_srt_rmdup_peaks_peak_2519
chr1 209132074 209132345
P19_NANOG_CNCC_D5_mem_q10_srt_rmdup_peaks_peak_2520
chr1 209253790 209254260
P19_NANOG_CNCC_D5_mem_q10_srt_rmdup_peaks_peak_2521
chr1 209557404 209557814
P19_NANOG_CNCC_D5_mem_q10_srt_rmdup_peaks_peak_2526
chr1 209722713 209723216
P19_NANOG_CNCC_D5_mem_q10_srt_rmdup_peaks_peak_2531
chr1 209826432 209826720
P19_NANOG_CNCC_D5_mem_q10_srt_rmdup_peaks_peak_2535
chr1 210794293 210794713
P19_NANOG_CNCC_D5_mem_q10_srt_rmdup_peaks_peak_2549
chr1 210930975 210931406
P19_NANOG_CNCC_D5_mem_q10_srt_rmdup_peaks_peak_2552
chr1 211091039 211091429
P19_NANOG_CNCC_D5_mem_q10_srt_rmdup_peaks_peak_2553
chr1 211696067 211696361
P19_NANOG_CNCC_D5_mem_q10_srt_rmdup_peaks_peak_2561
chr1 211754407 211754933
P19_NANOG_CNCC_D5_mem_q10_srt_rmdup_peaks_peak_2562
chr1 211803399 211803768
P19_NANOG_CNCC_D5_mem_q10_srt_rmdup_peaks_peak_2564
chr1 211877548 211877913
P19_NANOG_CNCC_D5_mem_q10_srt_rmdup_peaks_peak_2565
chr1 212680067 212680378
P19_NANOG_CNCC_D5_mem_q10_srt_rmdup_peaks_peak_2577
chr1 212788536 212788812
P19_NANOG_CNCC_D5_mem_q10_srt_rmdup_peaks_peak_2581
chr1 212829314 212829709
P19_NANOG_CNCC_D5_mem_q10_srt_rmdup_peaks_peak_2582
chr1 213831607 213831973
P19_NANOG_CNCC_D5_mem_q10_srt_rmdup_peaks_peak_2598
chr1 213867284 213867594
P19_NANOG_CNCC_D5_mem_q10_srt_rmdup_peaks_peak_2600
chr1 214366281 214366558
P19_NANOG_CNCC_D5_mem_q10_srt_rmdup_peaks_peak_2610
chr1 214553693 214554135
P19_NANOG_CNCC_D5_mem_q10_srt_rmdup_peaks_peak_2617
chr1 214649613 214649941
P19_NANOG_CNCC_D5_mem_q10_srt_rmdup_peaks_peak_2623
chr1 215740327 215740598
P19_NANOG_CNCC_D5_mem_q10_srt_rmdup_peaks_peak_2629
chr1 215756543 215757014

P19_NANOG_CNCC_D5_mem_q10_srt_rmdup_peaks_peak_2630
chr1	216955689	216956139
P19_NANOG_CNCC_D5_mem_q10_srt_rmdup_peaks_peak_2643
chr1	217029044	217029411
P19_NANOG_CNCC_D5_mem_q10_srt_rmdup_peaks_peak_2644
chr1	218633341	218633623
P19_NANOG_CNCC_D5_mem_q10_srt_rmdup_peaks_peak_2663
chr1	220011131	220011448
P19_NANOG_CNCC_D5_mem_q10_srt_rmdup_peaks_peak_2678
chr1	220959900	220960406
P19_NANOG_CNCC_D5_mem_q10_srt_rmdup_peaks_peak_2689
chr1	224026901	224027191
P19_NANOG_CNCC_D5_mem_q10_srt_rmdup_peaks_peak_2728
chr1	225898627	225898900
P19_NANOG_CNCC_D5_mem_q10_srt_rmdup_peaks_peak_2751
chr1	226038053	226038401
P19_NANOG_CNCC_D5_mem_q10_srt_rmdup_peaks_peak_2755
chr1	226891051	226891355
P19_NANOG_CNCC_D5_mem_q10_srt_rmdup_peaks_peak_2773
chr1	227038799	227039172
P19_NANOG_CNCC_D5_mem_q10_srt_rmdup_peaks_peak_2779
chr1	227127796	227128148
P19_NANOG_CNCC_D5_mem_q10_srt_rmdup_peaks_peak_2781
chr1	227259964	227260328
P19_NANOG_CNCC_D5_mem_q10_srt_rmdup_peaks_peak_2783
chr1	227774337	227774791
P19_NANOG_CNCC_D5_mem_q10_srt_rmdup_peaks_peak_2785
chr1	228196828	228197269
P19_NANOG_CNCC_D5_mem_q10_srt_rmdup_peaks_peak_2788
chr1	229478170	229478441
P19_NANOG_CNCC_D5_mem_q10_srt_rmdup_peaks_peak_2818
chr1	229698312	229699687
P19_NANOG_CNCC_D5_mem_q10_srt_rmdup_peaks_peak_2823
chr1	229759522	229759906
P19_NANOG_CNCC_D5_mem_q10_srt_rmdup_peaks_peak_2824
chr1	230154036	230154416
P19_NANOG_CNCC_D5_mem_q10_srt_rmdup_peaks_peak_2827
chr1	231181042	231181484
P19_NANOG_CNCC_D5_mem_q10_srt_rmdup_peaks_peak_2845
chr1	233248013	233248334
P19_NANOG_CNCC_D5_mem_q10_srt_rmdup_peaks_peak_2871
chr1	233430776	233431185
P19_NANOG_CNCC_D5_mem_q10_srt_rmdup_peaks_peak_2873
chr1	233813393	233813722
P19_NANOG_CNCC_D5_mem_q10_srt_rmdup_peaks_peak_2880
chr1	233815113	233815444
P19_NANOG_CNCC_D5_mem_q10_srt_rmdup_peaks_peak_2881
chr1	233861200	233861580
P19_NANOG_CNCC_D5_mem_q10_srt_rmdup_peaks_peak_2883
chr1	234881611	234881932

P19_NANOG_CNCC_D5_mem_q10_srt_rmdup_peaks_peak_2892
chr1 235433943 235434230
P19_NANOG_CNCC_D5_mem_q10_srt_rmdup_peaks_peak_2903
chr1 236687661 236687953
P19_NANOG_CNCC_D5_mem_q10_srt_rmdup_peaks_peak_2918
chr1 236958357 236958728
P19_NANOG_CNCC_D5_mem_q10_srt_rmdup_peaks_peak_2919
chr1 237278547 237278964
P19_NANOG_CNCC_D5_mem_q10_srt_rmdup_peaks_peak_2927
chr1 237402534 237402948
P19_NANOG_CNCC_D5_mem_q10_srt_rmdup_peaks_peak_2928
chr1 237410746 237411066
P19_NANOG_CNCC_D5_mem_q10_srt_rmdup_peaks_peak_2929
chr1 237899817 237900144
P19_NANOG_CNCC_D5_mem_q10_srt_rmdup_peaks_peak_2932
chr1 238059935 238060298
P19_NANOG_CNCC_D5_mem_q10_srt_rmdup_peaks_peak_2936
chr1 239289291 239289562
P19_NANOG_CNCC_D5_mem_q10_srt_rmdup_peaks_peak_2947
chr1 239550235 239550526
P19_NANOG_CNCC_D5_mem_q10_srt_rmdup_peaks_peak_2949
chr1 239796822 239797121
P19_NANOG_CNCC_D5_mem_q10_srt_rmdup_peaks_peak_2952
chr1 239846148 239846519
P19_NANOG_CNCC_D5_mem_q10_srt_rmdup_peaks_peak_2953
chr1 239918217 239918507
P19_NANOG_CNCC_D5_mem_q10_srt_rmdup_peaks_peak_2954
chr1 239997032 239997303
P19_NANOG_CNCC_D5_mem_q10_srt_rmdup_peaks_peak_2958
chr1 240654511 240654814
P19_NANOG_CNCC_D5_mem_q10_srt_rmdup_peaks_peak_2962
chr1 241119260 241119752
P19_NANOG_CNCC_D5_mem_q10_srt_rmdup_peaks_peak_2964
chr1 241486799 241487075
P19_NANOG_CNCC_D5_mem_q10_srt_rmdup_peaks_peak_2973
chr1 242011055 242011354
P19_NANOG_CNCC_D5_mem_q10_srt_rmdup_peaks_peak_2977
chr1 242379634 242380102
P19_NANOG_CNCC_D5_mem_q10_srt_rmdup_peaks_peak_2983
chr1 243552585 243552858
P19_NANOG_CNCC_D5_mem_q10_srt_rmdup_peaks_peak_2994
chr1 244080552 244080846
P19_NANOG_CNCC_D5_mem_q10_srt_rmdup_peaks_peak_3004
chr1 244090284 244090581
P19_NANOG_CNCC_D5_mem_q10_srt_rmdup_peaks_peak_3005
chr1 244515744 244516015
P19_NANOG_CNCC_D5_mem_q10_srt_rmdup_peaks_peak_3015
chr1 244659556 244659827
P19_NANOG_CNCC_D5_mem_q10_srt_rmdup_peaks_peak_3018
chr1 245938488 245939130

P19_NANOG_CNCC_D5_mem_q10_srt_rmdup_peaks_peak_3038
chr1 246001143 246001444
P19_NANOG_CNCC_D5_mem_q10_srt_rmdup_peaks_peak_3040
chr10 315417 315706
P19_NANOG_CNCC_D5_mem_q10_srt_rmdup_peaks_peak_3059
chr10 1466979 1467334
P19_NANOG_CNCC_D5_mem_q10_srt_rmdup_peaks_peak_3069
chr10 1827959 1828254
P19_NANOG_CNCC_D5_mem_q10_srt_rmdup_peaks_peak_3071
chr10 3375313 3375672
P19_NANOG_CNCC_D5_mem_q10_srt_rmdup_peaks_peak_3084
chr10 3513746 3514024
P19_NANOG_CNCC_D5_mem_q10_srt_rmdup_peaks_peak_3086
chr10 4912946 4913301
P19_NANOG_CNCC_D5_mem_q10_srt_rmdup_peaks_peak_3100
chr10 5790411 5790709
P19_NANOG_CNCC_D5_mem_q10_srt_rmdup_peaks_peak_3105
chr10 6143332 6143608
P19_NANOG_CNCC_D5_mem_q10_srt_rmdup_peaks_peak_3109
chr10 6241746 6242179
P19_NANOG_CNCC_D5_mem_q10_srt_rmdup_peaks_peak_3112
chr10 6345830 6346104
P19_NANOG_CNCC_D5_mem_q10_srt_rmdup_peaks_peak_3113
chr10 6871337 6871663
P19_NANOG_CNCC_D5_mem_q10_srt_rmdup_peaks_peak_3119
chr10 9604043 9604318
P19_NANOG_CNCC_D5_mem_q10_srt_rmdup_peaks_peak_3150
chr10 11027253 11027690
P19_NANOG_CNCC_D5_mem_q10_srt_rmdup_peaks_peak_3159
chr10 11206896 11207167
P19_NANOG_CNCC_D5_mem_q10_srt_rmdup_peaks_peak_3167
chr10 11246458 11247434
P19_NANOG_CNCC_D5_mem_q10_srt_rmdup_peaks_peak_3169
chr10 12838452 12838772
P19_NANOG_CNCC_D5_mem_q10_srt_rmdup_peaks_peak_3182
chr10 13383264 13383765
P19_NANOG_CNCC_D5_mem_q10_srt_rmdup_peaks_peak_3186
chr10 15126284 15126707
P19_NANOG_CNCC_D5_mem_q10_srt_rmdup_peaks_peak_3217
chr10 16992505 16992857
P19_NANOG_CNCC_D5_mem_q10_srt_rmdup_peaks_peak_3234
chr10 17213293 17213644
P19_NANOG_CNCC_D5_mem_q10_srt_rmdup_peaks_peak_3238
chr10 18294312 18294607
P19_NANOG_CNCC_D5_mem_q10_srt_rmdup_peaks_peak_3247
chr10 18639367 18639664
P19_NANOG_CNCC_D5_mem_q10_srt_rmdup_peaks_peak_3253
chr10 18955808 18956262
P19_NANOG_CNCC_D5_mem_q10_srt_rmdup_peaks_peak_3256
chr10 19093940 19094273

P19_NANOG_CNCC_D5_mem_q10_srt_rmdup_peaks_peak_3257
chr10 20122087 20122439
P19_NANOG_CNCC_D5_mem_q10_srt_rmdup_peaks_peak_3262
chr10 20275354 20275679
P19_NANOG_CNCC_D5_mem_q10_srt_rmdup_peaks_peak_3264
chr10 20565054 20565386
P19_NANOG_CNCC_D5_mem_q10_srt_rmdup_peaks_peak_3271
chr10 21404315 21404783
P19_NANOG_CNCC_D5_mem_q10_srt_rmdup_peaks_peak_3280
chr10 21807525 21807971
P19_NANOG_CNCC_D5_mem_q10_srt_rmdup_peaks_peak_3298
chr10 22292585 22293026
P19_NANOG_CNCC_D5_mem_q10_srt_rmdup_peaks_peak_3309
chr10 22633692 22633966
P19_NANOG_CNCC_D5_mem_q10_srt_rmdup_peaks_peak_3323
chr10 22885115 22885409
P19_NANOG_CNCC_D5_mem_q10_srt_rmdup_peaks_peak_3326
chr10 24342922 24343270
P19_NANOG_CNCC_D5_mem_q10_srt_rmdup_peaks_peak_3355
chr10 25369861 25370207
P19_NANOG_CNCC_D5_mem_q10_srt_rmdup_peaks_peak_3372
chr10 25394260 25394649
P19_NANOG_CNCC_D5_mem_q10_srt_rmdup_peaks_peak_3373
chr10 25730669 25731009
P19_NANOG_CNCC_D5_mem_q10_srt_rmdup_peaks_peak_3376
chr10 25780986 25781295
P19_NANOG_CNCC_D5_mem_q10_srt_rmdup_peaks_peak_3377
chr10 25788463 25788832
P19_NANOG_CNCC_D5_mem_q10_srt_rmdup_peaks_peak_3379
chr10 26806582 26806864
P19_NANOG_CNCC_D5_mem_q10_srt_rmdup_peaks_peak_3388
chr10 26811974 26812565
P19_NANOG_CNCC_D5_mem_q10_srt_rmdup_peaks_peak_3389
chr10 27547752 27548023
P19_NANOG_CNCC_D5_mem_q10_srt_rmdup_peaks_peak_3396
chr10 28957681 28957966
P19_NANOG_CNCC_D5_mem_q10_srt_rmdup_peaks_peak_3415
chr10 29011328 29011634
P19_NANOG_CNCC_D5_mem_q10_srt_rmdup_peaks_peak_3419
chr10 29493135 29493560
P19_NANOG_CNCC_D5_mem_q10_srt_rmdup_peaks_peak_3426
chr10 29830778 29831090
P19_NANOG_CNCC_D5_mem_q10_srt_rmdup_peaks_peak_3432
chr10 29844818 29845149
P19_NANOG_CNCC_D5_mem_q10_srt_rmdup_peaks_peak_3433
chr10 29845260 29845831
P19_NANOG_CNCC_D5_mem_q10_srt_rmdup_peaks_peak_3434
chr10 30226484 30226886
P19_NANOG_CNCC_D5_mem_q10_srt_rmdup_peaks_peak_3443
chr10 30994503 30994894

P19_NANOG_CNCC_D5_mem_q10_srt_rmdup_peaks_peak_3454
chr10 31100387 31100736
P19_NANOG_CNCC_D5_mem_q10_srt_rmdup_peaks_peak_3456
chr10 31475463 31475866
P19_NANOG_CNCC_D5_mem_q10_srt_rmdup_peaks_peak_3465
chr10 32266771 32267047
P19_NANOG_CNCC_D5_mem_q10_srt_rmdup_peaks_peak_3473
chr10 32636132 32636447
P19_NANOG_CNCC_D5_mem_q10_srt_rmdup_peaks_peak_3477
chr10 32969964 32970537
P19_NANOG_CNCC_D5_mem_q10_srt_rmdup_peaks_peak_3479
chr10 33167480 33167869
P19_NANOG_CNCC_D5_mem_q10_srt_rmdup_peaks_peak_3480
chr10 34577612 34577887
P19_NANOG_CNCC_D5_mem_q10_srt_rmdup_peaks_peak_3500
chr10 35329789 35330082
P19_NANOG_CNCC_D5_mem_q10_srt_rmdup_peaks_peak_3510
chr10 35683075 35683606
P19_NANOG_CNCC_D5_mem_q10_srt_rmdup_peaks_peak_3512
chr10 35946511 35947039
P19_NANOG_CNCC_D5_mem_q10_srt_rmdup_peaks_peak_3524
chr10 43904190 43904527
P19_NANOG_CNCC_D5_mem_q10_srt_rmdup_peaks_peak_3556
chr10 44783323 44783801
P19_NANOG_CNCC_D5_mem_q10_srt_rmdup_peaks_peak_3564
chr10 45374375 45374660
P19_NANOG_CNCC_D5_mem_q10_srt_rmdup_peaks_peak_3572
chr10 45737628 45738006
P19_NANOG_CNCC_D5_mem_q10_srt_rmdup_peaks_peak_3579
chr10 47599877 47600153
P19_NANOG_CNCC_D5_mem_q10_srt_rmdup_peaks_peak_3588
chr10 52269335 52269668
P19_NANOG_CNCC_D5_mem_q10_srt_rmdup_peaks_peak_3617
chr10 53252201 53252472
P19_NANOG_CNCC_D5_mem_q10_srt_rmdup_peaks_peak_3631
chr10 53350512 53351134
P19_NANOG_CNCC_D5_mem_q10_srt_rmdup_peaks_peak_3633
chr10 53852202 53852473
P19_NANOG_CNCC_D5_mem_q10_srt_rmdup_peaks_peak_3644
chr10 54577811 54578147
P19_NANOG_CNCC_D5_mem_q10_srt_rmdup_peaks_peak_3654
chr10 54644133 54644461
P19_NANOG_CNCC_D5_mem_q10_srt_rmdup_peaks_peak_3655
chr10 54709647 54710105
P19_NANOG_CNCC_D5_mem_q10_srt_rmdup_peaks_peak_3659
chr10 54719497 54720051
P19_NANOG_CNCC_D5_mem_q10_srt_rmdup_peaks_peak_3660
chr10 55016404 55016675
P19_NANOG_CNCC_D5_mem_q10_srt_rmdup_peaks_peak_3664
chr10 55252672 55252945

P19_NANOG_CNCC_D5_mem_q10_srt_rmdup_peaks_peak_3671
chr10 57018693 57019144
P19_NANOG_CNCC_D5_mem_q10_srt_rmdup_peaks_peak_3678
chr10 57906498 57906930
P19_NANOG_CNCC_D5_mem_q10_srt_rmdup_peaks_peak_3685
chr10 58346190 58346819
P19_NANOG_CNCC_D5_mem_q10_srt_rmdup_peaks_peak_3687
chr10 60024534 60024805
P19_NANOG_CNCC_D5_mem_q10_srt_rmdup_peaks_peak_3698
chr10 60172390 60172789
P19_NANOG_CNCC_D5_mem_q10_srt_rmdup_peaks_peak_3700
chr10 60237004 60237362
P19_NANOG_CNCC_D5_mem_q10_srt_rmdup_peaks_peak_3701
chr10 61981581 61981916
P19_NANOG_CNCC_D5_mem_q10_srt_rmdup_peaks_peak_3723
chr10 63605431 63605707
P19_NANOG_CNCC_D5_mem_q10_srt_rmdup_peaks_peak_3742
chr10 63663202 63663473
P19_NANOG_CNCC_D5_mem_q10_srt_rmdup_peaks_peak_3743
chr10 64186473 64186767
P19_NANOG_CNCC_D5_mem_q10_srt_rmdup_peaks_peak_3747
chr10 64273960 64274282
P19_NANOG_CNCC_D5_mem_q10_srt_rmdup_peaks_peak_3749
chr10 64892702 64893118
P19_NANOG_CNCC_D5_mem_q10_srt_rmdup_peaks_peak_3755
chr10 65263922 65264342
P19_NANOG_CNCC_D5_mem_q10_srt_rmdup_peaks_peak_3763
chr10 65558286 65558674
P19_NANOG_CNCC_D5_mem_q10_srt_rmdup_peaks_peak_3767
chr10 66311543 66311892
P19_NANOG_CNCC_D5_mem_q10_srt_rmdup_peaks_peak_3777
chr10 67076166 67076522
P19_NANOG_CNCC_D5_mem_q10_srt_rmdup_peaks_peak_3780
chr10 68019742 68020138
P19_NANOG_CNCC_D5_mem_q10_srt_rmdup_peaks_peak_3783
chr10 68858213 68858588
P19_NANOG_CNCC_D5_mem_q10_srt_rmdup_peaks_peak_3788
chr10 69043885 69044270
P19_NANOG_CNCC_D5_mem_q10_srt_rmdup_peaks_peak_3789
chr10 69388449 69388720
P19_NANOG_CNCC_D5_mem_q10_srt_rmdup_peaks_peak_3793
chr10 69389377 69389654
P19_NANOG_CNCC_D5_mem_q10_srt_rmdup_peaks_peak_3794
chr10 70364183 70364547
P19_NANOG_CNCC_D5_mem_q10_srt_rmdup_peaks_peak_3811
chr10 71026901 71027269
P19_NANOG_CNCC_D5_mem_q10_srt_rmdup_peaks_peak_3819
chr10 71105808 71106177
P19_NANOG_CNCC_D5_mem_q10_srt_rmdup_peaks_peak_3821
chr10 71435546 71436004

P19_NANOG_CNCC_D5_mem_q10_srt_rmdup_peaks_peak_3829
chr10 71659292 71659615
P19_NANOG_CNCC_D5_mem_q10_srt_rmdup_peaks_peak_3833
chr10 72639847 72640186
P19_NANOG_CNCC_D5_mem_q10_srt_rmdup_peaks_peak_3849
chr10 72936485 72936765
P19_NANOG_CNCC_D5_mem_q10_srt_rmdup_peaks_peak_3854
chr10 73078733 73079119
P19_NANOG_CNCC_D5_mem_q10_srt_rmdup_peaks_peak_3859
chr10 73328076 73328397
P19_NANOG_CNCC_D5_mem_q10_srt_rmdup_peaks_peak_3865
chr10 73402613 73402982
P19_NANOG_CNCC_D5_mem_q10_srt_rmdup_peaks_peak_3869
chr10 73599790 73600244
P19_NANOG_CNCC_D5_mem_q10_srt_rmdup_peaks_peak_3872
chr10 74155366 74155667
P19_NANOG_CNCC_D5_mem_q10_srt_rmdup_peaks_peak_3884
chr10 75351045 75351516
P19_NANOG_CNCC_D5_mem_q10_srt_rmdup_peaks_peak_3894
chr10 76178230 76178596
P19_NANOG_CNCC_D5_mem_q10_srt_rmdup_peaks_peak_3903
chr10 76179134 76179546
P19_NANOG_CNCC_D5_mem_q10_srt_rmdup_peaks_peak_3904
chr10 76489980 76490300
P19_NANOG_CNCC_D5_mem_q10_srt_rmdup_peaks_peak_3907
chr10 76677179 76677485
P19_NANOG_CNCC_D5_mem_q10_srt_rmdup_peaks_peak_3912
chr10 77727231 77727623
P19_NANOG_CNCC_D5_mem_q10_srt_rmdup_peaks_peak_3936
chr10 77955541 77955831
P19_NANOG_CNCC_D5_mem_q10_srt_rmdup_peaks_peak_3941
chr10 78409040 78409314
P19_NANOG_CNCC_D5_mem_q10_srt_rmdup_peaks_peak_3946
chr10 79388809 79389163
P19_NANOG_CNCC_D5_mem_q10_srt_rmdup_peaks_peak_3955
chr10 79708884 79709283
P19_NANOG_CNCC_D5_mem_q10_srt_rmdup_peaks_peak_3967
chr10 79793039 79793468
P19_NANOG_CNCC_D5_mem_q10_srt_rmdup_peaks_peak_3969
chr10 80831292 80831582
P19_NANOG_CNCC_D5_mem_q10_srt_rmdup_peaks_peak_3989
chr10 82359311 82359595
P19_NANOG_CNCC_D5_mem_q10_srt_rmdup_peaks_peak_4006
chr10 83641718 83642084
P19_NANOG_CNCC_D5_mem_q10_srt_rmdup_peaks_peak_4013
chr10 83677335 83677801
P19_NANOG_CNCC_D5_mem_q10_srt_rmdup_peaks_peak_4015
chr10 84573598 84574001
P19_NANOG_CNCC_D5_mem_q10_srt_rmdup_peaks_peak_4023
chr10 85920822 85921100

P19_NANOG_CNCC_D5_mem_q10_srt_rmdup_peaks_peak_4036
chr10 85954158 85954455
P19_NANOG_CNCC_D5_mem_q10_srt_rmdup_peaks_peak_4037
chr10 86073628 86073899
P19_NANOG_CNCC_D5_mem_q10_srt_rmdup_peaks_peak_4039
chr10 86816696 86817083
P19_NANOG_CNCC_D5_mem_q10_srt_rmdup_peaks_peak_4048
chr10 86910041 86910350
P19_NANOG_CNCC_D5_mem_q10_srt_rmdup_peaks_peak_4049
chr10 87806639 87807077
P19_NANOG_CNCC_D5_mem_q10_srt_rmdup_peaks_peak_4057
chr10 88345058 88345416
P19_NANOG_CNCC_D5_mem_q10_srt_rmdup_peaks_peak_4068
chr10 88583187 88583609
P19_NANOG_CNCC_D5_mem_q10_srt_rmdup_peaks_peak_4069
chr10 90175580 90175985
P19_NANOG_CNCC_D5_mem_q10_srt_rmdup_peaks_peak_4094
chr10 91145967 91146262
P19_NANOG_CNCC_D5_mem_q10_srt_rmdup_peaks_peak_4103
chr10 91455368 91455942
P19_NANOG_CNCC_D5_mem_q10_srt_rmdup_peaks_peak_4108
chr10 91461118 91461410
P19_NANOG_CNCC_D5_mem_q10_srt_rmdup_peaks_peak_4109
chr10 92535146 92535450
P19_NANOG_CNCC_D5_mem_q10_srt_rmdup_peaks_peak_4120
chr10 93672578 93672849
P19_NANOG_CNCC_D5_mem_q10_srt_rmdup_peaks_peak_4136
chr10 93713445 93713783
P19_NANOG_CNCC_D5_mem_q10_srt_rmdup_peaks_peak_4137
chr10 94114013 94114561
P19_NANOG_CNCC_D5_mem_q10_srt_rmdup_peaks_peak_4145
chr10 94594777 94595108
P19_NANOG_CNCC_D5_mem_q10_srt_rmdup_peaks_peak_4154
chr10 94652180 94652550
P19_NANOG_CNCC_D5_mem_q10_srt_rmdup_peaks_peak_4156
chr10 95608247 95608741
P19_NANOG_CNCC_D5_mem_q10_srt_rmdup_peaks_peak_4186
chr10 96122613 96122888
P19_NANOG_CNCC_D5_mem_q10_srt_rmdup_peaks_peak_4192
chr10 97092776 97093116
P19_NANOG_CNCC_D5_mem_q10_srt_rmdup_peaks_peak_4207
chr10 97311305 97311654
P19_NANOG_CNCC_D5_mem_q10_srt_rmdup_peaks_peak_4211
chr10 97982006 97982739
P19_NANOG_CNCC_D5_mem_q10_srt_rmdup_peaks_peak_4217
chr10 98068349 98068865
P19_NANOG_CNCC_D5_mem_q10_srt_rmdup_peaks_peak_4219
chr10 98739494 98739821
P19_NANOG_CNCC_D5_mem_q10_srt_rmdup_peaks_peak_4230
chr10 98962997 98963268

P19_NANOG_CNCC_D5_mem_q10_srt_rmdup_peaks_peak_4236
chr10 99486448 99486899
P19_NANOG_CNCC_D5_mem_q10_srt_rmdup_peaks_peak_4249
chr10 99713068 99713408
P19_NANOG_CNCC_D5_mem_q10_srt_rmdup_peaks_peak_4252
chr10 100978744 100979196
P19_NANOG_CNCC_D5_mem_q10_srt_rmdup_peaks_peak_4261
chr10 101210893 101211288
P19_NANOG_CNCC_D5_mem_q10_srt_rmdup_peaks_peak_4266
chr10 102118782 102119197
P19_NANOG_CNCC_D5_mem_q10_srt_rmdup_peaks_peak_4284
chr10 103113387 103113713
P19_NANOG_CNCC_D5_mem_q10_srt_rmdup_peaks_peak_4315
chr10 103113864 103114309
P19_NANOG_CNCC_D5_mem_q10_srt_rmdup_peaks_peak_4316
chr10 103366878 103367196
P19_NANOG_CNCC_D5_mem_q10_srt_rmdup_peaks_peak_4324
chr10 103467782 103468129
P19_NANOG_CNCC_D5_mem_q10_srt_rmdup_peaks_peak_4328
chr10 104070882 104071153
P19_NANOG_CNCC_D5_mem_q10_srt_rmdup_peaks_peak_4339
chr10 105114339 105114699
P19_NANOG_CNCC_D5_mem_q10_srt_rmdup_peaks_peak_4361
chr10 105688546 105689018
P19_NANOG_CNCC_D5_mem_q10_srt_rmdup_peaks_peak_4369
chr10 106179801 106180080
P19_NANOG_CNCC_D5_mem_q10_srt_rmdup_peaks_peak_4380
chr10 106862718 106863088
P19_NANOG_CNCC_D5_mem_q10_srt_rmdup_peaks_peak_4386
chr10 107812823 107813168
P19_NANOG_CNCC_D5_mem_q10_srt_rmdup_peaks_peak_4391
chr10 111652683 111653037
P19_NANOG_CNCC_D5_mem_q10_srt_rmdup_peaks_peak_4404
chr10 114343087 114343391
P19_NANOG_CNCC_D5_mem_q10_srt_rmdup_peaks_peak_4435
chr10 114748799 114749198
P19_NANOG_CNCC_D5_mem_q10_srt_rmdup_peaks_peak_4447
chr10 114868014 114868348
P19_NANOG_CNCC_D5_mem_q10_srt_rmdup_peaks_peak_4452
chr10 115354332 115354830
P19_NANOG_CNCC_D5_mem_q10_srt_rmdup_peaks_peak_4462
chr10 116163250 116163545
P19_NANOG_CNCC_D5_mem_q10_srt_rmdup_peaks_peak_4471
chr10 117896912 117897183
P19_NANOG_CNCC_D5_mem_q10_srt_rmdup_peaks_peak_4486
chr10 118608953 118609291
P19_NANOG_CNCC_D5_mem_q10_srt_rmdup_peaks_peak_4496
chr10 118794828 118795148
P19_NANOG_CNCC_D5_mem_q10_srt_rmdup_peaks_peak_4502
chr10 119112048 119112398

P19_NANOG_CNCC_D5_mem_q10_srt_rmdup_peaks_peak_4511
chr10 119964954 119965225
P19_NANOG_CNCC_D5_mem_q10_srt_rmdup_peaks_peak_4532
chr10 121152544 121152873
P19_NANOG_CNCC_D5_mem_q10_srt_rmdup_peaks_peak_4546
chr10 121221797 121222201
P19_NANOG_CNCC_D5_mem_q10_srt_rmdup_peaks_peak_4547
chr10 121834819 121835180
P19_NANOG_CNCC_D5_mem_q10_srt_rmdup_peaks_peak_4556
chr10 121890767 121891129
P19_NANOG_CNCC_D5_mem_q10_srt_rmdup_peaks_peak_4560
chr10 122562386 122562893
P19_NANOG_CNCC_D5_mem_q10_srt_rmdup_peaks_peak_4567
chr10 122722528 122723012
P19_NANOG_CNCC_D5_mem_q10_srt_rmdup_peaks_peak_4569
chr10 122739397 122739732
P19_NANOG_CNCC_D5_mem_q10_srt_rmdup_peaks_peak_4570
chr10 122912479 122912868
P19_NANOG_CNCC_D5_mem_q10_srt_rmdup_peaks_peak_4574
chr10 123349646 123350001
P19_NANOG_CNCC_D5_mem_q10_srt_rmdup_peaks_peak_4583
chr10 124632742 124633025
P19_NANOG_CNCC_D5_mem_q10_srt_rmdup_peaks_peak_4601
chr10 124903277 124903548
P19_NANOG_CNCC_D5_mem_q10_srt_rmdup_peaks_peak_4610
chr10 125010140 125010442
P19_NANOG_CNCC_D5_mem_q10_srt_rmdup_peaks_peak_4614
chr10 125272993 125273322
P19_NANOG_CNCC_D5_mem_q10_srt_rmdup_peaks_peak_4623
chr10 125488304 125488689
P19_NANOG_CNCC_D5_mem_q10_srt_rmdup_peaks_peak_4628
chr10 125514688 125514993
P19_NANOG_CNCC_D5_mem_q10_srt_rmdup_peaks_peak_4630
chr10 126138630 126138918
P19_NANOG_CNCC_D5_mem_q10_srt_rmdup_peaks_peak_4639
chr10 126490166 126490598
P19_NANOG_CNCC_D5_mem_q10_srt_rmdup_peaks_peak_4646
chr10 126864218 126864518
P19_NANOG_CNCC_D5_mem_q10_srt_rmdup_peaks_peak_4651
chr10 127568034 127568339
P19_NANOG_CNCC_D5_mem_q10_srt_rmdup_peaks_peak_4658
chr10 128531386 128531720
P19_NANOG_CNCC_D5_mem_q10_srt_rmdup_peaks_peak_4669
chr10 128879006 128879311
P19_NANOG_CNCC_D5_mem_q10_srt_rmdup_peaks_peak_4670
chr10 134528684 134529137
P19_NANOG_CNCC_D5_mem_q10_srt_rmdup_peaks_peak_4721
chr10 134957583 134958029
P19_NANOG_CNCC_D5_mem_q10_srt_rmdup_peaks_peak_4728
chr11 683484 683855

P19_NANOG_CNCC_D5_mem_q10_srt_rmdup_peaks_peak_4741
chr11 2349861 2350207
P19_NANOG_CNCC_D5_mem_q10_srt_rmdup_peaks_peak_4755
chr11 3116966 3117360
P19_NANOG_CNCC_D5_mem_q10_srt_rmdup_peaks_peak_4767
chr11 4115668 4116154
P19_NANOG_CNCC_D5_mem_q10_srt_rmdup_peaks_peak_4775
chr11 5642817 5643148
P19_NANOG_CNCC_D5_mem_q10_srt_rmdup_peaks_peak_4783
chr11 6711945 6712241
P19_NANOG_CNCC_D5_mem_q10_srt_rmdup_peaks_peak_4795
chr11 6838922 6839210
P19_NANOG_CNCC_D5_mem_q10_srt_rmdup_peaks_peak_4797
chr11 7098519 7098790
P19_NANOG_CNCC_D5_mem_q10_srt_rmdup_peaks_peak_4799
chr11 7620521 7620817
P19_NANOG_CNCC_D5_mem_q10_srt_rmdup_peaks_peak_4803
chr11 8350432 8350774
P19_NANOG_CNCC_D5_mem_q10_srt_rmdup_peaks_peak_4814
chr11 8970575 8971004
P19_NANOG_CNCC_D5_mem_q10_srt_rmdup_peaks_peak_4825
chr11 9824780 9825185
P19_NANOG_CNCC_D5_mem_q10_srt_rmdup_peaks_peak_4840
chr11 9860777 9861104
P19_NANOG_CNCC_D5_mem_q10_srt_rmdup_peaks_peak_4843
chr11 9945168 9945463
P19_NANOG_CNCC_D5_mem_q10_srt_rmdup_peaks_peak_4848
chr11 10315471 10315750
P19_NANOG_CNCC_D5_mem_q10_srt_rmdup_peaks_peak_4850
chr11 10329457 10329817
P19_NANOG_CNCC_D5_mem_q10_srt_rmdup_peaks_peak_4851
chr11 11093639 11094066
P19_NANOG_CNCC_D5_mem_q10_srt_rmdup_peaks_peak_4865
chr11 11444165 11444635
P19_NANOG_CNCC_D5_mem_q10_srt_rmdup_peaks_peak_4870
chr11 11476382 11476670
P19_NANOG_CNCC_D5_mem_q10_srt_rmdup_peaks_peak_4871
chr11 11522289 11522600
P19_NANOG_CNCC_D5_mem_q10_srt_rmdup_peaks_peak_4872
chr11 11531066 11531372
P19_NANOG_CNCC_D5_mem_q10_srt_rmdup_peaks_peak_4873
chr11 12767498 12767811
P19_NANOG_CNCC_D5_mem_q10_srt_rmdup_peaks_peak_4890
chr11 12847333 12847604
P19_NANOG_CNCC_D5_mem_q10_srt_rmdup_peaks_peak_4894
chr11 12916479 12916897
P19_NANOG_CNCC_D5_mem_q10_srt_rmdup_peaks_peak_4899
chr11 15128242 15128620
P19_NANOG_CNCC_D5_mem_q10_srt_rmdup_peaks_peak_4936
chr11 16491534 16491867

P19_NANOG_CNCC_D5_mem_q10_srt_rmdup_peaks_peak_4955
chr11 16961931 16962284
P19_NANOG_CNCC_D5_mem_q10_srt_rmdup_peaks_peak_4964
chr11 17043240 17043520
P19_NANOG_CNCC_D5_mem_q10_srt_rmdup_peaks_peak_4967
chr11 17342840 17343169
P19_NANOG_CNCC_D5_mem_q10_srt_rmdup_peaks_peak_4970
chr11 17707993 17708404
P19_NANOG_CNCC_D5_mem_q10_srt_rmdup_peaks_peak_4975
chr11 17733654 17734238
P19_NANOG_CNCC_D5_mem_q10_srt_rmdup_peaks_peak_4978
chr11 19452461 19452878
P19_NANOG_CNCC_D5_mem_q10_srt_rmdup_peaks_peak_4991
chr11 19561369 19561686
P19_NANOG_CNCC_D5_mem_q10_srt_rmdup_peaks_peak_4992
chr11 20311277 20311684
P19_NANOG_CNCC_D5_mem_q10_srt_rmdup_peaks_peak_5019
chr11 20761443 20761764
P19_NANOG_CNCC_D5_mem_q10_srt_rmdup_peaks_peak_5029
chr11 21093119 21093719
P19_NANOG_CNCC_D5_mem_q10_srt_rmdup_peaks_peak_5036
chr11 22923592 22923988
P19_NANOG_CNCC_D5_mem_q10_srt_rmdup_peaks_peak_5047
chr11 25698391 25698695
P19_NANOG_CNCC_D5_mem_q10_srt_rmdup_peaks_peak_5059
chr11 27371305 27371642
P19_NANOG_CNCC_D5_mem_q10_srt_rmdup_peaks_peak_5070
chr11 27462282 27462672
P19_NANOG_CNCC_D5_mem_q10_srt_rmdup_peaks_peak_5072
chr11 30501399 30501918
P19_NANOG_CNCC_D5_mem_q10_srt_rmdup_peaks_peak_5102
chr11 30852249 30852520
P19_NANOG_CNCC_D5_mem_q10_srt_rmdup_peaks_peak_5110
chr11 30884171 30884696
P19_NANOG_CNCC_D5_mem_q10_srt_rmdup_peaks_peak_5111
chr11 31131061 31131455
P19_NANOG_CNCC_D5_mem_q10_srt_rmdup_peaks_peak_5112
chr11 31160933 31161519
P19_NANOG_CNCC_D5_mem_q10_srt_rmdup_peaks_peak_5113
chr11 31672449 31673130
P19_NANOG_CNCC_D5_mem_q10_srt_rmdup_peaks_peak_5122
chr11 31741748 31742027
P19_NANOG_CNCC_D5_mem_q10_srt_rmdup_peaks_peak_5123
chr11 31960787 31961221
P19_NANOG_CNCC_D5_mem_q10_srt_rmdup_peaks_peak_5136
chr11 32619583 32619861
P19_NANOG_CNCC_D5_mem_q10_srt_rmdup_peaks_peak_5143
chr11 32814264 32814556
P19_NANOG_CNCC_D5_mem_q10_srt_rmdup_peaks_peak_5144
chr11 32851048 32851406

P19_NANOG_CNCC_D5_mem_q10_srt_rmdup_peaks_peak_5145
chr11 33155257 33155588
P19_NANOG_CNCC_D5_mem_q10_srt_rmdup_peaks_peak_5152
chr11 33397976 33398522
P19_NANOG_CNCC_D5_mem_q10_srt_rmdup_peaks_peak_5155
chr11 34226356 34226908
P19_NANOG_CNCC_D5_mem_q10_srt_rmdup_peaks_peak_5160
chr11 34258084 34258457
P19_NANOG_CNCC_D5_mem_q10_srt_rmdup_peaks_peak_5163
chr11 34568045 34568427
P19_NANOG_CNCC_D5_mem_q10_srt_rmdup_peaks_peak_5168
chr11 34817170 34817461
P19_NANOG_CNCC_D5_mem_q10_srt_rmdup_peaks_peak_5172
chr11 34847696 34848087
P19_NANOG_CNCC_D5_mem_q10_srt_rmdup_peaks_peak_5173
chr11 35081758 35082166
P19_NANOG_CNCC_D5_mem_q10_srt_rmdup_peaks_peak_5175
chr11 35317020 35317401
P19_NANOG_CNCC_D5_mem_q10_srt_rmdup_peaks_peak_5181
chr11 35808403 35808719
P19_NANOG_CNCC_D5_mem_q10_srt_rmdup_peaks_peak_5188
chr11 35965558 35965923
P19_NANOG_CNCC_D5_mem_q10_srt_rmdup_peaks_peak_5191
chr11 35987397 35987881
P19_NANOG_CNCC_D5_mem_q10_srt_rmdup_peaks_peak_5192
chr11 36114163 36114640
P19_NANOG_CNCC_D5_mem_q10_srt_rmdup_peaks_peak_5197
chr11 36310762 36311123
P19_NANOG_CNCC_D5_mem_q10_srt_rmdup_peaks_peak_5201
chr11 36768625 36768914
P19_NANOG_CNCC_D5_mem_q10_srt_rmdup_peaks_peak_5205
chr11 37525862 37526250
P19_NANOG_CNCC_D5_mem_q10_srt_rmdup_peaks_peak_5207
chr11 38347971 38348329
P19_NANOG_CNCC_D5_mem_q10_srt_rmdup_peaks_peak_5215
chr11 38604376 38604991
P19_NANOG_CNCC_D5_mem_q10_srt_rmdup_peaks_peak_5216
chr11 40738376 40738653
P19_NANOG_CNCC_D5_mem_q10_srt_rmdup_peaks_peak_5227
chr11 41479832 41480272
P19_NANOG_CNCC_D5_mem_q10_srt_rmdup_peaks_peak_5236
chr11 42103754 42104213
P19_NANOG_CNCC_D5_mem_q10_srt_rmdup_peaks_peak_5242
chr11 43300078 43300440
P19_NANOG_CNCC_D5_mem_q10_srt_rmdup_peaks_peak_5250
chr11 43975847 43976191
P19_NANOG_CNCC_D5_mem_q10_srt_rmdup_peaks_peak_5256
chr11 44545107 44545414
P19_NANOG_CNCC_D5_mem_q10_srt_rmdup_peaks_peak_5272
chr11 44710716 44711009

P19_NANOG_CNCC_D5_mem_q10_srt_rmdup_peaks_peak_5277
chr11 44883868 44884241
P19_NANOG_CNCC_D5_mem_q10_srt_rmdup_peaks_peak_5288
chr11 45864734 45865091
P19_NANOG_CNCC_D5_mem_q10_srt_rmdup_peaks_peak_5309
chr11 45874372 45874783
P19_NANOG_CNCC_D5_mem_q10_srt_rmdup_peaks_peak_5310
chr11 45917944 45918217
P19_NANOG_CNCC_D5_mem_q10_srt_rmdup_peaks_peak_5311
chr11 47270213 47270519
P19_NANOG_CNCC_D5_mem_q10_srt_rmdup_peaks_peak_5349
chr11 49319520 49319964
P19_NANOG_CNCC_D5_mem_q10_srt_rmdup_peaks_peak_5365
chr11 49526564 49527398
P19_NANOG_CNCC_D5_mem_q10_srt_rmdup_peaks_peak_5366
chr11 55753557 55754050
P19_NANOG_CNCC_D5_mem_q10_srt_rmdup_peaks_peak_5367
chr11 56105072 56105563
P19_NANOG_CNCC_D5_mem_q10_srt_rmdup_peaks_peak_5369
chr11 57335574 57335927
P19_NANOG_CNCC_D5_mem_q10_srt_rmdup_peaks_peak_5380
chr11 57610182 57610510
P19_NANOG_CNCC_D5_mem_q10_srt_rmdup_peaks_peak_5390
chr11 58731094 58731473
P19_NANOG_CNCC_D5_mem_q10_srt_rmdup_peaks_peak_5398
chr11 58822466 58822779
P19_NANOG_CNCC_D5_mem_q10_srt_rmdup_peaks_peak_5400
chr11 58947786 58948100
P19_NANOG_CNCC_D5_mem_q10_srt_rmdup_peaks_peak_5403
chr11 59333530 59333833
P19_NANOG_CNCC_D5_mem_q10_srt_rmdup_peaks_peak_5407
chr11 59577994 59578390
P19_NANOG_CNCC_D5_mem_q10_srt_rmdup_peaks_peak_5410
chr11 60088570 60088982
P19_NANOG_CNCC_D5_mem_q10_srt_rmdup_peaks_peak_5417
chr11 60512747 60513086
P19_NANOG_CNCC_D5_mem_q10_srt_rmdup_peaks_peak_5419
chr11 60619643 60620137
P19_NANOG_CNCC_D5_mem_q10_srt_rmdup_peaks_peak_5420
chr11 60809660 60810052
P19_NANOG_CNCC_D5_mem_q10_srt_rmdup_peaks_peak_5423
chr11 61335044 61335356
P19_NANOG_CNCC_D5_mem_q10_srt_rmdup_peaks_peak_5429
chr11 62123015 62123559
P19_NANOG_CNCC_D5_mem_q10_srt_rmdup_peaks_peak_5440
chr11 62319278 62319645
P19_NANOG_CNCC_D5_mem_q10_srt_rmdup_peaks_peak_5444
chr11 62647068 62647381
P19_NANOG_CNCC_D5_mem_q10_srt_rmdup_peaks_peak_5458
chr11 63275462 63275733

P19_NANOG_CNCC_D5_mem_q10_srt_rmdup_peaks_peak_5467
chr11 63298852 63299420
P19_NANOG_CNCC_D5_mem_q10_srt_rmdup_peaks_peak_5471
chr11 64397782 64398065
P19_NANOG_CNCC_D5_mem_q10_srt_rmdup_peaks_peak_5495
chr11 65082942 65083329
P19_NANOG_CNCC_D5_mem_q10_srt_rmdup_peaks_peak_5505
chr11 65121722 65122095
P19_NANOG_CNCC_D5_mem_q10_srt_rmdup_peaks_peak_5506
chr11 65547468 65547778
P19_NANOG_CNCC_D5_mem_q10_srt_rmdup_peaks_peak_5521
chr11 66007635 66007906
P19_NANOG_CNCC_D5_mem_q10_srt_rmdup_peaks_peak_5533
chr11 66095791 66096292
P19_NANOG_CNCC_D5_mem_q10_srt_rmdup_peaks_peak_5535
chr11 66139676 66140005
P19_NANOG_CNCC_D5_mem_q10_srt_rmdup_peaks_peak_5536
chr11 66610726 66611055
P19_NANOG_CNCC_D5_mem_q10_srt_rmdup_peaks_peak_5550
chr11 67468424 67468705
P19_NANOG_CNCC_D5_mem_q10_srt_rmdup_peaks_peak_5567
chr11 67654280 67654625
P19_NANOG_CNCC_D5_mem_q10_srt_rmdup_peaks_peak_5570
chr11 68110245 68110542
P19_NANOG_CNCC_D5_mem_q10_srt_rmdup_peaks_peak_5576
chr11 68227422 68227772
P19_NANOG_CNCC_D5_mem_q10_srt_rmdup_peaks_peak_5578
chr11 68400336 68400643
P19_NANOG_CNCC_D5_mem_q10_srt_rmdup_peaks_peak_5581
chr11 69451262 69451692
P19_NANOG_CNCC_D5_mem_q10_srt_rmdup_peaks_peak_5595
chr11 70235300 70235683
P19_NANOG_CNCC_D5_mem_q10_srt_rmdup_peaks_peak_5616
chr11 72135307 72135624
P19_NANOG_CNCC_D5_mem_q10_srt_rmdup_peaks_peak_5638
chr11 72273681 72273958
P19_NANOG_CNCC_D5_mem_q10_srt_rmdup_peaks_peak_5641
chr11 72853170 72853598
P19_NANOG_CNCC_D5_mem_q10_srt_rmdup_peaks_peak_5646
chr11 73249438 73249934
P19_NANOG_CNCC_D5_mem_q10_srt_rmdup_peaks_peak_5650
chr11 73287085 73287479
P19_NANOG_CNCC_D5_mem_q10_srt_rmdup_peaks_peak_5651
chr11 73852318 73852705
P19_NANOG_CNCC_D5_mem_q10_srt_rmdup_peaks_peak_5660
chr11 73960424 73960923
P19_NANOG_CNCC_D5_mem_q10_srt_rmdup_peaks_peak_5662
chr11 74466453 74466839
P19_NANOG_CNCC_D5_mem_q10_srt_rmdup_peaks_peak_5664
chr11 74763322 74763749

P19_NANOG_CNCC_D5_mem_q10_srt_rmdup_peaks_peak_5666
chr11 75099687 75099984
P19_NANOG_CNCC_D5_mem_q10_srt_rmdup_peaks_peak_5670
chr11 75701498 75701834
P19_NANOG_CNCC_D5_mem_q10_srt_rmdup_peaks_peak_5677
chr11 75921707 75921978
P19_NANOG_CNCC_D5_mem_q10_srt_rmdup_peaks_peak_5679
chr11 76777653 76777942
P19_NANOG_CNCC_D5_mem_q10_srt_rmdup_peaks_peak_5691
chr11 76802238 76802556
P19_NANOG_CNCC_D5_mem_q10_srt_rmdup_peaks_peak_5692
chr11 77899492 77899790
P19_NANOG_CNCC_D5_mem_q10_srt_rmdup_peaks_peak_5704
chr11 79143583 79143952
P19_NANOG_CNCC_D5_mem_q10_srt_rmdup_peaks_peak_5724
chr11 79152452 79152731
P19_NANOG_CNCC_D5_mem_q10_srt_rmdup_peaks_peak_5725
chr11 79254236 79254608
P19_NANOG_CNCC_D5_mem_q10_srt_rmdup_peaks_peak_5728
chr11 80564743 80565147
P19_NANOG_CNCC_D5_mem_q10_srt_rmdup_peaks_peak_5736
chr11 82756412 82756800
P19_NANOG_CNCC_D5_mem_q10_srt_rmdup_peaks_peak_5748
chr11 83868664 83869125
P19_NANOG_CNCC_D5_mem_q10_srt_rmdup_peaks_peak_5758
chr11 84121249 84121520
P19_NANOG_CNCC_D5_mem_q10_srt_rmdup_peaks_peak_5760
chr11 85565837 85566322
P19_NANOG_CNCC_D5_mem_q10_srt_rmdup_peaks_peak_5780
chr11 85647357 85647628
P19_NANOG_CNCC_D5_mem_q10_srt_rmdup_peaks_peak_5782
chr11 86518490 86518769
P19_NANOG_CNCC_D5_mem_q10_srt_rmdup_peaks_peak_5792
chr11 86749217 86749606
P19_NANOG_CNCC_D5_mem_q10_srt_rmdup_peaks_peak_5796
chr11 87284995 87285522
P19_NANOG_CNCC_D5_mem_q10_srt_rmdup_peaks_peak_5803
chr11 91952687 91952961
P19_NANOG_CNCC_D5_mem_q10_srt_rmdup_peaks_peak_5833
chr11 93231347 93231631
P19_NANOG_CNCC_D5_mem_q10_srt_rmdup_peaks_peak_5858
chr11 93612324 93612672
P19_NANOG_CNCC_D5_mem_q10_srt_rmdup_peaks_peak_5865
chr11 94395507 94395897
P19_NANOG_CNCC_D5_mem_q10_srt_rmdup_peaks_peak_5873
chr11 95159786 95160196
P19_NANOG_CNCC_D5_mem_q10_srt_rmdup_peaks_peak_5886
chr11 95349578 95350061
P19_NANOG_CNCC_D5_mem_q10_srt_rmdup_peaks_peak_5889
chr11 95931023 95931353

P19_NANOG_CNCC_D5_mem_q10_srt_rmdup_peaks_peak_5893
chr11 96197061 96197332
P19_NANOG_CNCC_D5_mem_q10_srt_rmdup_peaks_peak_5896
chr11 96518651 96519078
P19_NANOG_CNCC_D5_mem_q10_srt_rmdup_peaks_peak_5901
chr11 97507817 97508155
P19_NANOG_CNCC_D5_mem_q10_srt_rmdup_peaks_peak_5903
chr11 98832523 98832794
P19_NANOG_CNCC_D5_mem_q10_srt_rmdup_peaks_peak_5910
chr11 99142405 99142715
P19_NANOG_CNCC_D5_mem_q10_srt_rmdup_peaks_peak_5913
chr11 100002135 100002471
P19_NANOG_CNCC_D5_mem_q10_srt_rmdup_peaks_peak_5920
chr11 100059226 100059497
P19_NANOG_CNCC_D5_mem_q10_srt_rmdup_peaks_peak_5921
chr11 100798259 100798646
P19_NANOG_CNCC_D5_mem_q10_srt_rmdup_peaks_peak_5934
chr11 102322319 102322752
P19_NANOG_CNCC_D5_mem_q10_srt_rmdup_peaks_peak_5949
chr11 102322920 102323287
P19_NANOG_CNCC_D5_mem_q10_srt_rmdup_peaks_peak_5950
chr11 102782599 102782987
P19_NANOG_CNCC_D5_mem_q10_srt_rmdup_peaks_peak_5959
chr11 103399333 103399717
P19_NANOG_CNCC_D5_mem_q10_srt_rmdup_peaks_peak_5962
chr11 103452406 103453006
P19_NANOG_CNCC_D5_mem_q10_srt_rmdup_peaks_peak_5963
chr11 103454871 103455195
P19_NANOG_CNCC_D5_mem_q10_srt_rmdup_peaks_peak_5964
chr11 105737544 105738036
P19_NANOG_CNCC_D5_mem_q10_srt_rmdup_peaks_peak_5976
chr11 107027908 107028227
P19_NANOG_CNCC_D5_mem_q10_srt_rmdup_peaks_peak_5991
chr11 107125423 107125694
P19_NANOG_CNCC_D5_mem_q10_srt_rmdup_peaks_peak_5992
chr11 107143262 107143615
P19_NANOG_CNCC_D5_mem_q10_srt_rmdup_peaks_peak_5993
chr11 107468828 107469388
P19_NANOG_CNCC_D5_mem_q10_srt_rmdup_peaks_peak_5995
chr11 107537545 107537978
P19_NANOG_CNCC_D5_mem_q10_srt_rmdup_peaks_peak_5997
chr11 107583553 107583840
P19_NANOG_CNCC_D5_mem_q10_srt_rmdup_peaks_peak_5999
chr11 107615559 107615889
P19_NANOG_CNCC_D5_mem_q10_srt_rmdup_peaks_peak_6001
chr11 109947068 109947339
P19_NANOG_CNCC_D5_mem_q10_srt_rmdup_peaks_peak_6020
chr11 110233447 110234302
P19_NANOG_CNCC_D5_mem_q10_srt_rmdup_peaks_peak_6030
chr11 111173365 111173762

P19_NANOG_CNCC_D5_mem_q10_srt_rmdup_peaks_peak_6034
chr11	111693845	111694219
P19_NANOG_CNCC_D5_mem_q10_srt_rmdup_peaks_peak_6044
chr11	111847193	111847498
P19_NANOG_CNCC_D5_mem_q10_srt_rmdup_peaks_peak_6049
chr11	111852716	111853103
P19_NANOG_CNCC_D5_mem_q10_srt_rmdup_peaks_peak_6050
chr11	112192500	112192892
P19_NANOG_CNCC_D5_mem_q10_srt_rmdup_peaks_peak_6054
chr11	113449946	113450256
P19_NANOG_CNCC_D5_mem_q10_srt_rmdup_peaks_peak_6070
chr11	113756475	113756813
P19_NANOG_CNCC_D5_mem_q10_srt_rmdup_peaks_peak_6076
chr11	113845773	113846094
P19_NANOG_CNCC_D5_mem_q10_srt_rmdup_peaks_peak_6077
chr11	114173421	114173700
P19_NANOG_CNCC_D5_mem_q10_srt_rmdup_peaks_peak_6081
chr11	114190722	114191018
P19_NANOG_CNCC_D5_mem_q10_srt_rmdup_peaks_peak_6083
chr11	114351170	114351572
P19_NANOG_CNCC_D5_mem_q10_srt_rmdup_peaks_peak_6086
chr11	115461259	115461530
P19_NANOG_CNCC_D5_mem_q10_srt_rmdup_peaks_peak_6102
chr11	115535897	115536423
P19_NANOG_CNCC_D5_mem_q10_srt_rmdup_peaks_peak_6106
chr11	115904804	115905087
P19_NANOG_CNCC_D5_mem_q10_srt_rmdup_peaks_peak_6112
chr11	116404187	116404603
P19_NANOG_CNCC_D5_mem_q10_srt_rmdup_peaks_peak_6121
chr11	116929496	116929859
P19_NANOG_CNCC_D5_mem_q10_srt_rmdup_peaks_peak_6125
chr11	117594494	117594938
P19_NANOG_CNCC_D5_mem_q10_srt_rmdup_peaks_peak_6135
chr11	117605173	117605496
P19_NANOG_CNCC_D5_mem_q10_srt_rmdup_peaks_peak_6136
chr11	118067560	118068077
P19_NANOG_CNCC_D5_mem_q10_srt_rmdup_peaks_peak_6144
chr11	118486924	118487208
P19_NANOG_CNCC_D5_mem_q10_srt_rmdup_peaks_peak_6155
chr11	119295837	119296133
P19_NANOG_CNCC_D5_mem_q10_srt_rmdup_peaks_peak_6173
chr11	119496723	119497075
P19_NANOG_CNCC_D5_mem_q10_srt_rmdup_peaks_peak_6180
chr11	120070285	120070764
P19_NANOG_CNCC_D5_mem_q10_srt_rmdup_peaks_peak_6192
chr11	120436993	120437373
P19_NANOG_CNCC_D5_mem_q10_srt_rmdup_peaks_peak_6201
chr11	120776909	120777209
P19_NANOG_CNCC_D5_mem_q10_srt_rmdup_peaks_peak_6208
chr11	120869258	120869759

P19_NANOG_CNCC_D5_mem_q10_srt_rmdup_peaks_peak_6209
chr11 120882803 120883091
P19_NANOG_CNCC_D5_mem_q10_srt_rmdup_peaks_peak_6210
chr11 120971501 120971977
P19_NANOG_CNCC_D5_mem_q10_srt_rmdup_peaks_peak_6212
chr11 121245893 121246194
P19_NANOG_CNCC_D5_mem_q10_srt_rmdup_peaks_peak_6219
chr11 121322976 121323290
P19_NANOG_CNCC_D5_mem_q10_srt_rmdup_peaks_peak_6224
chr11 121526206 121526499
P19_NANOG_CNCC_D5_mem_q10_srt_rmdup_peaks_peak_6230
chr11 121559856 121560362
P19_NANOG_CNCC_D5_mem_q10_srt_rmdup_peaks_peak_6231
chr11 121679001 121679307
P19_NANOG_CNCC_D5_mem_q10_srt_rmdup_peaks_peak_6234
chr11 122392197 122392703
P19_NANOG_CNCC_D5_mem_q10_srt_rmdup_peaks_peak_6247
chr11 122675555 122675918
P19_NANOG_CNCC_D5_mem_q10_srt_rmdup_peaks_peak_6250
chr11 122703090 122703438
P19_NANOG_CNCC_D5_mem_q10_srt_rmdup_peaks_peak_6252
chr11 122901162 122901530
P19_NANOG_CNCC_D5_mem_q10_srt_rmdup_peaks_peak_6255
chr11 123657451 123658074
P19_NANOG_CNCC_D5_mem_q10_srt_rmdup_peaks_peak_6270
chr11 125821569 125821854
P19_NANOG_CNCC_D5_mem_q10_srt_rmdup_peaks_peak_6303
chr11 126601559 126601895
P19_NANOG_CNCC_D5_mem_q10_srt_rmdup_peaks_peak_6321
chr11 127331834 127332211
P19_NANOG_CNCC_D5_mem_q10_srt_rmdup_peaks_peak_6331
chr11 127479283 127479648
P19_NANOG_CNCC_D5_mem_q10_srt_rmdup_peaks_peak_6332
chr11 129024977 129025248
P19_NANOG_CNCC_D5_mem_q10_srt_rmdup_peaks_peak_6349
chr11 129079871 129080210
P19_NANOG_CNCC_D5_mem_q10_srt_rmdup_peaks_peak_6351
chr11 129161021 129161311
P19_NANOG_CNCC_D5_mem_q10_srt_rmdup_peaks_peak_6353
chr11 129161445 129161816
P19_NANOG_CNCC_D5_mem_q10_srt_rmdup_peaks_peak_6354
chr11 129201773 129202056
P19_NANOG_CNCC_D5_mem_q10_srt_rmdup_peaks_peak_6355
chr11 129272589 129272911
P19_NANOG_CNCC_D5_mem_q10_srt_rmdup_peaks_peak_6358
chr11 129907774 129908162
P19_NANOG_CNCC_D5_mem_q10_srt_rmdup_peaks_peak_6362
chr11 130401672 130402005
P19_NANOG_CNCC_D5_mem_q10_srt_rmdup_peaks_peak_6372
chr11 131353765 131354196

P19_NANOG_CNCC_D5_mem_q10_srt_rmdup_peaks_peak_6385
chr11	131381776	131382140
P19_NANOG_CNCC_D5_mem_q10_srt_rmdup_peaks_peak_6386
chr11	131720381	131720687
P19_NANOG_CNCC_D5_mem_q10_srt_rmdup_peaks_peak_6392
chr11	131985402	131985857
P19_NANOG_CNCC_D5_mem_q10_srt_rmdup_peaks_peak_6401
chr11	132056507	132056931
P19_NANOG_CNCC_D5_mem_q10_srt_rmdup_peaks_peak_6402
chr11	132888754	132889025
P19_NANOG_CNCC_D5_mem_q10_srt_rmdup_peaks_peak_6412
chr11	133998036	133998355
P19_NANOG_CNCC_D5_mem_q10_srt_rmdup_peaks_peak_6428
chr11	134374131	134374484
P19_NANOG_CNCC_D5_mem_q10_srt_rmdup_peaks_peak_6432
chr12	831694	832014
P19_NANOG_CNCC_D5_mem_q10_srt_rmdup_peaks_peak_6444
chr12	1427659	1427990
P19_NANOG_CNCC_D5_mem_q10_srt_rmdup_peaks_peak_6452
chr12	2298777	2299050
P19_NANOG_CNCC_D5_mem_q10_srt_rmdup_peaks_peak_6461
chr12	2442756	2443105
P19_NANOG_CNCC_D5_mem_q10_srt_rmdup_peaks_peak_6466
chr12	3244797	3245080
P19_NANOG_CNCC_D5_mem_q10_srt_rmdup_peaks_peak_6478
chr12	3528588	3529009
P19_NANOG_CNCC_D5_mem_q10_srt_rmdup_peaks_peak_6484
chr12	3566063	3566421
P19_NANOG_CNCC_D5_mem_q10_srt_rmdup_peaks_peak_6486
chr12	3901750	3902118
P19_NANOG_CNCC_D5_mem_q10_srt_rmdup_peaks_peak_6491
chr12	5542658	5543168
P19_NANOG_CNCC_D5_mem_q10_srt_rmdup_peaks_peak_6515
chr12	6995059	6995407
P19_NANOG_CNCC_D5_mem_q10_srt_rmdup_peaks_peak_6547
chr12	6995505	6995987
P19_NANOG_CNCC_D5_mem_q10_srt_rmdup_peaks_peak_6548
chr12	7079763	7080233
P19_NANOG_CNCC_D5_mem_q10_srt_rmdup_peaks_peak_6553
chr12	7435089	7435360
P19_NANOG_CNCC_D5_mem_q10_srt_rmdup_peaks_peak_6559
chr12	7449372	7449987
P19_NANOG_CNCC_D5_mem_q10_srt_rmdup_peaks_peak_6560
chr12	8176236	8176541
P19_NANOG_CNCC_D5_mem_q10_srt_rmdup_peaks_peak_6580
chr12	8191408	8191693
P19_NANOG_CNCC_D5_mem_q10_srt_rmdup_peaks_peak_6581
chr12	8614297	8614809
P19_NANOG_CNCC_D5_mem_q10_srt_rmdup_peaks_peak_6585
chr12	8923769	8924046

P19_NANOG_CNCC_D5_mem_q10_srt_rmdup_peaks_peak_6591
chr12 9216833 9217318
P19_NANOG_CNCC_D5_mem_q10_srt_rmdup_peaks_peak_6597
chr12 9408928 9409411
P19_NANOG_CNCC_D5_mem_q10_srt_rmdup_peaks_peak_6599
chr12 9428645 9429018
P19_NANOG_CNCC_D5_mem_q10_srt_rmdup_peaks_peak_6600
chr12 10169567 10169848
P19_NANOG_CNCC_D5_mem_q10_srt_rmdup_peaks_peak_6605
chr12 10479213 10479484
P19_NANOG_CNCC_D5_mem_q10_srt_rmdup_peaks_peak_6607
chr12 11323762 11324443
P19_NANOG_CNCC_D5_mem_q10_srt_rmdup_peaks_peak_6612
chr12 11801585 11801901
P19_NANOG_CNCC_D5_mem_q10_srt_rmdup_peaks_peak_6618
chr12 11895931 11896202
P19_NANOG_CNCC_D5_mem_q10_srt_rmdup_peaks_peak_6620
chr12 11916757 11917124
P19_NANOG_CNCC_D5_mem_q10_srt_rmdup_peaks_peak_6622
chr12 12504022 12504486
P19_NANOG_CNCC_D5_mem_q10_srt_rmdup_peaks_peak_6632
chr12 12578082 12578684
P19_NANOG_CNCC_D5_mem_q10_srt_rmdup_peaks_peak_6633
chr12 12660697 12661156
P19_NANOG_CNCC_D5_mem_q10_srt_rmdup_peaks_peak_6634
chr12 12715499 12715945
P19_NANOG_CNCC_D5_mem_q10_srt_rmdup_peaks_peak_6635
chr12 12796792 12797189
P19_NANOG_CNCC_D5_mem_q10_srt_rmdup_peaks_peak_6637
chr12 13253964 13254468
P19_NANOG_CNCC_D5_mem_q10_srt_rmdup_peaks_peak_6652
chr12 13683766 13684037
P19_NANOG_CNCC_D5_mem_q10_srt_rmdup_peaks_peak_6657
chr12 13854441 13854712
P19_NANOG_CNCC_D5_mem_q10_srt_rmdup_peaks_peak_6660
chr12 15736515 15737006
P19_NANOG_CNCC_D5_mem_q10_srt_rmdup_peaks_peak_6685
chr12 16763664 16763975
P19_NANOG_CNCC_D5_mem_q10_srt_rmdup_peaks_peak_6695
chr12 16941446 16941886
P19_NANOG_CNCC_D5_mem_q10_srt_rmdup_peaks_peak_6697
chr12 17183314 17183712
P19_NANOG_CNCC_D5_mem_q10_srt_rmdup_peaks_peak_6701
chr12 17236035 17236512
P19_NANOG_CNCC_D5_mem_q10_srt_rmdup_peaks_peak_6702
chr12 19124069 19124526
P19_NANOG_CNCC_D5_mem_q10_srt_rmdup_peaks_peak_6713
chr12 19283805 19284121
P19_NANOG_CNCC_D5_mem_q10_srt_rmdup_peaks_peak_6719
chr12 20009562 20009915

P19_NANOG_CNCC_D5_mem_q10_srt_rmdup_peaks_peak_6727
chr12 22514759 22515102
P19_NANOG_CNCC_D5_mem_q10_srt_rmdup_peaks_peak_6747
chr12 22695788 22696179
P19_NANOG_CNCC_D5_mem_q10_srt_rmdup_peaks_peak_6748
chr12 23913354 23913765
P19_NANOG_CNCC_D5_mem_q10_srt_rmdup_peaks_peak_6765
chr12 24714924 24715233
P19_NANOG_CNCC_D5_mem_q10_srt_rmdup_peaks_peak_6777
chr12 24913073 24913462
P19_NANOG_CNCC_D5_mem_q10_srt_rmdup_peaks_peak_6780
chr12 25057811 25058169
P19_NANOG_CNCC_D5_mem_q10_srt_rmdup_peaks_peak_6781
chr12 25100870 25101524
P19_NANOG_CNCC_D5_mem_q10_srt_rmdup_peaks_peak_6784
chr12 25830038 25830309
P19_NANOG_CNCC_D5_mem_q10_srt_rmdup_peaks_peak_6794
chr12 26042017 26042608
P19_NANOG_CNCC_D5_mem_q10_srt_rmdup_peaks_peak_6798
chr12 26120032 26120317
P19_NANOG_CNCC_D5_mem_q10_srt_rmdup_peaks_peak_6799
chr12 26865764 26866374
P19_NANOG_CNCC_D5_mem_q10_srt_rmdup_peaks_peak_6815
chr12 27794130 27794523
P19_NANOG_CNCC_D5_mem_q10_srt_rmdup_peaks_peak_6823
chr12 28104889 28105208
P19_NANOG_CNCC_D5_mem_q10_srt_rmdup_peaks_peak_6826
chr12 30404513 30404813
P19_NANOG_CNCC_D5_mem_q10_srt_rmdup_peaks_peak_6851
chr12 30796169 30796507
P19_NANOG_CNCC_D5_mem_q10_srt_rmdup_peaks_peak_6854
chr12 30862363 30862865
P19_NANOG_CNCC_D5_mem_q10_srt_rmdup_peaks_peak_6855
chr12 30930946 30931326
P19_NANOG_CNCC_D5_mem_q10_srt_rmdup_peaks_peak_6857
chr12 30958558 30959338
P19_NANOG_CNCC_D5_mem_q10_srt_rmdup_peaks_peak_6860
chr12 31412634 31412965
P19_NANOG_CNCC_D5_mem_q10_srt_rmdup_peaks_peak_6864
chr12 31493243 31493523
P19_NANOG_CNCC_D5_mem_q10_srt_rmdup_peaks_peak_6867
chr12 31742704 31743253
P19_NANOG_CNCC_D5_mem_q10_srt_rmdup_peaks_peak_6876
chr12 32139101 32139578
P19_NANOG_CNCC_D5_mem_q10_srt_rmdup_peaks_peak_6885
chr12 32242432 32243028
P19_NANOG_CNCC_D5_mem_q10_srt_rmdup_peaks_peak_6890
chr12 32253151 32253442
P19_NANOG_CNCC_D5_mem_q10_srt_rmdup_peaks_peak_6891
chr12 33417928 33418231

P19_NANOG_CNCC_D5_mem_q10_srt_rmdup_peaks_peak_6907
chr12	41617830	41618286
P19_NANOG_CNCC_D5_mem_q10_srt_rmdup_peaks_peak_6937
chr12	42519118	42519389
P19_NANOG_CNCC_D5_mem_q10_srt_rmdup_peaks_peak_6944
chr12	42763871	42764273
P19_NANOG_CNCC_D5_mem_q10_srt_rmdup_peaks_peak_6946
chr12	43293092	43293510
P19_NANOG_CNCC_D5_mem_q10_srt_rmdup_peaks_peak_6962
chr12	44991671	44991983
P19_NANOG_CNCC_D5_mem_q10_srt_rmdup_peaks_peak_6978
chr12	45829993	45830478
P19_NANOG_CNCC_D5_mem_q10_srt_rmdup_peaks_peak_6988
chr12	46287093	46287364
P19_NANOG_CNCC_D5_mem_q10_srt_rmdup_peaks_peak_6992
chr12	46754756	46755027
P19_NANOG_CNCC_D5_mem_q10_srt_rmdup_peaks_peak_6995
chr12	46877779	46878089
P19_NANOG_CNCC_D5_mem_q10_srt_rmdup_peaks_peak_6999
chr12	48842824	48843132
P19_NANOG_CNCC_D5_mem_q10_srt_rmdup_peaks_peak_7025
chr12	49284359	49284699
P19_NANOG_CNCC_D5_mem_q10_srt_rmdup_peaks_peak_7034
chr12	49362266	49362579
P19_NANOG_CNCC_D5_mem_q10_srt_rmdup_peaks_peak_7036
chr12	49822664	49823297
P19_NANOG_CNCC_D5_mem_q10_srt_rmdup_peaks_peak_7043
chr12	49995999	49996358
P19_NANOG_CNCC_D5_mem_q10_srt_rmdup_peaks_peak_7046
chr12	50878419	50878714
P19_NANOG_CNCC_D5_mem_q10_srt_rmdup_peaks_peak_7062
chr12	50932707	50933031
P19_NANOG_CNCC_D5_mem_q10_srt_rmdup_peaks_peak_7063
chr12	51566760	51567105
P19_NANOG_CNCC_D5_mem_q10_srt_rmdup_peaks_peak_7071
chr12	51659949	51660311
P19_NANOG_CNCC_D5_mem_q10_srt_rmdup_peaks_peak_7072
chr12	51765103	51765535
P19_NANOG_CNCC_D5_mem_q10_srt_rmdup_peaks_peak_7074
chr12	51784836	51785264
P19_NANOG_CNCC_D5_mem_q10_srt_rmdup_peaks_peak_7075
chr12	52241090	52241361
P19_NANOG_CNCC_D5_mem_q10_srt_rmdup_peaks_peak_7080
chr12	52249764	52250102
P19_NANOG_CNCC_D5_mem_q10_srt_rmdup_peaks_peak_7081
chr12	52419540	52419873
P19_NANOG_CNCC_D5_mem_q10_srt_rmdup_peaks_peak_7086
chr12	53319252	53319642
P19_NANOG_CNCC_D5_mem_q10_srt_rmdup_peaks_peak_7095
chr12	53397845	53398324

P19_NANOG_CNCC_D5_mem_q10_srt_rmdup_peaks_peak_7100
chr12 53715138 53715463
P19_NANOG_CNCC_D5_mem_q10_srt_rmdup_peaks_peak_7108
chr12 53718595 53718897
P19_NANOG_CNCC_D5_mem_q10_srt_rmdup_peaks_peak_7109
chr12 54071041 54071431
P19_NANOG_CNCC_D5_mem_q10_srt_rmdup_peaks_peak_7123
chr12 55129171 55129581
P19_NANOG_CNCC_D5_mem_q10_srt_rmdup_peaks_peak_7160
chr12 58419237 58419516
P19_NANOG_CNCC_D5_mem_q10_srt_rmdup_peaks_peak_7216
chr12 61374250 61374839
P19_NANOG_CNCC_D5_mem_q10_srt_rmdup_peaks_peak_7232
chr12 62646746 62647081
P19_NANOG_CNCC_D5_mem_q10_srt_rmdup_peaks_peak_7242
chr12 63111331 63111873
P19_NANOG_CNCC_D5_mem_q10_srt_rmdup_peaks_peak_7251
chr12 64286972 64287283
P19_NANOG_CNCC_D5_mem_q10_srt_rmdup_peaks_peak_7262
chr12 64714348 64714685
P19_NANOG_CNCC_D5_mem_q10_srt_rmdup_peaks_peak_7265
chr12 65085078 65085707
P19_NANOG_CNCC_D5_mem_q10_srt_rmdup_peaks_peak_7274
chr12 65283040 65283765
P19_NANOG_CNCC_D5_mem_q10_srt_rmdup_peaks_peak_7276
chr12 66846975 66847524
P19_NANOG_CNCC_D5_mem_q10_srt_rmdup_peaks_peak_7292
chr12 67082608 67082879
P19_NANOG_CNCC_D5_mem_q10_srt_rmdup_peaks_peak_7296
chr12 67812887 67813253
P19_NANOG_CNCC_D5_mem_q10_srt_rmdup_peaks_peak_7301
chr12 67915909 67916236
P19_NANOG_CNCC_D5_mem_q10_srt_rmdup_peaks_peak_7305
chr12 68542303 68542683
P19_NANOG_CNCC_D5_mem_q10_srt_rmdup_peaks_peak_7313
chr12 71589054 71589440
P19_NANOG_CNCC_D5_mem_q10_srt_rmdup_peaks_peak_7354
chr12 72608433 72609466
P19_NANOG_CNCC_D5_mem_q10_srt_rmdup_peaks_peak_7361
chr12 72772782 72773157
P19_NANOG_CNCC_D5_mem_q10_srt_rmdup_peaks_peak_7362
chr12 74071557 74071851
P19_NANOG_CNCC_D5_mem_q10_srt_rmdup_peaks_peak_7367
chr12 74162524 74162941
P19_NANOG_CNCC_D5_mem_q10_srt_rmdup_peaks_peak_7368
chr12 74802261 74802767
P19_NANOG_CNCC_D5_mem_q10_srt_rmdup_peaks_peak_7371
chr12 76278332 76278662
P19_NANOG_CNCC_D5_mem_q10_srt_rmdup_peaks_peak_7385
chr12 76924925 76925281

P19_NANOG_CNCC_D5_mem_q10_srt_rmdup_peaks_peak_7401
chr12 77713056 77713362
P19_NANOG_CNCC_D5_mem_q10_srt_rmdup_peaks_peak_7410
chr12 78070312 78070640
P19_NANOG_CNCC_D5_mem_q10_srt_rmdup_peaks_peak_7415
chr12 78359549 78359932
P19_NANOG_CNCC_D5_mem_q10_srt_rmdup_peaks_peak_7417
chr12 78767713 78768176
P19_NANOG_CNCC_D5_mem_q10_srt_rmdup_peaks_peak_7419
chr12 79590362 79590677
P19_NANOG_CNCC_D5_mem_q10_srt_rmdup_peaks_peak_7427
chr12 80048523 80049199
P19_NANOG_CNCC_D5_mem_q10_srt_rmdup_peaks_peak_7435
chr12 80081747 80082046
P19_NANOG_CNCC_D5_mem_q10_srt_rmdup_peaks_peak_7436
chr12 80238390 80238800
P19_NANOG_CNCC_D5_mem_q10_srt_rmdup_peaks_peak_7439
chr12 81109412 81109709
P19_NANOG_CNCC_D5_mem_q10_srt_rmdup_peaks_peak_7451
chr12 81182455 81183165
P19_NANOG_CNCC_D5_mem_q10_srt_rmdup_peaks_peak_7454
chr12 81600977 81601350
P19_NANOG_CNCC_D5_mem_q10_srt_rmdup_peaks_peak_7460
chr12 81795737 81796099
P19_NANOG_CNCC_D5_mem_q10_srt_rmdup_peaks_peak_7462
chr12 83336569 83337033
P19_NANOG_CNCC_D5_mem_q10_srt_rmdup_peaks_peak_7475
chr12 84664196 84664608
P19_NANOG_CNCC_D5_mem_q10_srt_rmdup_peaks_peak_7482
chr12 85984717 85985071
P19_NANOG_CNCC_D5_mem_q10_srt_rmdup_peaks_peak_7493
chr12 86539028 86539355
P19_NANOG_CNCC_D5_mem_q10_srt_rmdup_peaks_peak_7497
chr12 87310949 87311259
P19_NANOG_CNCC_D5_mem_q10_srt_rmdup_peaks_peak_7502
chr12 88972437 88972742
P19_NANOG_CNCC_D5_mem_q10_srt_rmdup_peaks_peak_7513
chr12 89017538 89017847
P19_NANOG_CNCC_D5_mem_q10_srt_rmdup_peaks_peak_7515
chr12 89619508 89620092
P19_NANOG_CNCC_D5_mem_q10_srt_rmdup_peaks_peak_7521
chr12 90042253 90042557
P19_NANOG_CNCC_D5_mem_q10_srt_rmdup_peaks_peak_7530
chr12 92601618 92601998
P19_NANOG_CNCC_D5_mem_q10_srt_rmdup_peaks_peak_7552
chr12 92725214 92725542
P19_NANOG_CNCC_D5_mem_q10_srt_rmdup_peaks_peak_7553
chr12 93032377 93032745
P19_NANOG_CNCC_D5_mem_q10_srt_rmdup_peaks_peak_7559
chr12 93323018 93323289

P19_NANOG_CNCC_D5_mem_q10_srt_rmdup_peaks_peak_7564
chr12 93861405 93861936
P19_NANOG_CNCC_D5_mem_q10_srt_rmdup_peaks_peak_7568
chr12 93967170 93967500
P19_NANOG_CNCC_D5_mem_q10_srt_rmdup_peaks_peak_7571
chr12 94496028 94496345
P19_NANOG_CNCC_D5_mem_q10_srt_rmdup_peaks_peak_7579
chr12 95383219 95383525
P19_NANOG_CNCC_D5_mem_q10_srt_rmdup_peaks_peak_7597
chr12 95588963 95589299
P19_NANOG_CNCC_D5_mem_q10_srt_rmdup_peaks_peak_7601
chr12 95805200 95805730
P19_NANOG_CNCC_D5_mem_q10_srt_rmdup_peaks_peak_7607
chr12 95939278 95939857
P19_NANOG_CNCC_D5_mem_q10_srt_rmdup_peaks_peak_7609
chr12 95968646 95968957
P19_NANOG_CNCC_D5_mem_q10_srt_rmdup_peaks_peak_7614
chr12 97016937 97017300
P19_NANOG_CNCC_D5_mem_q10_srt_rmdup_peaks_peak_7630
chr12 98685698 98686024
P19_NANOG_CNCC_D5_mem_q10_srt_rmdup_peaks_peak_7650
chr12 98902956 98903258
P19_NANOG_CNCC_D5_mem_q10_srt_rmdup_peaks_peak_7656
chr12 98910145 98910533
P19_NANOG_CNCC_D5_mem_q10_srt_rmdup_peaks_peak_7657
chr12 99672719 99672990
P19_NANOG_CNCC_D5_mem_q10_srt_rmdup_peaks_peak_7672
chr12 100163202 100163593
P19_NANOG_CNCC_D5_mem_q10_srt_rmdup_peaks_peak_7677
chr12 101188678 101188987
P19_NANOG_CNCC_D5_mem_q10_srt_rmdup_peaks_peak_7686
chr12 101540023 101540294
P19_NANOG_CNCC_D5_mem_q10_srt_rmdup_peaks_peak_7690
chr12 102548448 102548842
P19_NANOG_CNCC_D5_mem_q10_srt_rmdup_peaks_peak_7696
chr12 104351008 104351520
P19_NANOG_CNCC_D5_mem_q10_srt_rmdup_peaks_peak_7711
chr12 104530756 104531081
P19_NANOG_CNCC_D5_mem_q10_srt_rmdup_peaks_peak_7714
chr12 104697354 104697814
P19_NANOG_CNCC_D5_mem_q10_srt_rmdup_peaks_peak_7719
chr12 105873802 105874246
P19_NANOG_CNCC_D5_mem_q10_srt_rmdup_peaks_peak_7737
chr12 107538841 107539158
P19_NANOG_CNCC_D5_mem_q10_srt_rmdup_peaks_peak_7762
chr12 107714967 107715314
P19_NANOG_CNCC_D5_mem_q10_srt_rmdup_peaks_peak_7763
chr12 108297447 108297763
P19_NANOG_CNCC_D5_mem_q10_srt_rmdup_peaks_peak_7773
chr12 108401492 108401932

P19_NANOG_CNCC_D5_mem_q10_srt_rmdup_peaks_peak_7774
chr12 108576354 108576625
P19_NANOG_CNCC_D5_mem_q10_srt_rmdup_peaks_peak_7779
chr12 108819227 108819702
P19_NANOG_CNCC_D5_mem_q10_srt_rmdup_peaks_peak_7784
chr12 109018992 109019436
P19_NANOG_CNCC_D5_mem_q10_srt_rmdup_peaks_peak_7787
chr12 109221320 109221591
P19_NANOG_CNCC_D5_mem_q10_srt_rmdup_peaks_peak_7795
chr12 111247083 111247519
P19_NANOG_CNCC_D5_mem_q10_srt_rmdup_peaks_peak_7817
chr12 111252806 111253081
P19_NANOG_CNCC_D5_mem_q10_srt_rmdup_peaks_peak_7819
chr12 111264309 111264648
P19_NANOG_CNCC_D5_mem_q10_srt_rmdup_peaks_peak_7821
chr12 111325802 111326148
P19_NANOG_CNCC_D5_mem_q10_srt_rmdup_peaks_peak_7824
chr12 113293169 113293517
P19_NANOG_CNCC_D5_mem_q10_srt_rmdup_peaks_peak_7853
chr12 113590988 113591421
P19_NANOG_CNCC_D5_mem_q10_srt_rmdup_peaks_peak_7857
chr12 113697883 113698242
P19_NANOG_CNCC_D5_mem_q10_srt_rmdup_peaks_peak_7861
chr12 114080867 114081421
P19_NANOG_CNCC_D5_mem_q10_srt_rmdup_peaks_peak_7872
chr12 115103713 115104086
P19_NANOG_CNCC_D5_mem_q10_srt_rmdup_peaks_peak_7904
chr12 115735948 115736230
P19_NANOG_CNCC_D5_mem_q10_srt_rmdup_peaks_peak_7925
chr12 116310696 116311065
P19_NANOG_CNCC_D5_mem_q10_srt_rmdup_peaks_peak_7930
chr12 116770747 116771338
P19_NANOG_CNCC_D5_mem_q10_srt_rmdup_peaks_peak_7940
chr12 119178959 119179325
P19_NANOG_CNCC_D5_mem_q10_srt_rmdup_peaks_peak_7965
chr12 119212189 119212465
P19_NANOG_CNCC_D5_mem_q10_srt_rmdup_peaks_peak_7967
chr12 120378703 120379041
P19_NANOG_CNCC_D5_mem_q10_srt_rmdup_peaks_peak_7982
chr12 121647581 121647862
P19_NANOG_CNCC_D5_mem_q10_srt_rmdup_peaks_peak_8007
chr12 122501962 122502266
P19_NANOG_CNCC_D5_mem_q10_srt_rmdup_peaks_peak_8022
chr12 122503095 122503366
P19_NANOG_CNCC_D5_mem_q10_srt_rmdup_peaks_peak_8023
chr12 122708062 122708333
P19_NANOG_CNCC_D5_mem_q10_srt_rmdup_peaks_peak_8025
chr12 122710515 122711056
P19_NANOG_CNCC_D5_mem_q10_srt_rmdup_peaks_peak_8026
chr12 123365245 123365581

P19_NANOG_CNCC_D5_mem_q10_srt_rmdup_peaks_peak_8035
chr12 125353861 125354279
P19_NANOG_CNCC_D5_mem_q10_srt_rmdup_peaks_peak_8065
chr12 125534091 125534472
P19_NANOG_CNCC_D5_mem_q10_srt_rmdup_peaks_peak_8069
chr12 125591030 125591340
P19_NANOG_CNCC_D5_mem_q10_srt_rmdup_peaks_peak_8071
chr12 131260841 131261291
P19_NANOG_CNCC_D5_mem_q10_srt_rmdup_peaks_peak_8093
chr13 22051316 22051600
P19_NANOG_CNCC_D5_mem_q10_srt_rmdup_peaks_peak_8154
chr13 22307969 22308356
P19_NANOG_CNCC_D5_mem_q10_srt_rmdup_peaks_peak_8156
chr13 23388165 23388959
P19_NANOG_CNCC_D5_mem_q10_srt_rmdup_peaks_peak_8163
chr13 25638545 25638864
P19_NANOG_CNCC_D5_mem_q10_srt_rmdup_peaks_peak_8188
chr13 26606140 26606502
P19_NANOG_CNCC_D5_mem_q10_srt_rmdup_peaks_peak_8202
chr13 27244841 27245233
P19_NANOG_CNCC_D5_mem_q10_srt_rmdup_peaks_peak_8211
chr13 28114631 28114954
P19_NANOG_CNCC_D5_mem_q10_srt_rmdup_peaks_peak_8221
chr13 28177504 28177889
P19_NANOG_CNCC_D5_mem_q10_srt_rmdup_peaks_peak_8222
chr13 28548133 28548491
P19_NANOG_CNCC_D5_mem_q10_srt_rmdup_peaks_peak_8241
chr13 28550923 28551225
P19_NANOG_CNCC_D5_mem_q10_srt_rmdup_peaks_peak_8242
chr13 28597018 28597367
P19_NANOG_CNCC_D5_mem_q10_srt_rmdup_peaks_peak_8244
chr13 28615724 28616085
P19_NANOG_CNCC_D5_mem_q10_srt_rmdup_peaks_peak_8245
chr13 28812075 28812729
P19_NANOG_CNCC_D5_mem_q10_srt_rmdup_peaks_peak_8250
chr13 29866322 29866624
P19_NANOG_CNCC_D5_mem_q10_srt_rmdup_peaks_peak_8260
chr13 30002623 30002897
P19_NANOG_CNCC_D5_mem_q10_srt_rmdup_peaks_peak_8264
chr13 32581626 32581897
P19_NANOG_CNCC_D5_mem_q10_srt_rmdup_peaks_peak_8288
chr13 32656620 32657020
P19_NANOG_CNCC_D5_mem_q10_srt_rmdup_peaks_peak_8292
chr13 33243127 33243517
P19_NANOG_CNCC_D5_mem_q10_srt_rmdup_peaks_peak_8294
chr13 33666532 33666809
P19_NANOG_CNCC_D5_mem_q10_srt_rmdup_peaks_peak_8298
chr13 34116491 34116793
P19_NANOG_CNCC_D5_mem_q10_srt_rmdup_peaks_peak_8303
chr13 34736205 34736480

P19_NANOG_CNCC_D5_mem_q10_srt_rmdup_peaks_peak_8309
chr13 34753623 34753895
P19_NANOG_CNCC_D5_mem_q10_srt_rmdup_peaks_peak_8310
chr13 35517161 35517500
P19_NANOG_CNCC_D5_mem_q10_srt_rmdup_peaks_peak_8321
chr13 36046471 36047029
P19_NANOG_CNCC_D5_mem_q10_srt_rmdup_peaks_peak_8323
chr13 37186371 37186651
P19_NANOG_CNCC_D5_mem_q10_srt_rmdup_peaks_peak_8337
chr13 39609427 39609742
P19_NANOG_CNCC_D5_mem_q10_srt_rmdup_peaks_peak_8359
chr13 39701062 39701477
P19_NANOG_CNCC_D5_mem_q10_srt_rmdup_peaks_peak_8360
chr13 39915525 39915870
P19_NANOG_CNCC_D5_mem_q10_srt_rmdup_peaks_peak_8364
chr13 40084380 40084681
P19_NANOG_CNCC_D5_mem_q10_srt_rmdup_peaks_peak_8372
chr13 40089396 40089922
P19_NANOG_CNCC_D5_mem_q10_srt_rmdup_peaks_peak_8373
chr13 40515321 40515592
P19_NANOG_CNCC_D5_mem_q10_srt_rmdup_peaks_peak_8377
chr13 40630235 40630595
P19_NANOG_CNCC_D5_mem_q10_srt_rmdup_peaks_peak_8379
chr13 40729289 40729771
P19_NANOG_CNCC_D5_mem_q10_srt_rmdup_peaks_peak_8380
chr13 41142549 41142996
P19_NANOG_CNCC_D5_mem_q10_srt_rmdup_peaks_peak_8389
chr13 41222908 41223414
P19_NANOG_CNCC_D5_mem_q10_srt_rmdup_peaks_peak_8390
chr13 42575017 42575312
P19_NANOG_CNCC_D5_mem_q10_srt_rmdup_peaks_peak_8405
chr13 42957127 42957670
P19_NANOG_CNCC_D5_mem_q10_srt_rmdup_peaks_peak_8407
chr13 43447112 43447524
P19_NANOG_CNCC_D5_mem_q10_srt_rmdup_peaks_peak_8411
chr13 43597505 43597920
P19_NANOG_CNCC_D5_mem_q10_srt_rmdup_peaks_peak_8414
chr13 43630752 43631129
P19_NANOG_CNCC_D5_mem_q10_srt_rmdup_peaks_peak_8417
chr13 43652000 43652319
P19_NANOG_CNCC_D5_mem_q10_srt_rmdup_peaks_peak_8420
chr13 44539745 44540016
P19_NANOG_CNCC_D5_mem_q10_srt_rmdup_peaks_peak_8428
chr13 45090920 45091202
P19_NANOG_CNCC_D5_mem_q10_srt_rmdup_peaks_peak_8435
chr13 45626561 45626999
P19_NANOG_CNCC_D5_mem_q10_srt_rmdup_peaks_peak_8442
chr13 48127706 48127977
P19_NANOG_CNCC_D5_mem_q10_srt_rmdup_peaks_peak_8469
chr13 48660382 48660795

P19_NANOG_CNCC_D5_mem_q10_srt_rmdup_peaks_peak_8474
chr13 49447666 49448069
P19_NANOG_CNCC_D5_mem_q10_srt_rmdup_peaks_peak_8483
chr13 50114724 50114998
P19_NANOG_CNCC_D5_mem_q10_srt_rmdup_peaks_peak_8494
chr13 50952542 50952988
P19_NANOG_CNCC_D5_mem_q10_srt_rmdup_peaks_peak_8513
chr13 51720744 51721124
P19_NANOG_CNCC_D5_mem_q10_srt_rmdup_peaks_peak_8519
chr13 52736723 52737206
P19_NANOG_CNCC_D5_mem_q10_srt_rmdup_peaks_peak_8538
chr13 53382674 53382964
P19_NANOG_CNCC_D5_mem_q10_srt_rmdup_peaks_peak_8543
chr13 53473188 53473658
P19_NANOG_CNCC_D5_mem_q10_srt_rmdup_peaks_peak_8549
chr13 53530495 53530766
P19_NANOG_CNCC_D5_mem_q10_srt_rmdup_peaks_peak_8553
chr13 53542245 53542618
P19_NANOG_CNCC_D5_mem_q10_srt_rmdup_peaks_peak_8555
chr13 53812468 53812815
P19_NANOG_CNCC_D5_mem_q10_srt_rmdup_peaks_peak_8558
chr13 54842115 54842686
P19_NANOG_CNCC_D5_mem_q10_srt_rmdup_peaks_peak_8566
chr13 56014713 56015099
P19_NANOG_CNCC_D5_mem_q10_srt_rmdup_peaks_peak_8576
chr13 56145608 56146134
P19_NANOG_CNCC_D5_mem_q10_srt_rmdup_peaks_peak_8577
chr13 56147144 56147415
P19_NANOG_CNCC_D5_mem_q10_srt_rmdup_peaks_peak_8578
chr13 56229119 56229418
P19_NANOG_CNCC_D5_mem_q10_srt_rmdup_peaks_peak_8582
chr13 56234606 56234916
P19_NANOG_CNCC_D5_mem_q10_srt_rmdup_peaks_peak_8583
chr13 58175635 58176048
P19_NANOG_CNCC_D5_mem_q10_srt_rmdup_peaks_peak_8589
chr13 58256354 58256805
P19_NANOG_CNCC_D5_mem_q10_srt_rmdup_peaks_peak_8594
chr13 59050452 59051009
P19_NANOG_CNCC_D5_mem_q10_srt_rmdup_peaks_peak_8600
chr13 59255068 59255425
P19_NANOG_CNCC_D5_mem_q10_srt_rmdup_peaks_peak_8602
chr13 60738665 60738999
P19_NANOG_CNCC_D5_mem_q10_srt_rmdup_peaks_peak_8620
chr13 61270541 61271013
P19_NANOG_CNCC_D5_mem_q10_srt_rmdup_peaks_peak_8627
chr13 61331842 61332235
P19_NANOG_CNCC_D5_mem_q10_srt_rmdup_peaks_peak_8628
chr13 61758106 61758404
P19_NANOG_CNCC_D5_mem_q10_srt_rmdup_peaks_peak_8631
chr13 61932630 61933328

P19_NANOG_CNCC_D5_mem_q10_srt_rmdup_peaks_peak_8633
chr13 61989078 61989498
P19_NANOG_CNCC_D5_mem_q10_srt_rmdup_peaks_peak_8635
chr13 62351381 62351918
P19_NANOG_CNCC_D5_mem_q10_srt_rmdup_peaks_peak_8637
chr13 65432250 65432615
P19_NANOG_CNCC_D5_mem_q10_srt_rmdup_peaks_peak_8655
chr13 66811654 66812230
P19_NANOG_CNCC_D5_mem_q10_srt_rmdup_peaks_peak_8661
chr13 70988384 70988710
P19_NANOG_CNCC_D5_mem_q10_srt_rmdup_peaks_peak_8689
chr13 72257539 72258036
P19_NANOG_CNCC_D5_mem_q10_srt_rmdup_peaks_peak_8695
chr13 73534686 73535000
P19_NANOG_CNCC_D5_mem_q10_srt_rmdup_peaks_peak_8715
chr13 76911456 76911745
P19_NANOG_CNCC_D5_mem_q10_srt_rmdup_peaks_peak_8743
chr13 78323462 78323747
P19_NANOG_CNCC_D5_mem_q10_srt_rmdup_peaks_peak_8754
chr13 80936054 80936382
P19_NANOG_CNCC_D5_mem_q10_srt_rmdup_peaks_peak_8780
chr13 82151778 82152151
P19_NANOG_CNCC_D5_mem_q10_srt_rmdup_peaks_peak_8790
chr13 84413252 84413592
P19_NANOG_CNCC_D5_mem_q10_srt_rmdup_peaks_peak_8799
chr13 84889843 84890131
P19_NANOG_CNCC_D5_mem_q10_srt_rmdup_peaks_peak_8802
chr13 86736215 86736713
P19_NANOG_CNCC_D5_mem_q10_srt_rmdup_peaks_peak_8809
chr13 95039420 95039871
P19_NANOG_CNCC_D5_mem_q10_srt_rmdup_peaks_peak_8865
chr13 95636256 95636586
P19_NANOG_CNCC_D5_mem_q10_srt_rmdup_peaks_peak_8878
chr13 95693501 95693820
P19_NANOG_CNCC_D5_mem_q10_srt_rmdup_peaks_peak_8881
chr13 96152441 96152716
P19_NANOG_CNCC_D5_mem_q10_srt_rmdup_peaks_peak_8888
chr13 98926532 98926826
P19_NANOG_CNCC_D5_mem_q10_srt_rmdup_peaks_peak_8910
chr13 98943974 98944402
P19_NANOG_CNCC_D5_mem_q10_srt_rmdup_peaks_peak_8911
chr13 99611676 99612059
P19_NANOG_CNCC_D5_mem_q10_srt_rmdup_peaks_peak_8929
chr13 100383835 100384353
P19_NANOG_CNCC_D5_mem_q10_srt_rmdup_peaks_peak_8948
chr13 100396907 100397240
P19_NANOG_CNCC_D5_mem_q10_srt_rmdup_peaks_peak_8949
chr13 100588058 100588337
P19_NANOG_CNCC_D5_mem_q10_srt_rmdup_peaks_peak_8955
chr13 100636033 100636412

P19_NANOG_CNCC_D5_mem_q10_srt_rmdup_peaks_peak_8963
chr13 100639103 100639508
P19_NANOG_CNCC_D5_mem_q10_srt_rmdup_peaks_peak_8964
chr13 101506181 101506547
P19_NANOG_CNCC_D5_mem_q10_srt_rmdup_peaks_peak_8981
chr13 103413471 103413804
P19_NANOG_CNCC_D5_mem_q10_srt_rmdup_peaks_peak_8988
chr13 103451157 103451558
P19_NANOG_CNCC_D5_mem_q10_srt_rmdup_peaks_peak_8990
chr13 104204619 104205022
P19_NANOG_CNCC_D5_mem_q10_srt_rmdup_peaks_peak_8997
chr13 107180171 107180514
P19_NANOG_CNCC_D5_mem_q10_srt_rmdup_peaks_peak_9015
chr13 107188130 107188401
P19_NANOG_CNCC_D5_mem_q10_srt_rmdup_peaks_peak_9019
chr13 107948810 107949081
P19_NANOG_CNCC_D5_mem_q10_srt_rmdup_peaks_peak_9028
chr13 108214671 108214991
P19_NANOG_CNCC_D5_mem_q10_srt_rmdup_peaks_peak_9029
chr13 108484825 108485225
P19_NANOG_CNCC_D5_mem_q10_srt_rmdup_peaks_peak_9033
chr13 110556636 110556930
P19_NANOG_CNCC_D5_mem_q10_srt_rmdup_peaks_peak_9042
chr13 110726267 110726669
P19_NANOG_CNCC_D5_mem_q10_srt_rmdup_peaks_peak_9044
chr13 110994588 110994933
P19_NANOG_CNCC_D5_mem_q10_srt_rmdup_peaks_peak_9050
chr13 111138463 111139158
P19_NANOG_CNCC_D5_mem_q10_srt_rmdup_peaks_peak_9054
chr13 111365776 111366349
P19_NANOG_CNCC_D5_mem_q10_srt_rmdup_peaks_peak_9058
chr13 112708293 112708658
P19_NANOG_CNCC_D5_mem_q10_srt_rmdup_peaks_peak_9082
chr13 113596945 113597241
P19_NANOG_CNCC_D5_mem_q10_srt_rmdup_peaks_peak_9107
chr14 21131263 21131588
P19_NANOG_CNCC_D5_mem_q10_srt_rmdup_peaks_peak_9130
chr14 21252028 21252308
P19_NANOG_CNCC_D5_mem_q10_srt_rmdup_peaks_peak_9139
chr14 21996903 21997307
P19_NANOG_CNCC_D5_mem_q10_srt_rmdup_peaks_peak_9152
chr14 24013693 24014103
P19_NANOG_CNCC_D5_mem_q10_srt_rmdup_peaks_peak_9183
chr14 24740653 24740924
P19_NANOG_CNCC_D5_mem_q10_srt_rmdup_peaks_peak_9198
chr14 25309479 25309982
P19_NANOG_CNCC_D5_mem_q10_srt_rmdup_peaks_peak_9202
chr14 25407855 25408150
P19_NANOG_CNCC_D5_mem_q10_srt_rmdup_peaks_peak_9205
chr14 25427819 25428334

P19_NANOG_CNCC_D5_mem_q10_srt_rmdup_peaks_peak_9208
chr14 25560107 25560378
P19_NANOG_CNCC_D5_mem_q10_srt_rmdup_peaks_peak_9209
chr14 26607922 26608271
P19_NANOG_CNCC_D5_mem_q10_srt_rmdup_peaks_peak_9215
chr14 26863662 26864063
P19_NANOG_CNCC_D5_mem_q10_srt_rmdup_peaks_peak_9217
chr14 28158096 28158638
P19_NANOG_CNCC_D5_mem_q10_srt_rmdup_peaks_peak_9223
chr14 28425337 28425706
P19_NANOG_CNCC_D5_mem_q10_srt_rmdup_peaks_peak_9225
chr14 29244576 29245041
P19_NANOG_CNCC_D5_mem_q10_srt_rmdup_peaks_peak_9238
chr14 29859381 29860061
P19_NANOG_CNCC_D5_mem_q10_srt_rmdup_peaks_peak_9242
chr14 30001278 30001782
P19_NANOG_CNCC_D5_mem_q10_srt_rmdup_peaks_peak_9244
chr14 30118137 30118622
P19_NANOG_CNCC_D5_mem_q10_srt_rmdup_peaks_peak_9245
chr14 30739821 30740121
P19_NANOG_CNCC_D5_mem_q10_srt_rmdup_peaks_peak_9253
chr14 32687225 32687516
P19_NANOG_CNCC_D5_mem_q10_srt_rmdup_peaks_peak_9271
chr14 33723628 33723899
P19_NANOG_CNCC_D5_mem_q10_srt_rmdup_peaks_peak_9280
chr14 34844516 34844850
P19_NANOG_CNCC_D5_mem_q10_srt_rmdup_peaks_peak_9297
chr14 34930837 34931181
P19_NANOG_CNCC_D5_mem_q10_srt_rmdup_peaks_peak_9298
chr14 35307061 35307349
P19_NANOG_CNCC_D5_mem_q10_srt_rmdup_peaks_peak_9299
chr14 35939998 35940269
P19_NANOG_CNCC_D5_mem_q10_srt_rmdup_peaks_peak_9306
chr14 36489356 36489721
P19_NANOG_CNCC_D5_mem_q10_srt_rmdup_peaks_peak_9310
chr14 37274430 37274810
P19_NANOG_CNCC_D5_mem_q10_srt_rmdup_peaks_peak_9336
chr14 37872839 37873221
P19_NANOG_CNCC_D5_mem_q10_srt_rmdup_peaks_peak_9341
chr14 38668382 38668708
P19_NANOG_CNCC_D5_mem_q10_srt_rmdup_peaks_peak_9353
chr14 38691247 38691617
P19_NANOG_CNCC_D5_mem_q10_srt_rmdup_peaks_peak_9354
chr14 39583278 39583620
P19_NANOG_CNCC_D5_mem_q10_srt_rmdup_peaks_peak_9360
chr14 39837201 39837848
P19_NANOG_CNCC_D5_mem_q10_srt_rmdup_peaks_peak_9362
chr14 40523123 40523408
P19_NANOG_CNCC_D5_mem_q10_srt_rmdup_peaks_peak_9365
chr14 40832166 40832478

P19_NANOG_CNCC_D5_mem_q10_srt_rmdup_peaks_peak_9369
chr14 42977496 42977891
P19_NANOG_CNCC_D5_mem_q10_srt_rmdup_peaks_peak_9380
chr14 44418621 44419019
P19_NANOG_CNCC_D5_mem_q10_srt_rmdup_peaks_peak_9386
chr14 48444794 48445119
P19_NANOG_CNCC_D5_mem_q10_srt_rmdup_peaks_peak_9402
chr14 48737161 48737597
P19_NANOG_CNCC_D5_mem_q10_srt_rmdup_peaks_peak_9404
chr14 49935201 49935674
P19_NANOG_CNCC_D5_mem_q10_srt_rmdup_peaks_peak_9408
chr14 49973570 49973913
P19_NANOG_CNCC_D5_mem_q10_srt_rmdup_peaks_peak_9409
chr14 50075192 50075523
P19_NANOG_CNCC_D5_mem_q10_srt_rmdup_peaks_peak_9412
chr14 50235014 50235397
P19_NANOG_CNCC_D5_mem_q10_srt_rmdup_peaks_peak_9416
chr14 50419938 50420318
P19_NANOG_CNCC_D5_mem_q10_srt_rmdup_peaks_peak_9420
chr14 51377511 51377782
P19_NANOG_CNCC_D5_mem_q10_srt_rmdup_peaks_peak_9432
chr14 51605099 51605520
P19_NANOG_CNCC_D5_mem_q10_srt_rmdup_peaks_peak_9435
chr14 52598440 52598744
P19_NANOG_CNCC_D5_mem_q10_srt_rmdup_peaks_peak_9446
chr14 53256991 53257300
P19_NANOG_CNCC_D5_mem_q10_srt_rmdup_peaks_peak_9450
chr14 53426429 53426932
P19_NANOG_CNCC_D5_mem_q10_srt_rmdup_peaks_peak_9453
chr14 53948294 53948590
P19_NANOG_CNCC_D5_mem_q10_srt_rmdup_peaks_peak_9461
chr14 54297991 54298317
P19_NANOG_CNCC_D5_mem_q10_srt_rmdup_peaks_peak_9468
chr14 54387833 54388189
P19_NANOG_CNCC_D5_mem_q10_srt_rmdup_peaks_peak_9470
chr14 54757968 54758329
P19_NANOG_CNCC_D5_mem_q10_srt_rmdup_peaks_peak_9477
chr14 55369866 55370168
P19_NANOG_CNCC_D5_mem_q10_srt_rmdup_peaks_peak_9487
chr14 55658049 55658320
P19_NANOG_CNCC_D5_mem_q10_srt_rmdup_peaks_peak_9490
chr14 56058016 56058462
P19_NANOG_CNCC_D5_mem_q10_srt_rmdup_peaks_peak_9494
chr14 56331362 56331688
P19_NANOG_CNCC_D5_mem_q10_srt_rmdup_peaks_peak_9499
chr14 56348242 56348772
P19_NANOG_CNCC_D5_mem_q10_srt_rmdup_peaks_peak_9500
chr14 56599253 56599601
P19_NANOG_CNCC_D5_mem_q10_srt_rmdup_peaks_peak_9506
chr14 56639148 56639612

P19_NANOG_CNCC_D5_mem_q10_srt_rmdup_peaks_peak_9507
chr14 57311156 57311495
P19_NANOG_CNCC_D5_mem_q10_srt_rmdup_peaks_peak_9518
chr14 57354922 57355368
P19_NANOG_CNCC_D5_mem_q10_srt_rmdup_peaks_peak_9521
chr14 57465372 57465813
P19_NANOG_CNCC_D5_mem_q10_srt_rmdup_peaks_peak_9529
chr14 58428438 58428729
P19_NANOG_CNCC_D5_mem_q10_srt_rmdup_peaks_peak_9539
chr14 59073844 59074386
P19_NANOG_CNCC_D5_mem_q10_srt_rmdup_peaks_peak_9547
chr14 59093147 59093589
P19_NANOG_CNCC_D5_mem_q10_srt_rmdup_peaks_peak_9549
chr14 59495692 59496027
P19_NANOG_CNCC_D5_mem_q10_srt_rmdup_peaks_peak_9558
chr14 59751319 59751621
P19_NANOG_CNCC_D5_mem_q10_srt_rmdup_peaks_peak_9560
chr14 60627985 60628435
P19_NANOG_CNCC_D5_mem_q10_srt_rmdup_peaks_peak_9568
chr14 60798584 60798901
P19_NANOG_CNCC_D5_mem_q10_srt_rmdup_peaks_peak_9574
chr14 63362775 63363053
P19_NANOG_CNCC_D5_mem_q10_srt_rmdup_peaks_peak_9618
chr14 64233299 64233755
P19_NANOG_CNCC_D5_mem_q10_srt_rmdup_peaks_peak_9622
chr14 64320143 64320414
P19_NANOG_CNCC_D5_mem_q10_srt_rmdup_peaks_peak_9624
chr14 64872092 64872558
P19_NANOG_CNCC_D5_mem_q10_srt_rmdup_peaks_peak_9636
chr14 64970221 64970711
P19_NANOG_CNCC_D5_mem_q10_srt_rmdup_peaks_peak_9637
chr14 64971591 64971864
P19_NANOG_CNCC_D5_mem_q10_srt_rmdup_peaks_peak_9638
chr14 65192236 65192619
P19_NANOG_CNCC_D5_mem_q10_srt_rmdup_peaks_peak_9645
chr14 65945270 65945795
P19_NANOG_CNCC_D5_mem_q10_srt_rmdup_peaks_peak_9658
chr14 66423611 66423954
P19_NANOG_CNCC_D5_mem_q10_srt_rmdup_peaks_peak_9667
chr14 66850524 66850836
P19_NANOG_CNCC_D5_mem_q10_srt_rmdup_peaks_peak_9674
chr14 68005876 68006151
P19_NANOG_CNCC_D5_mem_q10_srt_rmdup_peaks_peak_9683
chr14 68141275 68141561
P19_NANOG_CNCC_D5_mem_q10_srt_rmdup_peaks_peak_9686
chr14 68332492 68332856
P19_NANOG_CNCC_D5_mem_q10_srt_rmdup_peaks_peak_9690
chr14 68404756 68405092
P19_NANOG_CNCC_D5_mem_q10_srt_rmdup_peaks_peak_9692
chr14 68541703 68541974

P19_NANOG_CNCC_D5_mem_q10_srt_rmdup_peaks_peak_9694
chr14 68571350 68571621
P19_NANOG_CNCC_D5_mem_q10_srt_rmdup_peaks_peak_9696
chr14 68710313 68710625
P19_NANOG_CNCC_D5_mem_q10_srt_rmdup_peaks_peak_9704
chr14 68818474 68818799
P19_NANOG_CNCC_D5_mem_q10_srt_rmdup_peaks_peak_9707
chr14 68944422 68944732
P19_NANOG_CNCC_D5_mem_q10_srt_rmdup_peaks_peak_9713
chr14 69112941 69113299
P19_NANOG_CNCC_D5_mem_q10_srt_rmdup_peaks_peak_9718
chr14 69504544 69504925
P19_NANOG_CNCC_D5_mem_q10_srt_rmdup_peaks_peak_9729
chr14 69560512 69560878
P19_NANOG_CNCC_D5_mem_q10_srt_rmdup_peaks_peak_9733
chr14 70162709 70163125
P19_NANOG_CNCC_D5_mem_q10_srt_rmdup_peaks_peak_9741
chr14 70759347 70759631
P19_NANOG_CNCC_D5_mem_q10_srt_rmdup_peaks_peak_9749
chr14 73494611 73494950
P19_NANOG_CNCC_D5_mem_q10_srt_rmdup_peaks_peak_9777
chr14 73973832 73974237
P19_NANOG_CNCC_D5_mem_q10_srt_rmdup_peaks_peak_9790
chr14 75619972 75620314
P19_NANOG_CNCC_D5_mem_q10_srt_rmdup_peaks_peak_9811
chr14 75735644 75736222
P19_NANOG_CNCC_D5_mem_q10_srt_rmdup_peaks_peak_9815
chr14 75981489 75981966
P19_NANOG_CNCC_D5_mem_q10_srt_rmdup_peaks_peak_9823
chr14 76120537 76120818
P19_NANOG_CNCC_D5_mem_q10_srt_rmdup_peaks_peak_9825
chr14 77111150 77111578
P19_NANOG_CNCC_D5_mem_q10_srt_rmdup_peaks_peak_9844
chr14 77571536 77572122
P19_NANOG_CNCC_D5_mem_q10_srt_rmdup_peaks_peak_9856
chr14 77782363 77782679
P19_NANOG_CNCC_D5_mem_q10_srt_rmdup_peaks_peak_9859
chr14 77938712 77939180
P19_NANOG_CNCC_D5_mem_q10_srt_rmdup_peaks_peak_9863
chr14 79105361 79105941
P19_NANOG_CNCC_D5_mem_q10_srt_rmdup_peaks_peak_9872
chr14 79950470 79950772
P19_NANOG_CNCC_D5_mem_q10_srt_rmdup_peaks_peak_9893
chr14 79982289 79982663
P19_NANOG_CNCC_D5_mem_q10_srt_rmdup_peaks_peak_9895
chr14 80262302 80262669
P19_NANOG_CNCC_D5_mem_q10_srt_rmdup_peaks_peak_9897
chr14 81391101 81391498
P19_NANOG_CNCC_D5_mem_q10_srt_rmdup_peaks_peak_9903
chr14 81484344 81484738

P19_NANOG_CNCC_D5_mem_q10_srt_rmdup_peaks_peak_9904
chr14 82079960 82080260
P19_NANOG_CNCC_D5_mem_q10_srt_rmdup_peaks_peak_9914
chr14 84172598 84173341
P19_NANOG_CNCC_D5_mem_q10_srt_rmdup_peaks_peak_9930
chr14 84902314 84902658
P19_NANOG_CNCC_D5_mem_q10_srt_rmdup_peaks_peak_9935
chr14 86041830 86042203
P19_NANOG_CNCC_D5_mem_q10_srt_rmdup_peaks_peak_9945
chr14 86342103 86342685
P19_NANOG_CNCC_D5_mem_q10_srt_rmdup_peaks_peak_9947
chr14 88559292 88559799
P19_NANOG_CNCC_D5_mem_q10_srt_rmdup_peaks_peak_9960
chr14 89653209 89653540
P19_NANOG_CNCC_D5_mem_q10_srt_rmdup_peaks_peak_9971
chr14 90422167 90422643
P19_NANOG_CNCC_D5_mem_q10_srt_rmdup_peaks_peak_9985
chr14 90625472 90625926
P19_NANOG_CNCC_D5_mem_q10_srt_rmdup_peaks_peak_9987
chr14 91341376 91341667
P19_NANOG_CNCC_D5_mem_q10_srt_rmdup_peaks_peak_9996
chr14 91879953 91880228
P19_NANOG_CNCC_D5_mem_q10_srt_rmdup_peaks_peak_10005
chr14 92161016 92161287
P19_NANOG_CNCC_D5_mem_q10_srt_rmdup_peaks_peak_10010
chr14 92566159 92566602
P19_NANOG_CNCC_D5_mem_q10_srt_rmdup_peaks_peak_10015
chr14 92953884 92954179
P19_NANOG_CNCC_D5_mem_q10_srt_rmdup_peaks_peak_10020
chr14 94984110 94984423
P19_NANOG_CNCC_D5_mem_q10_srt_rmdup_peaks_peak_10042
chr14 95446483 95446887
P19_NANOG_CNCC_D5_mem_q10_srt_rmdup_peaks_peak_10053
chr14 96445984 96446378
P19_NANOG_CNCC_D5_mem_q10_srt_rmdup_peaks_peak_10073
chr14 96448391 96448662
P19_NANOG_CNCC_D5_mem_q10_srt_rmdup_peaks_peak_10075
chr14 96748531 96749141
P19_NANOG_CNCC_D5_mem_q10_srt_rmdup_peaks_peak_10079
chr14 98097914 98098315
P19_NANOG_CNCC_D5_mem_q10_srt_rmdup_peaks_peak_10098
chr14 98152295 98152566
P19_NANOG_CNCC_D5_mem_q10_srt_rmdup_peaks_peak_10099
chr14 98314829 98315241
P19_NANOG_CNCC_D5_mem_q10_srt_rmdup_peaks_peak_10101
chr14 98699837 98700427
P19_NANOG_CNCC_D5_mem_q10_srt_rmdup_peaks_peak_10106
chr14 99429944 99430428
P19_NANOG_CNCC_D5_mem_q10_srt_rmdup_peaks_peak_10112
chr14 100196866 100197163

P19_NANOG_CNCC_D5_mem_q10_srt_rmdup_peaks_peak_10128
chr14 100621446 100621774
P19_NANOG_CNCC_D5_mem_q10_srt_rmdup_peaks_peak_10139
chr14 100687863 100688227
P19_NANOG_CNCC_D5_mem_q10_srt_rmdup_peaks_peak_10141
chr14 101266491 101266887
P19_NANOG_CNCC_D5_mem_q10_srt_rmdup_peaks_peak_10148
chr14 101800577 101801173
P19_NANOG_CNCC_D5_mem_q10_srt_rmdup_peaks_peak_10155
chr14 102729247 102729558
P19_NANOG_CNCC_D5_mem_q10_srt_rmdup_peaks_peak_10168
chr14 103092254 103092699
P19_NANOG_CNCC_D5_mem_q10_srt_rmdup_peaks_peak_10177
chr14 105148444 105148864
P19_NANOG_CNCC_D5_mem_q10_srt_rmdup_peaks_peak_10198
chr14 105310200 105310471
P19_NANOG_CNCC_D5_mem_q10_srt_rmdup_peaks_peak_10201
chr14 105748152 105748636
P19_NANOG_CNCC_D5_mem_q10_srt_rmdup_peaks_peak_10205
chr15 25164700 25165023
P19_NANOG_CNCC_D5_mem_q10_srt_rmdup_peaks_peak_10223
chr15 25850323 25850766
P19_NANOG_CNCC_D5_mem_q10_srt_rmdup_peaks_peak_10229
chr15 26327442 26327887
P19_NANOG_CNCC_D5_mem_q10_srt_rmdup_peaks_peak_10234
chr15 26450788 26451160
P19_NANOG_CNCC_D5_mem_q10_srt_rmdup_peaks_peak_10240
chr15 29247553 29247995
P19_NANOG_CNCC_D5_mem_q10_srt_rmdup_peaks_peak_10258
chr15 29407892 29408198
P19_NANOG_CNCC_D5_mem_q10_srt_rmdup_peaks_peak_10260
chr15 29656644 29656950
P19_NANOG_CNCC_D5_mem_q10_srt_rmdup_peaks_peak_10262
chr15 29863887 29864176
P19_NANOG_CNCC_D5_mem_q10_srt_rmdup_peaks_peak_10264
chr15 30227767 30228124
P19_NANOG_CNCC_D5_mem_q10_srt_rmdup_peaks_peak_10269
chr15 30244425 30244852
P19_NANOG_CNCC_D5_mem_q10_srt_rmdup_peaks_peak_10270
chr15 31251378 31251842
P19_NANOG_CNCC_D5_mem_q10_srt_rmdup_peaks_peak_10275
chr15 31705026 31705470
P19_NANOG_CNCC_D5_mem_q10_srt_rmdup_peaks_peak_10284
chr15 32962436 32962871
P19_NANOG_CNCC_D5_mem_q10_srt_rmdup_peaks_peak_10291
chr15 33073908 33074451
P19_NANOG_CNCC_D5_mem_q10_srt_rmdup_peaks_peak_10293
chr15 33494648 33494931
P19_NANOG_CNCC_D5_mem_q10_srt_rmdup_peaks_peak_10300
chr15 33620390 33620747

P19_NANOG_CNCC_D5_mem_q10_srt_rmdup_peaks_peak_10301
chr15 34152510 34152835
P19_NANOG_CNCC_D5_mem_q10_srt_rmdup_peaks_peak_10311
chr15 34286190 34286499
P19_NANOG_CNCC_D5_mem_q10_srt_rmdup_peaks_peak_10312
chr15 34635655 34635926
P19_NANOG_CNCC_D5_mem_q10_srt_rmdup_peaks_peak_10315
chr15 34729920 34730191
P19_NANOG_CNCC_D5_mem_q10_srt_rmdup_peaks_peak_10317
chr15 36014729 36015132
P19_NANOG_CNCC_D5_mem_q10_srt_rmdup_peaks_peak_10330
chr15 36077078 36077349
P19_NANOG_CNCC_D5_mem_q10_srt_rmdup_peaks_peak_10331
chr15 36871416 36871835
P19_NANOG_CNCC_D5_mem_q10_srt_rmdup_peaks_peak_10344
chr15 37428406 37428686
P19_NANOG_CNCC_D5_mem_q10_srt_rmdup_peaks_peak_10366
chr15 39461779 39462060
P19_NANOG_CNCC_D5_mem_q10_srt_rmdup_peaks_peak_10383
chr15 40185196 40185528
P19_NANOG_CNCC_D5_mem_q10_srt_rmdup_peaks_peak_10395
chr15 40268549 40268912
P19_NANOG_CNCC_D5_mem_q10_srt_rmdup_peaks_peak_10398
chr15 40417039 40417470
P19_NANOG_CNCC_D5_mem_q10_srt_rmdup_peaks_peak_10405
chr15 40417892 40418171
P19_NANOG_CNCC_D5_mem_q10_srt_rmdup_peaks_peak_10406
chr15 41092055 41092448
P19_NANOG_CNCC_D5_mem_q10_srt_rmdup_peaks_peak_10422
chr15 41203279 41203650
P19_NANOG_CNCC_D5_mem_q10_srt_rmdup_peaks_peak_10424
chr15 41220666 41221087
P19_NANOG_CNCC_D5_mem_q10_srt_rmdup_peaks_peak_10426
chr15 41234216 41234487
P19_NANOG_CNCC_D5_mem_q10_srt_rmdup_peaks_peak_10428
chr15 41472319 41472887
P19_NANOG_CNCC_D5_mem_q10_srt_rmdup_peaks_peak_10434
chr15 41873524 41873795
P19_NANOG_CNCC_D5_mem_q10_srt_rmdup_peaks_peak_10443
chr15 42958093 42958497
P19_NANOG_CNCC_D5_mem_q10_srt_rmdup_peaks_peak_10457
chr15 43585562 43585833
P19_NANOG_CNCC_D5_mem_q10_srt_rmdup_peaks_peak_10460
chr15 44084208 44084479
P19_NANOG_CNCC_D5_mem_q10_srt_rmdup_peaks_peak_10464
chr15 44116617 44117063
P19_NANOG_CNCC_D5_mem_q10_srt_rmdup_peaks_peak_10466
chr15 44251066 44251552
P19_NANOG_CNCC_D5_mem_q10_srt_rmdup_peaks_peak_10468
chr15 44331032 44331444

P19_NANOG_CNCC_D5_mem_q10_srt_rmdup_peaks_peak_10469
chr15 44553145 44553522
P19_NANOG_CNCC_D5_mem_q10_srt_rmdup_peaks_peak_10471
chr15 45492415 45492808
P19_NANOG_CNCC_D5_mem_q10_srt_rmdup_peaks_peak_10481
chr15 45497183 45497575
P19_NANOG_CNCC_D5_mem_q10_srt_rmdup_peaks_peak_10482
chr15 46463307 46463869
P19_NANOG_CNCC_D5_mem_q10_srt_rmdup_peaks_peak_10488
chr15 46589236 46589797
P19_NANOG_CNCC_D5_mem_q10_srt_rmdup_peaks_peak_10490
chr15 47874448 47874751
P19_NANOG_CNCC_D5_mem_q10_srt_rmdup_peaks_peak_10500
chr15 48260597 48261014
P19_NANOG_CNCC_D5_mem_q10_srt_rmdup_peaks_peak_10510
chr15 48904801 48905384
P19_NANOG_CNCC_D5_mem_q10_srt_rmdup_peaks_peak_10520
chr15 49098457 49098728
P19_NANOG_CNCC_D5_mem_q10_srt_rmdup_peaks_peak_10522
chr15 49246642 49246927
P19_NANOG_CNCC_D5_mem_q10_srt_rmdup_peaks_peak_10525
chr15 49338435 49339102
P19_NANOG_CNCC_D5_mem_q10_srt_rmdup_peaks_peak_10527
chr15 49402322 49402630
P19_NANOG_CNCC_D5_mem_q10_srt_rmdup_peaks_peak_10528
chr15 49447695 49448046
P19_NANOG_CNCC_D5_mem_q10_srt_rmdup_peaks_peak_10529
chr15 49517901 49518251
P19_NANOG_CNCC_D5_mem_q10_srt_rmdup_peaks_peak_10530
chr15 49607683 49608015
P19_NANOG_CNCC_D5_mem_q10_srt_rmdup_peaks_peak_10532
chr15 49961621 49961892
P19_NANOG_CNCC_D5_mem_q10_srt_rmdup_peaks_peak_10538
chr15 50646827 50647098
P19_NANOG_CNCC_D5_mem_q10_srt_rmdup_peaks_peak_10545
chr15 51455725 51456073
P19_NANOG_CNCC_D5_mem_q10_srt_rmdup_peaks_peak_10550
chr15 51605284 51605571
P19_NANOG_CNCC_D5_mem_q10_srt_rmdup_peaks_peak_10551
chr15 51655708 51656041
P19_NANOG_CNCC_D5_mem_q10_srt_rmdup_peaks_peak_10552
chr15 52156788 52157301
P19_NANOG_CNCC_D5_mem_q10_srt_rmdup_peaks_peak_10561
chr15 52550587 52550882
P19_NANOG_CNCC_D5_mem_q10_srt_rmdup_peaks_peak_10563
chr15 53075564 53075909
P19_NANOG_CNCC_D5_mem_q10_srt_rmdup_peaks_peak_10572
chr15 55616318 55616628
P19_NANOG_CNCC_D5_mem_q10_srt_rmdup_peaks_peak_10595
chr15 56035488 56035906

P19_NANOG_CNCC_D5_mem_q10_srt_rmdup_peaks_peak_10607
chr15 56091631 56091908
P19_NANOG_CNCC_D5_mem_q10_srt_rmdup_peaks_peak_10610
chr15 56285611 56285882
P19_NANOG_CNCC_D5_mem_q10_srt_rmdup_peaks_peak_10611
chr15 56657529 56657922
P19_NANOG_CNCC_D5_mem_q10_srt_rmdup_peaks_peak_10615
chr15 57578683 57578999
P19_NANOG_CNCC_D5_mem_q10_srt_rmdup_peaks_peak_10630
chr15 57694827 57695182
P19_NANOG_CNCC_D5_mem_q10_srt_rmdup_peaks_peak_10635
chr15 57704262 57704535
P19_NANOG_CNCC_D5_mem_q10_srt_rmdup_peaks_peak_10636
chr15 59639182 59639530
P19_NANOG_CNCC_D5_mem_q10_srt_rmdup_peaks_peak_10661
chr15 60095834 60096204
P19_NANOG_CNCC_D5_mem_q10_srt_rmdup_peaks_peak_10668
chr15 60851740 60852079
P19_NANOG_CNCC_D5_mem_q10_srt_rmdup_peaks_peak_10690
chr15 60953522 60953816
P19_NANOG_CNCC_D5_mem_q10_srt_rmdup_peaks_peak_10697
chr15 61019585 61019886
P19_NANOG_CNCC_D5_mem_q10_srt_rmdup_peaks_peak_10699
chr15 62140117 62140388
P19_NANOG_CNCC_D5_mem_q10_srt_rmdup_peaks_peak_10711
chr15 62589004 62589336
P19_NANOG_CNCC_D5_mem_q10_srt_rmdup_peaks_peak_10718
chr15 63776117 63776388
P19_NANOG_CNCC_D5_mem_q10_srt_rmdup_peaks_peak_10743
chr15 63797523 63797856
P19_NANOG_CNCC_D5_mem_q10_srt_rmdup_peaks_peak_10746
chr15 64260924 64261325
P19_NANOG_CNCC_D5_mem_q10_srt_rmdup_peaks_peak_10757
chr15 64782931 64783623
P19_NANOG_CNCC_D5_mem_q10_srt_rmdup_peaks_peak_10764
chr15 64807477 64807911
P19_NANOG_CNCC_D5_mem_q10_srt_rmdup_peaks_peak_10765
chr15 66808257 66808677
P19_NANOG_CNCC_D5_mem_q10_srt_rmdup_peaks_peak_10813
chr15 67695736 67696161
P19_NANOG_CNCC_D5_mem_q10_srt_rmdup_peaks_peak_10834
chr15 67794404 67794767
P19_NANOG_CNCC_D5_mem_q10_srt_rmdup_peaks_peak_10837
chr15 67813393 67813665
P19_NANOG_CNCC_D5_mem_q10_srt_rmdup_peaks_peak_10838
chr15 67813843 67814157
P19_NANOG_CNCC_D5_mem_q10_srt_rmdup_peaks_peak_10839
chr15 67856299 67857083
P19_NANOG_CNCC_D5_mem_q10_srt_rmdup_peaks_peak_10843
chr15 67999881 68000152

P19_NANOG_CNCC_D5_mem_q10_srt_rmdup_peaks_peak_10845
chr15 68320594 68320880
P19_NANOG_CNCC_D5_mem_q10_srt_rmdup_peaks_peak_10856
chr15 68573392 68573701
P19_NANOG_CNCC_D5_mem_q10_srt_rmdup_peaks_peak_10863
chr15 68871753 68872041
P19_NANOG_CNCC_D5_mem_q10_srt_rmdup_peaks_peak_10868
chr15 69836087 69836587
P19_NANOG_CNCC_D5_mem_q10_srt_rmdup_peaks_peak_10894
chr15 70154162 70154697
P19_NANOG_CNCC_D5_mem_q10_srt_rmdup_peaks_peak_10899
chr15 70488960 70489250
P19_NANOG_CNCC_D5_mem_q10_srt_rmdup_peaks_peak_10909
chr15 71940766 71941109
P19_NANOG_CNCC_D5_mem_q10_srt_rmdup_peaks_peak_10936
chr15 71943955 71944392
P19_NANOG_CNCC_D5_mem_q10_srt_rmdup_peaks_peak_10937
chr15 73655323 73655596
P19_NANOG_CNCC_D5_mem_q10_srt_rmdup_peaks_peak_10950
chr15 74988316 74988590
P19_NANOG_CNCC_D5_mem_q10_srt_rmdup_peaks_peak_10974
chr15 75742583 75742908
P19_NANOG_CNCC_D5_mem_q10_srt_rmdup_peaks_peak_10986
chr15 76286437 76286809
P19_NANOG_CNCC_D5_mem_q10_srt_rmdup_peaks_peak_10993
chr15 76327944 76328311
P19_NANOG_CNCC_D5_mem_q10_srt_rmdup_peaks_peak_10994
chr15 76356640 76357053
P19_NANOG_CNCC_D5_mem_q10_srt_rmdup_peaks_peak_10995
chr15 76443951 76444324
P19_NANOG_CNCC_D5_mem_q10_srt_rmdup_peaks_peak_10999
chr15 76732115 76732386
P19_NANOG_CNCC_D5_mem_q10_srt_rmdup_peaks_peak_11006
chr15 77095384 77095674
P19_NANOG_CNCC_D5_mem_q10_srt_rmdup_peaks_peak_11009
chr15 77766733 77767203
P19_NANOG_CNCC_D5_mem_q10_srt_rmdup_peaks_peak_11019
chr15 77840682 77841223
P19_NANOG_CNCC_D5_mem_q10_srt_rmdup_peaks_peak_11025
chr15 79505097 79505681
P19_NANOG_CNCC_D5_mem_q10_srt_rmdup_peaks_peak_11053
chr15 80905498 80905814
P19_NANOG_CNCC_D5_mem_q10_srt_rmdup_peaks_peak_11069
chr15 82123591 82124102
P19_NANOG_CNCC_D5_mem_q10_srt_rmdup_peaks_peak_11077
chr15 82200548 82200876
P19_NANOG_CNCC_D5_mem_q10_srt_rmdup_peaks_peak_11078
chr15 82233756 82234220
P19_NANOG_CNCC_D5_mem_q10_srt_rmdup_peaks_peak_11079
chr15 83632246 83632521

P19_NANOG_CNCC_D5_mem_q10_srt_rmdup_peaks_peak_11087
chr15	83747545	83747834
P19_NANOG_CNCC_D5_mem_q10_srt_rmdup_peaks_peak_11088
chr15	84340302	84340713
P19_NANOG_CNCC_D5_mem_q10_srt_rmdup_peaks_peak_11093
chr15	85260317	85260603
P19_NANOG_CNCC_D5_mem_q10_srt_rmdup_peaks_peak_11099
chr15	86232923	86233434
P19_NANOG_CNCC_D5_mem_q10_srt_rmdup_peaks_peak_11110
chr15	86259888	86260215
P19_NANOG_CNCC_D5_mem_q10_srt_rmdup_peaks_peak_11111
chr15	86294980	86295509
P19_NANOG_CNCC_D5_mem_q10_srt_rmdup_peaks_peak_11112
chr15	89152525	89152852
P19_NANOG_CNCC_D5_mem_q10_srt_rmdup_peaks_peak_11135
chr15	90548387	90548755
P19_NANOG_CNCC_D5_mem_q10_srt_rmdup_peaks_peak_11171
chr15	91576349	91576648
P19_NANOG_CNCC_D5_mem_q10_srt_rmdup_peaks_peak_11193
chr15	92060136	92060459
P19_NANOG_CNCC_D5_mem_q10_srt_rmdup_peaks_peak_11197
chr15	92419763	92420181
P19_NANOG_CNCC_D5_mem_q10_srt_rmdup_peaks_peak_11200
chr15	93187634	93188291
P19_NANOG_CNCC_D5_mem_q10_srt_rmdup_peaks_peak_11216
chr15	93213710	93214322
P19_NANOG_CNCC_D5_mem_q10_srt_rmdup_peaks_peak_11218
chr15	93257404	93257708
P19_NANOG_CNCC_D5_mem_q10_srt_rmdup_peaks_peak_11219
chr15	93286075	93286640
P19_NANOG_CNCC_D5_mem_q10_srt_rmdup_peaks_peak_11220
chr15	93364939	93365259
P19_NANOG_CNCC_D5_mem_q10_srt_rmdup_peaks_peak_11221
chr15	95387972	95388315
P19_NANOG_CNCC_D5_mem_q10_srt_rmdup_peaks_peak_11260
chr15	96838002	96838347
P19_NANOG_CNCC_D5_mem_q10_srt_rmdup_peaks_peak_11273
chr15	99297384	99297840
P19_NANOG_CNCC_D5_mem_q10_srt_rmdup_peaks_peak_11305
chr15	99329036	99329364
P19_NANOG_CNCC_D5_mem_q10_srt_rmdup_peaks_peak_11306
chr15	99395687	99396088
P19_NANOG_CNCC_D5_mem_q10_srt_rmdup_peaks_peak_11310
chr15	100107303	100107635
P19_NANOG_CNCC_D5_mem_q10_srt_rmdup_peaks_peak_11314
chr16	103501	103854
P19_NANOG_CNCC_D5_mem_q10_srt_rmdup_peaks_peak_11334
chr16	857721	858123
P19_NANOG_CNCC_D5_mem_q10_srt_rmdup_peaks_peak_11344
chr16	1696249	1696538

P19_NANOG_CNCC_D5_mem_q10_srt_rmdup_peaks_peak_11349
chr16 2273409 2273696
P19_NANOG_CNCC_D5_mem_q10_srt_rmdup_peaks_peak_11357
chr16 2977018 2977328
P19_NANOG_CNCC_D5_mem_q10_srt_rmdup_peaks_peak_11364
chr16 3070138 3070436
P19_NANOG_CNCC_D5_mem_q10_srt_rmdup_peaks_peak_11372
chr16 3128837 3129249
P19_NANOG_CNCC_D5_mem_q10_srt_rmdup_peaks_peak_11375
chr16 4079452 4080279
P19_NANOG_CNCC_D5_mem_q10_srt_rmdup_peaks_peak_11390
chr16 4321018 4321548
P19_NANOG_CNCC_D5_mem_q10_srt_rmdup_peaks_peak_11392
chr16 6395873 6396160
P19_NANOG_CNCC_D5_mem_q10_srt_rmdup_peaks_peak_11432
chr16 9060614 9060923
P19_NANOG_CNCC_D5_mem_q10_srt_rmdup_peaks_peak_11468
chr16 9242146 9242689
P19_NANOG_CNCC_D5_mem_q10_srt_rmdup_peaks_peak_11474
chr16 9254111 9254382
P19_NANOG_CNCC_D5_mem_q10_srt_rmdup_peaks_peak_11475
chr16 9260786 9261101
P19_NANOG_CNCC_D5_mem_q10_srt_rmdup_peaks_peak_11476
chr16 9828396 9828717
P19_NANOG_CNCC_D5_mem_q10_srt_rmdup_peaks_peak_11483
chr16 10065870 10066423
P19_NANOG_CNCC_D5_mem_q10_srt_rmdup_peaks_peak_11489
chr16 14396153 14396424
P19_NANOG_CNCC_D5_mem_q10_srt_rmdup_peaks_peak_11528
chr16 15052564 15053353
P19_NANOG_CNCC_D5_mem_q10_srt_rmdup_peaks_peak_11534
chr16 15068700 15068991
P19_NANOG_CNCC_D5_mem_q10_srt_rmdup_peaks_peak_11535
chr16 15993386 15993761
P19_NANOG_CNCC_D5_mem_q10_srt_rmdup_peaks_peak_11543
chr16 17510491 17510839
P19_NANOG_CNCC_D5_mem_q10_srt_rmdup_peaks_peak_11556
chr16 17722923 17723228
P19_NANOG_CNCC_D5_mem_q10_srt_rmdup_peaks_peak_11564
chr16 19485846 19486408
P19_NANOG_CNCC_D5_mem_q10_srt_rmdup_peaks_peak_11584
chr16 19799241 19799639
P19_NANOG_CNCC_D5_mem_q10_srt_rmdup_peaks_peak_11590
chr16 19828497 19828769
P19_NANOG_CNCC_D5_mem_q10_srt_rmdup_peaks_peak_11591
chr16 20127166 20127851
P19_NANOG_CNCC_D5_mem_q10_srt_rmdup_peaks_peak_11601
chr16 20214644 20215056
P19_NANOG_CNCC_D5_mem_q10_srt_rmdup_peaks_peak_11603
chr16 20752964 20753235

P19_NANOG_CNCC_D5_mem_q10_srt_rmdup_peaks_peak_11606
chr16 21964304 21964867
P19_NANOG_CNCC_D5_mem_q10_srt_rmdup_peaks_peak_11618
chr16 23105565 23105836
P19_NANOG_CNCC_D5_mem_q10_srt_rmdup_peaks_peak_11631
chr16 23611347 23611804
P19_NANOG_CNCC_D5_mem_q10_srt_rmdup_peaks_peak_11641
chr16 24162759 24163226
P19_NANOG_CNCC_D5_mem_q10_srt_rmdup_peaks_peak_11653
chr16 24175444 24175990
P19_NANOG_CNCC_D5_mem_q10_srt_rmdup_peaks_peak_11654
chr16 24368961 24369416
P19_NANOG_CNCC_D5_mem_q10_srt_rmdup_peaks_peak_11661
chr16 28104182 28104509
P19_NANOG_CNCC_D5_mem_q10_srt_rmdup_peaks_peak_11697
chr16 29973170 29973441
P19_NANOG_CNCC_D5_mem_q10_srt_rmdup_peaks_peak_11716
chr16 30032743 30033049
P19_NANOG_CNCC_D5_mem_q10_srt_rmdup_peaks_peak_11718
chr16 30043522 30043811
P19_NANOG_CNCC_D5_mem_q10_srt_rmdup_peaks_peak_11719
chr16 30389217 30389689
P19_NANOG_CNCC_D5_mem_q10_srt_rmdup_peaks_peak_11725
chr16 30418720 30418994
P19_NANOG_CNCC_D5_mem_q10_srt_rmdup_peaks_peak_11727
chr16 30808292 30808636
P19_NANOG_CNCC_D5_mem_q10_srt_rmdup_peaks_peak_11739
chr16 31053494 31053963
P19_NANOG_CNCC_D5_mem_q10_srt_rmdup_peaks_peak_11742
chr16 31181339 31181783
P19_NANOG_CNCC_D5_mem_q10_srt_rmdup_peaks_peak_11745
chr16 31519451 31519896
P19_NANOG_CNCC_D5_mem_q10_srt_rmdup_peaks_peak_11751
chr16 46966845 46967116
P19_NANOG_CNCC_D5_mem_q10_srt_rmdup_peaks_peak_11762
chr16 48387591 48387862
P19_NANOG_CNCC_D5_mem_q10_srt_rmdup_peaks_peak_11771
chr16 48645632 48646028
P19_NANOG_CNCC_D5_mem_q10_srt_rmdup_peaks_peak_11775
chr16 49732991 49733262
P19_NANOG_CNCC_D5_mem_q10_srt_rmdup_peaks_peak_11787
chr16 49888955 49889233
P19_NANOG_CNCC_D5_mem_q10_srt_rmdup_peaks_peak_11792
chr16 50514135 50514602
P19_NANOG_CNCC_D5_mem_q10_srt_rmdup_peaks_peak_11800
chr16 50539556 50539846
P19_NANOG_CNCC_D5_mem_q10_srt_rmdup_peaks_peak_11801
chr16 50882750 50883238
P19_NANOG_CNCC_D5_mem_q10_srt_rmdup_peaks_peak_11805
chr16 51572738 51573130

P19_NANOG_CNCC_D5_mem_q10_srt_rmdup_peaks_peak_11823
chr16 52298313 52298651
P19_NANOG_CNCC_D5_mem_q10_srt_rmdup_peaks_peak_11832
chr16 52496007 52496333
P19_NANOG_CNCC_D5_mem_q10_srt_rmdup_peaks_peak_11835
chr16 52503641 52503999
P19_NANOG_CNCC_D5_mem_q10_srt_rmdup_peaks_peak_11836
chr16 52567198 52567489
P19_NANOG_CNCC_D5_mem_q10_srt_rmdup_peaks_peak_11839
chr16 52579347 52579675
P19_NANOG_CNCC_D5_mem_q10_srt_rmdup_peaks_peak_11840
chr16 53420869 53421164
P19_NANOG_CNCC_D5_mem_q10_srt_rmdup_peaks_peak_11852
chr16 53748582 53748922
P19_NANOG_CNCC_D5_mem_q10_srt_rmdup_peaks_peak_11855
chr16 53790532 53790884
P19_NANOG_CNCC_D5_mem_q10_srt_rmdup_peaks_peak_11856
chr16 54088359 54088630
P19_NANOG_CNCC_D5_mem_q10_srt_rmdup_peaks_peak_11861
chr16 54210291 54210578
P19_NANOG_CNCC_D5_mem_q10_srt_rmdup_peaks_peak_11862
chr16 54964734 54965014
P19_NANOG_CNCC_D5_mem_q10_srt_rmdup_peaks_peak_11873
chr16 55866993 55867264
P19_NANOG_CNCC_D5_mem_q10_srt_rmdup_peaks_peak_11889
chr16 56279923 56280262
P19_NANOG_CNCC_D5_mem_q10_srt_rmdup_peaks_peak_11891
chr16 56659696 56660041
P19_NANOG_CNCC_D5_mem_q10_srt_rmdup_peaks_peak_11900
chr16 56826846 56827353
P19_NANOG_CNCC_D5_mem_q10_srt_rmdup_peaks_peak_11906
chr16 56835596 56836121
P19_NANOG_CNCC_D5_mem_q10_srt_rmdup_peaks_peak_11907
chr16 57046269 57046582
P19_NANOG_CNCC_D5_mem_q10_srt_rmdup_peaks_peak_11908
chr16 57221222 57221565
P19_NANOG_CNCC_D5_mem_q10_srt_rmdup_peaks_peak_11912
chr16 57294357 57294665
P19_NANOG_CNCC_D5_mem_q10_srt_rmdup_peaks_peak_11913
chr16 57570857 57571212
P19_NANOG_CNCC_D5_mem_q10_srt_rmdup_peaks_peak_11915
chr16 57897332 57897678
P19_NANOG_CNCC_D5_mem_q10_srt_rmdup_peaks_peak_11923
chr16 58956427 58956852
P19_NANOG_CNCC_D5_mem_q10_srt_rmdup_peaks_peak_11938
chr16 59258397 59258750
P19_NANOG_CNCC_D5_mem_q10_srt_rmdup_peaks_peak_11941
chr16 59950019 59950549
P19_NANOG_CNCC_D5_mem_q10_srt_rmdup_peaks_peak_11948
chr16 60447911 60448246

P19_NANOG_CNCC_D5_mem_q10_srt_rmdup_peaks_peak_11951
chr16 61453742 61454084
P19_NANOG_CNCC_D5_mem_q10_srt_rmdup_peaks_peak_11956
chr16 61539809 61540240
P19_NANOG_CNCC_D5_mem_q10_srt_rmdup_peaks_peak_11957
chr16 61960602 61960988
P19_NANOG_CNCC_D5_mem_q10_srt_rmdup_peaks_peak_11963
chr16 62984182 62984611
P19_NANOG_CNCC_D5_mem_q10_srt_rmdup_peaks_peak_11975
chr16 63116813 63117156
P19_NANOG_CNCC_D5_mem_q10_srt_rmdup_peaks_peak_11976
chr16 63219115 63219540
P19_NANOG_CNCC_D5_mem_q10_srt_rmdup_peaks_peak_11977
chr16 64578700 64579128
P19_NANOG_CNCC_D5_mem_q10_srt_rmdup_peaks_peak_11989
chr16 64952573 64953050
P19_NANOG_CNCC_D5_mem_q10_srt_rmdup_peaks_peak_11991
chr16 65188192 65188467
P19_NANOG_CNCC_D5_mem_q10_srt_rmdup_peaks_peak_11996
chr16 66684828 66685255
P19_NANOG_CNCC_D5_mem_q10_srt_rmdup_peaks_peak_12016
chr16 66728067 66728346
P19_NANOG_CNCC_D5_mem_q10_srt_rmdup_peaks_peak_12017
chr16 66773642 66774060
P19_NANOG_CNCC_D5_mem_q10_srt_rmdup_peaks_peak_12018
chr16 66907018 66907494
P19_NANOG_CNCC_D5_mem_q10_srt_rmdup_peaks_peak_12019
chr16 67240579 67240850
P19_NANOG_CNCC_D5_mem_q10_srt_rmdup_peaks_peak_12024
chr16 67448048 67448424
P19_NANOG_CNCC_D5_mem_q10_srt_rmdup_peaks_peak_12026
chr16 67627224 67627541
P19_NANOG_CNCC_D5_mem_q10_srt_rmdup_peaks_peak_12030
chr16 67628392 67628735
P19_NANOG_CNCC_D5_mem_q10_srt_rmdup_peaks_peak_12031
chr16 67976190 67976570
P19_NANOG_CNCC_D5_mem_q10_srt_rmdup_peaks_peak_12036
chr16 68827195 68827487
P19_NANOG_CNCC_D5_mem_q10_srt_rmdup_peaks_peak_12050
chr16 69853025 69853318
P19_NANOG_CNCC_D5_mem_q10_srt_rmdup_peaks_peak_12061
chr16 69867560 69867864
P19_NANOG_CNCC_D5_mem_q10_srt_rmdup_peaks_peak_12062
chr16 70835002 70835432
P19_NANOG_CNCC_D5_mem_q10_srt_rmdup_peaks_peak_12068
chr16 71598696 71599035
P19_NANOG_CNCC_D5_mem_q10_srt_rmdup_peaks_peak_12075
chr16 71756985 71757561
P19_NANOG_CNCC_D5_mem_q10_srt_rmdup_peaks_peak_12079
chr16 72975071 72975462

P19_NANOG_CNCC_D5_mem_q10_srt_rmdup_peaks_peak_12088
chr16 73368623 73368916
P19_NANOG_CNCC_D5_mem_q10_srt_rmdup_peaks_peak_12105
chr16 74464096 74464367
P19_NANOG_CNCC_D5_mem_q10_srt_rmdup_peaks_peak_12117
chr16 75126626 75126947
P19_NANOG_CNCC_D5_mem_q10_srt_rmdup_peaks_peak_12125
chr16 75550339 75550723
P19_NANOG_CNCC_D5_mem_q10_srt_rmdup_peaks_peak_12135
chr16 75623230 75623598
P19_NANOG_CNCC_D5_mem_q10_srt_rmdup_peaks_peak_12136
chr16 77408403 77408674
P19_NANOG_CNCC_D5_mem_q10_srt_rmdup_peaks_peak_12151
chr16 77823253 77823533
P19_NANOG_CNCC_D5_mem_q10_srt_rmdup_peaks_peak_12155
chr16 78616834 78617242
P19_NANOG_CNCC_D5_mem_q10_srt_rmdup_peaks_peak_12174
chr16 78801794 78802149
P19_NANOG_CNCC_D5_mem_q10_srt_rmdup_peaks_peak_12176
chr16 78807208 78807592
P19_NANOG_CNCC_D5_mem_q10_srt_rmdup_peaks_peak_12177
chr16 79421055 79421404
P19_NANOG_CNCC_D5_mem_q10_srt_rmdup_peaks_peak_12186
chr16 79517768 79518187
P19_NANOG_CNCC_D5_mem_q10_srt_rmdup_peaks_peak_12188
chr16 80796919 80797453
P19_NANOG_CNCC_D5_mem_q10_srt_rmdup_peaks_peak_12196
chr16 81012236 81012651
P19_NANOG_CNCC_D5_mem_q10_srt_rmdup_peaks_peak_12199
chr16 81973193 81973545
P19_NANOG_CNCC_D5_mem_q10_srt_rmdup_peaks_peak_12211
chr16 82007432 82007720
P19_NANOG_CNCC_D5_mem_q10_srt_rmdup_peaks_peak_12213
chr16 83580152 83580578
P19_NANOG_CNCC_D5_mem_q10_srt_rmdup_peaks_peak_12230
chr16 83942389 83942762
P19_NANOG_CNCC_D5_mem_q10_srt_rmdup_peaks_peak_12237
chr16 84172385 84172717
P19_NANOG_CNCC_D5_mem_q10_srt_rmdup_peaks_peak_12244
chr16 84589091 84589421
P19_NANOG_CNCC_D5_mem_q10_srt_rmdup_peaks_peak_12248
chr16 85558185 85558764
P19_NANOG_CNCC_D5_mem_q10_srt_rmdup_peaks_peak_12267
chr16 85722456 85722727
P19_NANOG_CNCC_D5_mem_q10_srt_rmdup_peaks_peak_12277
chr16 87122202 87122769
P19_NANOG_CNCC_D5_mem_q10_srt_rmdup_peaks_peak_12301
chr16 88022285 88022683
P19_NANOG_CNCC_D5_mem_q10_srt_rmdup_peaks_peak_12316
chr16 88616128 88616496

P19_NANOG_CNCC_D5_mem_q10_srt_rmdup_peaks_peak_12320
chr16 89496989 89497331
P19_NANOG_CNCC_D5_mem_q10_srt_rmdup_peaks_peak_12329
chr16 89989529 89989821
P19_NANOG_CNCC_D5_mem_q10_srt_rmdup_peaks_peak_12335
chr17 749749 750031
P19_NANOG_CNCC_D5_mem_q10_srt_rmdup_peaks_peak_12341
chr17 1462785 1463076
P19_NANOG_CNCC_D5_mem_q10_srt_rmdup_peaks_peak_12351
chr17 3416100 3416524
P19_NANOG_CNCC_D5_mem_q10_srt_rmdup_peaks_peak_12374
chr17 4375592 4376062
P19_NANOG_CNCC_D5_mem_q10_srt_rmdup_peaks_peak_12382
chr17 4622936 4623409
P19_NANOG_CNCC_D5_mem_q10_srt_rmdup_peaks_peak_12386
chr17 5606276 5606547
P19_NANOG_CNCC_D5_mem_q10_srt_rmdup_peaks_peak_12397
chr17 5816169 5816699
P19_NANOG_CNCC_D5_mem_q10_srt_rmdup_peaks_peak_12402
chr17 7358993 7359286
P19_NANOG_CNCC_D5_mem_q10_srt_rmdup_peaks_peak_12427
chr17 7493368 7493935
P19_NANOG_CNCC_D5_mem_q10_srt_rmdup_peaks_peak_12433
chr17 8029778 8030145
P19_NANOG_CNCC_D5_mem_q10_srt_rmdup_peaks_peak_12444
chr17 8152203 8152474
P19_NANOG_CNCC_D5_mem_q10_srt_rmdup_peaks_peak_12453
chr17 8270841 8271405
P19_NANOG_CNCC_D5_mem_q10_srt_rmdup_peaks_peak_12455
chr17 10651446 10651802
P19_NANOG_CNCC_D5_mem_q10_srt_rmdup_peaks_peak_12475
chr17 11924456 11924989
P19_NANOG_CNCC_D5_mem_q10_srt_rmdup_peaks_peak_12486
chr17 12125396 12125749
P19_NANOG_CNCC_D5_mem_q10_srt_rmdup_peaks_peak_12488
chr17 12645349 12645701
P19_NANOG_CNCC_D5_mem_q10_srt_rmdup_peaks_peak_12492
chr17 14234018 14234293
P19_NANOG_CNCC_D5_mem_q10_srt_rmdup_peaks_peak_12515
chr17 14935296 14935702
P19_NANOG_CNCC_D5_mem_q10_srt_rmdup_peaks_peak_12526
chr17 14980863 14981155
P19_NANOG_CNCC_D5_mem_q10_srt_rmdup_peaks_peak_12527
chr17 17627902 17628229
P19_NANOG_CNCC_D5_mem_q10_srt_rmdup_peaks_peak_12559
chr17 17653276 17653610
P19_NANOG_CNCC_D5_mem_q10_srt_rmdup_peaks_peak_12561
chr17 17845839 17846284
P19_NANOG_CNCC_D5_mem_q10_srt_rmdup_peaks_peak_12570
chr17 18056925 18057259

P19_NANOG_CNCC_D5_mem_q10_srt_rmdup_peaks_peak_12571
chr17 18163661 18164004
P19_NANOG_CNCC_D5_mem_q10_srt_rmdup_peaks_peak_12576
chr17 19483445 19483716
P19_NANOG_CNCC_D5_mem_q10_srt_rmdup_peaks_peak_12588
chr17 20580130 20580409
P19_NANOG_CNCC_D5_mem_q10_srt_rmdup_peaks_peak_12601
chr17 20755831 20756301
P19_NANOG_CNCC_D5_mem_q10_srt_rmdup_peaks_peak_12602
chr17 20990001 20990272
P19_NANOG_CNCC_D5_mem_q10_srt_rmdup_peaks_peak_12605
chr17 21104388 21104684
P19_NANOG_CNCC_D5_mem_q10_srt_rmdup_peaks_peak_12608
chr17 25783262 25783653
P19_NANOG_CNCC_D5_mem_q10_srt_rmdup_peaks_peak_12615
chr17 25856839 25857119
P19_NANOG_CNCC_D5_mem_q10_srt_rmdup_peaks_peak_12620
chr17 26183881 26184298
P19_NANOG_CNCC_D5_mem_q10_srt_rmdup_peaks_peak_12622
chr17 26663357 26663790
P19_NANOG_CNCC_D5_mem_q10_srt_rmdup_peaks_peak_12632
chr17 26733517 26733788
P19_NANOG_CNCC_D5_mem_q10_srt_rmdup_peaks_peak_12635
chr17 27169692 27170002
P19_NANOG_CNCC_D5_mem_q10_srt_rmdup_peaks_peak_12649
chr17 28098602 28098961
P19_NANOG_CNCC_D5_mem_q10_srt_rmdup_peaks_peak_12669
chr17 28705727 28706032
P19_NANOG_CNCC_D5_mem_q10_srt_rmdup_peaks_peak_12676
chr17 29233192 29233535
P19_NANOG_CNCC_D5_mem_q10_srt_rmdup_peaks_peak_12683
chr17 29794791 29795139
P19_NANOG_CNCC_D5_mem_q10_srt_rmdup_peaks_peak_12692
chr17 32688561 32688949
P19_NANOG_CNCC_D5_mem_q10_srt_rmdup_peaks_peak_12733
chr17 34068077 34068386
P19_NANOG_CNCC_D5_mem_q10_srt_rmdup_peaks_peak_12760
chr17 34135858 34136204
P19_NANOG_CNCC_D5_mem_q10_srt_rmdup_peaks_peak_12763
chr17 35295937 35296255
P19_NANOG_CNCC_D5_mem_q10_srt_rmdup_peaks_peak_12777
chr17 35466930 35467285
P19_NANOG_CNCC_D5_mem_q10_srt_rmdup_peaks_peak_12784
chr17 35476621 35476990
P19_NANOG_CNCC_D5_mem_q10_srt_rmdup_peaks_peak_12785
chr17 35492438 35492843
P19_NANOG_CNCC_D5_mem_q10_srt_rmdup_peaks_peak_12786
chr17 35855246 35855577
P19_NANOG_CNCC_D5_mem_q10_srt_rmdup_peaks_peak_12795
chr17 36095102 36095467

P19_NANOG_CNCC_D5_mem_q10_srt_rmdup_peaks_peak_12801
chr17 37039802 37040486
P19_NANOG_CNCC_D5_mem_q10_srt_rmdup_peaks_peak_12820
chr17 37886373 37886875
P19_NANOG_CNCC_D5_mem_q10_srt_rmdup_peaks_peak_12839
chr17 38267879 38268215
P19_NANOG_CNCC_D5_mem_q10_srt_rmdup_peaks_peak_12845
chr17 38270721 38271020
P19_NANOG_CNCC_D5_mem_q10_srt_rmdup_peaks_peak_12846
chr17 38374967 38375253
P19_NANOG_CNCC_D5_mem_q10_srt_rmdup_peaks_peak_12848
chr17 39093434 39093882
P19_NANOG_CNCC_D5_mem_q10_srt_rmdup_peaks_peak_12860
chr17 39569475 39569876
P19_NANOG_CNCC_D5_mem_q10_srt_rmdup_peaks_peak_12864
chr17 39684419 39684690
P19_NANOG_CNCC_D5_mem_q10_srt_rmdup_peaks_peak_12865
chr17 40394815 40395175
P19_NANOG_CNCC_D5_mem_q10_srt_rmdup_peaks_peak_12883
chr17 40831796 40832067
P19_NANOG_CNCC_D5_mem_q10_srt_rmdup_peaks_peak_12891
chr17 41069196 41069578
P19_NANOG_CNCC_D5_mem_q10_srt_rmdup_peaks_peak_12892
chr17 41174317 41174590
P19_NANOG_CNCC_D5_mem_q10_srt_rmdup_peaks_peak_12894
chr17 41363549 41363820
P19_NANOG_CNCC_D5_mem_q10_srt_rmdup_peaks_peak_12901
chr17 41515878 41516920
P19_NANOG_CNCC_D5_mem_q10_srt_rmdup_peaks_peak_12907
chr17 41795916 41796289
P19_NANOG_CNCC_D5_mem_q10_srt_rmdup_peaks_peak_12914
chr17 41969719 41970107
P19_NANOG_CNCC_D5_mem_q10_srt_rmdup_peaks_peak_12919
chr17 42263924 42264230
P19_NANOG_CNCC_D5_mem_q10_srt_rmdup_peaks_peak_12926
chr17 42298542 42298823
P19_NANOG_CNCC_D5_mem_q10_srt_rmdup_peaks_peak_12928
chr17 45058559 45058874
P19_NANOG_CNCC_D5_mem_q10_srt_rmdup_peaks_peak_12953
chr17 45765025 45765385
P19_NANOG_CNCC_D5_mem_q10_srt_rmdup_peaks_peak_12962
chr17 46048131 46048740
P19_NANOG_CNCC_D5_mem_q10_srt_rmdup_peaks_peak_12966
chr17 46269634 46270270
P19_NANOG_CNCC_D5_mem_q10_srt_rmdup_peaks_peak_12973
chr17 46627789 46628060
P19_NANOG_CNCC_D5_mem_q10_srt_rmdup_peaks_peak_12983
chr17 46670749 46671068
P19_NANOG_CNCC_D5_mem_q10_srt_rmdup_peaks_peak_12991
chr17 47132189 47132460

P19_NANOG_CNCC_D5_mem_q10_srt_rmdup_peaks_peak_13014
chr17 47269245 47269540
P19_NANOG_CNCC_D5_mem_q10_srt_rmdup_peaks_peak_13016
chr17 47269871 47270267
P19_NANOG_CNCC_D5_mem_q10_srt_rmdup_peaks_peak_13017
chr17 47354110 47354457
P19_NANOG_CNCC_D5_mem_q10_srt_rmdup_peaks_peak_13018
chr17 47479429 47479851
P19_NANOG_CNCC_D5_mem_q10_srt_rmdup_peaks_peak_13020
chr17 47589449 47589797
P19_NANOG_CNCC_D5_mem_q10_srt_rmdup_peaks_peak_13023
chr17 47679671 47680239
P19_NANOG_CNCC_D5_mem_q10_srt_rmdup_peaks_peak_13026
chr17 47695212 47695621
P19_NANOG_CNCC_D5_mem_q10_srt_rmdup_peaks_peak_13027
chr17 47902984 47903296
P19_NANOG_CNCC_D5_mem_q10_srt_rmdup_peaks_peak_13032
chr17 47953298 47953569
P19_NANOG_CNCC_D5_mem_q10_srt_rmdup_peaks_peak_13034
chr17 48053562 48054017
P19_NANOG_CNCC_D5_mem_q10_srt_rmdup_peaks_peak_13039
chr17 48217451 48217731
P19_NANOG_CNCC_D5_mem_q10_srt_rmdup_peaks_peak_13044
chr17 48450255 48450712
P19_NANOG_CNCC_D5_mem_q10_srt_rmdup_peaks_peak_13049
chr17 48658541 48658818
P19_NANOG_CNCC_D5_mem_q10_srt_rmdup_peaks_peak_13051
chr17 49337801 49338145
P19_NANOG_CNCC_D5_mem_q10_srt_rmdup_peaks_peak_13061
chr17 49379660 49379984
P19_NANOG_CNCC_D5_mem_q10_srt_rmdup_peaks_peak_13062
chr17 49495838 49496186
P19_NANOG_CNCC_D5_mem_q10_srt_rmdup_peaks_peak_13067
chr17 49717546 49717817
P19_NANOG_CNCC_D5_mem_q10_srt_rmdup_peaks_peak_13069
chr17 50514153 50514825
P19_NANOG_CNCC_D5_mem_q10_srt_rmdup_peaks_peak_13077
chr17 51220319 51220616
P19_NANOG_CNCC_D5_mem_q10_srt_rmdup_peaks_peak_13081
chr17 51822422 51822693
P19_NANOG_CNCC_D5_mem_q10_srt_rmdup_peaks_peak_13086
chr17 52607841 52608159
P19_NANOG_CNCC_D5_mem_q10_srt_rmdup_peaks_peak_13091
chr17 53150408 53150680
P19_NANOG_CNCC_D5_mem_q10_srt_rmdup_peaks_peak_13095
chr17 54307248 54307535
P19_NANOG_CNCC_D5_mem_q10_srt_rmdup_peaks_peak_13108
chr17 54340152 54340426
P19_NANOG_CNCC_D5_mem_q10_srt_rmdup_peaks_peak_13109
chr17 54776909 54777208

P19_NANOG_CNCC_D5_mem_q10_srt_rmdup_peaks_peak_13113
chr17 55655017 55655650
P19_NANOG_CNCC_D5_mem_q10_srt_rmdup_peaks_peak_13133
chr17 55775786 55776100
P19_NANOG_CNCC_D5_mem_q10_srt_rmdup_peaks_peak_13140
chr17 55910390 55910788
P19_NANOG_CNCC_D5_mem_q10_srt_rmdup_peaks_peak_13141
chr17 55982644 55983011
P19_NANOG_CNCC_D5_mem_q10_srt_rmdup_peaks_peak_13144
chr17 56070908 56071384
P19_NANOG_CNCC_D5_mem_q10_srt_rmdup_peaks_peak_13151
chr17 56401664 56402403
P19_NANOG_CNCC_D5_mem_q10_srt_rmdup_peaks_peak_13157
chr17 56499211 56499548
P19_NANOG_CNCC_D5_mem_q10_srt_rmdup_peaks_peak_13164
chr17 56679281 56679683
P19_NANOG_CNCC_D5_mem_q10_srt_rmdup_peaks_peak_13167
chr17 56943081 56943398
P19_NANOG_CNCC_D5_mem_q10_srt_rmdup_peaks_peak_13173
chr17 57374461 57374795
P19_NANOG_CNCC_D5_mem_q10_srt_rmdup_peaks_peak_13179
chr17 57930648 57930956
P19_NANOG_CNCC_D5_mem_q10_srt_rmdup_peaks_peak_13186
chr17 58985616 58986066
P19_NANOG_CNCC_D5_mem_q10_srt_rmdup_peaks_peak_13196
chr17 59436535 59437035
P19_NANOG_CNCC_D5_mem_q10_srt_rmdup_peaks_peak_13199
chr17 60501040 60501506
P19_NANOG_CNCC_D5_mem_q10_srt_rmdup_peaks_peak_13213
chr17 61904836 61905203
P19_NANOG_CNCC_D5_mem_q10_srt_rmdup_peaks_peak_13230
chr17 61926489 61926861
P19_NANOG_CNCC_D5_mem_q10_srt_rmdup_peaks_peak_13232
chr17 62235201 62235630
P19_NANOG_CNCC_D5_mem_q10_srt_rmdup_peaks_peak_13234
chr17 62334491 62334785
P19_NANOG_CNCC_D5_mem_q10_srt_rmdup_peaks_peak_13236
chr17 62503619 62503907
P19_NANOG_CNCC_D5_mem_q10_srt_rmdup_peaks_peak_13239
chr17 63204817 63205375
P19_NANOG_CNCC_D5_mem_q10_srt_rmdup_peaks_peak_13247
chr17 63786386 63786679
P19_NANOG_CNCC_D5_mem_q10_srt_rmdup_peaks_peak_13258
chr17 64489957 64490247
P19_NANOG_CNCC_D5_mem_q10_srt_rmdup_peaks_peak_13265
chr17 64760594 64760867
P19_NANOG_CNCC_D5_mem_q10_srt_rmdup_peaks_peak_13275
chr17 65471397 65471708
P19_NANOG_CNCC_D5_mem_q10_srt_rmdup_peaks_peak_13286
chr17 66201561 66201877

P19_NANOG_CNCC_D5_mem_q10_srt_rmdup_peaks_peak_13298
chr17 66290051 66290472
P19_NANOG_CNCC_D5_mem_q10_srt_rmdup_peaks_peak_13300
chr17 66530377 66530802
P19_NANOG_CNCC_D5_mem_q10_srt_rmdup_peaks_peak_13304
chr17 66599856 66600127
P19_NANOG_CNCC_D5_mem_q10_srt_rmdup_peaks_peak_13305
chr17 67366618 67366889
P19_NANOG_CNCC_D5_mem_q10_srt_rmdup_peaks_peak_13311
chr17 69457629 69457948
P19_NANOG_CNCC_D5_mem_q10_srt_rmdup_peaks_peak_13330
chr17 71227965 71228606
P19_NANOG_CNCC_D5_mem_q10_srt_rmdup_peaks_peak_13349
chr17 71649421 71649765
P19_NANOG_CNCC_D5_mem_q10_srt_rmdup_peaks_peak_13363
chr17 72232191 72232564
P19_NANOG_CNCC_D5_mem_q10_srt_rmdup_peaks_peak_13374
chr17 72667168 72667645
P19_NANOG_CNCC_D5_mem_q10_srt_rmdup_peaks_peak_13384
chr17 72978182 72978557
P19_NANOG_CNCC_D5_mem_q10_srt_rmdup_peaks_peak_13390
chr17 73826478 73826791
P19_NANOG_CNCC_D5_mem_q10_srt_rmdup_peaks_peak_13410
chr17 74255481 74255823
P19_NANOG_CNCC_D5_mem_q10_srt_rmdup_peaks_peak_13423
chr17 74580530 74580828
P19_NANOG_CNCC_D5_mem_q10_srt_rmdup_peaks_peak_13430
chr17 74609162 74609743
P19_NANOG_CNCC_D5_mem_q10_srt_rmdup_peaks_peak_13431
chr17 74679646 74679917
P19_NANOG_CNCC_D5_mem_q10_srt_rmdup_peaks_peak_13433
chr17 75063174 75063470
P19_NANOG_CNCC_D5_mem_q10_srt_rmdup_peaks_peak_13443
chr17 75277490 75277810
P19_NANOG_CNCC_D5_mem_q10_srt_rmdup_peaks_peak_13447
chr17 75467169 75467553
P19_NANOG_CNCC_D5_mem_q10_srt_rmdup_peaks_peak_13452
chr17 75542686 75543066
P19_NANOG_CNCC_D5_mem_q10_srt_rmdup_peaks_peak_13456
chr17 76347948 76348263
P19_NANOG_CNCC_D5_mem_q10_srt_rmdup_peaks_peak_13466
chr17 77110968 77111239
P19_NANOG_CNCC_D5_mem_q10_srt_rmdup_peaks_peak_13474
chr17 78620161 78620536
P19_NANOG_CNCC_D5_mem_q10_srt_rmdup_peaks_peak_13497
chr17 78684535 78685074
P19_NANOG_CNCC_D5_mem_q10_srt_rmdup_peaks_peak_13500
chr17 78757038 78757644
P19_NANOG_CNCC_D5_mem_q10_srt_rmdup_peaks_peak_13503
chr17 79026608 79027205

P19_NANOG_CNCC_D5_mem_q10_srt_rmdup_peaks_peak_13512
chr17 79313713 79313991
P19_NANOG_CNCC_D5_mem_q10_srt_rmdup_peaks_peak_13515
chr17 79339061 79339344
P19_NANOG_CNCC_D5_mem_q10_srt_rmdup_peaks_peak_13518
chr17 79456751 79457278
P19_NANOG_CNCC_D5_mem_q10_srt_rmdup_peaks_peak_13525
chr17 79935435 79935965
P19_NANOG_CNCC_D5_mem_q10_srt_rmdup_peaks_peak_13533
chr17 80055915 80056431
P19_NANOG_CNCC_D5_mem_q10_srt_rmdup_peaks_peak_13536
chr17 80778113 80779065
P19_NANOG_CNCC_D5_mem_q10_srt_rmdup_peaks_peak_13545
chr17 80779842 80780423
P19_NANOG_CNCC_D5_mem_q10_srt_rmdup_peaks_peak_13546
chr18 903627 904041
P19_NANOG_CNCC_D5_mem_q10_srt_rmdup_peaks_peak_13564
chr18 1045853 1046199
P19_NANOG_CNCC_D5_mem_q10_srt_rmdup_peaks_peak_13570
chr18 1208512 1208967
P19_NANOG_CNCC_D5_mem_q10_srt_rmdup_peaks_peak_13572
chr18 1297214 1297712
P19_NANOG_CNCC_D5_mem_q10_srt_rmdup_peaks_peak_13574
chr18 2173688 2174011
P19_NANOG_CNCC_D5_mem_q10_srt_rmdup_peaks_peak_13576
chr18 3605056 3605377
P19_NANOG_CNCC_D5_mem_q10_srt_rmdup_peaks_peak_13602
chr18 4917339 4917610
P19_NANOG_CNCC_D5_mem_q10_srt_rmdup_peaks_peak_13624
chr18 5196869 5197342
P19_NANOG_CNCC_D5_mem_q10_srt_rmdup_peaks_peak_13630
chr18 5276983 5277297
P19_NANOG_CNCC_D5_mem_q10_srt_rmdup_peaks_peak_13631
chr18 5477435 5477779
P19_NANOG_CNCC_D5_mem_q10_srt_rmdup_peaks_peak_13634
chr18 8367236 8367695
P19_NANOG_CNCC_D5_mem_q10_srt_rmdup_peaks_peak_13671
chr18 8794497 8794768
P19_NANOG_CNCC_D5_mem_q10_srt_rmdup_peaks_peak_13677
chr18 8980786 8981099
P19_NANOG_CNCC_D5_mem_q10_srt_rmdup_peaks_peak_13681
chr18 9417388 9417910
P19_NANOG_CNCC_D5_mem_q10_srt_rmdup_peaks_peak_13687
chr18 9429769 9430113
P19_NANOG_CNCC_D5_mem_q10_srt_rmdup_peaks_peak_13688
chr18 9839893 9840164
P19_NANOG_CNCC_D5_mem_q10_srt_rmdup_peaks_peak_13699
chr18 11255039 11255705
P19_NANOG_CNCC_D5_mem_q10_srt_rmdup_peaks_peak_13712
chr18 11689654 11689972

P19_NANOG_CNCC_D5_mem_q10_srt_rmdup_peaks_peak_13716
chr18 11905557 11905904
P19_NANOG_CNCC_D5_mem_q10_srt_rmdup_peaks_peak_13721
chr18 11947322 11947813
P19_NANOG_CNCC_D5_mem_q10_srt_rmdup_peaks_peak_13722
chr18 12741563 12741834
P19_NANOG_CNCC_D5_mem_q10_srt_rmdup_peaks_peak_13734
chr18 12993862 12994154
P19_NANOG_CNCC_D5_mem_q10_srt_rmdup_peaks_peak_13740
chr18 13712428 13712708
P19_NANOG_CNCC_D5_mem_q10_srt_rmdup_peaks_peak_13752
chr18 13954156 13954549
P19_NANOG_CNCC_D5_mem_q10_srt_rmdup_peaks_peak_13756
chr18 14105010 14105844
P19_NANOG_CNCC_D5_mem_q10_srt_rmdup_peaks_peak_13759
chr18 18883691 18883963
P19_NANOG_CNCC_D5_mem_q10_srt_rmdup_peaks_peak_13768
chr18 18971017 18971569
P19_NANOG_CNCC_D5_mem_q10_srt_rmdup_peaks_peak_13773
chr18 20019583 20020016
P19_NANOG_CNCC_D5_mem_q10_srt_rmdup_peaks_peak_13792
chr18 20218641 20218931
P19_NANOG_CNCC_D5_mem_q10_srt_rmdup_peaks_peak_13795
chr18 20852833 20853156
P19_NANOG_CNCC_D5_mem_q10_srt_rmdup_peaks_peak_13806
chr18 20949114 20949536
P19_NANOG_CNCC_D5_mem_q10_srt_rmdup_peaks_peak_13809
chr18 21198784 21199226
P19_NANOG_CNCC_D5_mem_q10_srt_rmdup_peaks_peak_13812
chr18 21441825 21442232
P19_NANOG_CNCC_D5_mem_q10_srt_rmdup_peaks_peak_13816
chr18 21530517 21530948
P19_NANOG_CNCC_D5_mem_q10_srt_rmdup_peaks_peak_13817
chr18 22006357 22006913
P19_NANOG_CNCC_D5_mem_q10_srt_rmdup_peaks_peak_13827
chr18 22409208 22409803
P19_NANOG_CNCC_D5_mem_q10_srt_rmdup_peaks_peak_13830
chr18 22555743 22556143
P19_NANOG_CNCC_D5_mem_q10_srt_rmdup_peaks_peak_13835
chr18 22567276 22567600
P19_NANOG_CNCC_D5_mem_q10_srt_rmdup_peaks_peak_13838
chr18 23259764 23260054
P19_NANOG_CNCC_D5_mem_q10_srt_rmdup_peaks_peak_13847
chr18 23841427 23841741
P19_NANOG_CNCC_D5_mem_q10_srt_rmdup_peaks_peak_13854
chr18 24265542 24266088
P19_NANOG_CNCC_D5_mem_q10_srt_rmdup_peaks_peak_13864
chr18 24289128 24289587
P19_NANOG_CNCC_D5_mem_q10_srt_rmdup_peaks_peak_13865
chr18 24812476 24812801

P19_NANOG_CNCC_D5_mem_q10_srt_rmdup_peaks_peak_13871
chr18 24878407 24878789
P19_NANOG_CNCC_D5_mem_q10_srt_rmdup_peaks_peak_13873
chr18 25063397 25063738
P19_NANOG_CNCC_D5_mem_q10_srt_rmdup_peaks_peak_13875
chr18 25625249 25625765
P19_NANOG_CNCC_D5_mem_q10_srt_rmdup_peaks_peak_13884
chr18 25645304 25645689
P19_NANOG_CNCC_D5_mem_q10_srt_rmdup_peaks_peak_13886
chr18 25820729 25821006
P19_NANOG_CNCC_D5_mem_q10_srt_rmdup_peaks_peak_13891
chr18 26021526 26021824
P19_NANOG_CNCC_D5_mem_q10_srt_rmdup_peaks_peak_13893
chr18 26649804 26650278
P19_NANOG_CNCC_D5_mem_q10_srt_rmdup_peaks_peak_13898
chr18 26828830 26829245
P19_NANOG_CNCC_D5_mem_q10_srt_rmdup_peaks_peak_13902
chr18 26938080 26938502
P19_NANOG_CNCC_D5_mem_q10_srt_rmdup_peaks_peak_13904
chr18 28102414 28103002
P19_NANOG_CNCC_D5_mem_q10_srt_rmdup_peaks_peak_13909
chr18 28820900 28821235
P19_NANOG_CNCC_D5_mem_q10_srt_rmdup_peaks_peak_13916
chr18 28900355 28900655
P19_NANOG_CNCC_D5_mem_q10_srt_rmdup_peaks_peak_13918
chr18 30058754 30059132
P19_NANOG_CNCC_D5_mem_q10_srt_rmdup_peaks_peak_13928
chr18 30465865 30466160
P19_NANOG_CNCC_D5_mem_q10_srt_rmdup_peaks_peak_13934
chr18 31103717 31104064
P19_NANOG_CNCC_D5_mem_q10_srt_rmdup_peaks_peak_13936
chr18 31186592 31186903
P19_NANOG_CNCC_D5_mem_q10_srt_rmdup_peaks_peak_13938
chr18 32389644 32390013
P19_NANOG_CNCC_D5_mem_q10_srt_rmdup_peaks_peak_13949
chr18 32424106 32424378
P19_NANOG_CNCC_D5_mem_q10_srt_rmdup_peaks_peak_13950
chr18 32502955 32503231
P19_NANOG_CNCC_D5_mem_q10_srt_rmdup_peaks_peak_13952
chr18 32558012 32558483
P19_NANOG_CNCC_D5_mem_q10_srt_rmdup_peaks_peak_13953
chr18 33629854 33630194
P19_NANOG_CNCC_D5_mem_q10_srt_rmdup_peaks_peak_13967
chr18 34143261 34143623
P19_NANOG_CNCC_D5_mem_q10_srt_rmdup_peaks_peak_13975
chr18 34179786 34180117
P19_NANOG_CNCC_D5_mem_q10_srt_rmdup_peaks_peak_13978
chr18 34367532 34367835
P19_NANOG_CNCC_D5_mem_q10_srt_rmdup_peaks_peak_13980
chr18 34921584 34921855

P19_NANOG_CNCC_D5_mem_q10_srt_rmdup_peaks_peak_13987
chr18	36183894	36184263
P19_NANOG_CNCC_D5_mem_q10_srt_rmdup_peaks_peak_14009
chr18	38095595	38095866
P19_NANOG_CNCC_D5_mem_q10_srt_rmdup_peaks_peak_14027
chr18	38726390	38726983
P19_NANOG_CNCC_D5_mem_q10_srt_rmdup_peaks_peak_14029
chr18	39143405	39143695
P19_NANOG_CNCC_D5_mem_q10_srt_rmdup_peaks_peak_14033
chr18	39211851	39212232
P19_NANOG_CNCC_D5_mem_q10_srt_rmdup_peaks_peak_14035
chr18	39598396	39598762
P19_NANOG_CNCC_D5_mem_q10_srt_rmdup_peaks_peak_14038
chr18	39919209	39919804
P19_NANOG_CNCC_D5_mem_q10_srt_rmdup_peaks_peak_14041
chr18	40321794	40322181
P19_NANOG_CNCC_D5_mem_q10_srt_rmdup_peaks_peak_14042
chr18	40401498	40401834
P19_NANOG_CNCC_D5_mem_q10_srt_rmdup_peaks_peak_14044
chr18	40968290	40968613
P19_NANOG_CNCC_D5_mem_q10_srt_rmdup_peaks_peak_14046
chr18	41427309	41427651
P19_NANOG_CNCC_D5_mem_q10_srt_rmdup_peaks_peak_14051
chr18	41621674	41622031
P19_NANOG_CNCC_D5_mem_q10_srt_rmdup_peaks_peak_14052
chr18	43622350	43622860
P19_NANOG_CNCC_D5_mem_q10_srt_rmdup_peaks_peak_14068
chr18	44374641	44374918
P19_NANOG_CNCC_D5_mem_q10_srt_rmdup_peaks_peak_14078
chr18	45844861	45845132
P19_NANOG_CNCC_D5_mem_q10_srt_rmdup_peaks_peak_14106
chr18	46049340	46049747
P19_NANOG_CNCC_D5_mem_q10_srt_rmdup_peaks_peak_14107
chr18	46381108	46381379
P19_NANOG_CNCC_D5_mem_q10_srt_rmdup_peaks_peak_14113
chr18	46479343	46479780
P19_NANOG_CNCC_D5_mem_q10_srt_rmdup_peaks_peak_14117
chr18	46500867	46501250
P19_NANOG_CNCC_D5_mem_q10_srt_rmdup_peaks_peak_14120
chr18	46986973	46987320
P19_NANOG_CNCC_D5_mem_q10_srt_rmdup_peaks_peak_14124
chr18	47088210	47088481
P19_NANOG_CNCC_D5_mem_q10_srt_rmdup_peaks_peak_14129
chr18	47934454	47934797
P19_NANOG_CNCC_D5_mem_q10_srt_rmdup_peaks_peak_14141
chr18	48122274	48122551
P19_NANOG_CNCC_D5_mem_q10_srt_rmdup_peaks_peak_14142
chr18	50208733	50209014
P19_NANOG_CNCC_D5_mem_q10_srt_rmdup_peaks_peak_14167
chr18	50353442	50353745

P19_NANOG_CNCC_D5_mem_q10_srt_rmdup_peaks_peak_14168
chr18 51211354 51211672
P19_NANOG_CNCC_D5_mem_q10_srt_rmdup_peaks_peak_14173
chr18 52325677 52325993
P19_NANOG_CNCC_D5_mem_q10_srt_rmdup_peaks_peak_14184
chr18 52895546 52895902
P19_NANOG_CNCC_D5_mem_q10_srt_rmdup_peaks_peak_14186
chr18 53523473 53523783
P19_NANOG_CNCC_D5_mem_q10_srt_rmdup_peaks_peak_14201
chr18 53665443 53665879
P19_NANOG_CNCC_D5_mem_q10_srt_rmdup_peaks_peak_14203
chr18 54394045 54394559
P19_NANOG_CNCC_D5_mem_q10_srt_rmdup_peaks_peak_14211
chr18 54503559 54503859
P19_NANOG_CNCC_D5_mem_q10_srt_rmdup_peaks_peak_14212
chr18 55747935 55748210
P19_NANOG_CNCC_D5_mem_q10_srt_rmdup_peaks_peak_14228
chr18 56588740 56589019
P19_NANOG_CNCC_D5_mem_q10_srt_rmdup_peaks_peak_14252
chr18 56716317 56716637
P19_NANOG_CNCC_D5_mem_q10_srt_rmdup_peaks_peak_14253
chr18 57733800 57734274
P19_NANOG_CNCC_D5_mem_q10_srt_rmdup_peaks_peak_14274
chr18 57734529 57735169
P19_NANOG_CNCC_D5_mem_q10_srt_rmdup_peaks_peak_14275
chr18 59320978 59321479
P19_NANOG_CNCC_D5_mem_q10_srt_rmdup_peaks_peak_14289
chr18 62965588 62965905
P19_NANOG_CNCC_D5_mem_q10_srt_rmdup_peaks_peak_14326
chr18 64512794 64513215
P19_NANOG_CNCC_D5_mem_q10_srt_rmdup_peaks_peak_14335
chr18 66106200 66106597
P19_NANOG_CNCC_D5_mem_q10_srt_rmdup_peaks_peak_14350
chr18 66231586 66231990
P19_NANOG_CNCC_D5_mem_q10_srt_rmdup_peaks_peak_14351
chr18 71250717 71251093
P19_NANOG_CNCC_D5_mem_q10_srt_rmdup_peaks_peak_14377
chr18 71781369 71781827
P19_NANOG_CNCC_D5_mem_q10_srt_rmdup_peaks_peak_14381
chr18 71851549 71852386
P19_NANOG_CNCC_D5_mem_q10_srt_rmdup_peaks_peak_14383
chr18 72634651 72634934
P19_NANOG_CNCC_D5_mem_q10_srt_rmdup_peaks_peak_14392
chr18 72674692 72675091
P19_NANOG_CNCC_D5_mem_q10_srt_rmdup_peaks_peak_14393
chr18 73222254 73222555
P19_NANOG_CNCC_D5_mem_q10_srt_rmdup_peaks_peak_14401
chr18 74391949 74392361
P19_NANOG_CNCC_D5_mem_q10_srt_rmdup_peaks_peak_14412
chr18 74608741 74609152

P19_NANOG_CNCC_D5_mem_q10_srt_rmdup_peaks_peak_14413
chr18 76062072 76062420
P19_NANOG_CNCC_D5_mem_q10_srt_rmdup_peaks_peak_14428
chr18 76114157 76114428
P19_NANOG_CNCC_D5_mem_q10_srt_rmdup_peaks_peak_14429
chr18 77417339 77417816
P19_NANOG_CNCC_D5_mem_q10_srt_rmdup_peaks_peak_14447
chr19 1248913 1249185
P19_NANOG_CNCC_D5_mem_q10_srt_rmdup_peaks_peak_14469
chr19 1750637 1750929
P19_NANOG_CNCC_D5_mem_q10_srt_rmdup_peaks_peak_14480
chr19 1795853 1796127
P19_NANOG_CNCC_D5_mem_q10_srt_rmdup_peaks_peak_14481
chr19 1904940 1905211
P19_NANOG_CNCC_D5_mem_q10_srt_rmdup_peaks_peak_14483
chr19 2041923 2042373
P19_NANOG_CNCC_D5_mem_q10_srt_rmdup_peaks_peak_14485
chr19 2425109 2425504
P19_NANOG_CNCC_D5_mem_q10_srt_rmdup_peaks_peak_14491
chr19 4204665 4205004
P19_NANOG_CNCC_D5_mem_q10_srt_rmdup_peaks_peak_14517
chr19 4994871 4995158
P19_NANOG_CNCC_D5_mem_q10_srt_rmdup_peaks_peak_14529
chr19 5667919 5668203
P19_NANOG_CNCC_D5_mem_q10_srt_rmdup_peaks_peak_14541
chr19 7587272 7587595
P19_NANOG_CNCC_D5_mem_q10_srt_rmdup_peaks_peak_14556
chr19 7598846 7599167
P19_NANOG_CNCC_D5_mem_q10_srt_rmdup_peaks_peak_14557
chr19 7985110 7985493
P19_NANOG_CNCC_D5_mem_q10_srt_rmdup_peaks_peak_14563
chr19 8067356 8067698
P19_NANOG_CNCC_D5_mem_q10_srt_rmdup_peaks_peak_14565
chr19 8590482 8590772
P19_NANOG_CNCC_D5_mem_q10_srt_rmdup_peaks_peak_14571
chr19 9434328 9434833
P19_NANOG_CNCC_D5_mem_q10_srt_rmdup_peaks_peak_14579
chr19 9692648 9693316
P19_NANOG_CNCC_D5_mem_q10_srt_rmdup_peaks_peak_14580
chr19 10025381 10025708
P19_NANOG_CNCC_D5_mem_q10_srt_rmdup_peaks_peak_14586
chr19 10341959 10342289
P19_NANOG_CNCC_D5_mem_q10_srt_rmdup_peaks_peak_14591
chr19 10514356 10514701
P19_NANOG_CNCC_D5_mem_q10_srt_rmdup_peaks_peak_14594
chr19 10527222 10527551
P19_NANOG_CNCC_D5_mem_q10_srt_rmdup_peaks_peak_14595
chr19 11076106 11076388
P19_NANOG_CNCC_D5_mem_q10_srt_rmdup_peaks_peak_14607
chr19 11248445 11248749

P19_NANOG_CNCC_D5_mem_q10_srt_rmdup_peaks_peak_14608
chr19 11308287 11308657
P19_NANOG_CNCC_D5_mem_q10_srt_rmdup_peaks_peak_14609
chr19 11766111 11766513
P19_NANOG_CNCC_D5_mem_q10_srt_rmdup_peaks_peak_14614
chr19 13206422 13206693
P19_NANOG_CNCC_D5_mem_q10_srt_rmdup_peaks_peak_14638
chr19 13266322 13267033
P19_NANOG_CNCC_D5_mem_q10_srt_rmdup_peaks_peak_14644
chr19 13571032 13571333
P19_NANOG_CNCC_D5_mem_q10_srt_rmdup_peaks_peak_14647
chr19 13641571 13641870
P19_NANOG_CNCC_D5_mem_q10_srt_rmdup_peaks_peak_14649
chr19 14630709 14631067
P19_NANOG_CNCC_D5_mem_q10_srt_rmdup_peaks_peak_14667
chr19 14684910 14685312
P19_NANOG_CNCC_D5_mem_q10_srt_rmdup_peaks_peak_14669
chr19 16187267 16187800
P19_NANOG_CNCC_D5_mem_q10_srt_rmdup_peaks_peak_14675
chr19 16308676 16308995
P19_NANOG_CNCC_D5_mem_q10_srt_rmdup_peaks_peak_14676
chr19 16396828 16397401
P19_NANOG_CNCC_D5_mem_q10_srt_rmdup_peaks_peak_14677
chr19 16940074 16940376
P19_NANOG_CNCC_D5_mem_q10_srt_rmdup_peaks_peak_14680
chr19 17715502 17715800
P19_NANOG_CNCC_D5_mem_q10_srt_rmdup_peaks_peak_14699
chr19 18029946 18030337
P19_NANOG_CNCC_D5_mem_q10_srt_rmdup_peaks_peak_14702
chr19 18343841 18344119
P19_NANOG_CNCC_D5_mem_q10_srt_rmdup_peaks_peak_14710
chr19 18528138 18528510
P19_NANOG_CNCC_D5_mem_q10_srt_rmdup_peaks_peak_14716
chr19 20213858 20214207
P19_NANOG_CNCC_D5_mem_q10_srt_rmdup_peaks_peak_14746
chr19 20262749 20263049
P19_NANOG_CNCC_D5_mem_q10_srt_rmdup_peaks_peak_14748
chr19 20396680 20396975
P19_NANOG_CNCC_D5_mem_q10_srt_rmdup_peaks_peak_14753
chr19 20649025 20649823
P19_NANOG_CNCC_D5_mem_q10_srt_rmdup_peaks_peak_14757
chr19 21646601 21646876
P19_NANOG_CNCC_D5_mem_q10_srt_rmdup_peaks_peak_14768
chr19 21807017 21807615
P19_NANOG_CNCC_D5_mem_q10_srt_rmdup_peaks_peak_14772
chr19 22039578 22039862
P19_NANOG_CNCC_D5_mem_q10_srt_rmdup_peaks_peak_14778
chr19 23260593 23260928
P19_NANOG_CNCC_D5_mem_q10_srt_rmdup_peaks_peak_14780
chr19 23301170 23301630

P19_NANOG_CNCC_D5_mem_q10_srt_rmdup_peaks_peak_14782
chr19 23732944 23733299
P19_NANOG_CNCC_D5_mem_q10_srt_rmdup_peaks_peak_14785
chr19 28883150 28883444
P19_NANOG_CNCC_D5_mem_q10_srt_rmdup_peaks_peak_14798
chr19 29322443 29322855
P19_NANOG_CNCC_D5_mem_q10_srt_rmdup_peaks_peak_14804
chr19 29858758 29859279
P19_NANOG_CNCC_D5_mem_q10_srt_rmdup_peaks_peak_14813
chr19 30015151 30015491
P19_NANOG_CNCC_D5_mem_q10_srt_rmdup_peaks_peak_14815
chr19 30419308 30419625
P19_NANOG_CNCC_D5_mem_q10_srt_rmdup_peaks_peak_14822
chr19 30623125 30623615
P19_NANOG_CNCC_D5_mem_q10_srt_rmdup_peaks_peak_14825
chr19 31915638 31916289
P19_NANOG_CNCC_D5_mem_q10_srt_rmdup_peaks_peak_14845
chr19 33028655 33029037
P19_NANOG_CNCC_D5_mem_q10_srt_rmdup_peaks_peak_14852
chr19 33546585 33547104
P19_NANOG_CNCC_D5_mem_q10_srt_rmdup_peaks_peak_14859
chr19 33667966 33668237
P19_NANOG_CNCC_D5_mem_q10_srt_rmdup_peaks_peak_14860
chr19 35168328 35168626
P19_NANOG_CNCC_D5_mem_q10_srt_rmdup_peaks_peak_14877
chr19 35581683 35582007
P19_NANOG_CNCC_D5_mem_q10_srt_rmdup_peaks_peak_14883
chr19 38146403 38146677
P19_NANOG_CNCC_D5_mem_q10_srt_rmdup_peaks_peak_14914
chr19 38885174 38885552
P19_NANOG_CNCC_D5_mem_q10_srt_rmdup_peaks_peak_14929
chr19 38893417 38893808
P19_NANOG_CNCC_D5_mem_q10_srt_rmdup_peaks_peak_14930
chr19 39086646 39087211
P19_NANOG_CNCC_D5_mem_q10_srt_rmdup_peaks_peak_14931
chr19 39156754 39157197
P19_NANOG_CNCC_D5_mem_q10_srt_rmdup_peaks_peak_14934
chr19 39322358 39322654
P19_NANOG_CNCC_D5_mem_q10_srt_rmdup_peaks_peak_14938
chr19 39794006 39794290
P19_NANOG_CNCC_D5_mem_q10_srt_rmdup_peaks_peak_14944
chr19 40029947 40030376
P19_NANOG_CNCC_D5_mem_q10_srt_rmdup_peaks_peak_14949
chr19 40098087 40098389
P19_NANOG_CNCC_D5_mem_q10_srt_rmdup_peaks_peak_14950
chr19 40161621 40161895
P19_NANOG_CNCC_D5_mem_q10_srt_rmdup_peaks_peak_14951
chr19 40723167 40723497
P19_NANOG_CNCC_D5_mem_q10_srt_rmdup_peaks_peak_14958
chr19 41431068 41431581

P19_NANOG_CNCC_D5_mem_q10_srt_rmdup_peaks_peak_14971
chr19 41724802 41725087
P19_NANOG_CNCC_D5_mem_q10_srt_rmdup_peaks_peak_14980
chr19 41913153 41913436
P19_NANOG_CNCC_D5_mem_q10_srt_rmdup_peaks_peak_14983
chr19 42426268 42426593
P19_NANOG_CNCC_D5_mem_q10_srt_rmdup_peaks_peak_14990
chr19 42463420 42463837
P19_NANOG_CNCC_D5_mem_q10_srt_rmdup_peaks_peak_14992
chr19 42963124 42963487
P19_NANOG_CNCC_D5_mem_q10_srt_rmdup_peaks_peak_15003
chr19 43966939 43967437
P19_NANOG_CNCC_D5_mem_q10_srt_rmdup_peaks_peak_15010
chr19 44576110 44576381
P19_NANOG_CNCC_D5_mem_q10_srt_rmdup_peaks_peak_15019
chr19 45250764 45251140
P19_NANOG_CNCC_D5_mem_q10_srt_rmdup_peaks_peak_15020
chr19 45280843 45281133
P19_NANOG_CNCC_D5_mem_q10_srt_rmdup_peaks_peak_15021
chr19 45737966 45738255
P19_NANOG_CNCC_D5_mem_q10_srt_rmdup_peaks_peak_15027
chr19 46218529 46218837
P19_NANOG_CNCC_D5_mem_q10_srt_rmdup_peaks_peak_15043
chr19 46456336 46456624
P19_NANOG_CNCC_D5_mem_q10_srt_rmdup_peaks_peak_15051
chr19 47364184 47364503
P19_NANOG_CNCC_D5_mem_q10_srt_rmdup_peaks_peak_15060
chr19 47633902 47634269
P19_NANOG_CNCC_D5_mem_q10_srt_rmdup_peaks_peak_15065
chr19 47951832 47952509
P19_NANOG_CNCC_D5_mem_q10_srt_rmdup_peaks_peak_15072
chr19 48610630 48611072
P19_NANOG_CNCC_D5_mem_q10_srt_rmdup_peaks_peak_15083
chr19 48983368 48983882
P19_NANOG_CNCC_D5_mem_q10_srt_rmdup_peaks_peak_15092
chr19 49240676 49240947
P19_NANOG_CNCC_D5_mem_q10_srt_rmdup_peaks_peak_15100
chr19 49653733 49654034
P19_NANOG_CNCC_D5_mem_q10_srt_rmdup_peaks_peak_15108
chr19 49728125 49728396
P19_NANOG_CNCC_D5_mem_q10_srt_rmdup_peaks_peak_15110
chr19 50016424 50016830
P19_NANOG_CNCC_D5_mem_q10_srt_rmdup_peaks_peak_15117
chr19 50370796 50371171
P19_NANOG_CNCC_D5_mem_q10_srt_rmdup_peaks_peak_15125
chr19 50379863 50380238
P19_NANOG_CNCC_D5_mem_q10_srt_rmdup_peaks_peak_15127
chr19 50964488 50964824
P19_NANOG_CNCC_D5_mem_q10_srt_rmdup_peaks_peak_15136
chr19 51162125 51162544

P19_NANOG_CNCC_D5_mem_q10_srt_rmdup_peaks_peak_15139
chr19 51263271 51263560
P19_NANOG_CNCC_D5_mem_q10_srt_rmdup_peaks_peak_15141
chr19 52383025 52383544
P19_NANOG_CNCC_D5_mem_q10_srt_rmdup_peaks_peak_15150
chr19 52412173 52412497
P19_NANOG_CNCC_D5_mem_q10_srt_rmdup_peaks_peak_15154
chr19 53161855 53162168
P19_NANOG_CNCC_D5_mem_q10_srt_rmdup_peaks_peak_15163
chr19 53193999 53194271
P19_NANOG_CNCC_D5_mem_q10_srt_rmdup_peaks_peak_15164
chr19 53446061 53446409
P19_NANOG_CNCC_D5_mem_q10_srt_rmdup_peaks_peak_15167
chr19 54115905 54116275
P19_NANOG_CNCC_D5_mem_q10_srt_rmdup_peaks_peak_15174
chr19 54145742 54146065
P19_NANOG_CNCC_D5_mem_q10_srt_rmdup_peaks_peak_15175
chr19 54342355 54342743
P19_NANOG_CNCC_D5_mem_q10_srt_rmdup_peaks_peak_15186
chr19 54372451 54372996
P19_NANOG_CNCC_D5_mem_q10_srt_rmdup_peaks_peak_15187
chr19 54463528 54463812
P19_NANOG_CNCC_D5_mem_q10_srt_rmdup_peaks_peak_15190
chr19 54640867 54641401
P19_NANOG_CNCC_D5_mem_q10_srt_rmdup_peaks_peak_15194
chr19 55690085 55690433
P19_NANOG_CNCC_D5_mem_q10_srt_rmdup_peaks_peak_15208
chr19 57874545 57874845
P19_NANOG_CNCC_D5_mem_q10_srt_rmdup_peaks_peak_15231
chr19 58280805 58281111
P19_NANOG_CNCC_D5_mem_q10_srt_rmdup_peaks_peak_15235
chr19 58427925 58428197
P19_NANOG_CNCC_D5_mem_q10_srt_rmdup_peaks_peak_15239
chr19 58554434 58554767
P19_NANOG_CNCC_D5_mem_q10_srt_rmdup_peaks_peak_15242
chr19 58666377 58666670
P19_NANOG_CNCC_D5_mem_q10_srt_rmdup_peaks_peak_15244
chr19 58673999 58674394
P19_NANOG_CNCC_D5_mem_q10_srt_rmdup_peaks_peak_15245
chr2 240467 240887
P19_NANOG_CNCC_D5_mem_q10_srt_rmdup_peaks_peak_15263
chr2 289632 289936
P19_NANOG_CNCC_D5_mem_q10_srt_rmdup_peaks_peak_15266
chr2 6763395 6763794
P19_NANOG_CNCC_D5_mem_q10_srt_rmdup_peaks_peak_15326
chr2 8185671 8186064
P19_NANOG_CNCC_D5_mem_q10_srt_rmdup_peaks_peak_15348
chr2 10908080 10908351
P19_NANOG_CNCC_D5_mem_q10_srt_rmdup_peaks_peak_15384
chr2 11148241 11148512

P19_NANOG_CNCC_D5_mem_q10_srt_rmdup_peaks_peak_15389
chr2 11682837 11683113
P19_NANOG_CNCC_D5_mem_q10_srt_rmdup_peaks_peak_15394
chr2 11828668 11829044
P19_NANOG_CNCC_D5_mem_q10_srt_rmdup_peaks_peak_15396
chr2 11970222 11970527
P19_NANOG_CNCC_D5_mem_q10_srt_rmdup_peaks_peak_15398
chr2 16105633 16106180
P19_NANOG_CNCC_D5_mem_q10_srt_rmdup_peaks_peak_15433
chr2 16349855 16350223
P19_NANOG_CNCC_D5_mem_q10_srt_rmdup_peaks_peak_15439
chr2 16404548 16404952
P19_NANOG_CNCC_D5_mem_q10_srt_rmdup_peaks_peak_15442
chr2 21270150 21270422
P19_NANOG_CNCC_D5_mem_q10_srt_rmdup_peaks_peak_15492
chr2 21881277 21881557
P19_NANOG_CNCC_D5_mem_q10_srt_rmdup_peaks_peak_15498
chr2 23935141 23935492
P19_NANOG_CNCC_D5_mem_q10_srt_rmdup_peaks_peak_15515
chr2 24086100 24086500
P19_NANOG_CNCC_D5_mem_q10_srt_rmdup_peaks_peak_15516
chr2 24634645 24635130
P19_NANOG_CNCC_D5_mem_q10_srt_rmdup_peaks_peak_15519
chr2 25264912 25265266
P19_NANOG_CNCC_D5_mem_q10_srt_rmdup_peaks_peak_15524
chr2 25781121 25781470
P19_NANOG_CNCC_D5_mem_q10_srt_rmdup_peaks_peak_15530
chr2 26407701 26408031
P19_NANOG_CNCC_D5_mem_q10_srt_rmdup_peaks_peak_15537
chr2 26915555 26915966
P19_NANOG_CNCC_D5_mem_q10_srt_rmdup_peaks_peak_15542
chr2 26979201 26979543
P19_NANOG_CNCC_D5_mem_q10_srt_rmdup_peaks_peak_15545
chr2 27255593 27255887
P19_NANOG_CNCC_D5_mem_q10_srt_rmdup_peaks_peak_15549
chr2 28903939 28904300
P19_NANOG_CNCC_D5_mem_q10_srt_rmdup_peaks_peak_15563
chr2 30175715 30176086
P19_NANOG_CNCC_D5_mem_q10_srt_rmdup_peaks_peak_15577
chr2 31625280 31625678
P19_NANOG_CNCC_D5_mem_q10_srt_rmdup_peaks_peak_15589
chr2 31900629 31901008
P19_NANOG_CNCC_D5_mem_q10_srt_rmdup_peaks_peak_15592
chr2 32502912 32503183
P19_NANOG_CNCC_D5_mem_q10_srt_rmdup_peaks_peak_15597
chr2 33533097 33533442
P19_NANOG_CNCC_D5_mem_q10_srt_rmdup_peaks_peak_15604
chr2 33843183 33843491
P19_NANOG_CNCC_D5_mem_q10_srt_rmdup_peaks_peak_15610
chr2 34230902 34231219

P19_NANOG_CNCC_D5_mem_q10_srt_rmdup_peaks_peak_15611
chr2 34943251 34943690
P19_NANOG_CNCC_D5_mem_q10_srt_rmdup_peaks_peak_15615
chr2 36049833 36050251
P19_NANOG_CNCC_D5_mem_q10_srt_rmdup_peaks_peak_15624
chr2 36096862 36097141
P19_NANOG_CNCC_D5_mem_q10_srt_rmdup_peaks_peak_15625
chr2 36688868 36689314
P19_NANOG_CNCC_D5_mem_q10_srt_rmdup_peaks_peak_15635
chr2 37192455 37192726
P19_NANOG_CNCC_D5_mem_q10_srt_rmdup_peaks_peak_15646
chr2 37416764 37417220
P19_NANOG_CNCC_D5_mem_q10_srt_rmdup_peaks_peak_15649
chr2 38334988 38335459
P19_NANOG_CNCC_D5_mem_q10_srt_rmdup_peaks_peak_15665
chr2 38659909 38660270
P19_NANOG_CNCC_D5_mem_q10_srt_rmdup_peaks_peak_15670
chr2 39219204 39219508
P19_NANOG_CNCC_D5_mem_q10_srt_rmdup_peaks_peak_15678
chr2 39584184 39584515
P19_NANOG_CNCC_D5_mem_q10_srt_rmdup_peaks_peak_15688
chr2 39711604 39712224
P19_NANOG_CNCC_D5_mem_q10_srt_rmdup_peaks_peak_15692
chr2 40330248 40330705
P19_NANOG_CNCC_D5_mem_q10_srt_rmdup_peaks_peak_15696
chr2 40469341 40469976
P19_NANOG_CNCC_D5_mem_q10_srt_rmdup_peaks_peak_15700
chr2 40561034 40561390
P19_NANOG_CNCC_D5_mem_q10_srt_rmdup_peaks_peak_15701
chr2 43133896 43134283
P19_NANOG_CNCC_D5_mem_q10_srt_rmdup_peaks_peak_15728
chr2 43406220 43406491
P19_NANOG_CNCC_D5_mem_q10_srt_rmdup_peaks_peak_15735
chr2 43413965 43414373
P19_NANOG_CNCC_D5_mem_q10_srt_rmdup_peaks_peak_15737
chr2 43442927 43443335
P19_NANOG_CNCC_D5_mem_q10_srt_rmdup_peaks_peak_15738
chr2 44298888 44299257
P19_NANOG_CNCC_D5_mem_q10_srt_rmdup_peaks_peak_15748
chr2 44358339 44358670
P19_NANOG_CNCC_D5_mem_q10_srt_rmdup_peaks_peak_15751
chr2 44406140 44406434
P19_NANOG_CNCC_D5_mem_q10_srt_rmdup_peaks_peak_15754
chr2 44625403 44625740
P19_NANOG_CNCC_D5_mem_q10_srt_rmdup_peaks_peak_15758
chr2 44704678 44704954
P19_NANOG_CNCC_D5_mem_q10_srt_rmdup_peaks_peak_15764
chr2 44901460 44902210
P19_NANOG_CNCC_D5_mem_q10_srt_rmdup_peaks_peak_15768
chr2 44932673 44932948

P19_NANOG_CNCC_D5_mem_q10_srt_rmdup_peaks_peak_15770
chr2 45002967 45003432
P19_NANOG_CNCC_D5_mem_q10_srt_rmdup_peaks_peak_15774
chr2 45020483 45020804
P19_NANOG_CNCC_D5_mem_q10_srt_rmdup_peaks_peak_15777
chr2 45048678 45049055
P19_NANOG_CNCC_D5_mem_q10_srt_rmdup_peaks_peak_15782
chr2 45446056 45446370
P19_NANOG_CNCC_D5_mem_q10_srt_rmdup_peaks_peak_15793
chr2 45617594 45617970
P19_NANOG_CNCC_D5_mem_q10_srt_rmdup_peaks_peak_15794
chr2 45876865 45877228
P19_NANOG_CNCC_D5_mem_q10_srt_rmdup_peaks_peak_15798
chr2 45877642 45877913
P19_NANOG_CNCC_D5_mem_q10_srt_rmdup_peaks_peak_15799
chr2 46186007 46186278
P19_NANOG_CNCC_D5_mem_q10_srt_rmdup_peaks_peak_15803
chr2 47007808 47008079
P19_NANOG_CNCC_D5_mem_q10_srt_rmdup_peaks_peak_15812
chr2 47494603 47494878
P19_NANOG_CNCC_D5_mem_q10_srt_rmdup_peaks_peak_15817
chr2 47562155 47562431
P19_NANOG_CNCC_D5_mem_q10_srt_rmdup_peaks_peak_15820
chr2 47584649 47585065
P19_NANOG_CNCC_D5_mem_q10_srt_rmdup_peaks_peak_15823
chr2 47820640 47820911
P19_NANOG_CNCC_D5_mem_q10_srt_rmdup_peaks_peak_15828
chr2 47968513 47968939
P19_NANOG_CNCC_D5_mem_q10_srt_rmdup_peaks_peak_15833
chr2 48097071 48097618
P19_NANOG_CNCC_D5_mem_q10_srt_rmdup_peaks_peak_15836
chr2 48573250 48573641
P19_NANOG_CNCC_D5_mem_q10_srt_rmdup_peaks_peak_15843
chr2 48946608 48946981
P19_NANOG_CNCC_D5_mem_q10_srt_rmdup_peaks_peak_15848
chr2 51302746 51303114
P19_NANOG_CNCC_D5_mem_q10_srt_rmdup_peaks_peak_15873
chr2 51337644 51337925
P19_NANOG_CNCC_D5_mem_q10_srt_rmdup_peaks_peak_15874
chr2 51875765 51876036
P19_NANOG_CNCC_D5_mem_q10_srt_rmdup_peaks_peak_15877
chr2 52480695 52481079
P19_NANOG_CNCC_D5_mem_q10_srt_rmdup_peaks_peak_15881
chr2 54134418 54134919
P19_NANOG_CNCC_D5_mem_q10_srt_rmdup_peaks_peak_15896
chr2 54861633 54862197
P19_NANOG_CNCC_D5_mem_q10_srt_rmdup_peaks_peak_15907
chr2 55400777 55401180
P19_NANOG_CNCC_D5_mem_q10_srt_rmdup_peaks_peak_15913
chr2 55514459 55514813

P19_NANOG_CNCC_D5_mem_q10_srt_rmdup_peaks_peak_15917
chr2 56460021 56460428
P19_NANOG_CNCC_D5_mem_q10_srt_rmdup_peaks_peak_15927
chr2 57412772 57413106
P19_NANOG_CNCC_D5_mem_q10_srt_rmdup_peaks_peak_15929
chr2 57635023 57635313
P19_NANOG_CNCC_D5_mem_q10_srt_rmdup_peaks_peak_15930
chr2 58464848 58465327
P19_NANOG_CNCC_D5_mem_q10_srt_rmdup_peaks_peak_15938
chr2 58546035 58546346
P19_NANOG_CNCC_D5_mem_q10_srt_rmdup_peaks_peak_15940
chr2 58749339 58749732
P19_NANOG_CNCC_D5_mem_q10_srt_rmdup_peaks_peak_15945
chr2 58947764 58948101
P19_NANOG_CNCC_D5_mem_q10_srt_rmdup_peaks_peak_15950
chr2 59058805 59059231
P19_NANOG_CNCC_D5_mem_q10_srt_rmdup_peaks_peak_15952
chr2 59690633 59690907
P19_NANOG_CNCC_D5_mem_q10_srt_rmdup_peaks_peak_15957
chr2 60442545 60442876
P19_NANOG_CNCC_D5_mem_q10_srt_rmdup_peaks_peak_15961
chr2 60524938 60525482
P19_NANOG_CNCC_D5_mem_q10_srt_rmdup_peaks_peak_15963
chr2 60724095 60724715
P19_NANOG_CNCC_D5_mem_q10_srt_rmdup_peaks_peak_15968
chr2 60741934 60742205
P19_NANOG_CNCC_D5_mem_q10_srt_rmdup_peaks_peak_15969
chr2 61153186 61153474
P19_NANOG_CNCC_D5_mem_q10_srt_rmdup_peaks_peak_15977
chr2 62964075 62964709
P19_NANOG_CNCC_D5_mem_q10_srt_rmdup_peaks_peak_15996
chr2 63561540 63561845
P19_NANOG_CNCC_D5_mem_q10_srt_rmdup_peaks_peak_16007
chr2 63732784 63733085
P19_NANOG_CNCC_D5_mem_q10_srt_rmdup_peaks_peak_16009
chr2 65215568 65216021
P19_NANOG_CNCC_D5_mem_q10_srt_rmdup_peaks_peak_16042
chr2 65388842 65389144
P19_NANOG_CNCC_D5_mem_q10_srt_rmdup_peaks_peak_16047
chr2 65861300 65861892
P19_NANOG_CNCC_D5_mem_q10_srt_rmdup_peaks_peak_16058
chr2 66418251 66418572
P19_NANOG_CNCC_D5_mem_q10_srt_rmdup_peaks_peak_16061
chr2 66764137 66764515
P19_NANOG_CNCC_D5_mem_q10_srt_rmdup_peaks_peak_16075
chr2 66770763 66771147
P19_NANOG_CNCC_D5_mem_q10_srt_rmdup_peaks_peak_16076
chr2 66945157 66945573
P19_NANOG_CNCC_D5_mem_q10_srt_rmdup_peaks_peak_16085
chr2 67564215 67564518

P19_NANOG_CNCC_D5_mem_q10_srt_rmdup_peaks_peak_16090
chr2 67888248 67888936
P19_NANOG_CNCC_D5_mem_q10_srt_rmdup_peaks_peak_16091
chr2 70314241 70314512
P19_NANOG_CNCC_D5_mem_q10_srt_rmdup_peaks_peak_16113
chr2 70474425 70474705
P19_NANOG_CNCC_D5_mem_q10_srt_rmdup_peaks_peak_16119
chr2 70568220 70568598
P19_NANOG_CNCC_D5_mem_q10_srt_rmdup_peaks_peak_16123
chr2 71132149 71132455
P19_NANOG_CNCC_D5_mem_q10_srt_rmdup_peaks_peak_16137
chr2 71383595 71384068
P19_NANOG_CNCC_D5_mem_q10_srt_rmdup_peaks_peak_16143
chr2 71754911 71755318
P19_NANOG_CNCC_D5_mem_q10_srt_rmdup_peaks_peak_16151
chr2 73402646 73403147
P19_NANOG_CNCC_D5_mem_q10_srt_rmdup_peaks_peak_16163
chr2 73496182 73496508
P19_NANOG_CNCC_D5_mem_q10_srt_rmdup_peaks_peak_16165
chr2 73612790 73613134
P19_NANOG_CNCC_D5_mem_q10_srt_rmdup_peaks_peak_16167
chr2 74419254 74419540
P19_NANOG_CNCC_D5_mem_q10_srt_rmdup_peaks_peak_16174
chr2 74584777 74585172
P19_NANOG_CNCC_D5_mem_q10_srt_rmdup_peaks_peak_16176
chr2 74743232 74743557
P19_NANOG_CNCC_D5_mem_q10_srt_rmdup_peaks_peak_16181
chr2 75503652 75503923
P19_NANOG_CNCC_D5_mem_q10_srt_rmdup_peaks_peak_16192
chr2 75937617 75937963
P19_NANOG_CNCC_D5_mem_q10_srt_rmdup_peaks_peak_16198
chr2 76148177 76148805
P19_NANOG_CNCC_D5_mem_q10_srt_rmdup_peaks_peak_16202
chr2 76772028 76772497
P19_NANOG_CNCC_D5_mem_q10_srt_rmdup_peaks_peak_16203
chr2 76960811 76961082
P19_NANOG_CNCC_D5_mem_q10_srt_rmdup_peaks_peak_16207
chr2 77212372 77212656
P19_NANOG_CNCC_D5_mem_q10_srt_rmdup_peaks_peak_16208
chr2 77263861 77264290
P19_NANOG_CNCC_D5_mem_q10_srt_rmdup_peaks_peak_16211
chr2 77793038 77793322
P19_NANOG_CNCC_D5_mem_q10_srt_rmdup_peaks_peak_16217
chr2 77856652 77857148
P19_NANOG_CNCC_D5_mem_q10_srt_rmdup_peaks_peak_16218
chr2 78024044 78024688
P19_NANOG_CNCC_D5_mem_q10_srt_rmdup_peaks_peak_16221
chr2 78984303 78984688
P19_NANOG_CNCC_D5_mem_q10_srt_rmdup_peaks_peak_16226
chr2 78995034 78995376

P19_NANOG_CNCC_D5_mem_q10_srt_rmdup_peaks_peak_16227
chr2 79620619 79621399
P19_NANOG_CNCC_D5_mem_q10_srt_rmdup_peaks_peak_16234
chr2 79664730 79665281
P19_NANOG_CNCC_D5_mem_q10_srt_rmdup_peaks_peak_16237
chr2 79816975 79817335
P19_NANOG_CNCC_D5_mem_q10_srt_rmdup_peaks_peak_16240
chr2 80145303 80145612
P19_NANOG_CNCC_D5_mem_q10_srt_rmdup_peaks_peak_16245
chr2 80281238 80281741
P19_NANOG_CNCC_D5_mem_q10_srt_rmdup_peaks_peak_16247
chr2 80497335 80497668
P19_NANOG_CNCC_D5_mem_q10_srt_rmdup_peaks_peak_16252
chr2 80551399 80551800
P19_NANOG_CNCC_D5_mem_q10_srt_rmdup_peaks_peak_16256
chr2 82232719 82233225
P19_NANOG_CNCC_D5_mem_q10_srt_rmdup_peaks_peak_16265
chr2 82426379 82426766
P19_NANOG_CNCC_D5_mem_q10_srt_rmdup_peaks_peak_16267
chr2 82508072 82508359
P19_NANOG_CNCC_D5_mem_q10_srt_rmdup_peaks_peak_16268
chr2 82509217 82509599
P19_NANOG_CNCC_D5_mem_q10_srt_rmdup_peaks_peak_16269
chr2 85493424 85493738
P19_NANOG_CNCC_D5_mem_q10_srt_rmdup_peaks_peak_16293
chr2 86333073 86333359
P19_NANOG_CNCC_D5_mem_q10_srt_rmdup_peaks_peak_16305
chr2 87510999 87511410
P19_NANOG_CNCC_D5_mem_q10_srt_rmdup_peaks_peak_16312
chr2 87961914 87962185
P19_NANOG_CNCC_D5_mem_q10_srt_rmdup_peaks_peak_16315
chr2 88571879 88572153
P19_NANOG_CNCC_D5_mem_q10_srt_rmdup_peaks_peak_16320
chr2 88998328 88998708
P19_NANOG_CNCC_D5_mem_q10_srt_rmdup_peaks_peak_16335
chr2 89014505 89014815
P19_NANOG_CNCC_D5_mem_q10_srt_rmdup_peaks_peak_16336
chr2 97153997 97154365
P19_NANOG_CNCC_D5_mem_q10_srt_rmdup_peaks_peak_16347
chr2 97213291 97213638
P19_NANOG_CNCC_D5_mem_q10_srt_rmdup_peaks_peak_16349
chr2 99111015 99111733
P19_NANOG_CNCC_D5_mem_q10_srt_rmdup_peaks_peak_16365
chr2 99698458 99698734
P19_NANOG_CNCC_D5_mem_q10_srt_rmdup_peaks_peak_16367
chr2 99862117 99862388
P19_NANOG_CNCC_D5_mem_q10_srt_rmdup_peaks_peak_16370
chr2 99979864 99980200
P19_NANOG_CNCC_D5_mem_q10_srt_rmdup_peaks_peak_16371
chr2 100188467 100189087

P19_NANOG_CNCC_D5_mem_q10_srt_rmdup_peaks_peak_16376
chr2	101029433	101029833
P19_NANOG_CNCC_D5_mem_q10_srt_rmdup_peaks_peak_16387
chr2	101487294	101487635
P19_NANOG_CNCC_D5_mem_q10_srt_rmdup_peaks_peak_16391
chr2	102590435	102590943
P19_NANOG_CNCC_D5_mem_q10_srt_rmdup_peaks_peak_16411
chr2	102918285	102918642
P19_NANOG_CNCC_D5_mem_q10_srt_rmdup_peaks_peak_16413
chr2	103130487	103130824
P19_NANOG_CNCC_D5_mem_q10_srt_rmdup_peaks_peak_16415
chr2	103202602	103202952
P19_NANOG_CNCC_D5_mem_q10_srt_rmdup_peaks_peak_16416
chr2	104623136	104623432
P19_NANOG_CNCC_D5_mem_q10_srt_rmdup_peaks_peak_16427
chr2	105031616	105032073
P19_NANOG_CNCC_D5_mem_q10_srt_rmdup_peaks_peak_16434
chr2	106773862	106774380
P19_NANOG_CNCC_D5_mem_q10_srt_rmdup_peaks_peak_16464
chr2	107192412	107192767
P19_NANOG_CNCC_D5_mem_q10_srt_rmdup_peaks_peak_16466
chr2	107350595	107350915
P19_NANOG_CNCC_D5_mem_q10_srt_rmdup_peaks_peak_16467
chr2	107506328	107506599
P19_NANOG_CNCC_D5_mem_q10_srt_rmdup_peaks_peak_16473
chr2	108174535	108175282
P19_NANOG_CNCC_D5_mem_q10_srt_rmdup_peaks_peak_16478
chr2	108691489	108691788
P19_NANOG_CNCC_D5_mem_q10_srt_rmdup_peaks_peak_16481
chr2	109683555	109684046
P19_NANOG_CNCC_D5_mem_q10_srt_rmdup_peaks_peak_16493
chr2	111442002	111442273
P19_NANOG_CNCC_D5_mem_q10_srt_rmdup_peaks_peak_16502
chr2	111486671	111486989
P19_NANOG_CNCC_D5_mem_q10_srt_rmdup_peaks_peak_16504
chr2	111591608	111592153
P19_NANOG_CNCC_D5_mem_q10_srt_rmdup_peaks_peak_16509
chr2	111879787	111880087
P19_NANOG_CNCC_D5_mem_q10_srt_rmdup_peaks_peak_16512
chr2	113609743	113610014
P19_NANOG_CNCC_D5_mem_q10_srt_rmdup_peaks_peak_16536
chr2	113956517	113956840
P19_NANOG_CNCC_D5_mem_q10_srt_rmdup_peaks_peak_16537
chr2	114032866	114033274
P19_NANOG_CNCC_D5_mem_q10_srt_rmdup_peaks_peak_16540
chr2	114584253	114584524
P19_NANOG_CNCC_D5_mem_q10_srt_rmdup_peaks_peak_16543
chr2	116358032	116358461
P19_NANOG_CNCC_D5_mem_q10_srt_rmdup_peaks_peak_16568
chr2	116917981	116918333

P19_NANOG_CNCC_D5_mem_q10_srt_rmdup_peaks_peak_16570
chr2 118036276 118036552
P19_NANOG_CNCC_D5_mem_q10_srt_rmdup_peaks_peak_16578
chr2 118570078 118570484
P19_NANOG_CNCC_D5_mem_q10_srt_rmdup_peaks_peak_16584
chr2 118607856 118608221
P19_NANOG_CNCC_D5_mem_q10_srt_rmdup_peaks_peak_16586
chr2 118857351 118857772
P19_NANOG_CNCC_D5_mem_q10_srt_rmdup_peaks_peak_16588
chr2 119582403 119582969
P19_NANOG_CNCC_D5_mem_q10_srt_rmdup_peaks_peak_16602
chr2 121332737 121333064
P19_NANOG_CNCC_D5_mem_q10_srt_rmdup_peaks_peak_16637
chr2 121411988 121412266
P19_NANOG_CNCC_D5_mem_q10_srt_rmdup_peaks_peak_16639
chr2 121540893 121541270
P19_NANOG_CNCC_D5_mem_q10_srt_rmdup_peaks_peak_16645
chr2 121645195 121645513
P19_NANOG_CNCC_D5_mem_q10_srt_rmdup_peaks_peak_16648
chr2 121776206 121776605
P19_NANOG_CNCC_D5_mem_q10_srt_rmdup_peaks_peak_16651
chr2 122548921 122549257
P19_NANOG_CNCC_D5_mem_q10_srt_rmdup_peaks_peak_16657
chr2 124416676 124417070
P19_NANOG_CNCC_D5_mem_q10_srt_rmdup_peaks_peak_16669
chr2 127589099 127589409
P19_NANOG_CNCC_D5_mem_q10_srt_rmdup_peaks_peak_16685
chr2 127592347 127592962
P19_NANOG_CNCC_D5_mem_q10_srt_rmdup_peaks_peak_16686
chr2 127782959 127783490
P19_NANOG_CNCC_D5_mem_q10_srt_rmdup_peaks_peak_16688
chr2 129136571 129136879
P19_NANOG_CNCC_D5_mem_q10_srt_rmdup_peaks_peak_16707
chr2 131484846 131485359
P19_NANOG_CNCC_D5_mem_q10_srt_rmdup_peaks_peak_16727
chr2 131601838 131602295
P19_NANOG_CNCC_D5_mem_q10_srt_rmdup_peaks_peak_16731
chr2 131887125 131887772
P19_NANOG_CNCC_D5_mem_q10_srt_rmdup_peaks_peak_16734
chr2 133177602 133177878
P19_NANOG_CNCC_D5_mem_q10_srt_rmdup_peaks_peak_16742
chr2 134904165 134904436
P19_NANOG_CNCC_D5_mem_q10_srt_rmdup_peaks_peak_16755
chr2 135530377 135530673
P19_NANOG_CNCC_D5_mem_q10_srt_rmdup_peaks_peak_16759
chr2 135532873 135533383
P19_NANOG_CNCC_D5_mem_q10_srt_rmdup_peaks_peak_16760
chr2 136023645 136023984
P19_NANOG_CNCC_D5_mem_q10_srt_rmdup_peaks_peak_16762
chr2 137086032 137086392

P19_NANOG_CNCC_D5_mem_q10_srt_rmdup_peaks_peak_16769
chr2 140029496 140029774
P19_NANOG_CNCC_D5_mem_q10_srt_rmdup_peaks_peak_16791
chr2 140482601 140482887
P19_NANOG_CNCC_D5_mem_q10_srt_rmdup_peaks_peak_16800
chr2 141867318 141867622
P19_NANOG_CNCC_D5_mem_q10_srt_rmdup_peaks_peak_16807
chr2 142582397 142583075
P19_NANOG_CNCC_D5_mem_q10_srt_rmdup_peaks_peak_16810
chr2 143732044 143732363
P19_NANOG_CNCC_D5_mem_q10_srt_rmdup_peaks_peak_16817
chr2 144425849 144426185
P19_NANOG_CNCC_D5_mem_q10_srt_rmdup_peaks_peak_16821
chr2 144610494 144610765
P19_NANOG_CNCC_D5_mem_q10_srt_rmdup_peaks_peak_16824
chr2 144650959 144651239
P19_NANOG_CNCC_D5_mem_q10_srt_rmdup_peaks_peak_16826
chr2 144988080 144988503
P19_NANOG_CNCC_D5_mem_q10_srt_rmdup_peaks_peak_16829
chr2 145107612 145107926
P19_NANOG_CNCC_D5_mem_q10_srt_rmdup_peaks_peak_16831
chr2 145470998 145471415
P19_NANOG_CNCC_D5_mem_q10_srt_rmdup_peaks_peak_16842
chr2 147840341 147840681
P19_NANOG_CNCC_D5_mem_q10_srt_rmdup_peaks_peak_16854
chr2 148601620 148602024
P19_NANOG_CNCC_D5_mem_q10_srt_rmdup_peaks_peak_16866
chr2 150977015 150977693
P19_NANOG_CNCC_D5_mem_q10_srt_rmdup_peaks_peak_16899
chr2 151839638 151839956
P19_NANOG_CNCC_D5_mem_q10_srt_rmdup_peaks_peak_16910
chr2 152031665 152032002
P19_NANOG_CNCC_D5_mem_q10_srt_rmdup_peaks_peak_16913
chr2 152301017 152301309
P19_NANOG_CNCC_D5_mem_q10_srt_rmdup_peaks_peak_16920
chr2 153916184 153916503
P19_NANOG_CNCC_D5_mem_q10_srt_rmdup_peaks_peak_16935
chr2 154941803 154942074
P19_NANOG_CNCC_D5_mem_q10_srt_rmdup_peaks_peak_16942
chr2 155826198 155826469
P19_NANOG_CNCC_D5_mem_q10_srt_rmdup_peaks_peak_16950
chr2 158618083 158618397
P19_NANOG_CNCC_D5_mem_q10_srt_rmdup_peaks_peak_16968
chr2 159722481 159722794
P19_NANOG_CNCC_D5_mem_q10_srt_rmdup_peaks_peak_16977
chr2 160893199 160893556
P19_NANOG_CNCC_D5_mem_q10_srt_rmdup_peaks_peak_16993
chr2 162833917 162834281
P19_NANOG_CNCC_D5_mem_q10_srt_rmdup_peaks_peak_17017
chr2 164064292 164064586

P19_NANOG_CNCC_D5_mem_q10_srt_rmdup_peaks_peak_17026
chr2 164415126 164415470
P19_NANOG_CNCC_D5_mem_q10_srt_rmdup_peaks_peak_17028
chr2 164497102 164497445
P19_NANOG_CNCC_D5_mem_q10_srt_rmdup_peaks_peak_17031
chr2 164870631 164871034
P19_NANOG_CNCC_D5_mem_q10_srt_rmdup_peaks_peak_17037
chr2 165735254 165735570
P19_NANOG_CNCC_D5_mem_q10_srt_rmdup_peaks_peak_17044
chr2 167429877 167430210
P19_NANOG_CNCC_D5_mem_q10_srt_rmdup_peaks_peak_17064
chr2 167471185 167471475
P19_NANOG_CNCC_D5_mem_q10_srt_rmdup_peaks_peak_17066
chr2 168352752 168353074
P19_NANOG_CNCC_D5_mem_q10_srt_rmdup_peaks_peak_17076
chr2 168398182 168398528
P19_NANOG_CNCC_D5_mem_q10_srt_rmdup_peaks_peak_17078
chr2 169042339 169042944
P19_NANOG_CNCC_D5_mem_q10_srt_rmdup_peaks_peak_17090
chr2 169375115 169375500
P19_NANOG_CNCC_D5_mem_q10_srt_rmdup_peaks_peak_17093
chr2 170333943 170334311
P19_NANOG_CNCC_D5_mem_q10_srt_rmdup_peaks_peak_17105
chr2 170711807 170712204
P19_NANOG_CNCC_D5_mem_q10_srt_rmdup_peaks_peak_17108
chr2 170929001 170929407
P19_NANOG_CNCC_D5_mem_q10_srt_rmdup_peaks_peak_17110
chr2 171146561 171147225
P19_NANOG_CNCC_D5_mem_q10_srt_rmdup_peaks_peak_17116
chr2 171634161 171634518
P19_NANOG_CNCC_D5_mem_q10_srt_rmdup_peaks_peak_17133
chr2 172378724 172379240
P19_NANOG_CNCC_D5_mem_q10_srt_rmdup_peaks_peak_17147
chr2 172649369 172649971
P19_NANOG_CNCC_D5_mem_q10_srt_rmdup_peaks_peak_17152
chr2 174455068 174455486
P19_NANOG_CNCC_D5_mem_q10_srt_rmdup_peaks_peak_17181
chr2 174897667 174898043
P19_NANOG_CNCC_D5_mem_q10_srt_rmdup_peaks_peak_17191
chr2 175306162 175306448
P19_NANOG_CNCC_D5_mem_q10_srt_rmdup_peaks_peak_17207
chr2 176174390 176174957
P19_NANOG_CNCC_D5_mem_q10_srt_rmdup_peaks_peak_17218
chr2 176319690 176319986
P19_NANOG_CNCC_D5_mem_q10_srt_rmdup_peaks_peak_17219
chr2 176334166 176334631
P19_NANOG_CNCC_D5_mem_q10_srt_rmdup_peaks_peak_17220
chr2 176465313 176465628
P19_NANOG_CNCC_D5_mem_q10_srt_rmdup_peaks_peak_17223
chr2 176699276 176699563

P19_NANOG_CNCC_D5_mem_q10_srt_rmdup_peaks_peak_17225
chr2 177386880 177387495
P19_NANOG_CNCC_D5_mem_q10_srt_rmdup_peaks_peak_17260
chr2 177388282 177388635
P19_NANOG_CNCC_D5_mem_q10_srt_rmdup_peaks_peak_17261
chr2 177543905 177544176
P19_NANOG_CNCC_D5_mem_q10_srt_rmdup_peaks_peak_17264
chr2 177737189 177737460
P19_NANOG_CNCC_D5_mem_q10_srt_rmdup_peaks_peak_17269
chr2 178516453 178516929
P19_NANOG_CNCC_D5_mem_q10_srt_rmdup_peaks_peak_17280
chr2 178524254 178524872
P19_NANOG_CNCC_D5_mem_q10_srt_rmdup_peaks_peak_17281
chr2 178556442 178556896
P19_NANOG_CNCC_D5_mem_q10_srt_rmdup_peaks_peak_17284
chr2 179587084 179587403
P19_NANOG_CNCC_D5_mem_q10_srt_rmdup_peaks_peak_17297
chr2 179701601 179701945
P19_NANOG_CNCC_D5_mem_q10_srt_rmdup_peaks_peak_17298
chr2 179916501 179916774
P19_NANOG_CNCC_D5_mem_q10_srt_rmdup_peaks_peak_17301
chr2 182068088 182068599
P19_NANOG_CNCC_D5_mem_q10_srt_rmdup_peaks_peak_17319
chr2 182291446 182291798
P19_NANOG_CNCC_D5_mem_q10_srt_rmdup_peaks_peak_17321
chr2 182523240 182523599
P19_NANOG_CNCC_D5_mem_q10_srt_rmdup_peaks_peak_17323
chr2 183456641 183457043
P19_NANOG_CNCC_D5_mem_q10_srt_rmdup_peaks_peak_17328
chr2 183792675 183792989
P19_NANOG_CNCC_D5_mem_q10_srt_rmdup_peaks_peak_17333
chr2 183903205 183903501
P19_NANOG_CNCC_D5_mem_q10_srt_rmdup_peaks_peak_17336
chr2 183915185 183915601
P19_NANOG_CNCC_D5_mem_q10_srt_rmdup_peaks_peak_17337
chr2 185389860 185390275
P19_NANOG_CNCC_D5_mem_q10_srt_rmdup_peaks_peak_17351
chr2 188854828 188855285
P19_NANOG_CNCC_D5_mem_q10_srt_rmdup_peaks_peak_17371
chr2 190342327 190342652
P19_NANOG_CNCC_D5_mem_q10_srt_rmdup_peaks_peak_17385
chr2 190953669 190953968
P19_NANOG_CNCC_D5_mem_q10_srt_rmdup_peaks_peak_17387
chr2 191080138 191080633
P19_NANOG_CNCC_D5_mem_q10_srt_rmdup_peaks_peak_17388
chr2 191081515 191081803
P19_NANOG_CNCC_D5_mem_q10_srt_rmdup_peaks_peak_17389
chr2 191371458 191371738
P19_NANOG_CNCC_D5_mem_q10_srt_rmdup_peaks_peak_17391
chr2 192333674 192334077

P19_NANOG_CNCC_D5_mem_q10_srt_rmdup_peaks_peak_17400
chr2 192477147 192477530
P19_NANOG_CNCC_D5_mem_q10_srt_rmdup_peaks_peak_17402
chr2 192554055 192554398
P19_NANOG_CNCC_D5_mem_q10_srt_rmdup_peaks_peak_17403
chr2 193406822 193407194
P19_NANOG_CNCC_D5_mem_q10_srt_rmdup_peaks_peak_17410
chr2 193781426 193781829
P19_NANOG_CNCC_D5_mem_q10_srt_rmdup_peaks_peak_17411
chr2 197415786 197416085
P19_NANOG_CNCC_D5_mem_q10_srt_rmdup_peaks_peak_17428
chr2 197822464 197822819
P19_NANOG_CNCC_D5_mem_q10_srt_rmdup_peaks_peak_17432
chr2 197832007 197832304
P19_NANOG_CNCC_D5_mem_q10_srt_rmdup_peaks_peak_17433
chr2 198002216 198002561
P19_NANOG_CNCC_D5_mem_q10_srt_rmdup_peaks_peak_17434
chr2 198522339 198522668
P19_NANOG_CNCC_D5_mem_q10_srt_rmdup_peaks_peak_17443
chr2 199362787 199363209
P19_NANOG_CNCC_D5_mem_q10_srt_rmdup_peaks_peak_17450
chr2 200945929 200946414
P19_NANOG_CNCC_D5_mem_q10_srt_rmdup_peaks_peak_17468
chr2 201245091 201245384
P19_NANOG_CNCC_D5_mem_q10_srt_rmdup_peaks_peak_17474
chr2 201390633 201391017
P19_NANOG_CNCC_D5_mem_q10_srt_rmdup_peaks_peak_17476
chr2 202507350 202507697
P19_NANOG_CNCC_D5_mem_q10_srt_rmdup_peaks_peak_17486
chr2 202737376 202737931
P19_NANOG_CNCC_D5_mem_q10_srt_rmdup_peaks_peak_17491
chr2 202841783 202842181
P19_NANOG_CNCC_D5_mem_q10_srt_rmdup_peaks_peak_17497
chr2 202889716 202889987
P19_NANOG_CNCC_D5_mem_q10_srt_rmdup_peaks_peak_17501
chr2 202978268 202978690
P19_NANOG_CNCC_D5_mem_q10_srt_rmdup_peaks_peak_17509
chr2 203776725 203776996
P19_NANOG_CNCC_D5_mem_q10_srt_rmdup_peaks_peak_17516
chr2 204398001 204398281
P19_NANOG_CNCC_D5_mem_q10_srt_rmdup_peaks_peak_17523
chr2 205438279 205438709
P19_NANOG_CNCC_D5_mem_q10_srt_rmdup_peaks_peak_17533
chr2 206078330 206078855
P19_NANOG_CNCC_D5_mem_q10_srt_rmdup_peaks_peak_17538
chr2 206336557 206336867
P19_NANOG_CNCC_D5_mem_q10_srt_rmdup_peaks_peak_17544
chr2 206395856 206396155
P19_NANOG_CNCC_D5_mem_q10_srt_rmdup_peaks_peak_17546
chr2 206421355 206421835

P19_NANOG_CNCC_D5_mem_q10_srt_rmdup_peaks_peak_17548
chr2 206530432 206530725
P19_NANOG_CNCC_D5_mem_q10_srt_rmdup_peaks_peak_17550
chr2 206551595 206551997
P19_NANOG_CNCC_D5_mem_q10_srt_rmdup_peaks_peak_17551
chr2 208614948 208615452
P19_NANOG_CNCC_D5_mem_q10_srt_rmdup_peaks_peak_17590
chr2 208661385 208661903
P19_NANOG_CNCC_D5_mem_q10_srt_rmdup_peaks_peak_17594
chr2 208713109 208713397
P19_NANOG_CNCC_D5_mem_q10_srt_rmdup_peaks_peak_17597
chr2 208738140 208738534
P19_NANOG_CNCC_D5_mem_q10_srt_rmdup_peaks_peak_17598
chr2 209240512 209241043
P19_NANOG_CNCC_D5_mem_q10_srt_rmdup_peaks_peak_17608
chr2 209241693 209241974
P19_NANOG_CNCC_D5_mem_q10_srt_rmdup_peaks_peak_17609
chr2 210306221 210306576
P19_NANOG_CNCC_D5_mem_q10_srt_rmdup_peaks_peak_17621
chr2 210337133 210337554
P19_NANOG_CNCC_D5_mem_q10_srt_rmdup_peaks_peak_17624
chr2 213291883 213292154
P19_NANOG_CNCC_D5_mem_q10_srt_rmdup_peaks_peak_17653
chr2 214097868 214098282
P19_NANOG_CNCC_D5_mem_q10_srt_rmdup_peaks_peak_17660
chr2 215109592 215109920
P19_NANOG_CNCC_D5_mem_q10_srt_rmdup_peaks_peak_17666
chr2 216176590 216176861
P19_NANOG_CNCC_D5_mem_q10_srt_rmdup_peaks_peak_17676
chr2 217150369 217150680
P19_NANOG_CNCC_D5_mem_q10_srt_rmdup_peaks_peak_17685
chr2 217483582 217483869
P19_NANOG_CNCC_D5_mem_q10_srt_rmdup_peaks_peak_17695
chr2 217514581 217514894
P19_NANOG_CNCC_D5_mem_q10_srt_rmdup_peaks_peak_17697
chr2 217642342 217642668
P19_NANOG_CNCC_D5_mem_q10_srt_rmdup_peaks_peak_17702
chr2 217789222 217789550
P19_NANOG_CNCC_D5_mem_q10_srt_rmdup_peaks_peak_17704
chr2 217929012 217929333
P19_NANOG_CNCC_D5_mem_q10_srt_rmdup_peaks_peak_17706
chr2 218826107 218826471
P19_NANOG_CNCC_D5_mem_q10_srt_rmdup_peaks_peak_17719
chr2 219271132 219271548
P19_NANOG_CNCC_D5_mem_q10_srt_rmdup_peaks_peak_17722
chr2 219366355 219366681
P19_NANOG_CNCC_D5_mem_q10_srt_rmdup_peaks_peak_17724
chr2 219585312 219585666
P19_NANOG_CNCC_D5_mem_q10_srt_rmdup_peaks_peak_17729
chr2 219670300 219670674

P19_NANOG_CNCC_D5_mem_q10_srt_rmdup_peaks_peak_17730
chr2 219844746 219845121
P19_NANOG_CNCC_D5_mem_q10_srt_rmdup_peaks_peak_17731
chr2 221535117 221535738
P19_NANOG_CNCC_D5_mem_q10_srt_rmdup_peaks_peak_17756
chr2 222439116 222439649
P19_NANOG_CNCC_D5_mem_q10_srt_rmdup_peaks_peak_17768
chr2 223041532 223041945
P19_NANOG_CNCC_D5_mem_q10_srt_rmdup_peaks_peak_17780
chr2 223162286 223162767
P19_NANOG_CNCC_D5_mem_q10_srt_rmdup_peaks_peak_17789
chr2 223400260 223400658
P19_NANOG_CNCC_D5_mem_q10_srt_rmdup_peaks_peak_17804
chr2 223662043 223662326
P19_NANOG_CNCC_D5_mem_q10_srt_rmdup_peaks_peak_17812
chr2 223770877 223771377
P19_NANOG_CNCC_D5_mem_q10_srt_rmdup_peaks_peak_17814
chr2 224953495 224953921
P19_NANOG_CNCC_D5_mem_q10_srt_rmdup_peaks_peak_17828
chr2 225330937 225331275
P19_NANOG_CNCC_D5_mem_q10_srt_rmdup_peaks_peak_17831
chr2 225349970 225350271
P19_NANOG_CNCC_D5_mem_q10_srt_rmdup_peaks_peak_17832
chr2 225510680 225511196
P19_NANOG_CNCC_D5_mem_q10_srt_rmdup_peaks_peak_17833
chr2 226317148 226317577
P19_NANOG_CNCC_D5_mem_q10_srt_rmdup_peaks_peak_17839
chr2 227656311 227656584
P19_NANOG_CNCC_D5_mem_q10_srt_rmdup_peaks_peak_17850
chr2 229714165 229714517
P19_NANOG_CNCC_D5_mem_q10_srt_rmdup_peaks_peak_17863
chr2 232027863 232028322
P19_NANOG_CNCC_D5_mem_q10_srt_rmdup_peaks_peak_17877
chr2 232290636 232290985
P19_NANOG_CNCC_D5_mem_q10_srt_rmdup_peaks_peak_17882
chr2 232340733 232341158
P19_NANOG_CNCC_D5_mem_q10_srt_rmdup_peaks_peak_17884
chr2 232436546 232436876
P19_NANOG_CNCC_D5_mem_q10_srt_rmdup_peaks_peak_17885
chr2 233771728 233772047
P19_NANOG_CNCC_D5_mem_q10_srt_rmdup_peaks_peak_17907
chr2 233993997 233994268
P19_NANOG_CNCC_D5_mem_q10_srt_rmdup_peaks_peak_17913
chr2 234063487 234063770
P19_NANOG_CNCC_D5_mem_q10_srt_rmdup_peaks_peak_17914
chr2 234077784 234078209
P19_NANOG_CNCC_D5_mem_q10_srt_rmdup_peaks_peak_17915
chr2 234197371 234197808
P19_NANOG_CNCC_D5_mem_q10_srt_rmdup_peaks_peak_17916
chr2 235272581 235272877

P19_NANOG_CNCC_D5_mem_q10_srt_rmdup_peaks_peak_17924
chr2 235457588 235457885
P19_NANOG_CNCC_D5_mem_q10_srt_rmdup_peaks_peak_17931
chr2 235473149 235473727
P19_NANOG_CNCC_D5_mem_q10_srt_rmdup_peaks_peak_17932
chr2 236135492 236135881
P19_NANOG_CNCC_D5_mem_q10_srt_rmdup_peaks_peak_17956
chr2 236812910 236813242
P19_NANOG_CNCC_D5_mem_q10_srt_rmdup_peaks_peak_17971
chr2 236904731 236905002
P19_NANOG_CNCC_D5_mem_q10_srt_rmdup_peaks_peak_17973
chr2 236963169 236963492
P19_NANOG_CNCC_D5_mem_q10_srt_rmdup_peaks_peak_17975
chr2 237297521 237297793
P19_NANOG_CNCC_D5_mem_q10_srt_rmdup_peaks_peak_17985
chr2 237393996 237394675
P19_NANOG_CNCC_D5_mem_q10_srt_rmdup_peaks_peak_17986
chr2 237719825 237720142
P19_NANOG_CNCC_D5_mem_q10_srt_rmdup_peaks_peak_17990
chr2 242556761 242557067
P19_NANOG_CNCC_D5_mem_q10_srt_rmdup_peaks_peak_18033
chr2 242641288 242641579
P19_NANOG_CNCC_D5_mem_q10_srt_rmdup_peaks_peak_18034
chr20 555265 555720
P19_NANOG_CNCC_D5_mem_q10_srt_rmdup_peaks_peak_18053
chr20 656031 656365
P19_NANOG_CNCC_D5_mem_q10_srt_rmdup_peaks_peak_18056
chr20 770740 771204
P19_NANOG_CNCC_D5_mem_q10_srt_rmdup_peaks_peak_18058
chr20 796548 796917
P19_NANOG_CNCC_D5_mem_q10_srt_rmdup_peaks_peak_18060
chr20 1499190 1499662
P19_NANOG_CNCC_D5_mem_q10_srt_rmdup_peaks_peak_18069
chr20 1820561 1820908
P19_NANOG_CNCC_D5_mem_q10_srt_rmdup_peaks_peak_18077
chr20 2781344 2781755
P19_NANOG_CNCC_D5_mem_q10_srt_rmdup_peaks_peak_18101
chr20 4031324 4031669
P19_NANOG_CNCC_D5_mem_q10_srt_rmdup_peaks_peak_18112
chr20 5533793 5534443
P19_NANOG_CNCC_D5_mem_q10_srt_rmdup_peaks_peak_18125
chr20 6850882 6851180
P19_NANOG_CNCC_D5_mem_q10_srt_rmdup_peaks_peak_18139
chr20 7930956 7931410
P19_NANOG_CNCC_D5_mem_q10_srt_rmdup_peaks_peak_18142
chr20 8189680 8190231
P19_NANOG_CNCC_D5_mem_q10_srt_rmdup_peaks_peak_18144
chr20 9159962 9160278
P19_NANOG_CNCC_D5_mem_q10_srt_rmdup_peaks_peak_18151
chr20 9370511 9370854

P19_NANOG_CNCC_D5_mem_q10_srt_rmdup_peaks_peak_18154
chr20 9893163 9893537
P19_NANOG_CNCC_D5_mem_q10_srt_rmdup_peaks_peak_18162
chr20 10547177 10547496
P19_NANOG_CNCC_D5_mem_q10_srt_rmdup_peaks_peak_18177
chr20 11758070 11758576
P19_NANOG_CNCC_D5_mem_q10_srt_rmdup_peaks_peak_18197
chr20 13040066 13040337
P19_NANOG_CNCC_D5_mem_q10_srt_rmdup_peaks_peak_18213
chr20 13288696 13288987
P19_NANOG_CNCC_D5_mem_q10_srt_rmdup_peaks_peak_18215
chr20 14548888 14549234
P19_NANOG_CNCC_D5_mem_q10_srt_rmdup_peaks_peak_18224
chr20 15049915 15050337
P19_NANOG_CNCC_D5_mem_q10_srt_rmdup_peaks_peak_18236
chr20 16402321 16402611
P19_NANOG_CNCC_D5_mem_q10_srt_rmdup_peaks_peak_18251
chr20 17209905 17210258
P19_NANOG_CNCC_D5_mem_q10_srt_rmdup_peaks_peak_18257
chr20 18144872 18145252
P19_NANOG_CNCC_D5_mem_q10_srt_rmdup_peaks_peak_18269
chr20 18384887 18385277
P19_NANOG_CNCC_D5_mem_q10_srt_rmdup_peaks_peak_18271
chr20 19756786 19757127
P19_NANOG_CNCC_D5_mem_q10_srt_rmdup_peaks_peak_18284
chr20 20198165 20198713
P19_NANOG_CNCC_D5_mem_q10_srt_rmdup_peaks_peak_18287
chr20 20205937 20206350
P19_NANOG_CNCC_D5_mem_q10_srt_rmdup_peaks_peak_18288
chr20 20211419 20211819
P19_NANOG_CNCC_D5_mem_q10_srt_rmdup_peaks_peak_18289
chr20 20458360 20458656
P19_NANOG_CNCC_D5_mem_q10_srt_rmdup_peaks_peak_18298
chr20 22202701 22203015
P19_NANOG_CNCC_D5_mem_q10_srt_rmdup_peaks_peak_18331
chr20 22352591 22352929
P19_NANOG_CNCC_D5_mem_q10_srt_rmdup_peaks_peak_18332
chr20 23745255 23745818
P19_NANOG_CNCC_D5_mem_q10_srt_rmdup_peaks_peak_18359
chr20 25221962 25222589
P19_NANOG_CNCC_D5_mem_q10_srt_rmdup_peaks_peak_18374
chr20 25228651 25228922
P19_NANOG_CNCC_D5_mem_q10_srt_rmdup_peaks_peak_18375
chr20 30048441 30048755
P19_NANOG_CNCC_D5_mem_q10_srt_rmdup_peaks_peak_18382
chr20 30105192 30105601
P19_NANOG_CNCC_D5_mem_q10_srt_rmdup_peaks_peak_18383
chr20 30160621 30161215
P19_NANOG_CNCC_D5_mem_q10_srt_rmdup_peaks_peak_18384
chr20 30704403 30704688

P19_NANOG_CNCC_D5_mem_q10_srt_rmdup_peaks_peak_18403
chr20 30865279 30865550
P19_NANOG_CNCC_D5_mem_q10_srt_rmdup_peaks_peak_18405
chr20 31359858 31360206
P19_NANOG_CNCC_D5_mem_q10_srt_rmdup_peaks_peak_18419
chr20 32617504 32617783
P19_NANOG_CNCC_D5_mem_q10_srt_rmdup_peaks_peak_18432
chr20 32640198 32640599
P19_NANOG_CNCC_D5_mem_q10_srt_rmdup_peaks_peak_18435
chr20 32717422 32717782
P19_NANOG_CNCC_D5_mem_q10_srt_rmdup_peaks_peak_18436
chr20 33678436 33678767
P19_NANOG_CNCC_D5_mem_q10_srt_rmdup_peaks_peak_18443
chr20 34522063 34522530
P19_NANOG_CNCC_D5_mem_q10_srt_rmdup_peaks_peak_18454
chr20 36921791 36922101
P19_NANOG_CNCC_D5_mem_q10_srt_rmdup_peaks_peak_18483
chr20 37357075 37357543
P19_NANOG_CNCC_D5_mem_q10_srt_rmdup_peaks_peak_18494
chr20 37554740 37555176
P19_NANOG_CNCC_D5_mem_q10_srt_rmdup_peaks_peak_18500
chr20 39085990 39086492
P19_NANOG_CNCC_D5_mem_q10_srt_rmdup_peaks_peak_18515
chr20 42142604 42142972
P19_NANOG_CNCC_D5_mem_q10_srt_rmdup_peaks_peak_18530
chr20 42876779 42877092
P19_NANOG_CNCC_D5_mem_q10_srt_rmdup_peaks_peak_18536
chr20 44483374 44483697
P19_NANOG_CNCC_D5_mem_q10_srt_rmdup_peaks_peak_18557
chr20 44660272 44660798
P19_NANOG_CNCC_D5_mem_q10_srt_rmdup_peaks_peak_18562
chr20 45191006 45191495
P19_NANOG_CNCC_D5_mem_q10_srt_rmdup_peaks_peak_18571
chr20 45527152 45527449
P19_NANOG_CNCC_D5_mem_q10_srt_rmdup_peaks_peak_18578
chr20 45552526 45552823
P19_NANOG_CNCC_D5_mem_q10_srt_rmdup_peaks_peak_18580
chr20 45574160 45574470
P19_NANOG_CNCC_D5_mem_q10_srt_rmdup_peaks_peak_18581
chr20 46001633 46002060
P19_NANOG_CNCC_D5_mem_q10_srt_rmdup_peaks_peak_18594
chr20 46051869 46052661
P19_NANOG_CNCC_D5_mem_q10_srt_rmdup_peaks_peak_18596
chr20 46394987 46395418
P19_NANOG_CNCC_D5_mem_q10_srt_rmdup_peaks_peak_18604
chr20 46624607 46624890
P19_NANOG_CNCC_D5_mem_q10_srt_rmdup_peaks_peak_18609
chr20 46815514 46815995
P19_NANOG_CNCC_D5_mem_q10_srt_rmdup_peaks_peak_18610
chr20 46945384 46945693

P19_NANOG_CNCC_D5_mem_q10_srt_rmdup_peaks_peak_18612
chr20 47214988 47215433
P19_NANOG_CNCC_D5_mem_q10_srt_rmdup_peaks_peak_18621
chr20 48066732 48067048
P19_NANOG_CNCC_D5_mem_q10_srt_rmdup_peaks_peak_18629
chr20 48158306 48158731
P19_NANOG_CNCC_D5_mem_q10_srt_rmdup_peaks_peak_18632
chr20 48505171 48505576
P19_NANOG_CNCC_D5_mem_q10_srt_rmdup_peaks_peak_18643
chr20 49252566 49252963
P19_NANOG_CNCC_D5_mem_q10_srt_rmdup_peaks_peak_18654
chr20 49307125 49307647
P19_NANOG_CNCC_D5_mem_q10_srt_rmdup_peaks_peak_18655
chr20 49573150 49573535
P19_NANOG_CNCC_D5_mem_q10_srt_rmdup_peaks_peak_18663
chr20 49708406 49708915
P19_NANOG_CNCC_D5_mem_q10_srt_rmdup_peaks_peak_18668
chr20 51168788 51169140
P19_NANOG_CNCC_D5_mem_q10_srt_rmdup_peaks_peak_18695
chr20 51464984 51465255
P19_NANOG_CNCC_D5_mem_q10_srt_rmdup_peaks_peak_18696
chr20 52212160 52212478
P19_NANOG_CNCC_D5_mem_q10_srt_rmdup_peaks_peak_18704
chr20 52259220 52259540
P19_NANOG_CNCC_D5_mem_q10_srt_rmdup_peaks_peak_18705
chr20 56975091 56975399
P19_NANOG_CNCC_D5_mem_q10_srt_rmdup_peaks_peak_18758
chr20 57194828 57195158
P19_NANOG_CNCC_D5_mem_q10_srt_rmdup_peaks_peak_18764
chr20 57782810 57783137
P19_NANOG_CNCC_D5_mem_q10_srt_rmdup_peaks_peak_18775
chr20 57950751 57951180
P19_NANOG_CNCC_D5_mem_q10_srt_rmdup_peaks_peak_18777
chr20 58013296 58013693
P19_NANOG_CNCC_D5_mem_q10_srt_rmdup_peaks_peak_18778
chr20 60943661 60943949
P19_NANOG_CNCC_D5_mem_q10_srt_rmdup_peaks_peak_18798
chr20 61372653 61373099
P19_NANOG_CNCC_D5_mem_q10_srt_rmdup_peaks_peak_18800
chr21 15467308 15467701
P19_NANOG_CNCC_D5_mem_q10_srt_rmdup_peaks_peak_18832
chr21 15477151 15477453
P19_NANOG_CNCC_D5_mem_q10_srt_rmdup_peaks_peak_18833
chr21 15681811 15682092
P19_NANOG_CNCC_D5_mem_q10_srt_rmdup_peaks_peak_18835
chr21 16095160 16095503
P19_NANOG_CNCC_D5_mem_q10_srt_rmdup_peaks_peak_18838
chr21 16933746 16934035
P19_NANOG_CNCC_D5_mem_q10_srt_rmdup_peaks_peak_18852
chr21 16976589 16976943

P19_NANOG_CNCC_D5_mem_q10_srt_rmdup_peaks_peak_18853
chr21 17111193 17111515
P19_NANOG_CNCC_D5_mem_q10_srt_rmdup_peaks_peak_18854
chr21 17959500 17959915
P19_NANOG_CNCC_D5_mem_q10_srt_rmdup_peaks_peak_18860
chr21 18058249 18058527
P19_NANOG_CNCC_D5_mem_q10_srt_rmdup_peaks_peak_18862
chr21 19333919 19334244
P19_NANOG_CNCC_D5_mem_q10_srt_rmdup_peaks_peak_18873
chr21 20600604 20600902
P19_NANOG_CNCC_D5_mem_q10_srt_rmdup_peaks_peak_18882
chr21 21722106 21722394
P19_NANOG_CNCC_D5_mem_q10_srt_rmdup_peaks_peak_18888
chr21 22013625 22013901
P19_NANOG_CNCC_D5_mem_q10_srt_rmdup_peaks_peak_18891
chr21 22052075 22052361
P19_NANOG_CNCC_D5_mem_q10_srt_rmdup_peaks_peak_18893
chr21 23461909 23462354
P19_NANOG_CNCC_D5_mem_q10_srt_rmdup_peaks_peak_18905
chr21 24406384 24406731
P19_NANOG_CNCC_D5_mem_q10_srt_rmdup_peaks_peak_18909
chr21 24942054 24942399
P19_NANOG_CNCC_D5_mem_q10_srt_rmdup_peaks_peak_18912
chr21 25794202 25794710
P19_NANOG_CNCC_D5_mem_q10_srt_rmdup_peaks_peak_18913
chr21 27521271 27521688
P19_NANOG_CNCC_D5_mem_q10_srt_rmdup_peaks_peak_18928
chr21 30487162 30487464
P19_NANOG_CNCC_D5_mem_q10_srt_rmdup_peaks_peak_18952
chr21 30802831 30803225
P19_NANOG_CNCC_D5_mem_q10_srt_rmdup_peaks_peak_18954
chr21 31159170 31159441
P19_NANOG_CNCC_D5_mem_q10_srt_rmdup_peaks_peak_18957
chr21 32529969 32530472
P19_NANOG_CNCC_D5_mem_q10_srt_rmdup_peaks_peak_18962
chr21 32746924 32747195
P19_NANOG_CNCC_D5_mem_q10_srt_rmdup_peaks_peak_18966
chr21 32955928 32956622
P19_NANOG_CNCC_D5_mem_q10_srt_rmdup_peaks_peak_18972
chr21 33266684 33267075
P19_NANOG_CNCC_D5_mem_q10_srt_rmdup_peaks_peak_18976
chr21 33942170 33942441
P19_NANOG_CNCC_D5_mem_q10_srt_rmdup_peaks_peak_18988
chr21 34107850 34108125
P19_NANOG_CNCC_D5_mem_q10_srt_rmdup_peaks_peak_18991
chr21 34112200 34112599
P19_NANOG_CNCC_D5_mem_q10_srt_rmdup_peaks_peak_18992
chr21 34220179 34220689
P19_NANOG_CNCC_D5_mem_q10_srt_rmdup_peaks_peak_18994
chr21 34569328 34569633

P19_NANOG_CNCC_D5_mem_q10_srt_rmdup_peaks_peak_19006
chr21 35023532 35023820
P19_NANOG_CNCC_D5_mem_q10_srt_rmdup_peaks_peak_19012
chr21 36776595 36777102
P19_NANOG_CNCC_D5_mem_q10_srt_rmdup_peaks_peak_19040
chr21 36829881 36830179
P19_NANOG_CNCC_D5_mem_q10_srt_rmdup_peaks_peak_19041
chr21 37582004 37582385
P19_NANOG_CNCC_D5_mem_q10_srt_rmdup_peaks_peak_19053
chr21 38073335 38073622
P19_NANOG_CNCC_D5_mem_q10_srt_rmdup_peaks_peak_19060
chr21 39288566 39288900
P19_NANOG_CNCC_D5_mem_q10_srt_rmdup_peaks_peak_19089
chr21 39599945 39600265
P19_NANOG_CNCC_D5_mem_q10_srt_rmdup_peaks_peak_19090
chr21 39844704 39845028
P19_NANOG_CNCC_D5_mem_q10_srt_rmdup_peaks_peak_19094
chr21 39859897 39860476
P19_NANOG_CNCC_D5_mem_q10_srt_rmdup_peaks_peak_19095
chr21 40185090 40185441
P19_NANOG_CNCC_D5_mem_q10_srt_rmdup_peaks_peak_19098
chr21 40984575 40984903
P19_NANOG_CNCC_D5_mem_q10_srt_rmdup_peaks_peak_19116
chr21 41285913 41286270
P19_NANOG_CNCC_D5_mem_q10_srt_rmdup_peaks_peak_19122
chr21 41537519 41538023
P19_NANOG_CNCC_D5_mem_q10_srt_rmdup_peaks_peak_19124
chr21 41663438 41663898
P19_NANOG_CNCC_D5_mem_q10_srt_rmdup_peaks_peak_19125
chr21 41707554 41708169
P19_NANOG_CNCC_D5_mem_q10_srt_rmdup_peaks_peak_19126
chr21 41758140 41758581
P19_NANOG_CNCC_D5_mem_q10_srt_rmdup_peaks_peak_19127
chr21 41995249 41996029
P19_NANOG_CNCC_D5_mem_q10_srt_rmdup_peaks_peak_19129
chr21 43655089 43655460
P19_NANOG_CNCC_D5_mem_q10_srt_rmdup_peaks_peak_19144
chr21 43944746 43945272
P19_NANOG_CNCC_D5_mem_q10_srt_rmdup_peaks_peak_19147
chr21 44938605 44938970
P19_NANOG_CNCC_D5_mem_q10_srt_rmdup_peaks_peak_19159
chr21 45089645 45090145
P19_NANOG_CNCC_D5_mem_q10_srt_rmdup_peaks_peak_19162
chr21 45148543 45148814
P19_NANOG_CNCC_D5_mem_q10_srt_rmdup_peaks_peak_19164
chr21 46124686 46125071
P19_NANOG_CNCC_D5_mem_q10_srt_rmdup_peaks_peak_19172
chr21 46776744 46777159
P19_NANOG_CNCC_D5_mem_q10_srt_rmdup_peaks_peak_19185
chr21 47830926 47831368

P19_NANOG_CNCC_D5_mem_q10_srt_rmdup_peaks_peak_19194
chr21 48087033 48087346
P19_NANOG_CNCC_D5_mem_q10_srt_rmdup_peaks_peak_19197
chr22 18985619 18986070
P19_NANOG_CNCC_D5_mem_q10_srt_rmdup_peaks_peak_19220
chr22 20905314 20905670
P19_NANOG_CNCC_D5_mem_q10_srt_rmdup_peaks_peak_19244
chr22 22487444 22487937
P19_NANOG_CNCC_D5_mem_q10_srt_rmdup_peaks_peak_19255
chr22 22702396 22702834
P19_NANOG_CNCC_D5_mem_q10_srt_rmdup_peaks_peak_19257
chr22 23708349 23708620
P19_NANOG_CNCC_D5_mem_q10_srt_rmdup_peaks_peak_19265
chr22 25084242 25084595
P19_NANOG_CNCC_D5_mem_q10_srt_rmdup_peaks_peak_19280
chr22 25087026 25087594
P19_NANOG_CNCC_D5_mem_q10_srt_rmdup_peaks_peak_19281
chr22 25296019 25296316
P19_NANOG_CNCC_D5_mem_q10_srt_rmdup_peaks_peak_19284
chr22 26273844 26274348
P19_NANOG_CNCC_D5_mem_q10_srt_rmdup_peaks_peak_19293
chr22 27175051 27175379
P19_NANOG_CNCC_D5_mem_q10_srt_rmdup_peaks_peak_19319
chr22 27289602 27290070
P19_NANOG_CNCC_D5_mem_q10_srt_rmdup_peaks_peak_19321
chr22 27391559 27392096
P19_NANOG_CNCC_D5_mem_q10_srt_rmdup_peaks_peak_19326
chr22 28777806 28778235
P19_NANOG_CNCC_D5_mem_q10_srt_rmdup_peaks_peak_19351
chr22 29949415 29949896
P19_NANOG_CNCC_D5_mem_q10_srt_rmdup_peaks_peak_19370
chr22 30404470 30404771
P19_NANOG_CNCC_D5_mem_q10_srt_rmdup_peaks_peak_19383
chr22 30821472 30821743
P19_NANOG_CNCC_D5_mem_q10_srt_rmdup_peaks_peak_19387
chr22 31366255 31366617
P19_NANOG_CNCC_D5_mem_q10_srt_rmdup_peaks_peak_19397
chr22 32287345 32287616
P19_NANOG_CNCC_D5_mem_q10_srt_rmdup_peaks_peak_19407
chr22 33309568 33309941
P19_NANOG_CNCC_D5_mem_q10_srt_rmdup_peaks_peak_19416
chr22 34129578 34130119
P19_NANOG_CNCC_D5_mem_q10_srt_rmdup_peaks_peak_19421
chr22 34381425 34381696
P19_NANOG_CNCC_D5_mem_q10_srt_rmdup_peaks_peak_19429
chr22 34451725 34452057
P19_NANOG_CNCC_D5_mem_q10_srt_rmdup_peaks_peak_19431
chr22 34740360 34740887
P19_NANOG_CNCC_D5_mem_q10_srt_rmdup_peaks_peak_19435
chr22 35178921 35179192

P19_NANOG_CNCC_D5_mem_q10_srt_rmdup_peaks_peak_19437
chr22 36557758 36558097
P19_NANOG_CNCC_D5_mem_q10_srt_rmdup_peaks_peak_19459
chr22 36780830 36781537
P19_NANOG_CNCC_D5_mem_q10_srt_rmdup_peaks_peak_19464
chr22 36943010 36943307
P19_NANOG_CNCC_D5_mem_q10_srt_rmdup_peaks_peak_19470
chr22 36981857 36982170
P19_NANOG_CNCC_D5_mem_q10_srt_rmdup_peaks_peak_19471
chr22 37178117 37178429
P19_NANOG_CNCC_D5_mem_q10_srt_rmdup_peaks_peak_19475
chr22 37952750 37953226
P19_NANOG_CNCC_D5_mem_q10_srt_rmdup_peaks_peak_19487
chr22 38667861 38668302
P19_NANOG_CNCC_D5_mem_q10_srt_rmdup_peaks_peak_19495
chr22 39014346 39014783
P19_NANOG_CNCC_D5_mem_q10_srt_rmdup_peaks_peak_19502
chr22 39268183 39268454
P19_NANOG_CNCC_D5_mem_q10_srt_rmdup_peaks_peak_19507
chr22 39269890 39270329
P19_NANOG_CNCC_D5_mem_q10_srt_rmdup_peaks_peak_19508
chr22 39715534 39715829
P19_NANOG_CNCC_D5_mem_q10_srt_rmdup_peaks_peak_19520
chr22 40573024 40573399
P19_NANOG_CNCC_D5_mem_q10_srt_rmdup_peaks_peak_19534
chr22 41032578 41032901
P19_NANOG_CNCC_D5_mem_q10_srt_rmdup_peaks_peak_19540
chr22 41079024 41079334
P19_NANOG_CNCC_D5_mem_q10_srt_rmdup_peaks_peak_19541
chr22 41842743 41843103
P19_NANOG_CNCC_D5_mem_q10_srt_rmdup_peaks_peak_19555
chr22 42159196 42159467
P19_NANOG_CNCC_D5_mem_q10_srt_rmdup_peaks_peak_19563
chr22 43378604 43379001
P19_NANOG_CNCC_D5_mem_q10_srt_rmdup_peaks_peak_19594
chr22 43932721 43933047
P19_NANOG_CNCC_D5_mem_q10_srt_rmdup_peaks_peak_19599
chr22 45367935 45368302
P19_NANOG_CNCC_D5_mem_q10_srt_rmdup_peaks_peak_19619
chr22 45410447 45411110
P19_NANOG_CNCC_D5_mem_q10_srt_rmdup_peaks_peak_19621
chr22 45486436 45486707
P19_NANOG_CNCC_D5_mem_q10_srt_rmdup_peaks_peak_19624
chr22 46167727 46168156
P19_NANOG_CNCC_D5_mem_q10_srt_rmdup_peaks_peak_19634
chr22 46457226 46457729
P19_NANOG_CNCC_D5_mem_q10_srt_rmdup_peaks_peak_19638
chr22 47262576 47262915
P19_NANOG_CNCC_D5_mem_q10_srt_rmdup_peaks_peak_19654
chr22 48468510 48469088

P19_NANOG_CNCC_D5_mem_q10_srt_rmdup_peaks_peak_19660
chr22	48480053	48480529
P19_NANOG_CNCC_D5_mem_q10_srt_rmdup_peaks_peak_19661
chr22	48614644	48614960
P19_NANOG_CNCC_D5_mem_q10_srt_rmdup_peaks_peak_19662
chr22	48635232	48636293
P19_NANOG_CNCC_D5_mem_q10_srt_rmdup_peaks_peak_19663
chr22	48765592	48766346
P19_NANOG_CNCC_D5_mem_q10_srt_rmdup_peaks_peak_19664
chr22	49178640	49179002
P19_NANOG_CNCC_D5_mem_q10_srt_rmdup_peaks_peak_19665
chr22	49731991	49732291
P19_NANOG_CNCC_D5_mem_q10_srt_rmdup_peaks_peak_19666
chr22	50354048	50354319
P19_NANOG_CNCC_D5_mem_q10_srt_rmdup_peaks_peak_19668
chr22	50446479	50447051
P19_NANOG_CNCC_D5_mem_q10_srt_rmdup_peaks_peak_19670
chr22	50781599	50781922
P19_NANOG_CNCC_D5_mem_q10_srt_rmdup_peaks_peak_19674
chr3	4949275	4949635
P19_NANOG_CNCC_D5_mem_q10_srt_rmdup_peaks_peak_19704
chr3	8155387	8155795
P19_NANOG_CNCC_D5_mem_q10_srt_rmdup_peaks_peak_19724
chr3	9166814	9167140
P19_NANOG_CNCC_D5_mem_q10_srt_rmdup_peaks_peak_19742
chr3	9246469	9246740
P19_NANOG_CNCC_D5_mem_q10_srt_rmdup_peaks_peak_19745
chr3	9996781	9997105
P19_NANOG_CNCC_D5_mem_q10_srt_rmdup_peaks_peak_19758
chr3	10501864	10502184
P19_NANOG_CNCC_D5_mem_q10_srt_rmdup_peaks_peak_19766
chr3	10716205	10716605
P19_NANOG_CNCC_D5_mem_q10_srt_rmdup_peaks_peak_19768
chr3	11024932	11025566
P19_NANOG_CNCC_D5_mem_q10_srt_rmdup_peaks_peak_19771
chr3	11660470	11660783
P19_NANOG_CNCC_D5_mem_q10_srt_rmdup_peaks_peak_19778
chr3	11758390	11758726
P19_NANOG_CNCC_D5_mem_q10_srt_rmdup_peaks_peak_19781
chr3	14339415	14339874
P19_NANOG_CNCC_D5_mem_q10_srt_rmdup_peaks_peak_19805
chr3	15671619	15671904
P19_NANOG_CNCC_D5_mem_q10_srt_rmdup_peaks_peak_19827
chr3	16650302	16650641
P19_NANOG_CNCC_D5_mem_q10_srt_rmdup_peaks_peak_19839
chr3	17054618	17054921
P19_NANOG_CNCC_D5_mem_q10_srt_rmdup_peaks_peak_19853
chr3	17274898	17275169
P19_NANOG_CNCC_D5_mem_q10_srt_rmdup_peaks_peak_19857
chr3	17626828	17627335

P19_NANOG_CNCC_D5_mem_q10_srt_rmdup_peaks_peak_19863
chr3 18140336 18140681
P19_NANOG_CNCC_D5_mem_q10_srt_rmdup_peaks_peak_19865
chr3 19785843 19786269
P19_NANOG_CNCC_D5_mem_q10_srt_rmdup_peaks_peak_19884
chr3 20082348 20082619
P19_NANOG_CNCC_D5_mem_q10_srt_rmdup_peaks_peak_19888
chr3 20958743 20959035
P19_NANOG_CNCC_D5_mem_q10_srt_rmdup_peaks_peak_19897
chr3 21544248 21544530
P19_NANOG_CNCC_D5_mem_q10_srt_rmdup_peaks_peak_19900
chr3 23077419 23077790
P19_NANOG_CNCC_D5_mem_q10_srt_rmdup_peaks_peak_19904
chr3 23282827 23283173
P19_NANOG_CNCC_D5_mem_q10_srt_rmdup_peaks_peak_19908
chr3 23436143 23436576
P19_NANOG_CNCC_D5_mem_q10_srt_rmdup_peaks_peak_19911
chr3 23686257 23686792
P19_NANOG_CNCC_D5_mem_q10_srt_rmdup_peaks_peak_19916
chr3 23688997 23689570
P19_NANOG_CNCC_D5_mem_q10_srt_rmdup_peaks_peak_19917
chr3 23851760 23852031
P19_NANOG_CNCC_D5_mem_q10_srt_rmdup_peaks_peak_19923
chr3 23958622 23958928
P19_NANOG_CNCC_D5_mem_q10_srt_rmdup_peaks_peak_19925
chr3 24815322 24815641
P19_NANOG_CNCC_D5_mem_q10_srt_rmdup_peaks_peak_19939
chr3 25028085 25028487
P19_NANOG_CNCC_D5_mem_q10_srt_rmdup_peaks_peak_19942
chr3 25179069 25179340
P19_NANOG_CNCC_D5_mem_q10_srt_rmdup_peaks_peak_19943
chr3 25322840 25323272
P19_NANOG_CNCC_D5_mem_q10_srt_rmdup_peaks_peak_19945
chr3 27924662 27925098
P19_NANOG_CNCC_D5_mem_q10_srt_rmdup_peaks_peak_19984
chr3 28546524 28546804
P19_NANOG_CNCC_D5_mem_q10_srt_rmdup_peaks_peak_19990
chr3 29910118 29910528
P19_NANOG_CNCC_D5_mem_q10_srt_rmdup_peaks_peak_20001
chr3 30228192 30228476
P19_NANOG_CNCC_D5_mem_q10_srt_rmdup_peaks_peak_20007
chr3 30743995 30744294
P19_NANOG_CNCC_D5_mem_q10_srt_rmdup_peaks_peak_20013
chr3 30950056 30950411
P19_NANOG_CNCC_D5_mem_q10_srt_rmdup_peaks_peak_20015
chr3 31082652 31083165
P19_NANOG_CNCC_D5_mem_q10_srt_rmdup_peaks_peak_20018
chr3 31182861 31183195
P19_NANOG_CNCC_D5_mem_q10_srt_rmdup_peaks_peak_20020
chr3 31330078 31330729

P19_NANOG_CNCC_D5_mem_q10_srt_rmdup_peaks_peak_20022
chr3 31391927 31392445
P19_NANOG_CNCC_D5_mem_q10_srt_rmdup_peaks_peak_20024
chr3 31720016 31720287
P19_NANOG_CNCC_D5_mem_q10_srt_rmdup_peaks_peak_20027
chr3 31938184 31938707
P19_NANOG_CNCC_D5_mem_q10_srt_rmdup_peaks_peak_20030
chr3 32904062 32904388
P19_NANOG_CNCC_D5_mem_q10_srt_rmdup_peaks_peak_20054
chr3 33297836 33298107
P19_NANOG_CNCC_D5_mem_q10_srt_rmdup_peaks_peak_20064
chr3 34960369 34960939
P19_NANOG_CNCC_D5_mem_q10_srt_rmdup_peaks_peak_20079
chr3 36990766 36991163
P19_NANOG_CNCC_D5_mem_q10_srt_rmdup_peaks_peak_20083
chr3 39447936 39448224
P19_NANOG_CNCC_D5_mem_q10_srt_rmdup_peaks_peak_20104
chr3 40063905 40064378
P19_NANOG_CNCC_D5_mem_q10_srt_rmdup_peaks_peak_20109
chr3 40518478 40518851
P19_NANOG_CNCC_D5_mem_q10_srt_rmdup_peaks_peak_20113
chr3 40626113 40626625
P19_NANOG_CNCC_D5_mem_q10_srt_rmdup_peaks_peak_20114
chr3 40790512 40790822
P19_NANOG_CNCC_D5_mem_q10_srt_rmdup_peaks_peak_20116
chr3 41042794 41043134
P19_NANOG_CNCC_D5_mem_q10_srt_rmdup_peaks_peak_20118
chr3 41075498 41075783
P19_NANOG_CNCC_D5_mem_q10_srt_rmdup_peaks_peak_20119
chr3 41407304 41407861
P19_NANOG_CNCC_D5_mem_q10_srt_rmdup_peaks_peak_20125
chr3 41723009 41723296
P19_NANOG_CNCC_D5_mem_q10_srt_rmdup_peaks_peak_20129
chr3 41783794 41784065
P19_NANOG_CNCC_D5_mem_q10_srt_rmdup_peaks_peak_20130
chr3 42702258 42702783
P19_NANOG_CNCC_D5_mem_q10_srt_rmdup_peaks_peak_20140
chr3 42925876 42926174
P19_NANOG_CNCC_D5_mem_q10_srt_rmdup_peaks_peak_20146
chr3 43614198 43614497
P19_NANOG_CNCC_D5_mem_q10_srt_rmdup_peaks_peak_20156
chr3 44008992 44009291
P19_NANOG_CNCC_D5_mem_q10_srt_rmdup_peaks_peak_20159
chr3 44277277 44277681
P19_NANOG_CNCC_D5_mem_q10_srt_rmdup_peaks_peak_20166
chr3 44407442 44407769
P19_NANOG_CNCC_D5_mem_q10_srt_rmdup_peaks_peak_20167
chr3 44770955 44771309
P19_NANOG_CNCC_D5_mem_q10_srt_rmdup_peaks_peak_20169
chr3 44806871 44807142

P19_NANOG_CNCC_D5_mem_q10_srt_rmdup_peaks_peak_20171
chr3 44973654 44974191
P19_NANOG_CNCC_D5_mem_q10_srt_rmdup_peaks_peak_20173
chr3 45483385 45483690
P19_NANOG_CNCC_D5_mem_q10_srt_rmdup_peaks_peak_20181
chr3 45730447 45730738
P19_NANOG_CNCC_D5_mem_q10_srt_rmdup_peaks_peak_20189
chr3 46352625 46352902
P19_NANOG_CNCC_D5_mem_q10_srt_rmdup_peaks_peak_20193
chr3 47517319 47517590
P19_NANOG_CNCC_D5_mem_q10_srt_rmdup_peaks_peak_20209
chr3 47765243 47765550
P19_NANOG_CNCC_D5_mem_q10_srt_rmdup_peaks_peak_20212
chr3 48544942 48545213
P19_NANOG_CNCC_D5_mem_q10_srt_rmdup_peaks_peak_20230
chr3 49306798 49307191
P19_NANOG_CNCC_D5_mem_q10_srt_rmdup_peaks_peak_20238
chr3 49459648 49459919
P19_NANOG_CNCC_D5_mem_q10_srt_rmdup_peaks_peak_20239
chr3 49467065 49467370
P19_NANOG_CNCC_D5_mem_q10_srt_rmdup_peaks_peak_20240
chr3 50491427 50491736
P19_NANOG_CNCC_D5_mem_q10_srt_rmdup_peaks_peak_20262
chr3 50576379 50576843
P19_NANOG_CNCC_D5_mem_q10_srt_rmdup_peaks_peak_20265
chr3 50991322 50991611
P19_NANOG_CNCC_D5_mem_q10_srt_rmdup_peaks_peak_20275
chr3 51740933 51741206
P19_NANOG_CNCC_D5_mem_q10_srt_rmdup_peaks_peak_20282
chr3 51753146 51753427
P19_NANOG_CNCC_D5_mem_q10_srt_rmdup_peaks_peak_20283
chr3 51989713 51990090
P19_NANOG_CNCC_D5_mem_q10_srt_rmdup_peaks_peak_20286
chr3 52125223 52125614
P19_NANOG_CNCC_D5_mem_q10_srt_rmdup_peaks_peak_20291
chr3 53064924 53065351
P19_NANOG_CNCC_D5_mem_q10_srt_rmdup_peaks_peak_20296
chr3 53512898 53513317
P19_NANOG_CNCC_D5_mem_q10_srt_rmdup_peaks_peak_20303
chr3 53545909 53546262
P19_NANOG_CNCC_D5_mem_q10_srt_rmdup_peaks_peak_20305
chr3 53778545 53778914
P19_NANOG_CNCC_D5_mem_q10_srt_rmdup_peaks_peak_20309
chr3 53879545 53879926
P19_NANOG_CNCC_D5_mem_q10_srt_rmdup_peaks_peak_20311
chr3 54262987 54263271
P19_NANOG_CNCC_D5_mem_q10_srt_rmdup_peaks_peak_20317
chr3 54665525 54666172
P19_NANOG_CNCC_D5_mem_q10_srt_rmdup_peaks_peak_20318
chr3 54970315 54970721

P19_NANOG_CNCC_D5_mem_q10_srt_rmdup_peaks_peak_20327
chr3 55142830 55143240
P19_NANOG_CNCC_D5_mem_q10_srt_rmdup_peaks_peak_20331
chr3 55236779 55237247
P19_NANOG_CNCC_D5_mem_q10_srt_rmdup_peaks_peak_20332
chr3 55876183 55876465
P19_NANOG_CNCC_D5_mem_q10_srt_rmdup_peaks_peak_20341
chr3 56288205 56288489
P19_NANOG_CNCC_D5_mem_q10_srt_rmdup_peaks_peak_20350
chr3 56559220 56559491
P19_NANOG_CNCC_D5_mem_q10_srt_rmdup_peaks_peak_20355
chr3 57094445 57094765
P19_NANOG_CNCC_D5_mem_q10_srt_rmdup_peaks_peak_20359
chr3 57236834 57237261
P19_NANOG_CNCC_D5_mem_q10_srt_rmdup_peaks_peak_20370
chr3 57810975 57811261
P19_NANOG_CNCC_D5_mem_q10_srt_rmdup_peaks_peak_20377
chr3 57813111 57813476
P19_NANOG_CNCC_D5_mem_q10_srt_rmdup_peaks_peak_20378
chr3 57969890 57970258
P19_NANOG_CNCC_D5_mem_q10_srt_rmdup_peaks_peak_20381
chr3 58941021 58941473
P19_NANOG_CNCC_D5_mem_q10_srt_rmdup_peaks_peak_20396
chr3 59426367 59426720
P19_NANOG_CNCC_D5_mem_q10_srt_rmdup_peaks_peak_20399
chr3 60645734 60646173
P19_NANOG_CNCC_D5_mem_q10_srt_rmdup_peaks_peak_20409
chr3 61041925 61042416
P19_NANOG_CNCC_D5_mem_q10_srt_rmdup_peaks_peak_20414
chr3 61091534 61091868
P19_NANOG_CNCC_D5_mem_q10_srt_rmdup_peaks_peak_20416
chr3 61178522 61178913
P19_NANOG_CNCC_D5_mem_q10_srt_rmdup_peaks_peak_20419
chr3 61281176 61281501
P19_NANOG_CNCC_D5_mem_q10_srt_rmdup_peaks_peak_20420
chr3 61285067 61285442
P19_NANOG_CNCC_D5_mem_q10_srt_rmdup_peaks_peak_20421
chr3 61507782 61508085
P19_NANOG_CNCC_D5_mem_q10_srt_rmdup_peaks_peak_20423
chr3 61694430 61694825
P19_NANOG_CNCC_D5_mem_q10_srt_rmdup_peaks_peak_20434
chr3 61711300 61711726
P19_NANOG_CNCC_D5_mem_q10_srt_rmdup_peaks_peak_20435
chr3 61727267 61727664
P19_NANOG_CNCC_D5_mem_q10_srt_rmdup_peaks_peak_20436
chr3 62128205 62128514
P19_NANOG_CNCC_D5_mem_q10_srt_rmdup_peaks_peak_20447
chr3 62363076 62363395
P19_NANOG_CNCC_D5_mem_q10_srt_rmdup_peaks_peak_20455
chr3 62524400 62524671

P19_NANOG_CNCC_D5_mem_q10_srt_rmdup_peaks_peak_20461
chr3 62990956 62991239
P19_NANOG_CNCC_D5_mem_q10_srt_rmdup_peaks_peak_20470
chr3 63152696 63153120
P19_NANOG_CNCC_D5_mem_q10_srt_rmdup_peaks_peak_20475
chr3 63569398 63569669
P19_NANOG_CNCC_D5_mem_q10_srt_rmdup_peaks_peak_20481
chr3 63847037 63847308
P19_NANOG_CNCC_D5_mem_q10_srt_rmdup_peaks_peak_20488
chr3 63950371 63950779
P19_NANOG_CNCC_D5_mem_q10_srt_rmdup_peaks_peak_20489
chr3 64092670 64093038
P19_NANOG_CNCC_D5_mem_q10_srt_rmdup_peaks_peak_20495
chr3 64128885 64129342
P19_NANOG_CNCC_D5_mem_q10_srt_rmdup_peaks_peak_20497
chr3 64264345 64264627
P19_NANOG_CNCC_D5_mem_q10_srt_rmdup_peaks_peak_20504
chr3 64491115 64491453
P19_NANOG_CNCC_D5_mem_q10_srt_rmdup_peaks_peak_20508
chr3 64811209 64811635
P19_NANOG_CNCC_D5_mem_q10_srt_rmdup_peaks_peak_20514
chr3 65535858 65536153
P19_NANOG_CNCC_D5_mem_q10_srt_rmdup_peaks_peak_20528
chr3 65590546 65590849
P19_NANOG_CNCC_D5_mem_q10_srt_rmdup_peaks_peak_20530
chr3 66052718 66053158
P19_NANOG_CNCC_D5_mem_q10_srt_rmdup_peaks_peak_20549
chr3 66342914 66343260
P19_NANOG_CNCC_D5_mem_q10_srt_rmdup_peaks_peak_20552
chr3 66660314 66660638
P19_NANOG_CNCC_D5_mem_q10_srt_rmdup_peaks_peak_20565
chr3 67048484 67048797
P19_NANOG_CNCC_D5_mem_q10_srt_rmdup_peaks_peak_20571
chr3 68868359 68868763
P19_NANOG_CNCC_D5_mem_q10_srt_rmdup_peaks_peak_20579
chr3 69819470 69820020
P19_NANOG_CNCC_D5_mem_q10_srt_rmdup_peaks_peak_20596
chr3 69820523 69820794
P19_NANOG_CNCC_D5_mem_q10_srt_rmdup_peaks_peak_20597
chr3 70120623 70121066
P19_NANOG_CNCC_D5_mem_q10_srt_rmdup_peaks_peak_20602
chr3 70125605 70125929
P19_NANOG_CNCC_D5_mem_q10_srt_rmdup_peaks_peak_20603
chr3 71136490 71136772
P19_NANOG_CNCC_D5_mem_q10_srt_rmdup_peaks_peak_20616
chr3 71517518 71517967
P19_NANOG_CNCC_D5_mem_q10_srt_rmdup_peaks_peak_20622
chr3 71721604 71721875
P19_NANOG_CNCC_D5_mem_q10_srt_rmdup_peaks_peak_20625
chr3 71752906 71753482

P19_NANOG_CNCC_D5_mem_q10_srt_rmdup_peaks_peak_20627
chr3 71926054 71926370
P19_NANOG_CNCC_D5_mem_q10_srt_rmdup_peaks_peak_20633
chr3 72464364 72464710
P19_NANOG_CNCC_D5_mem_q10_srt_rmdup_peaks_peak_20641
chr3 72540008 72540518
P19_NANOG_CNCC_D5_mem_q10_srt_rmdup_peaks_peak_20643
chr3 73006335 73006833
P19_NANOG_CNCC_D5_mem_q10_srt_rmdup_peaks_peak_20656
chr3 73607762 73608066
P19_NANOG_CNCC_D5_mem_q10_srt_rmdup_peaks_peak_20665
chr3 73673369 73673640
P19_NANOG_CNCC_D5_mem_q10_srt_rmdup_peaks_peak_20666
chr3 74003731 74004124
P19_NANOG_CNCC_D5_mem_q10_srt_rmdup_peaks_peak_20672
chr3 74464868 74465229
P19_NANOG_CNCC_D5_mem_q10_srt_rmdup_peaks_peak_20674
chr3 75888919 75889550
P19_NANOG_CNCC_D5_mem_q10_srt_rmdup_peaks_peak_20678
chr3 76705931 76706301
P19_NANOG_CNCC_D5_mem_q10_srt_rmdup_peaks_peak_20682
chr3 76962818 76963142
P19_NANOG_CNCC_D5_mem_q10_srt_rmdup_peaks_peak_20685
chr3 76979863 76980152
P19_NANOG_CNCC_D5_mem_q10_srt_rmdup_peaks_peak_20686
chr3 77101339 77101757
P19_NANOG_CNCC_D5_mem_q10_srt_rmdup_peaks_peak_20690
chr3 77330892 77331188
P19_NANOG_CNCC_D5_mem_q10_srt_rmdup_peaks_peak_20692
chr3 77542656 77542984
P19_NANOG_CNCC_D5_mem_q10_srt_rmdup_peaks_peak_20696
chr3 77833400 77833752
P19_NANOG_CNCC_D5_mem_q10_srt_rmdup_peaks_peak_20699
chr3 78146214 78146551
P19_NANOG_CNCC_D5_mem_q10_srt_rmdup_peaks_peak_20705
chr3 78556541 78556925
P19_NANOG_CNCC_D5_mem_q10_srt_rmdup_peaks_peak_20713
chr3 78842596 78842923
P19_NANOG_CNCC_D5_mem_q10_srt_rmdup_peaks_peak_20715
chr3 78962904 78963306
P19_NANOG_CNCC_D5_mem_q10_srt_rmdup_peaks_peak_20716
chr3 79035872 79036216
P19_NANOG_CNCC_D5_mem_q10_srt_rmdup_peaks_peak_20717
chr3 79227757 79228294
P19_NANOG_CNCC_D5_mem_q10_srt_rmdup_peaks_peak_20721
chr3 79721017 79721514
P19_NANOG_CNCC_D5_mem_q10_srt_rmdup_peaks_peak_20723
chr3 80802196 80802751
P19_NANOG_CNCC_D5_mem_q10_srt_rmdup_peaks_peak_20729
chr3 81298582 81298879

P19_NANOG_CNCC_D5_mem_q10_srt_rmdup_peaks_peak_20732
chr3 81913442 81913788
P19_NANOG_CNCC_D5_mem_q10_srt_rmdup_peaks_peak_20738
chr3 82014878 82015200
P19_NANOG_CNCC_D5_mem_q10_srt_rmdup_peaks_peak_20740
chr3 83027032 83027440
P19_NANOG_CNCC_D5_mem_q10_srt_rmdup_peaks_peak_20744
chr3 83623262 83623723
P19_NANOG_CNCC_D5_mem_q10_srt_rmdup_peaks_peak_20747
chr3 83950546 83950953
P19_NANOG_CNCC_D5_mem_q10_srt_rmdup_peaks_peak_20748
chr3 84005404 84005841
P19_NANOG_CNCC_D5_mem_q10_srt_rmdup_peaks_peak_20749
chr3 84600687 84601008
P19_NANOG_CNCC_D5_mem_q10_srt_rmdup_peaks_peak_20754
chr3 85984379 85984710
P19_NANOG_CNCC_D5_mem_q10_srt_rmdup_peaks_peak_20771
chr3 86151702 86152087
P19_NANOG_CNCC_D5_mem_q10_srt_rmdup_peaks_peak_20772
chr3 93838252 93838582
P19_NANOG_CNCC_D5_mem_q10_srt_rmdup_peaks_peak_20805
chr3 94723104 94723397
P19_NANOG_CNCC_D5_mem_q10_srt_rmdup_peaks_peak_20809
chr3 97858414 97858796
P19_NANOG_CNCC_D5_mem_q10_srt_rmdup_peaks_peak_20824
chr3 98045325 98045777
P19_NANOG_CNCC_D5_mem_q10_srt_rmdup_peaks_peak_20827
chr3 98535269 98535624
P19_NANOG_CNCC_D5_mem_q10_srt_rmdup_peaks_peak_20830
chr3 99614613 99614940
P19_NANOG_CNCC_D5_mem_q10_srt_rmdup_peaks_peak_20838
chr3 100970689 100971058
P19_NANOG_CNCC_D5_mem_q10_srt_rmdup_peaks_peak_20856
chr3 102396608 102397071
P19_NANOG_CNCC_D5_mem_q10_srt_rmdup_peaks_peak_20866
chr3 103230803 103231420
P19_NANOG_CNCC_D5_mem_q10_srt_rmdup_peaks_peak_20868
chr3 103537587 103538045
P19_NANOG_CNCC_D5_mem_q10_srt_rmdup_peaks_peak_20871
chr3 105563378 105563755
P19_NANOG_CNCC_D5_mem_q10_srt_rmdup_peaks_peak_20888
chr3 106014869 106015194
P19_NANOG_CNCC_D5_mem_q10_srt_rmdup_peaks_peak_20893
chr3 107736591 107736862
P19_NANOG_CNCC_D5_mem_q10_srt_rmdup_peaks_peak_20911
chr3 108689453 108689809
P19_NANOG_CNCC_D5_mem_q10_srt_rmdup_peaks_peak_20916
chr3 109035242 109035638
P19_NANOG_CNCC_D5_mem_q10_srt_rmdup_peaks_peak_20920
chr3 110245683 110246046

P19_NANOG_CNCC_D5_mem_q10_srt_rmdup_peaks_peak_20927
chr3 110795581 110795944
P19_NANOG_CNCC_D5_mem_q10_srt_rmdup_peaks_peak_20936
chr3 110929714 110930258
P19_NANOG_CNCC_D5_mem_q10_srt_rmdup_peaks_peak_20939
chr3 110948058 110948426
P19_NANOG_CNCC_D5_mem_q10_srt_rmdup_peaks_peak_20940
chr3 111236735 111237008
P19_NANOG_CNCC_D5_mem_q10_srt_rmdup_peaks_peak_20942
chr3 111767582 111768212
P19_NANOG_CNCC_D5_mem_q10_srt_rmdup_peaks_peak_20950
chr3 111771997 111772321
P19_NANOG_CNCC_D5_mem_q10_srt_rmdup_peaks_peak_20951
chr3 112054242 112054558
P19_NANOG_CNCC_D5_mem_q10_srt_rmdup_peaks_peak_20958
chr3 112353901 112354478
P19_NANOG_CNCC_D5_mem_q10_srt_rmdup_peaks_peak_20966
chr3 112543462 112543775
P19_NANOG_CNCC_D5_mem_q10_srt_rmdup_peaks_peak_20970
chr3 112961937 112962361
P19_NANOG_CNCC_D5_mem_q10_srt_rmdup_peaks_peak_20977
chr3 114052249 114053003
P19_NANOG_CNCC_D5_mem_q10_srt_rmdup_peaks_peak_20993
chr3 114579789 114580082
P19_NANOG_CNCC_D5_mem_q10_srt_rmdup_peaks_peak_20999
chr3 115273169 115273520
P19_NANOG_CNCC_D5_mem_q10_srt_rmdup_peaks_peak_21009
chr3 115345936 115346242
P19_NANOG_CNCC_D5_mem_q10_srt_rmdup_peaks_peak_21012
chr3 116560676 116560992
P19_NANOG_CNCC_D5_mem_q10_srt_rmdup_peaks_peak_21032
chr3 116827752 116828110
P19_NANOG_CNCC_D5_mem_q10_srt_rmdup_peaks_peak_21037
chr3 117308361 117308710
P19_NANOG_CNCC_D5_mem_q10_srt_rmdup_peaks_peak_21044
chr3 117329944 117330263
P19_NANOG_CNCC_D5_mem_q10_srt_rmdup_peaks_peak_21045
chr3 117411205 117411671
P19_NANOG_CNCC_D5_mem_q10_srt_rmdup_peaks_peak_21047
chr3 117520774 117521082
P19_NANOG_CNCC_D5_mem_q10_srt_rmdup_peaks_peak_21050
chr3 117539769 117540150
P19_NANOG_CNCC_D5_mem_q10_srt_rmdup_peaks_peak_21051
chr3 118197773 118198168
P19_NANOG_CNCC_D5_mem_q10_srt_rmdup_peaks_peak_21054
chr3 118753624 118754003
P19_NANOG_CNCC_D5_mem_q10_srt_rmdup_peaks_peak_21058
chr3 120167665 120168046
P19_NANOG_CNCC_D5_mem_q10_srt_rmdup_peaks_peak_21073
chr3 120217602 120217911

P19_NANOG_CNCC_D5_mem_q10_srt_rmdup_peaks_peak_21074
chr3 121724805 121725082
P19_NANOG_CNCC_D5_mem_q10_srt_rmdup_peaks_peak_21089
chr3 122512695 122512966
P19_NANOG_CNCC_D5_mem_q10_srt_rmdup_peaks_peak_21099
chr3 122693643 122693914
P19_NANOG_CNCC_D5_mem_q10_srt_rmdup_peaks_peak_21100
chr3 122740209 122740593
P19_NANOG_CNCC_D5_mem_q10_srt_rmdup_peaks_peak_21103
chr3 123485607 123486216
P19_NANOG_CNCC_D5_mem_q10_srt_rmdup_peaks_peak_21115
chr3 123580384 123580713
P19_NANOG_CNCC_D5_mem_q10_srt_rmdup_peaks_peak_21117
chr3 123914369 123914675
P19_NANOG_CNCC_D5_mem_q10_srt_rmdup_peaks_peak_21120
chr3 124264752 124265108
P19_NANOG_CNCC_D5_mem_q10_srt_rmdup_peaks_peak_21124
chr3 125059007 125059528
P19_NANOG_CNCC_D5_mem_q10_srt_rmdup_peaks_peak_21135
chr3 127122839 127123646
P19_NANOG_CNCC_D5_mem_q10_srt_rmdup_peaks_peak_21161
chr3 127819054 127819596
P19_NANOG_CNCC_D5_mem_q10_srt_rmdup_peaks_peak_21171
chr3 127842650 127842944
P19_NANOG_CNCC_D5_mem_q10_srt_rmdup_peaks_peak_21172
chr3 127886660 127887055
P19_NANOG_CNCC_D5_mem_q10_srt_rmdup_peaks_peak_21173
chr3 129107613 129107927
P19_NANOG_CNCC_D5_mem_q10_srt_rmdup_peaks_peak_21196
chr3 129118508 129118805
P19_NANOG_CNCC_D5_mem_q10_srt_rmdup_peaks_peak_21197
chr3 129167065 129167421
P19_NANOG_CNCC_D5_mem_q10_srt_rmdup_peaks_peak_21199
chr3 131633129 131633415
P19_NANOG_CNCC_D5_mem_q10_srt_rmdup_peaks_peak_21227
chr3 131854692 131855321
P19_NANOG_CNCC_D5_mem_q10_srt_rmdup_peaks_peak_21230
chr3 132598599 132599094
P19_NANOG_CNCC_D5_mem_q10_srt_rmdup_peaks_peak_21237
chr3 133291640 133292118
P19_NANOG_CNCC_D5_mem_q10_srt_rmdup_peaks_peak_21242
chr3 134568894 134569178
P19_NANOG_CNCC_D5_mem_q10_srt_rmdup_peaks_peak_21261
chr3 135412998 135413355
P19_NANOG_CNCC_D5_mem_q10_srt_rmdup_peaks_peak_21274
chr3 135882559 135882830
P19_NANOG_CNCC_D5_mem_q10_srt_rmdup_peaks_peak_21281
chr3 136893483 136894071
P19_NANOG_CNCC_D5_mem_q10_srt_rmdup_peaks_peak_21294
chr3 137366093 137366538

P19_NANOG_CNCC_D5_mem_q10_srt_rmdup_peaks_peak_21298
chr3	137567086	137567569
P19_NANOG_CNCC_D5_mem_q10_srt_rmdup_peaks_peak_21308
chr3	139465407	139465681
P19_NANOG_CNCC_D5_mem_q10_srt_rmdup_peaks_peak_21339
chr3	140430223	140430789
P19_NANOG_CNCC_D5_mem_q10_srt_rmdup_peaks_peak_21351
chr3	140957830	140958143
P19_NANOG_CNCC_D5_mem_q10_srt_rmdup_peaks_peak_21361
chr3	141087076	141087347
P19_NANOG_CNCC_D5_mem_q10_srt_rmdup_peaks_peak_21362
chr3	141337865	141338227
P19_NANOG_CNCC_D5_mem_q10_srt_rmdup_peaks_peak_21367
chr3	143712378	143712847
P19_NANOG_CNCC_D5_mem_q10_srt_rmdup_peaks_peak_21386
chr3	144704757	144705065
P19_NANOG_CNCC_D5_mem_q10_srt_rmdup_peaks_peak_21394
chr3	144828928	144829319
P19_NANOG_CNCC_D5_mem_q10_srt_rmdup_peaks_peak_21395
chr3	145399010	145399374
P19_NANOG_CNCC_D5_mem_q10_srt_rmdup_peaks_peak_21399
chr3	145621055	145621389
P19_NANOG_CNCC_D5_mem_q10_srt_rmdup_peaks_peak_21403
chr3	145793004	145793409
P19_NANOG_CNCC_D5_mem_q10_srt_rmdup_peaks_peak_21404
chr3	145878915	145879271
P19_NANOG_CNCC_D5_mem_q10_srt_rmdup_peaks_peak_21406
chr3	148675462	148675739
P19_NANOG_CNCC_D5_mem_q10_srt_rmdup_peaks_peak_21450
chr3	148963949	148964229
P19_NANOG_CNCC_D5_mem_q10_srt_rmdup_peaks_peak_21456
chr3	149061791	149062163
P19_NANOG_CNCC_D5_mem_q10_srt_rmdup_peaks_peak_21458
chr3	149293911	149294261
P19_NANOG_CNCC_D5_mem_q10_srt_rmdup_peaks_peak_21464
chr3	149853277	149853559
P19_NANOG_CNCC_D5_mem_q10_srt_rmdup_peaks_peak_21476
chr3	150688834	150689162
P19_NANOG_CNCC_D5_mem_q10_srt_rmdup_peaks_peak_21487
chr3	150803459	150803776
P19_NANOG_CNCC_D5_mem_q10_srt_rmdup_peaks_peak_21489
chr3	151195844	151196209
P19_NANOG_CNCC_D5_mem_q10_srt_rmdup_peaks_peak_21495
chr3	152120947	152121279
P19_NANOG_CNCC_D5_mem_q10_srt_rmdup_peaks_peak_21506
chr3	153360259	153360799
P19_NANOG_CNCC_D5_mem_q10_srt_rmdup_peaks_peak_21516
chr3	154494172	154494509
P19_NANOG_CNCC_D5_mem_q10_srt_rmdup_peaks_peak_21523
chr3	154546144	154546437

P19_NANOG_CNCC_D5_mem_q10_srt_rmdup_peaks_peak_21524
chr3 156704600 156705290
P19_NANOG_CNCC_D5_mem_q10_srt_rmdup_peaks_peak_21548
chr3 157105801 157106149
P19_NANOG_CNCC_D5_mem_q10_srt_rmdup_peaks_peak_21561
chr3 157963367 157963696
P19_NANOG_CNCC_D5_mem_q10_srt_rmdup_peaks_peak_21567
chr3 159337144 159337458
P19_NANOG_CNCC_D5_mem_q10_srt_rmdup_peaks_peak_21579
chr3 159481549 159481874
P19_NANOG_CNCC_D5_mem_q10_srt_rmdup_peaks_peak_21581
chr3 160822268 160822618
P19_NANOG_CNCC_D5_mem_q10_srt_rmdup_peaks_peak_21594
chr3 161089191 161089691
P19_NANOG_CNCC_D5_mem_q10_srt_rmdup_peaks_peak_21595
chr3 161360538 161360903
P19_NANOG_CNCC_D5_mem_q10_srt_rmdup_peaks_peak_21598
chr3 163497160 163497528
P19_NANOG_CNCC_D5_mem_q10_srt_rmdup_peaks_peak_21608
chr3 166444368 166444793
P19_NANOG_CNCC_D5_mem_q10_srt_rmdup_peaks_peak_21622
chr3 167813184 167813455
P19_NANOG_CNCC_D5_mem_q10_srt_rmdup_peaks_peak_21632
chr3 167966537 167966828
P19_NANOG_CNCC_D5_mem_q10_srt_rmdup_peaks_peak_21634
chr3 168182460 168182796
P19_NANOG_CNCC_D5_mem_q10_srt_rmdup_peaks_peak_21636
chr3 168371108 168371419
P19_NANOG_CNCC_D5_mem_q10_srt_rmdup_peaks_peak_21645
chr3 168546758 168547157
P19_NANOG_CNCC_D5_mem_q10_srt_rmdup_peaks_peak_21650
chr3 169480804 169481075
P19_NANOG_CNCC_D5_mem_q10_srt_rmdup_peaks_peak_21666
chr3 169684031 169684540
P19_NANOG_CNCC_D5_mem_q10_srt_rmdup_peaks_peak_21668
chr3 170246163 170246514
P19_NANOG_CNCC_D5_mem_q10_srt_rmdup_peaks_peak_21675
chr3 170848645 170848974
P19_NANOG_CNCC_D5_mem_q10_srt_rmdup_peaks_peak_21681
chr3 171527815 171528142
P19_NANOG_CNCC_D5_mem_q10_srt_rmdup_peaks_peak_21692
chr3 171931829 171932136
P19_NANOG_CNCC_D5_mem_q10_srt_rmdup_peaks_peak_21698
chr3 171945993 171946266
P19_NANOG_CNCC_D5_mem_q10_srt_rmdup_peaks_peak_21700
chr3 172109756 172110091
P19_NANOG_CNCC_D5_mem_q10_srt_rmdup_peaks_peak_21702
chr3 172709727 172710002
P19_NANOG_CNCC_D5_mem_q10_srt_rmdup_peaks_peak_21707
chr3 172857184 172857591

P19_NANOG_CNCC_D5_mem_q10_srt_rmdup_peaks_peak_21708
chr3 173877403 173877868
P19_NANOG_CNCC_D5_mem_q10_srt_rmdup_peaks_peak_21725
chr3 174184173 174184674
P19_NANOG_CNCC_D5_mem_q10_srt_rmdup_peaks_peak_21729
chr3 174254212 174254644
P19_NANOG_CNCC_D5_mem_q10_srt_rmdup_peaks_peak_21730
chr3 175658748 175659436
P19_NANOG_CNCC_D5_mem_q10_srt_rmdup_peaks_peak_21737
chr3 176240827 176241170
P19_NANOG_CNCC_D5_mem_q10_srt_rmdup_peaks_peak_21745
chr3 176913008 176913321
P19_NANOG_CNCC_D5_mem_q10_srt_rmdup_peaks_peak_21749
chr3 177239189 177239461
P19_NANOG_CNCC_D5_mem_q10_srt_rmdup_peaks_peak_21755
chr3 177329080 177329395
P19_NANOG_CNCC_D5_mem_q10_srt_rmdup_peaks_peak_21758
chr3 177411579 177411954
P19_NANOG_CNCC_D5_mem_q10_srt_rmdup_peaks_peak_21759
chr3 178898966 178899409
P19_NANOG_CNCC_D5_mem_q10_srt_rmdup_peaks_peak_21766
chr3 178914529 178914829
P19_NANOG_CNCC_D5_mem_q10_srt_rmdup_peaks_peak_21767
chr3 179547073 179547533
P19_NANOG_CNCC_D5_mem_q10_srt_rmdup_peaks_peak_21774
chr3 179559889 179560198
P19_NANOG_CNCC_D5_mem_q10_srt_rmdup_peaks_peak_21775
chr3 179657344 179657675
P19_NANOG_CNCC_D5_mem_q10_srt_rmdup_peaks_peak_21778
chr3 180319833 180320105
P19_NANOG_CNCC_D5_mem_q10_srt_rmdup_peaks_peak_21785
chr3 180424745 180425087
P19_NANOG_CNCC_D5_mem_q10_srt_rmdup_peaks_peak_21787
chr3 180462026 180462318
P19_NANOG_CNCC_D5_mem_q10_srt_rmdup_peaks_peak_21789
chr3 180888989 180889329
P19_NANOG_CNCC_D5_mem_q10_srt_rmdup_peaks_peak_21795
chr3 181172409 181172689
P19_NANOG_CNCC_D5_mem_q10_srt_rmdup_peaks_peak_21797
chr3 181684080 181684545
P19_NANOG_CNCC_D5_mem_q10_srt_rmdup_peaks_peak_21818
chr3 182168713 182169166
P19_NANOG_CNCC_D5_mem_q10_srt_rmdup_peaks_peak_21829
chr3 182983842 182984186
P19_NANOG_CNCC_D5_mem_q10_srt_rmdup_peaks_peak_21839
chr3 182986257 182986568
P19_NANOG_CNCC_D5_mem_q10_srt_rmdup_peaks_peak_21840
chr3 183146774 183147214
P19_NANOG_CNCC_D5_mem_q10_srt_rmdup_peaks_peak_21842
chr3 183980315 183980613

P19_NANOG_CNCC_D5_mem_q10_srt_rmdup_peaks_peak_21851
chr3 184243586 184243868
P19_NANOG_CNCC_D5_mem_q10_srt_rmdup_peaks_peak_21856
chr3 184256078 184256635
P19_NANOG_CNCC_D5_mem_q10_srt_rmdup_peaks_peak_21857
chr3 184267653 184268049
P19_NANOG_CNCC_D5_mem_q10_srt_rmdup_peaks_peak_21858
chr3 184374657 184374965
P19_NANOG_CNCC_D5_mem_q10_srt_rmdup_peaks_peak_21866
chr3 184445210 184445577
P19_NANOG_CNCC_D5_mem_q10_srt_rmdup_peaks_peak_21870
chr3 184525923 184526232
P19_NANOG_CNCC_D5_mem_q10_srt_rmdup_peaks_peak_21873
chr3 185048529 185048816
P19_NANOG_CNCC_D5_mem_q10_srt_rmdup_peaks_peak_21877
chr3 185301639 185302081
P19_NANOG_CNCC_D5_mem_q10_srt_rmdup_peaks_peak_21881
chr3 185467739 185468097
P19_NANOG_CNCC_D5_mem_q10_srt_rmdup_peaks_peak_21886
chr3 185520585 185520874
P19_NANOG_CNCC_D5_mem_q10_srt_rmdup_peaks_peak_21891
chr3 186100232 186100613
P19_NANOG_CNCC_D5_mem_q10_srt_rmdup_peaks_peak_21902
chr3 186898790 186899155
P19_NANOG_CNCC_D5_mem_q10_srt_rmdup_peaks_peak_21918
chr3 187183984 187184598
P19_NANOG_CNCC_D5_mem_q10_srt_rmdup_peaks_peak_21923
chr3 187437491 187438023
P19_NANOG_CNCC_D5_mem_q10_srt_rmdup_peaks_peak_21929
chr3 187721552 187721962
P19_NANOG_CNCC_D5_mem_q10_srt_rmdup_peaks_peak_21936
chr3 187903565 187904080
P19_NANOG_CNCC_D5_mem_q10_srt_rmdup_peaks_peak_21940
chr3 189166811 189167268
P19_NANOG_CNCC_D5_mem_q10_srt_rmdup_peaks_peak_21951
chr3 189976368 189976725
P19_NANOG_CNCC_D5_mem_q10_srt_rmdup_peaks_peak_21963
chr3 190610351 190610708
P19_NANOG_CNCC_D5_mem_q10_srt_rmdup_peaks_peak_21968
chr3 190766626 190766921
P19_NANOG_CNCC_D5_mem_q10_srt_rmdup_peaks_peak_21972
chr3 191019788 191020230
P19_NANOG_CNCC_D5_mem_q10_srt_rmdup_peaks_peak_21976
chr3 191076802 191077107
P19_NANOG_CNCC_D5_mem_q10_srt_rmdup_peaks_peak_21978
chr3 191464596 191464935
P19_NANOG_CNCC_D5_mem_q10_srt_rmdup_peaks_peak_21980
chr3 192746008 192746551
P19_NANOG_CNCC_D5_mem_q10_srt_rmdup_peaks_peak_21993
chr3 193106331 193106658

P19_NANOG_CNCC_D5_mem_q10_srt_rmdup_peaks_peak_21996
chr3 193432279 193432689
P19_NANOG_CNCC_D5_mem_q10_srt_rmdup_peaks_peak_22003
chr3 194060052 194061149
P19_NANOG_CNCC_D5_mem_q10_srt_rmdup_peaks_peak_22029
chr3 194654542 194654924
P19_NANOG_CNCC_D5_mem_q10_srt_rmdup_peaks_peak_22038
chr3 194944019 194944594
P19_NANOG_CNCC_D5_mem_q10_srt_rmdup_peaks_peak_22043
chr3 194980612 194981075
P19_NANOG_CNCC_D5_mem_q10_srt_rmdup_peaks_peak_22045
chr3 195717184 195717468
P19_NANOG_CNCC_D5_mem_q10_srt_rmdup_peaks_peak_22057
chr3 196346319 196346972
P19_NANOG_CNCC_D5_mem_q10_srt_rmdup_peaks_peak_22065
chr3 196756470 196756903
P19_NANOG_CNCC_D5_mem_q10_srt_rmdup_peaks_peak_22069
chr4 171402 171724
P19_NANOG_CNCC_D5_mem_q10_srt_rmdup_peaks_peak_22092
chr4 1340742 1341038
P19_NANOG_CNCC_D5_mem_q10_srt_rmdup_peaks_peak_22100
chr4 2463239 2463561
P19_NANOG_CNCC_D5_mem_q10_srt_rmdup_peaks_peak_22115
chr4 4036738 4037009
P19_NANOG_CNCC_D5_mem_q10_srt_rmdup_peaks_peak_22139
chr4 4432075 4432495
P19_NANOG_CNCC_D5_mem_q10_srt_rmdup_peaks_peak_22146
chr4 4459661 4459944
P19_NANOG_CNCC_D5_mem_q10_srt_rmdup_peaks_peak_22147
chr4 5203683 5204131
P19_NANOG_CNCC_D5_mem_q10_srt_rmdup_peaks_peak_22174
chr4 5367406 5367679
P19_NANOG_CNCC_D5_mem_q10_srt_rmdup_peaks_peak_22175
chr4 6784788 6785136
P19_NANOG_CNCC_D5_mem_q10_srt_rmdup_peaks_peak_22198
chr4 7432165 7432687
P19_NANOG_CNCC_D5_mem_q10_srt_rmdup_peaks_peak_22210
chr4 7635150 7635585
P19_NANOG_CNCC_D5_mem_q10_srt_rmdup_peaks_peak_22213
chr4 8566439 8566710
P19_NANOG_CNCC_D5_mem_q10_srt_rmdup_peaks_peak_22227
chr4 8835998 8836366
P19_NANOG_CNCC_D5_mem_q10_srt_rmdup_peaks_peak_22230
chr4 8869315 8869795
P19_NANOG_CNCC_D5_mem_q10_srt_rmdup_peaks_peak_22235
chr4 10537540 10537992
P19_NANOG_CNCC_D5_mem_q10_srt_rmdup_peaks_peak_22251
chr4 11430538 11430869
P19_NANOG_CNCC_D5_mem_q10_srt_rmdup_peaks_peak_22258
chr4 12421221 12421492

P19_NANOG_CNCC_D5_mem_q10_srt_rmdup_peaks_peak_22266
chr4 13091414 13091685
P19_NANOG_CNCC_D5_mem_q10_srt_rmdup_peaks_peak_22271
chr4 14748145 14748416
P19_NANOG_CNCC_D5_mem_q10_srt_rmdup_peaks_peak_22294
chr4 15003725 15004005
P19_NANOG_CNCC_D5_mem_q10_srt_rmdup_peaks_peak_22296
chr4 15011903 15012180
P19_NANOG_CNCC_D5_mem_q10_srt_rmdup_peaks_peak_22297
chr4 15503538 15503982
P19_NANOG_CNCC_D5_mem_q10_srt_rmdup_peaks_peak_22306
chr4 15907733 15908159
P19_NANOG_CNCC_D5_mem_q10_srt_rmdup_peaks_peak_22314
chr4 16228051 16228328
P19_NANOG_CNCC_D5_mem_q10_srt_rmdup_peaks_peak_22320
chr4 16791532 16791881
P19_NANOG_CNCC_D5_mem_q10_srt_rmdup_peaks_peak_22329
chr4 16870122 16870541
P19_NANOG_CNCC_D5_mem_q10_srt_rmdup_peaks_peak_22332
chr4 17008226 17008522
P19_NANOG_CNCC_D5_mem_q10_srt_rmdup_peaks_peak_22336
chr4 17634056 17634793
P19_NANOG_CNCC_D5_mem_q10_srt_rmdup_peaks_peak_22341
chr4 19012458 19012842
P19_NANOG_CNCC_D5_mem_q10_srt_rmdup_peaks_peak_22346
chr4 20215609 20215967
P19_NANOG_CNCC_D5_mem_q10_srt_rmdup_peaks_peak_22352
chr4 20334981 20335252
P19_NANOG_CNCC_D5_mem_q10_srt_rmdup_peaks_peak_22355
chr4 20353246 20353580
P19_NANOG_CNCC_D5_mem_q10_srt_rmdup_peaks_peak_22356
chr4 21710181 21710582
P19_NANOG_CNCC_D5_mem_q10_srt_rmdup_peaks_peak_22363
chr4 21767392 21767779
P19_NANOG_CNCC_D5_mem_q10_srt_rmdup_peaks_peak_22364
chr4 22188879 22189166
P19_NANOG_CNCC_D5_mem_q10_srt_rmdup_peaks_peak_22365
chr4 22500569 22500891
P19_NANOG_CNCC_D5_mem_q10_srt_rmdup_peaks_peak_22367
chr4 22501701 22502077
P19_NANOG_CNCC_D5_mem_q10_srt_rmdup_peaks_peak_22368
chr4 22517545 22517931
P19_NANOG_CNCC_D5_mem_q10_srt_rmdup_peaks_peak_22369
chr4 23086169 23086451
P19_NANOG_CNCC_D5_mem_q10_srt_rmdup_peaks_peak_22375
chr4 24031541 24031914
P19_NANOG_CNCC_D5_mem_q10_srt_rmdup_peaks_peak_22385
chr4 24512316 24512808
P19_NANOG_CNCC_D5_mem_q10_srt_rmdup_peaks_peak_22392
chr4 24560642 24560961

P19_NANOG_CNCC_D5_mem_q10_srt_rmdup_peaks_peak_22394
chr4 24705839 24706169
P19_NANOG_CNCC_D5_mem_q10_srt_rmdup_peaks_peak_22399
chr4 25066046 25066419
P19_NANOG_CNCC_D5_mem_q10_srt_rmdup_peaks_peak_22406
chr4 25591811 25592094
P19_NANOG_CNCC_D5_mem_q10_srt_rmdup_peaks_peak_22416
chr4 26740623 26740910
P19_NANOG_CNCC_D5_mem_q10_srt_rmdup_peaks_peak_22439
chr4 27086455 27086789
P19_NANOG_CNCC_D5_mem_q10_srt_rmdup_peaks_peak_22445
chr4 27089308 27089601
P19_NANOG_CNCC_D5_mem_q10_srt_rmdup_peaks_peak_22446
chr4 30057676 30058026
P19_NANOG_CNCC_D5_mem_q10_srt_rmdup_peaks_peak_22457
chr4 30795299 30795602
P19_NANOG_CNCC_D5_mem_q10_srt_rmdup_peaks_peak_22464
chr4 31359146 31359517
P19_NANOG_CNCC_D5_mem_q10_srt_rmdup_peaks_peak_22475
chr4 31790493 31790789
P19_NANOG_CNCC_D5_mem_q10_srt_rmdup_peaks_peak_22476
chr4 32633472 32633743
P19_NANOG_CNCC_D5_mem_q10_srt_rmdup_peaks_peak_22480
chr4 33178302 33178587
P19_NANOG_CNCC_D5_mem_q10_srt_rmdup_peaks_peak_22483
chr4 33382752 33383062
P19_NANOG_CNCC_D5_mem_q10_srt_rmdup_peaks_peak_22485
chr4 34167222 34167684
P19_NANOG_CNCC_D5_mem_q10_srt_rmdup_peaks_peak_22486
chr4 35361488 35361792
P19_NANOG_CNCC_D5_mem_q10_srt_rmdup_peaks_peak_22490
chr4 37671455 37671807
P19_NANOG_CNCC_D5_mem_q10_srt_rmdup_peaks_peak_22507
chr4 38003568 38004059
P19_NANOG_CNCC_D5_mem_q10_srt_rmdup_peaks_peak_22512
chr4 40398140 40398557
P19_NANOG_CNCC_D5_mem_q10_srt_rmdup_peaks_peak_22534
chr4 40527740 40528128
P19_NANOG_CNCC_D5_mem_q10_srt_rmdup_peaks_peak_22537
chr4 40623635 40623927
P19_NANOG_CNCC_D5_mem_q10_srt_rmdup_peaks_peak_22538
chr4 40630358 40630688
P19_NANOG_CNCC_D5_mem_q10_srt_rmdup_peaks_peak_22539
chr4 40683536 40683969
P19_NANOG_CNCC_D5_mem_q10_srt_rmdup_peaks_peak_22542
chr4 41369066 41369338
P19_NANOG_CNCC_D5_mem_q10_srt_rmdup_peaks_peak_22557
chr4 41750002 41750334
P19_NANOG_CNCC_D5_mem_q10_srt_rmdup_peaks_peak_22568
chr4 42398300 42398672

P19_NANOG_CNCC_D5_mem_q10_srt_rmdup_peaks_peak_22582
chr4 42489064 42489432
P19_NANOG_CNCC_D5_mem_q10_srt_rmdup_peaks_peak_22586
chr4 44103619 44104132
P19_NANOG_CNCC_D5_mem_q10_srt_rmdup_peaks_peak_22598
chr4 44258560 44258960
P19_NANOG_CNCC_D5_mem_q10_srt_rmdup_peaks_peak_22599
chr4 44793901 44794192
P19_NANOG_CNCC_D5_mem_q10_srt_rmdup_peaks_peak_22603
chr4 45250018 45250423
P19_NANOG_CNCC_D5_mem_q10_srt_rmdup_peaks_peak_22607
chr4 46312272 46312593
P19_NANOG_CNCC_D5_mem_q10_srt_rmdup_peaks_peak_22610
chr4 47826385 47826731
P19_NANOG_CNCC_D5_mem_q10_srt_rmdup_peaks_peak_22615
chr4 48030689 48031147
P19_NANOG_CNCC_D5_mem_q10_srt_rmdup_peaks_peak_22619
chr4 48372346 48372635
P19_NANOG_CNCC_D5_mem_q10_srt_rmdup_peaks_peak_22625
chr4 53355424 53355848
P19_NANOG_CNCC_D5_mem_q10_srt_rmdup_peaks_peak_22642
chr4 53782752 53783143
P19_NANOG_CNCC_D5_mem_q10_srt_rmdup_peaks_peak_22653
chr4 54363533 54363978
P19_NANOG_CNCC_D5_mem_q10_srt_rmdup_peaks_peak_22656
chr4 56305116 56305644
P19_NANOG_CNCC_D5_mem_q10_srt_rmdup_peaks_peak_22687
chr4 56501128 56501910
P19_NANOG_CNCC_D5_mem_q10_srt_rmdup_peaks_peak_22690
chr4 56915782 56916072
P19_NANOG_CNCC_D5_mem_q10_srt_rmdup_peaks_peak_22693
chr4 56952175 56952510
P19_NANOG_CNCC_D5_mem_q10_srt_rmdup_peaks_peak_22694
chr4 57371646 57372123
P19_NANOG_CNCC_D5_mem_q10_srt_rmdup_peaks_peak_22702
chr4 58577838 58578351
P19_NANOG_CNCC_D5_mem_q10_srt_rmdup_peaks_peak_22722
chr4 59058452 59058776
P19_NANOG_CNCC_D5_mem_q10_srt_rmdup_peaks_peak_22723
chr4 60788250 60788596
P19_NANOG_CNCC_D5_mem_q10_srt_rmdup_peaks_peak_22728
chr4 62104989 62105354
P19_NANOG_CNCC_D5_mem_q10_srt_rmdup_peaks_peak_22734
chr4 62701369 62701780
P19_NANOG_CNCC_D5_mem_q10_srt_rmdup_peaks_peak_22742
chr4 63243469 63243748
P19_NANOG_CNCC_D5_mem_q10_srt_rmdup_peaks_peak_22748
chr4 64693301 64693749
P19_NANOG_CNCC_D5_mem_q10_srt_rmdup_peaks_peak_22753
chr4 65308105 65308582

P19_NANOG_CNCC_D5_mem_q10_srt_rmdup_peaks_peak_22755
chr4 65386196 65386534
P19_NANOG_CNCC_D5_mem_q10_srt_rmdup_peaks_peak_22756
chr4 66400810 66401117
P19_NANOG_CNCC_D5_mem_q10_srt_rmdup_peaks_peak_22760
chr4 69419017 69419288
P19_NANOG_CNCC_D5_mem_q10_srt_rmdup_peaks_peak_22775
chr4 70314883 70315196
P19_NANOG_CNCC_D5_mem_q10_srt_rmdup_peaks_peak_22779
chr4 75027349 75027711
P19_NANOG_CNCC_D5_mem_q10_srt_rmdup_peaks_peak_22811
chr4 76254756 76255135
P19_NANOG_CNCC_D5_mem_q10_srt_rmdup_peaks_peak_22819
chr4 76434331 76434617
P19_NANOG_CNCC_D5_mem_q10_srt_rmdup_peaks_peak_22820
chr4 76633361 76633712
P19_NANOG_CNCC_D5_mem_q10_srt_rmdup_peaks_peak_22824
chr4 77135144 77135535
P19_NANOG_CNCC_D5_mem_q10_srt_rmdup_peaks_peak_22830
chr4 77467502 77467859
P19_NANOG_CNCC_D5_mem_q10_srt_rmdup_peaks_peak_22836
chr4 77609376 77609874
P19_NANOG_CNCC_D5_mem_q10_srt_rmdup_peaks_peak_22842
chr4 77819348 77819745
P19_NANOG_CNCC_D5_mem_q10_srt_rmdup_peaks_peak_22847
chr4 77923110 77923531
P19_NANOG_CNCC_D5_mem_q10_srt_rmdup_peaks_peak_22849
chr4 78230035 78230306
P19_NANOG_CNCC_D5_mem_q10_srt_rmdup_peaks_peak_22855
chr4 79282853 79283164
P19_NANOG_CNCC_D5_mem_q10_srt_rmdup_peaks_peak_22866
chr4 79583144 79583630
P19_NANOG_CNCC_D5_mem_q10_srt_rmdup_peaks_peak_22870
chr4 79585324 79585697
P19_NANOG_CNCC_D5_mem_q10_srt_rmdup_peaks_peak_22871
chr4 80162928 80163286
P19_NANOG_CNCC_D5_mem_q10_srt_rmdup_peaks_peak_22879
chr4 80880697 80881270
P19_NANOG_CNCC_D5_mem_q10_srt_rmdup_peaks_peak_22883
chr4 80885345 80886278
P19_NANOG_CNCC_D5_mem_q10_srt_rmdup_peaks_peak_22884
chr4 81341301 81341583
P19_NANOG_CNCC_D5_mem_q10_srt_rmdup_peaks_peak_22892
chr4 82374116 82374624
P19_NANOG_CNCC_D5_mem_q10_srt_rmdup_peaks_peak_22901
chr4 82393420 82393691
P19_NANOG_CNCC_D5_mem_q10_srt_rmdup_peaks_peak_22902
chr4 82603646 82604100
P19_NANOG_CNCC_D5_mem_q10_srt_rmdup_peaks_peak_22908
chr4 83822030 83822364

P19_NANOG_CNCC_D5_mem_q10_srt_rmdup_peaks_peak_22925
chr4 84131207 84131635
P19_NANOG_CNCC_D5_mem_q10_srt_rmdup_peaks_peak_22928
chr4 84972673 84973110
P19_NANOG_CNCC_D5_mem_q10_srt_rmdup_peaks_peak_22941
chr4 85086412 85086834
P19_NANOG_CNCC_D5_mem_q10_srt_rmdup_peaks_peak_22944
chr4 85246506 85246877
P19_NANOG_CNCC_D5_mem_q10_srt_rmdup_peaks_peak_22947
chr4 85334860 85335154
P19_NANOG_CNCC_D5_mem_q10_srt_rmdup_peaks_peak_22952
chr4 86046825 86047298
P19_NANOG_CNCC_D5_mem_q10_srt_rmdup_peaks_peak_22968
chr4 86049745 86050038
P19_NANOG_CNCC_D5_mem_q10_srt_rmdup_peaks_peak_22970
chr4 86664422 86664693
P19_NANOG_CNCC_D5_mem_q10_srt_rmdup_peaks_peak_22972
chr4 88329393 88329779
P19_NANOG_CNCC_D5_mem_q10_srt_rmdup_peaks_peak_22997
chr4 88894272 88894698
P19_NANOG_CNCC_D5_mem_q10_srt_rmdup_peaks_peak_23009
chr4 89360489 89360810
P19_NANOG_CNCC_D5_mem_q10_srt_rmdup_peaks_peak_23013
chr4 90423797 90424101
P19_NANOG_CNCC_D5_mem_q10_srt_rmdup_peaks_peak_23025
chr4 90758516 90758873
P19_NANOG_CNCC_D5_mem_q10_srt_rmdup_peaks_peak_23029
chr4 91985631 91986059
P19_NANOG_CNCC_D5_mem_q10_srt_rmdup_peaks_peak_23034
chr4 92035538 92035836
P19_NANOG_CNCC_D5_mem_q10_srt_rmdup_peaks_peak_23038
chr4 92544442 92545161
P19_NANOG_CNCC_D5_mem_q10_srt_rmdup_peaks_peak_23043
chr4 93125273 93125590
P19_NANOG_CNCC_D5_mem_q10_srt_rmdup_peaks_peak_23046
chr4 93195278 93195549
P19_NANOG_CNCC_D5_mem_q10_srt_rmdup_peaks_peak_23048
chr4 93536336 93536631
P19_NANOG_CNCC_D5_mem_q10_srt_rmdup_peaks_peak_23056
chr4 94104481 94104907
P19_NANOG_CNCC_D5_mem_q10_srt_rmdup_peaks_peak_23060
chr4 94714479 94714750
P19_NANOG_CNCC_D5_mem_q10_srt_rmdup_peaks_peak_23066
chr4 95461740 95462032
P19_NANOG_CNCC_D5_mem_q10_srt_rmdup_peaks_peak_23078
chr4 95498297 95498663
P19_NANOG_CNCC_D5_mem_q10_srt_rmdup_peaks_peak_23079
chr4 95732396 95732776
P19_NANOG_CNCC_D5_mem_q10_srt_rmdup_peaks_peak_23081
chr4 96319720 96320030

P19_NANOG_CNCC_D5_mem_q10_srt_rmdup_peaks_peak_23088
chr4 96681963 96682365
P19_NANOG_CNCC_D5_mem_q10_srt_rmdup_peaks_peak_23090
chr4 99404455 99404736
P19_NANOG_CNCC_D5_mem_q10_srt_rmdup_peaks_peak_23110
chr4 99975780 99976051
P19_NANOG_CNCC_D5_mem_q10_srt_rmdup_peaks_peak_23122
chr4 101111708 101112010
P19_NANOG_CNCC_D5_mem_q10_srt_rmdup_peaks_peak_23137
chr4 101261235 101261614
P19_NANOG_CNCC_D5_mem_q10_srt_rmdup_peaks_peak_23139
chr4 101726710 101727017
P19_NANOG_CNCC_D5_mem_q10_srt_rmdup_peaks_peak_23140
chr4 102023926 102024197
P19_NANOG_CNCC_D5_mem_q10_srt_rmdup_peaks_peak_23148
chr4 102076526 102076849
P19_NANOG_CNCC_D5_mem_q10_srt_rmdup_peaks_peak_23150
chr4 102340742 102341271
P19_NANOG_CNCC_D5_mem_q10_srt_rmdup_peaks_peak_23155
chr4 102871458 102871731
P19_NANOG_CNCC_D5_mem_q10_srt_rmdup_peaks_peak_23160
chr4 103243936 103244217
P19_NANOG_CNCC_D5_mem_q10_srt_rmdup_peaks_peak_23165
chr4 103681949 103682308
P19_NANOG_CNCC_D5_mem_q10_srt_rmdup_peaks_peak_23167
chr4 103751242 103751524
P19_NANOG_CNCC_D5_mem_q10_srt_rmdup_peaks_peak_23170
chr4 105267795 105268114
P19_NANOG_CNCC_D5_mem_q10_srt_rmdup_peaks_peak_23181
chr4 107684053 107684575
P19_NANOG_CNCC_D5_mem_q10_srt_rmdup_peaks_peak_23198
chr4 108336957 108337285
P19_NANOG_CNCC_D5_mem_q10_srt_rmdup_peaks_peak_23202
chr4 109068024 109068322
P19_NANOG_CNCC_D5_mem_q10_srt_rmdup_peaks_peak_23211
chr4 109636021 109636411
P19_NANOG_CNCC_D5_mem_q10_srt_rmdup_peaks_peak_23217
chr4 109861662 109862132
P19_NANOG_CNCC_D5_mem_q10_srt_rmdup_peaks_peak_23220
chr4 110196142 110196459
P19_NANOG_CNCC_D5_mem_q10_srt_rmdup_peaks_peak_23223
chr4 110624343 110624802
P19_NANOG_CNCC_D5_mem_q10_srt_rmdup_peaks_peak_23227
chr4 111025862 111026171
P19_NANOG_CNCC_D5_mem_q10_srt_rmdup_peaks_peak_23232
chr4 112531793 112532372
P19_NANOG_CNCC_D5_mem_q10_srt_rmdup_peaks_peak_23259
chr4 112691415 112691782
P19_NANOG_CNCC_D5_mem_q10_srt_rmdup_peaks_peak_23261
chr4 113565865 113566287

P19_NANOG_CNCC_D5_mem_q10_srt_rmdup_peaks_peak_23285
chr4 113576175 113576457
P19_NANOG_CNCC_D5_mem_q10_srt_rmdup_peaks_peak_23289
chr4 114306575 114307123
P19_NANOG_CNCC_D5_mem_q10_srt_rmdup_peaks_peak_23308
chr4 114373320 114373669
P19_NANOG_CNCC_D5_mem_q10_srt_rmdup_peaks_peak_23309
chr4 114567026 114567321
P19_NANOG_CNCC_D5_mem_q10_srt_rmdup_peaks_peak_23313
chr4 114799889 114800293
P19_NANOG_CNCC_D5_mem_q10_srt_rmdup_peaks_peak_23316
chr4 114805818 114806151
P19_NANOG_CNCC_D5_mem_q10_srt_rmdup_peaks_peak_23317
chr4 114917843 114918250
P19_NANOG_CNCC_D5_mem_q10_srt_rmdup_peaks_peak_23318
chr4 115713604 115713907
P19_NANOG_CNCC_D5_mem_q10_srt_rmdup_peaks_peak_23326
chr4 116155927 116156316
P19_NANOG_CNCC_D5_mem_q10_srt_rmdup_peaks_peak_23330
chr4 118742917 118743381
P19_NANOG_CNCC_D5_mem_q10_srt_rmdup_peaks_peak_23337
chr4 119426656 119426927
P19_NANOG_CNCC_D5_mem_q10_srt_rmdup_peaks_peak_23341
chr4 119644462 119644889
P19_NANOG_CNCC_D5_mem_q10_srt_rmdup_peaks_peak_23344
chr4 119945229 119945736
P19_NANOG_CNCC_D5_mem_q10_srt_rmdup_peaks_peak_23350
chr4 119957147 119957463
P19_NANOG_CNCC_D5_mem_q10_srt_rmdup_peaks_peak_23351
chr4 120594152 120594461
P19_NANOG_CNCC_D5_mem_q10_srt_rmdup_peaks_peak_23362
chr4 122662811 122663221
P19_NANOG_CNCC_D5_mem_q10_srt_rmdup_peaks_peak_23373
chr4 123684061 123684572
P19_NANOG_CNCC_D5_mem_q10_srt_rmdup_peaks_peak_23383
chr4 124468453 124468869
P19_NANOG_CNCC_D5_mem_q10_srt_rmdup_peaks_peak_23397
chr4 124897463 124897873
P19_NANOG_CNCC_D5_mem_q10_srt_rmdup_peaks_peak_23404
chr4 125728662 125728942
P19_NANOG_CNCC_D5_mem_q10_srt_rmdup_peaks_peak_23411
chr4 127504603 127504936
P19_NANOG_CNCC_D5_mem_q10_srt_rmdup_peaks_peak_23423
chr4 129283930 129284399
P19_NANOG_CNCC_D5_mem_q10_srt_rmdup_peaks_peak_23437
chr4 129569745 129570059
P19_NANOG_CNCC_D5_mem_q10_srt_rmdup_peaks_peak_23442
chr4 129575175 129575460
P19_NANOG_CNCC_D5_mem_q10_srt_rmdup_peaks_peak_23443
chr4 130085350 130085710

P19_NANOG_CNCC_D5_mem_q10_srt_rmdup_peaks_peak_23454
chr4 131520741 131521059
P19_NANOG_CNCC_D5_mem_q10_srt_rmdup_peaks_peak_23464
chr4 134397249 134397585
P19_NANOG_CNCC_D5_mem_q10_srt_rmdup_peaks_peak_23478
chr4 135439934 135440346
P19_NANOG_CNCC_D5_mem_q10_srt_rmdup_peaks_peak_23483
chr4 136107998 136108359
P19_NANOG_CNCC_D5_mem_q10_srt_rmdup_peaks_peak_23489
chr4 136482427 136482719
P19_NANOG_CNCC_D5_mem_q10_srt_rmdup_peaks_peak_23491
chr4 136760128 136760455
P19_NANOG_CNCC_D5_mem_q10_srt_rmdup_peaks_peak_23494
chr4 137724569 137725001
P19_NANOG_CNCC_D5_mem_q10_srt_rmdup_peaks_peak_23503
chr4 139138754 139139266
P19_NANOG_CNCC_D5_mem_q10_srt_rmdup_peaks_peak_23513
chr4 139813000 139813299
P19_NANOG_CNCC_D5_mem_q10_srt_rmdup_peaks_peak_23522
chr4 139951923 139952194
P19_NANOG_CNCC_D5_mem_q10_srt_rmdup_peaks_peak_23526
chr4 140052456 140052876
P19_NANOG_CNCC_D5_mem_q10_srt_rmdup_peaks_peak_23528
chr4 140464121 140464413
P19_NANOG_CNCC_D5_mem_q10_srt_rmdup_peaks_peak_23540
chr4 141538868 141539258
P19_NANOG_CNCC_D5_mem_q10_srt_rmdup_peaks_peak_23566
chr4 141998816 141999229
P19_NANOG_CNCC_D5_mem_q10_srt_rmdup_peaks_peak_23573
chr4 142678583 142678937
P19_NANOG_CNCC_D5_mem_q10_srt_rmdup_peaks_peak_23577
chr4 143144593 143144923
P19_NANOG_CNCC_D5_mem_q10_srt_rmdup_peaks_peak_23580
chr4 143471063 143471466
P19_NANOG_CNCC_D5_mem_q10_srt_rmdup_peaks_peak_23584
chr4 145568077 145568348
P19_NANOG_CNCC_D5_mem_q10_srt_rmdup_peaks_peak_23591
chr4 146608147 146608496
P19_NANOG_CNCC_D5_mem_q10_srt_rmdup_peaks_peak_23599
chr4 146713306 146713706
P19_NANOG_CNCC_D5_mem_q10_srt_rmdup_peaks_peak_23600
chr4 146989402 146989704
P19_NANOG_CNCC_D5_mem_q10_srt_rmdup_peaks_peak_23602
chr4 147302584 147302937
P19_NANOG_CNCC_D5_mem_q10_srt_rmdup_peaks_peak_23603
chr4 147765841 147766133
P19_NANOG_CNCC_D5_mem_q10_srt_rmdup_peaks_peak_23610
chr4 147923761 147924151
P19_NANOG_CNCC_D5_mem_q10_srt_rmdup_peaks_peak_23611
chr4 148096901 148097230

P19_NANOG_CNCC_D5_mem_q10_srt_rmdup_peaks_peak_23614
chr4 148119603 148119930
P19_NANOG_CNCC_D5_mem_q10_srt_rmdup_peaks_peak_23615
chr4 148261918 148262189
P19_NANOG_CNCC_D5_mem_q10_srt_rmdup_peaks_peak_23616
chr4 148756228 148756698
P19_NANOG_CNCC_D5_mem_q10_srt_rmdup_peaks_peak_23625
chr4 150037615 150038023
P19_NANOG_CNCC_D5_mem_q10_srt_rmdup_peaks_peak_23636
chr4 150471187 150471493
P19_NANOG_CNCC_D5_mem_q10_srt_rmdup_peaks_peak_23638
chr4 151503466 151503779
P19_NANOG_CNCC_D5_mem_q10_srt_rmdup_peaks_peak_23643
chr4 151885566 151885996
P19_NANOG_CNCC_D5_mem_q10_srt_rmdup_peaks_peak_23648
chr4 152462757 152463034
P19_NANOG_CNCC_D5_mem_q10_srt_rmdup_peaks_peak_23659
chr4 152475085 152475356
P19_NANOG_CNCC_D5_mem_q10_srt_rmdup_peaks_peak_23660
chr4 152666708 152666979
P19_NANOG_CNCC_D5_mem_q10_srt_rmdup_peaks_peak_23662
chr4 152752694 152753067
P19_NANOG_CNCC_D5_mem_q10_srt_rmdup_peaks_peak_23665
chr4 152931824 152932220
P19_NANOG_CNCC_D5_mem_q10_srt_rmdup_peaks_peak_23669
chr4 153599931 153600397
P19_NANOG_CNCC_D5_mem_q10_srt_rmdup_peaks_peak_23675
chr4 153649943 153650244
P19_NANOG_CNCC_D5_mem_q10_srt_rmdup_peaks_peak_23680
chr4 153905029 153905391
P19_NANOG_CNCC_D5_mem_q10_srt_rmdup_peaks_peak_23683
chr4 154101758 154102417
P19_NANOG_CNCC_D5_mem_q10_srt_rmdup_peaks_peak_23691
chr4 154930282 154930662
P19_NANOG_CNCC_D5_mem_q10_srt_rmdup_peaks_peak_23706
chr4 155413024 155413393
P19_NANOG_CNCC_D5_mem_q10_srt_rmdup_peaks_peak_23711
chr4 155789225 155789786
P19_NANOG_CNCC_D5_mem_q10_srt_rmdup_peaks_peak_23718
chr4 156941076 156941421
P19_NANOG_CNCC_D5_mem_q10_srt_rmdup_peaks_peak_23723
chr4 158941451 158941887
P19_NANOG_CNCC_D5_mem_q10_srt_rmdup_peaks_peak_23734
chr4 159823811 159824205
P19_NANOG_CNCC_D5_mem_q10_srt_rmdup_peaks_peak_23742
chr4 160079317 160079694
P19_NANOG_CNCC_D5_mem_q10_srt_rmdup_peaks_peak_23748
chr4 160124029 160124359
P19_NANOG_CNCC_D5_mem_q10_srt_rmdup_peaks_peak_23749
chr4 163998926 163999223

P19_NANOG_CNCC_D5_mem_q10_srt_rmdup_peaks_peak_23771
chr4 165385460 165385995
P19_NANOG_CNCC_D5_mem_q10_srt_rmdup_peaks_peak_23778
chr4 166531929 166532350
P19_NANOG_CNCC_D5_mem_q10_srt_rmdup_peaks_peak_23792
chr4 167502612 167502971
P19_NANOG_CNCC_D5_mem_q10_srt_rmdup_peaks_peak_23796
chr4 168747634 168748010
P19_NANOG_CNCC_D5_mem_q10_srt_rmdup_peaks_peak_23797
chr4 168938151 168938422
P19_NANOG_CNCC_D5_mem_q10_srt_rmdup_peaks_peak_23801
chr4 169197786 169198246
P19_NANOG_CNCC_D5_mem_q10_srt_rmdup_peaks_peak_23803
chr4 170188170 170188679
P19_NANOG_CNCC_D5_mem_q10_srt_rmdup_peaks_peak_23822
chr4 170820481 170820765
P19_NANOG_CNCC_D5_mem_q10_srt_rmdup_peaks_peak_23828
chr4 170856183 170856462
P19_NANOG_CNCC_D5_mem_q10_srt_rmdup_peaks_peak_23829
chr4 172914356 172914685
P19_NANOG_CNCC_D5_mem_q10_srt_rmdup_peaks_peak_23839
chr4 172994798 172995188
P19_NANOG_CNCC_D5_mem_q10_srt_rmdup_peaks_peak_23841
chr4 173074813 173075263
P19_NANOG_CNCC_D5_mem_q10_srt_rmdup_peaks_peak_23842
chr4 173789560 173789831
P19_NANOG_CNCC_D5_mem_q10_srt_rmdup_peaks_peak_23848
chr4 174133054 174133355
P19_NANOG_CNCC_D5_mem_q10_srt_rmdup_peaks_peak_23853
chr4 174290367 174290695
P19_NANOG_CNCC_D5_mem_q10_srt_rmdup_peaks_peak_23857
chr4 174292850 174293139
P19_NANOG_CNCC_D5_mem_q10_srt_rmdup_peaks_peak_23859
chr4 174449747 174450235
P19_NANOG_CNCC_D5_mem_q10_srt_rmdup_peaks_peak_23871
chr4 175453019 175453380
P19_NANOG_CNCC_D5_mem_q10_srt_rmdup_peaks_peak_23889
chr4 175528020 175528291
P19_NANOG_CNCC_D5_mem_q10_srt_rmdup_peaks_peak_23891
chr4 175976139 175976448
P19_NANOG_CNCC_D5_mem_q10_srt_rmdup_peaks_peak_23894
chr4 176136385 176136783
P19_NANOG_CNCC_D5_mem_q10_srt_rmdup_peaks_peak_23895
chr4 179302146 179302607
P19_NANOG_CNCC_D5_mem_q10_srt_rmdup_peaks_peak_23910
chr4 179867222 179867496
P19_NANOG_CNCC_D5_mem_q10_srt_rmdup_peaks_peak_23915
chr4 180087654 180088058
P19_NANOG_CNCC_D5_mem_q10_srt_rmdup_peaks_peak_23916
chr4 180386589 180386866

P19_NANOG_CNCC_D5_mem_q10_srt_rmdup_peaks_peak_23920
chr4 181724935 181725245
P19_NANOG_CNCC_D5_mem_q10_srt_rmdup_peaks_peak_23928
chr4 182167728 182167999
P19_NANOG_CNCC_D5_mem_q10_srt_rmdup_peaks_peak_23935
chr4 182723141 182723498
P19_NANOG_CNCC_D5_mem_q10_srt_rmdup_peaks_peak_23945
chr4 183260378 183260948
P19_NANOG_CNCC_D5_mem_q10_srt_rmdup_peaks_peak_23954
chr4 183615573 183616039
P19_NANOG_CNCC_D5_mem_q10_srt_rmdup_peaks_peak_23966
chr4 184029234 184029560
P19_NANOG_CNCC_D5_mem_q10_srt_rmdup_peaks_peak_23972
chr4 185297470 185297839
P19_NANOG_CNCC_D5_mem_q10_srt_rmdup_peaks_peak_23996
chr4 185306324 185306595
P19_NANOG_CNCC_D5_mem_q10_srt_rmdup_peaks_peak_23997
chr4 185450447 185450804
P19_NANOG_CNCC_D5_mem_q10_srt_rmdup_peaks_peak_24002
chr4 185976007 185976561
P19_NANOG_CNCC_D5_mem_q10_srt_rmdup_peaks_peak_24011
chr4 186001868 186002239
P19_NANOG_CNCC_D5_mem_q10_srt_rmdup_peaks_peak_24012
chr4 186029239 186029542
P19_NANOG_CNCC_D5_mem_q10_srt_rmdup_peaks_peak_24013
chr4 186254221 186254570
P19_NANOG_CNCC_D5_mem_q10_srt_rmdup_peaks_peak_24019
chr4 186768858 186769137
P19_NANOG_CNCC_D5_mem_q10_srt_rmdup_peaks_peak_24031
chr4 187597608 187598003
P19_NANOG_CNCC_D5_mem_q10_srt_rmdup_peaks_peak_24044
chr4 188916609 188916987
P19_NANOG_CNCC_D5_mem_q10_srt_rmdup_peaks_peak_24055
chr4 189394756 189395082
P19_NANOG_CNCC_D5_mem_q10_srt_rmdup_peaks_peak_24064
chr5 523579 523850
P19_NANOG_CNCC_D5_mem_q10_srt_rmdup_peaks_peak_24080
chr5 586186 586503
P19_NANOG_CNCC_D5_mem_q10_srt_rmdup_peaks_peak_24081
chr5 794157 794469
P19_NANOG_CNCC_D5_mem_q10_srt_rmdup_peaks_peak_24082
chr5 4942770 4943169
P19_NANOG_CNCC_D5_mem_q10_srt_rmdup_peaks_peak_24125
chr5 5365185 5365610
P19_NANOG_CNCC_D5_mem_q10_srt_rmdup_peaks_peak_24135
chr5 6712193 6712561
P19_NANOG_CNCC_D5_mem_q10_srt_rmdup_peaks_peak_24148
chr5 6713269 6713694
P19_NANOG_CNCC_D5_mem_q10_srt_rmdup_peaks_peak_24149
chr5 6821025 6821368

P19_NANOG_CNCC_D5_mem_q10_srt_rmdup_peaks_peak_24151
chr5 7261221 7261617
P19_NANOG_CNCC_D5_mem_q10_srt_rmdup_peaks_peak_24153
chr5 7519230 7519583
P19_NANOG_CNCC_D5_mem_q10_srt_rmdup_peaks_peak_24161
chr5 10091868 10092270
P19_NANOG_CNCC_D5_mem_q10_srt_rmdup_peaks_peak_24179
chr5 10102298 10102624
P19_NANOG_CNCC_D5_mem_q10_srt_rmdup_peaks_peak_24180
chr5 10339618 10339941
P19_NANOG_CNCC_D5_mem_q10_srt_rmdup_peaks_peak_24184
chr5 11538324 11538627
P19_NANOG_CNCC_D5_mem_q10_srt_rmdup_peaks_peak_24208
chr5 12865698 12866083
P19_NANOG_CNCC_D5_mem_q10_srt_rmdup_peaks_peak_24226
chr5 13505501 13505913
P19_NANOG_CNCC_D5_mem_q10_srt_rmdup_peaks_peak_24229
chr5 13942540 13942817
P19_NANOG_CNCC_D5_mem_q10_srt_rmdup_peaks_peak_24231
chr5 13943679 13943997
P19_NANOG_CNCC_D5_mem_q10_srt_rmdup_peaks_peak_24232
chr5 14230932 14231288
P19_NANOG_CNCC_D5_mem_q10_srt_rmdup_peaks_peak_24239
chr5 14238254 14238585
P19_NANOG_CNCC_D5_mem_q10_srt_rmdup_peaks_peak_24241
chr5 14340967 14341413
P19_NANOG_CNCC_D5_mem_q10_srt_rmdup_peaks_peak_24246
chr5 14741773 14742141
P19_NANOG_CNCC_D5_mem_q10_srt_rmdup_peaks_peak_24251
chr5 16774137 16774441
P19_NANOG_CNCC_D5_mem_q10_srt_rmdup_peaks_peak_24275
chr5 17014802 17015157
P19_NANOG_CNCC_D5_mem_q10_srt_rmdup_peaks_peak_24280
chr5 18459566 18459882
P19_NANOG_CNCC_D5_mem_q10_srt_rmdup_peaks_peak_24293
chr5 18798253 18798563
P19_NANOG_CNCC_D5_mem_q10_srt_rmdup_peaks_peak_24296
chr5 19777570 19777844
P19_NANOG_CNCC_D5_mem_q10_srt_rmdup_peaks_peak_24303
chr5 19915192 19915555
P19_NANOG_CNCC_D5_mem_q10_srt_rmdup_peaks_peak_24305
chr5 22138164 22138435
P19_NANOG_CNCC_D5_mem_q10_srt_rmdup_peaks_peak_24316
chr5 22611420 22611797
P19_NANOG_CNCC_D5_mem_q10_srt_rmdup_peaks_peak_24318
chr5 22855096 22855515
P19_NANOG_CNCC_D5_mem_q10_srt_rmdup_peaks_peak_24321
chr5 23848828 23849217
P19_NANOG_CNCC_D5_mem_q10_srt_rmdup_peaks_peak_24326
chr5 25558992 25559263

P19_NANOG_CNCC_D5_mem_q10_srt_rmdup_peaks_peak_24338
chr5 27387825 27388220
P19_NANOG_CNCC_D5_mem_q10_srt_rmdup_peaks_peak_24348
chr5 27548751 27549039
P19_NANOG_CNCC_D5_mem_q10_srt_rmdup_peaks_peak_24349
chr5 28516827 28517110
P19_NANOG_CNCC_D5_mem_q10_srt_rmdup_peaks_peak_24352
chr5 30522116 30522636
P19_NANOG_CNCC_D5_mem_q10_srt_rmdup_peaks_peak_24356
chr5 30959656 30959946
P19_NANOG_CNCC_D5_mem_q10_srt_rmdup_peaks_peak_24358
chr5 31161922 31162199
P19_NANOG_CNCC_D5_mem_q10_srt_rmdup_peaks_peak_24362
chr5 31453691 31454026
P19_NANOG_CNCC_D5_mem_q10_srt_rmdup_peaks_peak_24364
chr5 31856302 31856701
P19_NANOG_CNCC_D5_mem_q10_srt_rmdup_peaks_peak_24367
chr5 31992326 31992709
P19_NANOG_CNCC_D5_mem_q10_srt_rmdup_peaks_peak_24369
chr5 32251942 32252272
P19_NANOG_CNCC_D5_mem_q10_srt_rmdup_peaks_peak_24371
chr5 32288150 32288458
P19_NANOG_CNCC_D5_mem_q10_srt_rmdup_peaks_peak_24374
chr5 32443722 32444592
P19_NANOG_CNCC_D5_mem_q10_srt_rmdup_peaks_peak_24377
chr5 32851510 32852099
P19_NANOG_CNCC_D5_mem_q10_srt_rmdup_peaks_peak_24380
chr5 32852466 32852945
P19_NANOG_CNCC_D5_mem_q10_srt_rmdup_peaks_peak_24381
chr5 33189147 33189841
P19_NANOG_CNCC_D5_mem_q10_srt_rmdup_peaks_peak_24385
chr5 33363072 33363343
P19_NANOG_CNCC_D5_mem_q10_srt_rmdup_peaks_peak_24388
chr5 33863391 33863703
P19_NANOG_CNCC_D5_mem_q10_srt_rmdup_peaks_peak_24397
chr5 34415625 34415985
P19_NANOG_CNCC_D5_mem_q10_srt_rmdup_peaks_peak_24401
chr5 34654101 34654474
P19_NANOG_CNCC_D5_mem_q10_srt_rmdup_peaks_peak_24408
chr5 34839305 34839638
P19_NANOG_CNCC_D5_mem_q10_srt_rmdup_peaks_peak_24413
chr5 35358385 35358727
P19_NANOG_CNCC_D5_mem_q10_srt_rmdup_peaks_peak_24418
chr5 35770640 35771209
P19_NANOG_CNCC_D5_mem_q10_srt_rmdup_peaks_peak_24422
chr5 36242154 36242552
P19_NANOG_CNCC_D5_mem_q10_srt_rmdup_peaks_peak_24427
chr5 36596430 36596731
P19_NANOG_CNCC_D5_mem_q10_srt_rmdup_peaks_peak_24431
chr5 36693137 36693485

P19_NANOG_CNCC_D5_mem_q10_srt_rmdup_peaks_peak_24433
chr5 36874500 36874942
P19_NANOG_CNCC_D5_mem_q10_srt_rmdup_peaks_peak_24436
chr5 37090286 37090591
P19_NANOG_CNCC_D5_mem_q10_srt_rmdup_peaks_peak_24439
chr5 38916764 38917269
P19_NANOG_CNCC_D5_mem_q10_srt_rmdup_peaks_peak_24449
chr5 40141949 40142368
P19_NANOG_CNCC_D5_mem_q10_srt_rmdup_peaks_peak_24456
chr5 40801690 40801989
P19_NANOG_CNCC_D5_mem_q10_srt_rmdup_peaks_peak_24460
chr5 44808835 44809230
P19_NANOG_CNCC_D5_mem_q10_srt_rmdup_peaks_peak_24495
chr5 45097626 45097944
P19_NANOG_CNCC_D5_mem_q10_srt_rmdup_peaks_peak_24498
chr5 45192967 45193238
P19_NANOG_CNCC_D5_mem_q10_srt_rmdup_peaks_peak_24501
chr5 45419493 45419831
P19_NANOG_CNCC_D5_mem_q10_srt_rmdup_peaks_peak_24508
chr5 50575106 50575619
P19_NANOG_CNCC_D5_mem_q10_srt_rmdup_peaks_peak_24525
chr5 50816507 50816835
P19_NANOG_CNCC_D5_mem_q10_srt_rmdup_peaks_peak_24539
chr5 51412020 51412366
P19_NANOG_CNCC_D5_mem_q10_srt_rmdup_peaks_peak_24549
chr5 52754866 52755137
P19_NANOG_CNCC_D5_mem_q10_srt_rmdup_peaks_peak_24554
chr5 53317068 53317631
P19_NANOG_CNCC_D5_mem_q10_srt_rmdup_peaks_peak_24564
chr5 53388752 53389419
P19_NANOG_CNCC_D5_mem_q10_srt_rmdup_peaks_peak_24565
chr5 53476437 53476914
P19_NANOG_CNCC_D5_mem_q10_srt_rmdup_peaks_peak_24566
chr5 54321404 54321766
P19_NANOG_CNCC_D5_mem_q10_srt_rmdup_peaks_peak_24573
chr5 54934640 54934933
P19_NANOG_CNCC_D5_mem_q10_srt_rmdup_peaks_peak_24585
chr5 57246588 57247106
P19_NANOG_CNCC_D5_mem_q10_srt_rmdup_peaks_peak_24602
chr5 58777896 58778456
P19_NANOG_CNCC_D5_mem_q10_srt_rmdup_peaks_peak_24616
chr5 59232776 59233047
P19_NANOG_CNCC_D5_mem_q10_srt_rmdup_peaks_peak_24623
chr5 60075360 60075631
P19_NANOG_CNCC_D5_mem_q10_srt_rmdup_peaks_peak_24627
chr5 60202141 60202454
P19_NANOG_CNCC_D5_mem_q10_srt_rmdup_peaks_peak_24629
chr5 60667355 60667843
P19_NANOG_CNCC_D5_mem_q10_srt_rmdup_peaks_peak_24635
chr5 62529305 62529714

P19_NANOG_CNCC_D5_mem_q10_srt_rmdup_peaks_peak_24654
chr5 63801856 63802209
P19_NANOG_CNCC_D5_mem_q10_srt_rmdup_peaks_peak_24659
chr5 64333616 64333921
P19_NANOG_CNCC_D5_mem_q10_srt_rmdup_peaks_peak_24664
chr5 65236964 65237255
P19_NANOG_CNCC_D5_mem_q10_srt_rmdup_peaks_peak_24673
chr5 65861617 65862165
P19_NANOG_CNCC_D5_mem_q10_srt_rmdup_peaks_peak_24679
chr5 65951277 65951681
P19_NANOG_CNCC_D5_mem_q10_srt_rmdup_peaks_peak_24680
chr5 66110153 66110437
P19_NANOG_CNCC_D5_mem_q10_srt_rmdup_peaks_peak_24683
chr5 66332622 66333165
P19_NANOG_CNCC_D5_mem_q10_srt_rmdup_peaks_peak_24689
chr5 66627164 66627487
P19_NANOG_CNCC_D5_mem_q10_srt_rmdup_peaks_peak_24693
chr5 67140735 67141144
P19_NANOG_CNCC_D5_mem_q10_srt_rmdup_peaks_peak_24698
chr5 67256652 67256996
P19_NANOG_CNCC_D5_mem_q10_srt_rmdup_peaks_peak_24699
chr5 67583976 67584284
P19_NANOG_CNCC_D5_mem_q10_srt_rmdup_peaks_peak_24703
chr5 67751862 67752202
P19_NANOG_CNCC_D5_mem_q10_srt_rmdup_peaks_peak_24704
chr5 68710696 68711074
P19_NANOG_CNCC_D5_mem_q10_srt_rmdup_peaks_peak_24711
chr5 71441102 71441538
P19_NANOG_CNCC_D5_mem_q10_srt_rmdup_peaks_peak_24723
chr5 71797306 71797599
P19_NANOG_CNCC_D5_mem_q10_srt_rmdup_peaks_peak_24731
chr5 71852440 71852964
P19_NANOG_CNCC_D5_mem_q10_srt_rmdup_peaks_peak_24732
chr5 71930446 71930717
P19_NANOG_CNCC_D5_mem_q10_srt_rmdup_peaks_peak_24735
chr5 72400049 72400533
P19_NANOG_CNCC_D5_mem_q10_srt_rmdup_peaks_peak_24739
chr5 72586475 72586951
P19_NANOG_CNCC_D5_mem_q10_srt_rmdup_peaks_peak_24744
chr5 72617610 72618301
P19_NANOG_CNCC_D5_mem_q10_srt_rmdup_peaks_peak_24748
chr5 72789929 72790244
P19_NANOG_CNCC_D5_mem_q10_srt_rmdup_peaks_peak_24757
chr5 73913338 73913683
P19_NANOG_CNCC_D5_mem_q10_srt_rmdup_peaks_peak_24773
chr5 74238482 74238799
P19_NANOG_CNCC_D5_mem_q10_srt_rmdup_peaks_peak_24779
chr5 75823688 75823959
P19_NANOG_CNCC_D5_mem_q10_srt_rmdup_peaks_peak_24800
chr5 76441017 76441629

P19_NANOG_CNCC_D5_mem_q10_srt_rmdup_peaks_peak_24806
chr5 78223478 78223847
P19_NANOG_CNCC_D5_mem_q10_srt_rmdup_peaks_peak_24847
chr5 78550017 78550320
P19_NANOG_CNCC_D5_mem_q10_srt_rmdup_peaks_peak_24850
chr5 79489273 79489770
P19_NANOG_CNCC_D5_mem_q10_srt_rmdup_peaks_peak_24864
chr5 80543299 80543814
P19_NANOG_CNCC_D5_mem_q10_srt_rmdup_peaks_peak_24874
chr5 80548899 80549213
P19_NANOG_CNCC_D5_mem_q10_srt_rmdup_peaks_peak_24876
chr5 81017836 81018204
P19_NANOG_CNCC_D5_mem_q10_srt_rmdup_peaks_peak_24881
chr5 81315655 81315999
P19_NANOG_CNCC_D5_mem_q10_srt_rmdup_peaks_peak_24888
chr5 81521480 81521751
P19_NANOG_CNCC_D5_mem_q10_srt_rmdup_peaks_peak_24892
chr5 81600294 81600589
P19_NANOG_CNCC_D5_mem_q10_srt_rmdup_peaks_peak_24896
chr5 82395255 82395652
P19_NANOG_CNCC_D5_mem_q10_srt_rmdup_peaks_peak_24906
chr5 83010615 83011103
P19_NANOG_CNCC_D5_mem_q10_srt_rmdup_peaks_peak_24915
chr5 83020794 83021065
P19_NANOG_CNCC_D5_mem_q10_srt_rmdup_peaks_peak_24917
chr5 83409123 83409478
P19_NANOG_CNCC_D5_mem_q10_srt_rmdup_peaks_peak_24919
chr5 83979505 83979796
P19_NANOG_CNCC_D5_mem_q10_srt_rmdup_peaks_peak_24924
chr5 84574001 84574394
P19_NANOG_CNCC_D5_mem_q10_srt_rmdup_peaks_peak_24925
chr5 84824902 84825269
P19_NANOG_CNCC_D5_mem_q10_srt_rmdup_peaks_peak_24926
chr5 85095332 85095693
P19_NANOG_CNCC_D5_mem_q10_srt_rmdup_peaks_peak_24927
chr5 87145959 87146358
P19_NANOG_CNCC_D5_mem_q10_srt_rmdup_peaks_peak_24946
chr5 87388209 87388518
P19_NANOG_CNCC_D5_mem_q10_srt_rmdup_peaks_peak_24950
chr5 87685392 87685663
P19_NANOG_CNCC_D5_mem_q10_srt_rmdup_peaks_peak_24958
chr5 87783871 87784342
P19_NANOG_CNCC_D5_mem_q10_srt_rmdup_peaks_peak_24960
chr5 87847592 87848018
P19_NANOG_CNCC_D5_mem_q10_srt_rmdup_peaks_peak_24961
chr5 88902693 88903011
P19_NANOG_CNCC_D5_mem_q10_srt_rmdup_peaks_peak_24986
chr5 89702109 89702410
P19_NANOG_CNCC_D5_mem_q10_srt_rmdup_peaks_peak_24992
chr5 89853260 89853689

P19_NANOG_CNCC_D5_mem_q10_srt_rmdup_peaks_peak_24998
chr5 90033651 90034052
P19_NANOG_CNCC_D5_mem_q10_srt_rmdup_peaks_peak_25003
chr5 90172180 90172502
P19_NANOG_CNCC_D5_mem_q10_srt_rmdup_peaks_peak_25004
chr5 90496521 90496926
P19_NANOG_CNCC_D5_mem_q10_srt_rmdup_peaks_peak_25008
chr5 91040319 91040621
P19_NANOG_CNCC_D5_mem_q10_srt_rmdup_peaks_peak_25018
chr5 92010076 92010370
P19_NANOG_CNCC_D5_mem_q10_srt_rmdup_peaks_peak_25025
chr5 92455825 92456111
P19_NANOG_CNCC_D5_mem_q10_srt_rmdup_peaks_peak_25030
chr5 92909695 92910130
P19_NANOG_CNCC_D5_mem_q10_srt_rmdup_peaks_peak_25037
chr5 93059844 93060156
P19_NANOG_CNCC_D5_mem_q10_srt_rmdup_peaks_peak_25055
chr5 94509592 94509916
P19_NANOG_CNCC_D5_mem_q10_srt_rmdup_peaks_peak_25067
chr5 95431923 95432309
P19_NANOG_CNCC_D5_mem_q10_srt_rmdup_peaks_peak_25085
chr5 95503819 95504133
P19_NANOG_CNCC_D5_mem_q10_srt_rmdup_peaks_peak_25086
chr5 95621466 95621882
P19_NANOG_CNCC_D5_mem_q10_srt_rmdup_peaks_peak_25087
chr5 96126403 96127279
P19_NANOG_CNCC_D5_mem_q10_srt_rmdup_peaks_peak_25091
chr5 96447371 96447726
P19_NANOG_CNCC_D5_mem_q10_srt_rmdup_peaks_peak_25096
chr5 96861458 96861744
P19_NANOG_CNCC_D5_mem_q10_srt_rmdup_peaks_peak_25104
chr5 96911512 96911783
P19_NANOG_CNCC_D5_mem_q10_srt_rmdup_peaks_peak_25105
chr5 98610938 98611209
P19_NANOG_CNCC_D5_mem_q10_srt_rmdup_peaks_peak_25123
chr5 101025227 101025532
P19_NANOG_CNCC_D5_mem_q10_srt_rmdup_peaks_peak_25136
chr5 101065069 101065434
P19_NANOG_CNCC_D5_mem_q10_srt_rmdup_peaks_peak_25137
chr5 102445075 102445361
P19_NANOG_CNCC_D5_mem_q10_srt_rmdup_peaks_peak_25148
chr5 103601220 103601675
P19_NANOG_CNCC_D5_mem_q10_srt_rmdup_peaks_peak_25156
chr5 103691097 103691392
P19_NANOG_CNCC_D5_mem_q10_srt_rmdup_peaks_peak_25157
chr5 106314129 106314596
P19_NANOG_CNCC_D5_mem_q10_srt_rmdup_peaks_peak_25173
chr5 106319400 106319740
P19_NANOG_CNCC_D5_mem_q10_srt_rmdup_peaks_peak_25174
chr5 106577974 106578433

P19_NANOG_CNCC_D5_mem_q10_srt_rmdup_peaks_peak_25179
chr5 106648697 106649270
P19_NANOG_CNCC_D5_mem_q10_srt_rmdup_peaks_peak_25180
chr5 106854280 106854719
P19_NANOG_CNCC_D5_mem_q10_srt_rmdup_peaks_peak_25188
chr5 107268997 107269303
P19_NANOG_CNCC_D5_mem_q10_srt_rmdup_peaks_peak_25207
chr5 107410376 107410671
P19_NANOG_CNCC_D5_mem_q10_srt_rmdup_peaks_peak_25209
chr5 108079042 108079489
P19_NANOG_CNCC_D5_mem_q10_srt_rmdup_peaks_peak_25220
chr5 108221189 108221491
P19_NANOG_CNCC_D5_mem_q10_srt_rmdup_peaks_peak_25223
chr5 112023008 112023456
P19_NANOG_CNCC_D5_mem_q10_srt_rmdup_peaks_peak_25256
chr5 112571529 112571909
P19_NANOG_CNCC_D5_mem_q10_srt_rmdup_peaks_peak_25270
chr5 112970731 112971095
P19_NANOG_CNCC_D5_mem_q10_srt_rmdup_peaks_peak_25276
chr5 113723196 113723573
P19_NANOG_CNCC_D5_mem_q10_srt_rmdup_peaks_peak_25285
chr5 113793352 113793804
P19_NANOG_CNCC_D5_mem_q10_srt_rmdup_peaks_peak_25286
chr5 114506179 114506457
P19_NANOG_CNCC_D5_mem_q10_srt_rmdup_peaks_peak_25288
chr5 114591422 114591693
P19_NANOG_CNCC_D5_mem_q10_srt_rmdup_peaks_peak_25290
chr5 115021570 115021841
P19_NANOG_CNCC_D5_mem_q10_srt_rmdup_peaks_peak_25299
chr5 115697164 115697558
P19_NANOG_CNCC_D5_mem_q10_srt_rmdup_peaks_peak_25306
chr5 115976021 115976319
P19_NANOG_CNCC_D5_mem_q10_srt_rmdup_peaks_peak_25312
chr5 116157545 116157816
P19_NANOG_CNCC_D5_mem_q10_srt_rmdup_peaks_peak_25325
chr5 116203333 116203855
P19_NANOG_CNCC_D5_mem_q10_srt_rmdup_peaks_peak_25329
chr5 116439341 116439763
P19_NANOG_CNCC_D5_mem_q10_srt_rmdup_peaks_peak_25334
chr5 116760831 116761146
P19_NANOG_CNCC_D5_mem_q10_srt_rmdup_peaks_peak_25336
chr5 116773278 116773552
P19_NANOG_CNCC_D5_mem_q10_srt_rmdup_peaks_peak_25337
chr5 117002005 117002324
P19_NANOG_CNCC_D5_mem_q10_srt_rmdup_peaks_peak_25338
chr5 118254947 118255218
P19_NANOG_CNCC_D5_mem_q10_srt_rmdup_peaks_peak_25345
chr5 118385594 118386027
P19_NANOG_CNCC_D5_mem_q10_srt_rmdup_peaks_peak_25348
chr5 118916768 118917156

P19_NANOG_CNCC_D5_mem_q10_srt_rmdup_peaks_peak_25359
chr5	120814348	120814646
P19_NANOG_CNCC_D5_mem_q10_srt_rmdup_peaks_peak_25377
chr5	121295432	121295834
P19_NANOG_CNCC_D5_mem_q10_srt_rmdup_peaks_peak_25379
chr5	121297493	121297764
P19_NANOG_CNCC_D5_mem_q10_srt_rmdup_peaks_peak_25380
chr5	121355722	121356060
P19_NANOG_CNCC_D5_mem_q10_srt_rmdup_peaks_peak_25381
chr5	122333190	122333726
P19_NANOG_CNCC_D5_mem_q10_srt_rmdup_peaks_peak_25396
chr5	123644852	123645911
P19_NANOG_CNCC_D5_mem_q10_srt_rmdup_peaks_peak_25422
chr5	123908712	123909021
P19_NANOG_CNCC_D5_mem_q10_srt_rmdup_peaks_peak_25427
chr5	123909919	123910224
P19_NANOG_CNCC_D5_mem_q10_srt_rmdup_peaks_peak_25428
chr5	124536144	124536513
P19_NANOG_CNCC_D5_mem_q10_srt_rmdup_peaks_peak_25454
chr5	125534250	125534588
P19_NANOG_CNCC_D5_mem_q10_srt_rmdup_peaks_peak_25474
chr5	125758812	125759190
P19_NANOG_CNCC_D5_mem_q10_srt_rmdup_peaks_peak_25477
chr5	126009940	126010217
P19_NANOG_CNCC_D5_mem_q10_srt_rmdup_peaks_peak_25481
chr5	126237139	126237439
P19_NANOG_CNCC_D5_mem_q10_srt_rmdup_peaks_peak_25486
chr5	127772455	127772864
P19_NANOG_CNCC_D5_mem_q10_srt_rmdup_peaks_peak_25506
chr5	128017527	128017798
P19_NANOG_CNCC_D5_mem_q10_srt_rmdup_peaks_peak_25510
chr5	131337460	131337731
P19_NANOG_CNCC_D5_mem_q10_srt_rmdup_peaks_peak_25531
chr5	131629247	131629642
P19_NANOG_CNCC_D5_mem_q10_srt_rmdup_peaks_peak_25533
chr5	131690544	131690894
P19_NANOG_CNCC_D5_mem_q10_srt_rmdup_peaks_peak_25534
chr5	132090648	132090983
P19_NANOG_CNCC_D5_mem_q10_srt_rmdup_peaks_peak_25538
chr5	132299049	132299492
P19_NANOG_CNCC_D5_mem_q10_srt_rmdup_peaks_peak_25540
chr5	133561104	133561400
P19_NANOG_CNCC_D5_mem_q10_srt_rmdup_peaks_peak_25558
chr5	134485513	134485794
P19_NANOG_CNCC_D5_mem_q10_srt_rmdup_peaks_peak_25583
chr5	134682389	134682776
P19_NANOG_CNCC_D5_mem_q10_srt_rmdup_peaks_peak_25585
chr5	135546913	135547315
P19_NANOG_CNCC_D5_mem_q10_srt_rmdup_peaks_peak_25605
chr5	135700835	135701135

P19_NANOG_CNCC_D5_mem_q10_srt_rmdup_peaks_peak_25608
chr5 135727602 135728013
P19_NANOG_CNCC_D5_mem_q10_srt_rmdup_peaks_peak_25610
chr5 135736637 135737079
P19_NANOG_CNCC_D5_mem_q10_srt_rmdup_peaks_peak_25611
chr5 137805130 137806168
P19_NANOG_CNCC_D5_mem_q10_srt_rmdup_peaks_peak_25632
chr5 137859205 137859476
P19_NANOG_CNCC_D5_mem_q10_srt_rmdup_peaks_peak_25633
chr5 138307280 138307639
P19_NANOG_CNCC_D5_mem_q10_srt_rmdup_peaks_peak_25640
chr5 138531910 138532417
P19_NANOG_CNCC_D5_mem_q10_srt_rmdup_peaks_peak_25643
chr5 139012794 139013194
P19_NANOG_CNCC_D5_mem_q10_srt_rmdup_peaks_peak_25655
chr5 139384723 139384996
P19_NANOG_CNCC_D5_mem_q10_srt_rmdup_peaks_peak_25664
chr5 140514956 140515459
P19_NANOG_CNCC_D5_mem_q10_srt_rmdup_peaks_peak_25677
chr5 140557348 140557794
P19_NANOG_CNCC_D5_mem_q10_srt_rmdup_peaks_peak_25678
chr5 140624820 140625308
P19_NANOG_CNCC_D5_mem_q10_srt_rmdup_peaks_peak_25680
chr5 140792431 140793506
P19_NANOG_CNCC_D5_mem_q10_srt_rmdup_peaks_peak_25681
chr5 140856120 140856403
P19_NANOG_CNCC_D5_mem_q10_srt_rmdup_peaks_peak_25683
chr5 141225044 141225373
P19_NANOG_CNCC_D5_mem_q10_srt_rmdup_peaks_peak_25694
chr5 141554640 141554972
P19_NANOG_CNCC_D5_mem_q10_srt_rmdup_peaks_peak_25705
chr5 142975706 142975977
P19_NANOG_CNCC_D5_mem_q10_srt_rmdup_peaks_peak_25733
chr5 143116833 143117311
P19_NANOG_CNCC_D5_mem_q10_srt_rmdup_peaks_peak_25735
chr5 143204645 143205052
P19_NANOG_CNCC_D5_mem_q10_srt_rmdup_peaks_peak_25737
chr5 143867285 143867570
P19_NANOG_CNCC_D5_mem_q10_srt_rmdup_peaks_peak_25744
chr5 143904442 143904839
P19_NANOG_CNCC_D5_mem_q10_srt_rmdup_peaks_peak_25746
chr5 145458112 145458641
P19_NANOG_CNCC_D5_mem_q10_srt_rmdup_peaks_peak_25768
chr5 145774836 145775107
P19_NANOG_CNCC_D5_mem_q10_srt_rmdup_peaks_peak_25776
chr5 146204587 146204891
P19_NANOG_CNCC_D5_mem_q10_srt_rmdup_peaks_peak_25784
chr5 146219415 146220175
P19_NANOG_CNCC_D5_mem_q10_srt_rmdup_peaks_peak_25785
chr5 146547165 146547444

P19_NANOG_CNCC_D5_mem_q10_srt_rmdup_peaks_peak_25791
chr5 146614375 146614770
P19_NANOG_CNCC_D5_mem_q10_srt_rmdup_peaks_peak_25793
chr5 146830792 146831171
P19_NANOG_CNCC_D5_mem_q10_srt_rmdup_peaks_peak_25799
chr5 146833430 146833970
P19_NANOG_CNCC_D5_mem_q10_srt_rmdup_peaks_peak_25801
chr5 147048010 147048281
P19_NANOG_CNCC_D5_mem_q10_srt_rmdup_peaks_peak_25809
chr5 147128094 147128579
P19_NANOG_CNCC_D5_mem_q10_srt_rmdup_peaks_peak_25810
chr5 147314618 147314901
P19_NANOG_CNCC_D5_mem_q10_srt_rmdup_peaks_peak_25817
chr5 148941398 148941740
P19_NANOG_CNCC_D5_mem_q10_srt_rmdup_peaks_peak_25829
chr5 149016476 149016789
P19_NANOG_CNCC_D5_mem_q10_srt_rmdup_peaks_peak_25830
chr5 149338816 149339131
P19_NANOG_CNCC_D5_mem_q10_srt_rmdup_peaks_peak_25833
chr5 150036012 150036345
P19_NANOG_CNCC_D5_mem_q10_srt_rmdup_peaks_peak_25846
chr5 150180823 150181153
P19_NANOG_CNCC_D5_mem_q10_srt_rmdup_peaks_peak_25849
chr5 150460721 150461014
P19_NANOG_CNCC_D5_mem_q10_srt_rmdup_peaks_peak_25850
chr5 150926716 150927009
P19_NANOG_CNCC_D5_mem_q10_srt_rmdup_peaks_peak_25858
chr5 151548073 151548460
P19_NANOG_CNCC_D5_mem_q10_srt_rmdup_peaks_peak_25870
chr5 151838977 151839248
P19_NANOG_CNCC_D5_mem_q10_srt_rmdup_peaks_peak_25873
chr5 153038176 153038550
P19_NANOG_CNCC_D5_mem_q10_srt_rmdup_peaks_peak_25890
chr5 153162268 153162718
P19_NANOG_CNCC_D5_mem_q10_srt_rmdup_peaks_peak_25892
chr5 153545436 153545745
P19_NANOG_CNCC_D5_mem_q10_srt_rmdup_peaks_peak_25903
chr5 153989927 153990198
P19_NANOG_CNCC_D5_mem_q10_srt_rmdup_peaks_peak_25917
chr5 154317602 154317927
P19_NANOG_CNCC_D5_mem_q10_srt_rmdup_peaks_peak_25929
chr5 154320423 154320760
P19_NANOG_CNCC_D5_mem_q10_srt_rmdup_peaks_peak_25930
chr5 155577329 155577600
P19_NANOG_CNCC_D5_mem_q10_srt_rmdup_peaks_peak_25942
chr5 156112564 156113017
P19_NANOG_CNCC_D5_mem_q10_srt_rmdup_peaks_peak_25947
chr5 156147501 156147828
P19_NANOG_CNCC_D5_mem_q10_srt_rmdup_peaks_peak_25948
chr5 157170389 157170746

P19_NANOG_CNCC_D5_mem_q10_srt_rmdup_peaks_peak_25956
chr5	157898217	157898488
P19_NANOG_CNCC_D5_mem_q10_srt_rmdup_peaks_peak_25967
chr5	159743113	159743547
P19_NANOG_CNCC_D5_mem_q10_srt_rmdup_peaks_peak_25998
chr5	159767526	159767888
P19_NANOG_CNCC_D5_mem_q10_srt_rmdup_peaks_peak_25999
chr5	159865595	159866089
P19_NANOG_CNCC_D5_mem_q10_srt_rmdup_peaks_peak_26001
chr5	159912527	159912814
P19_NANOG_CNCC_D5_mem_q10_srt_rmdup_peaks_peak_26002
chr5	160200761	160201655
P19_NANOG_CNCC_D5_mem_q10_srt_rmdup_peaks_peak_26005
chr5	162457400	162457719
P19_NANOG_CNCC_D5_mem_q10_srt_rmdup_peaks_peak_26019
chr5	163942283	163942581
P19_NANOG_CNCC_D5_mem_q10_srt_rmdup_peaks_peak_26033
chr5	165707355	165707815
P19_NANOG_CNCC_D5_mem_q10_srt_rmdup_peaks_peak_26048
chr5	165796590	165797213
P19_NANOG_CNCC_D5_mem_q10_srt_rmdup_peaks_peak_26051
chr5	166199638	166199983
P19_NANOG_CNCC_D5_mem_q10_srt_rmdup_peaks_peak_26058
chr5	166492744	166493216
P19_NANOG_CNCC_D5_mem_q10_srt_rmdup_peaks_peak_26063
chr5	166805846	166806706
P19_NANOG_CNCC_D5_mem_q10_srt_rmdup_peaks_peak_26069
chr5	167545313	167545968
P19_NANOG_CNCC_D5_mem_q10_srt_rmdup_peaks_peak_26083
chr5	168316318	168316630
P19_NANOG_CNCC_D5_mem_q10_srt_rmdup_peaks_peak_26094
chr5	168440403	168440700
P19_NANOG_CNCC_D5_mem_q10_srt_rmdup_peaks_peak_26096
chr5	169334245	169334601
P19_NANOG_CNCC_D5_mem_q10_srt_rmdup_peaks_peak_26103
chr5	169678238	169678814
P19_NANOG_CNCC_D5_mem_q10_srt_rmdup_peaks_peak_26110
chr5	170947704	170947975
P19_NANOG_CNCC_D5_mem_q10_srt_rmdup_peaks_peak_26130
chr5	171094626	171095210
P19_NANOG_CNCC_D5_mem_q10_srt_rmdup_peaks_peak_26132
chr5	171433607	171434103
P19_NANOG_CNCC_D5_mem_q10_srt_rmdup_peaks_peak_26137
chr5	172781623	172782014
P19_NANOG_CNCC_D5_mem_q10_srt_rmdup_peaks_peak_26160
chr5	173433715	173433986
P19_NANOG_CNCC_D5_mem_q10_srt_rmdup_peaks_peak_26169
chr5	173962706	173963235
P19_NANOG_CNCC_D5_mem_q10_srt_rmdup_peaks_peak_26174
chr5	174102118	174102420

P19_NANOG_CNCC_D5_mem_q10_srt_rmdup_peaks_peak_26177
chr5 174803583 174804058
P19_NANOG_CNCC_D5_mem_q10_srt_rmdup_peaks_peak_26186
chr5 174863618 174864010
P19_NANOG_CNCC_D5_mem_q10_srt_rmdup_peaks_peak_26187
chr5 175000698 175001014
P19_NANOG_CNCC_D5_mem_q10_srt_rmdup_peaks_peak_26191
chr5 175014749 175015027
P19_NANOG_CNCC_D5_mem_q10_srt_rmdup_peaks_peak_26192
chr5 175970422 175970752
P19_NANOG_CNCC_D5_mem_q10_srt_rmdup_peaks_peak_26203
chr5 176882196 176882467
P19_NANOG_CNCC_D5_mem_q10_srt_rmdup_peaks_peak_26222
chr5 178450502 178450787
P19_NANOG_CNCC_D5_mem_q10_srt_rmdup_peaks_peak_26238
chr5 178487123 178487403
P19_NANOG_CNCC_D5_mem_q10_srt_rmdup_peaks_peak_26239
chr5 179414651 179414940
P19_NANOG_CNCC_D5_mem_q10_srt_rmdup_peaks_peak_26242
chr5 179434674 179434948
P19_NANOG_CNCC_D5_mem_q10_srt_rmdup_peaks_peak_26243
chr5 179477594 179477865
P19_NANOG_CNCC_D5_mem_q10_srt_rmdup_peaks_peak_26244
chr5 179498915 179499231
P19_NANOG_CNCC_D5_mem_q10_srt_rmdup_peaks_peak_26246
chr5 179662024 179662455
P19_NANOG_CNCC_D5_mem_q10_srt_rmdup_peaks_peak_26251
chr5 180046012 180046385
P19_NANOG_CNCC_D5_mem_q10_srt_rmdup_peaks_peak_26256
chr6 701670 702015
P19_NANOG_CNCC_D5_mem_q10_srt_rmdup_peaks_peak_26273
chr6 1055261 1055532
P19_NANOG_CNCC_D5_mem_q10_srt_rmdup_peaks_peak_26275
chr6 1105212 1105487
P19_NANOG_CNCC_D5_mem_q10_srt_rmdup_peaks_peak_26277
chr6 1380911 1381224
P19_NANOG_CNCC_D5_mem_q10_srt_rmdup_peaks_peak_26281
chr6 1463489 1463891
P19_NANOG_CNCC_D5_mem_q10_srt_rmdup_peaks_peak_26284
chr6 1919941 1920385
P19_NANOG_CNCC_D5_mem_q10_srt_rmdup_peaks_peak_26295
chr6 2197016 2197297
P19_NANOG_CNCC_D5_mem_q10_srt_rmdup_peaks_peak_26298
chr6 2970636 2971175
P19_NANOG_CNCC_D5_mem_q10_srt_rmdup_peaks_peak_26318
chr6 3157635 3157993
P19_NANOG_CNCC_D5_mem_q10_srt_rmdup_peaks_peak_26326
chr6 3251814 3252096
P19_NANOG_CNCC_D5_mem_q10_srt_rmdup_peaks_peak_26331
chr6 3861589 3862013

P19_NANOG_CNCC_D5_mem_q10_srt_rmdup_peaks_peak_26340
chr6	4133802	4134115
P19_NANOG_CNCC_D5_mem_q10_srt_rmdup_peaks_peak_26345
chr6	4486921	4487375
P19_NANOG_CNCC_D5_mem_q10_srt_rmdup_peaks_peak_26351
chr6	5192559	5192986
P19_NANOG_CNCC_D5_mem_q10_srt_rmdup_peaks_peak_26363
chr6	7110616	7111061
P19_NANOG_CNCC_D5_mem_q10_srt_rmdup_peaks_peak_26393
chr6	7156299	7156857
P19_NANOG_CNCC_D5_mem_q10_srt_rmdup_peaks_peak_26395
chr6	7544878	7545368
P19_NANOG_CNCC_D5_mem_q10_srt_rmdup_peaks_peak_26402
chr6	8064522	8064916
P19_NANOG_CNCC_D5_mem_q10_srt_rmdup_peaks_peak_26410
chr6	8336946	8337312
P19_NANOG_CNCC_D5_mem_q10_srt_rmdup_peaks_peak_26413
chr6	9474079	9474435
P19_NANOG_CNCC_D5_mem_q10_srt_rmdup_peaks_peak_26419
chr6	10480536	10480898
P19_NANOG_CNCC_D5_mem_q10_srt_rmdup_peaks_peak_26438
chr6	10537324	10537616
P19_NANOG_CNCC_D5_mem_q10_srt_rmdup_peaks_peak_26441
chr6	10574229	10574512
P19_NANOG_CNCC_D5_mem_q10_srt_rmdup_peaks_peak_26444
chr6	10954920	10955233
P19_NANOG_CNCC_D5_mem_q10_srt_rmdup_peaks_peak_26452
chr6	11429886	11430217
P19_NANOG_CNCC_D5_mem_q10_srt_rmdup_peaks_peak_26459
chr6	11609651	11609934
P19_NANOG_CNCC_D5_mem_q10_srt_rmdup_peaks_peak_26461
chr6	12336086	12336813
P19_NANOG_CNCC_D5_mem_q10_srt_rmdup_peaks_peak_26473
chr6	12840184	12840461
P19_NANOG_CNCC_D5_mem_q10_srt_rmdup_peaks_peak_26479
chr6	13187209	13187508
P19_NANOG_CNCC_D5_mem_q10_srt_rmdup_peaks_peak_26490
chr6	13328594	13329014
P19_NANOG_CNCC_D5_mem_q10_srt_rmdup_peaks_peak_26493
chr6	14055914	14056185
P19_NANOG_CNCC_D5_mem_q10_srt_rmdup_peaks_peak_26505
chr6	14305940	14306315
P19_NANOG_CNCC_D5_mem_q10_srt_rmdup_peaks_peak_26509
chr6	14789384	14789681
P19_NANOG_CNCC_D5_mem_q10_srt_rmdup_peaks_peak_26525
chr6	15415168	15415439
P19_NANOG_CNCC_D5_mem_q10_srt_rmdup_peaks_peak_26551
chr6	15492273	15492603
P19_NANOG_CNCC_D5_mem_q10_srt_rmdup_peaks_peak_26553
chr6	15897941	15898290

P19_NANOG_CNCC_D5_mem_q10_srt_rmdup_peaks_peak_26559
chr6 15949513 15949943
P19_NANOG_CNCC_D5_mem_q10_srt_rmdup_peaks_peak_26561
chr6 16331559 16331892
P19_NANOG_CNCC_D5_mem_q10_srt_rmdup_peaks_peak_26568
chr6 16332329 16332645
P19_NANOG_CNCC_D5_mem_q10_srt_rmdup_peaks_peak_26569
chr6 16965056 16965327
P19_NANOG_CNCC_D5_mem_q10_srt_rmdup_peaks_peak_26576
chr6 17706458 17706750
P19_NANOG_CNCC_D5_mem_q10_srt_rmdup_peaks_peak_26579
chr6 17706826 17707201
P19_NANOG_CNCC_D5_mem_q10_srt_rmdup_peaks_peak_26580
chr6 18197543 18197995
P19_NANOG_CNCC_D5_mem_q10_srt_rmdup_peaks_peak_26584
chr6 18264717 18265048
P19_NANOG_CNCC_D5_mem_q10_srt_rmdup_peaks_peak_26585
chr6 18295368 18295686
P19_NANOG_CNCC_D5_mem_q10_srt_rmdup_peaks_peak_26587
chr6 18488737 18489075
P19_NANOG_CNCC_D5_mem_q10_srt_rmdup_peaks_peak_26592
chr6 18990579 18990850
P19_NANOG_CNCC_D5_mem_q10_srt_rmdup_peaks_peak_26597
chr6 19247613 19248012
P19_NANOG_CNCC_D5_mem_q10_srt_rmdup_peaks_peak_26599
chr6 19811535 19812055
P19_NANOG_CNCC_D5_mem_q10_srt_rmdup_peaks_peak_26607
chr6 20811409 20811791
P19_NANOG_CNCC_D5_mem_q10_srt_rmdup_peaks_peak_26623
chr6 20933257 20933866
P19_NANOG_CNCC_D5_mem_q10_srt_rmdup_peaks_peak_26627
chr6 21922451 21922874
P19_NANOG_CNCC_D5_mem_q10_srt_rmdup_peaks_peak_26646
chr6 22367006 22367446
P19_NANOG_CNCC_D5_mem_q10_srt_rmdup_peaks_peak_26651
chr6 22447992 22448513
P19_NANOG_CNCC_D5_mem_q10_srt_rmdup_peaks_peak_26652
chr6 22466462 22467313
P19_NANOG_CNCC_D5_mem_q10_srt_rmdup_peaks_peak_26653
chr6 24126959 24127230
P19_NANOG_CNCC_D5_mem_q10_srt_rmdup_peaks_peak_26665
chr6 24405427 24405851
P19_NANOG_CNCC_D5_mem_q10_srt_rmdup_peaks_peak_26671
chr6 25016467 25016773
P19_NANOG_CNCC_D5_mem_q10_srt_rmdup_peaks_peak_26684
chr6 26029153 26029431
P19_NANOG_CNCC_D5_mem_q10_srt_rmdup_peaks_peak_26706
chr6 26198094 26198606
P19_NANOG_CNCC_D5_mem_q10_srt_rmdup_peaks_peak_26717
chr6 26272785 26273148

P19_NANOG_CNCC_D5_mem_q10_srt_rmdup_peaks_peak_26731
chr6 26327912 26328197
P19_NANOG_CNCC_D5_mem_q10_srt_rmdup_peaks_peak_26734
chr6 26341810 26342085
P19_NANOG_CNCC_D5_mem_q10_srt_rmdup_peaks_peak_26735
chr6 26474234 26474853
P19_NANOG_CNCC_D5_mem_q10_srt_rmdup_peaks_peak_26736
chr6 26476386 26476669
P19_NANOG_CNCC_D5_mem_q10_srt_rmdup_peaks_peak_26737
chr6 26682491 26682772
P19_NANOG_CNCC_D5_mem_q10_srt_rmdup_peaks_peak_26741
chr6 27034297 27034588
P19_NANOG_CNCC_D5_mem_q10_srt_rmdup_peaks_peak_26744
chr6 27114109 27114382
P19_NANOG_CNCC_D5_mem_q10_srt_rmdup_peaks_peak_26747
chr6 27509511 27509793
P19_NANOG_CNCC_D5_mem_q10_srt_rmdup_peaks_peak_26760
chr6 27560304 27560755
P19_NANOG_CNCC_D5_mem_q10_srt_rmdup_peaks_peak_26761
chr6 27735560 27736067
P19_NANOG_CNCC_D5_mem_q10_srt_rmdup_peaks_peak_26769
chr6 27782574 27783034
P19_NANOG_CNCC_D5_mem_q10_srt_rmdup_peaks_peak_26773
chr6 27838699 27839153
P19_NANOG_CNCC_D5_mem_q10_srt_rmdup_peaks_peak_26779
chr6 27859181 27859486
P19_NANOG_CNCC_D5_mem_q10_srt_rmdup_peaks_peak_26783
chr6 33548077 33548504
P19_NANOG_CNCC_D5_mem_q10_srt_rmdup_peaks_peak_26796
chr6 34004724 34005076
P19_NANOG_CNCC_D5_mem_q10_srt_rmdup_peaks_peak_26810
chr6 34954663 34955042
P19_NANOG_CNCC_D5_mem_q10_srt_rmdup_peaks_peak_26818
chr6 35037925 35038415
P19_NANOG_CNCC_D5_mem_q10_srt_rmdup_peaks_peak_26824
chr6 35475209 35475499
P19_NANOG_CNCC_D5_mem_q10_srt_rmdup_peaks_peak_26831
chr6 36461722 36462107
P19_NANOG_CNCC_D5_mem_q10_srt_rmdup_peaks_peak_26851
chr6 36646353 36646689
P19_NANOG_CNCC_D5_mem_q10_srt_rmdup_peaks_peak_26853
chr6 36754075 36754353
P19_NANOG_CNCC_D5_mem_q10_srt_rmdup_peaks_peak_26854
chr6 36915768 36916270
P19_NANOG_CNCC_D5_mem_q10_srt_rmdup_peaks_peak_26857
chr6 37072922 37073357
P19_NANOG_CNCC_D5_mem_q10_srt_rmdup_peaks_peak_26861
chr6 37112223 37112518
P19_NANOG_CNCC_D5_mem_q10_srt_rmdup_peaks_peak_26865
chr6 37791191 37791466

P19_NANOG_CNCC_D5_mem_q10_srt_rmdup_peaks_peak_26881
chr6 38165247 38165670
P19_NANOG_CNCC_D5_mem_q10_srt_rmdup_peaks_peak_26889
chr6 38229722 38230075
P19_NANOG_CNCC_D5_mem_q10_srt_rmdup_peaks_peak_26890
chr6 40492047 40492474
P19_NANOG_CNCC_D5_mem_q10_srt_rmdup_peaks_peak_26912
chr6 41262883 41263308
P19_NANOG_CNCC_D5_mem_q10_srt_rmdup_peaks_peak_26924
chr6 41528351 41528731
P19_NANOG_CNCC_D5_mem_q10_srt_rmdup_peaks_peak_26933
chr6 41541072 41541443
P19_NANOG_CNCC_D5_mem_q10_srt_rmdup_peaks_peak_26935
chr6 42145782 42146095
P19_NANOG_CNCC_D5_mem_q10_srt_rmdup_peaks_peak_26949
chr6 42386332 42386651
P19_NANOG_CNCC_D5_mem_q10_srt_rmdup_peaks_peak_26953
chr6 42585827 42586109
P19_NANOG_CNCC_D5_mem_q10_srt_rmdup_peaks_peak_26957
chr6 42723951 42724235
P19_NANOG_CNCC_D5_mem_q10_srt_rmdup_peaks_peak_26960
chr6 42758684 42758955
P19_NANOG_CNCC_D5_mem_q10_srt_rmdup_peaks_peak_26963
chr6 42847501 42847787
P19_NANOG_CNCC_D5_mem_q10_srt_rmdup_peaks_peak_26965
chr6 43043611 43043882
P19_NANOG_CNCC_D5_mem_q10_srt_rmdup_peaks_peak_26969
chr6 43364123 43364398
P19_NANOG_CNCC_D5_mem_q10_srt_rmdup_peaks_peak_26975
chr6 44284787 44285222
P19_NANOG_CNCC_D5_mem_q10_srt_rmdup_peaks_peak_26997
chr6 44440225 44440735
P19_NANOG_CNCC_D5_mem_q10_srt_rmdup_peaks_peak_26999
chr6 45413810 45414101
P19_NANOG_CNCC_D5_mem_q10_srt_rmdup_peaks_peak_27007
chr6 45534575 45535053
P19_NANOG_CNCC_D5_mem_q10_srt_rmdup_peaks_peak_27008
chr6 46403214 46403569
P19_NANOG_CNCC_D5_mem_q10_srt_rmdup_peaks_peak_27016
chr6 50832319 50832730
P19_NANOG_CNCC_D5_mem_q10_srt_rmdup_peaks_peak_27064
chr6 50946865 50947518
P19_NANOG_CNCC_D5_mem_q10_srt_rmdup_peaks_peak_27065
chr6 51415049 51415482
P19_NANOG_CNCC_D5_mem_q10_srt_rmdup_peaks_peak_27067
chr6 51773110 51773429
P19_NANOG_CNCC_D5_mem_q10_srt_rmdup_peaks_peak_27070
chr6 52796129 52796616
P19_NANOG_CNCC_D5_mem_q10_srt_rmdup_peaks_peak_27081
chr6 53406047 53406347

P19_NANOG_CNCC_D5_mem_q10_srt_rmdup_peaks_peak_27091
chr6 53452092 53452477
P19_NANOG_CNCC_D5_mem_q10_srt_rmdup_peaks_peak_27095
chr6 53865162 53865738
P19_NANOG_CNCC_D5_mem_q10_srt_rmdup_peaks_peak_27100
chr6 54255113 54255562
P19_NANOG_CNCC_D5_mem_q10_srt_rmdup_peaks_peak_27103
chr6 54443785 54444207
P19_NANOG_CNCC_D5_mem_q10_srt_rmdup_peaks_peak_27104
chr6 54847385 54847672
P19_NANOG_CNCC_D5_mem_q10_srt_rmdup_peaks_peak_27107
chr6 54853869 54854220
P19_NANOG_CNCC_D5_mem_q10_srt_rmdup_peaks_peak_27108
chr6 55115208 55115479
P19_NANOG_CNCC_D5_mem_q10_srt_rmdup_peaks_peak_27109
chr6 56616716 56617143
P19_NANOG_CNCC_D5_mem_q10_srt_rmdup_peaks_peak_27122
chr6 56934723 56935225
P19_NANOG_CNCC_D5_mem_q10_srt_rmdup_peaks_peak_27131
chr6 57286214 57286603
P19_NANOG_CNCC_D5_mem_q10_srt_rmdup_peaks_peak_27138
chr6 57336235 57336592
P19_NANOG_CNCC_D5_mem_q10_srt_rmdup_peaks_peak_27140
chr6 57540340 57540674
P19_NANOG_CNCC_D5_mem_q10_srt_rmdup_peaks_peak_27144
chr6 62350372 62350649
P19_NANOG_CNCC_D5_mem_q10_srt_rmdup_peaks_peak_27154
chr6 62469385 62470047
P19_NANOG_CNCC_D5_mem_q10_srt_rmdup_peaks_peak_27155
chr6 63614573 63614967
P19_NANOG_CNCC_D5_mem_q10_srt_rmdup_peaks_peak_27161
chr6 63946327 63946598
P19_NANOG_CNCC_D5_mem_q10_srt_rmdup_peaks_peak_27162
chr6 64247515 64247880
P19_NANOG_CNCC_D5_mem_q10_srt_rmdup_peaks_peak_27163
chr6 64726541 64726833
P19_NANOG_CNCC_D5_mem_q10_srt_rmdup_peaks_peak_27169
chr6 64990443 64990714
P19_NANOG_CNCC_D5_mem_q10_srt_rmdup_peaks_peak_27170
chr6 67372621 67373286
P19_NANOG_CNCC_D5_mem_q10_srt_rmdup_peaks_peak_27177
chr6 67719785 67720081
P19_NANOG_CNCC_D5_mem_q10_srt_rmdup_peaks_peak_27178
chr6 70341586 70342099
P19_NANOG_CNCC_D5_mem_q10_srt_rmdup_peaks_peak_27190
chr6 70623912 70624247
P19_NANOG_CNCC_D5_mem_q10_srt_rmdup_peaks_peak_27191
chr6 71383403 71383686
P19_NANOG_CNCC_D5_mem_q10_srt_rmdup_peaks_peak_27200
chr6 71864143 71864525

P19_NANOG_CNCC_D5_mem_q10_srt_rmdup_peaks_peak_27204
chr6 72165844 72166223
P19_NANOG_CNCC_D5_mem_q10_srt_rmdup_peaks_peak_27208
chr6 72770034 72770355
P19_NANOG_CNCC_D5_mem_q10_srt_rmdup_peaks_peak_27213
chr6 75913983 75914269
P19_NANOG_CNCC_D5_mem_q10_srt_rmdup_peaks_peak_27224
chr6 76459237 76459620
P19_NANOG_CNCC_D5_mem_q10_srt_rmdup_peaks_peak_27235
chr6 76655912 76656203
P19_NANOG_CNCC_D5_mem_q10_srt_rmdup_peaks_peak_27237
chr6 77036655 77036926
P19_NANOG_CNCC_D5_mem_q10_srt_rmdup_peaks_peak_27244
chr6 77190737 77191227
P19_NANOG_CNCC_D5_mem_q10_srt_rmdup_peaks_peak_27246
chr6 77545018 77545506
P19_NANOG_CNCC_D5_mem_q10_srt_rmdup_peaks_peak_27251
chr6 79109636 79110067
P19_NANOG_CNCC_D5_mem_q10_srt_rmdup_peaks_peak_27257
chr6 79928531 79928899
P19_NANOG_CNCC_D5_mem_q10_srt_rmdup_peaks_peak_27266
chr6 80204629 80204936
P19_NANOG_CNCC_D5_mem_q10_srt_rmdup_peaks_peak_27268
chr6 80519785 80520419
P19_NANOG_CNCC_D5_mem_q10_srt_rmdup_peaks_peak_27275
chr6 81914552 81914968
P19_NANOG_CNCC_D5_mem_q10_srt_rmdup_peaks_peak_27285
chr6 83073003 83073323
P19_NANOG_CNCC_D5_mem_q10_srt_rmdup_peaks_peak_27296
chr6 83505678 83506005
P19_NANOG_CNCC_D5_mem_q10_srt_rmdup_peaks_peak_27301
chr6 83821096 83821526
P19_NANOG_CNCC_D5_mem_q10_srt_rmdup_peaks_peak_27304
chr6 85038784 85039149
P19_NANOG_CNCC_D5_mem_q10_srt_rmdup_peaks_peak_27309
chr6 85483425 85483758
P19_NANOG_CNCC_D5_mem_q10_srt_rmdup_peaks_peak_27315
chr6 87221746 87222017
P19_NANOG_CNCC_D5_mem_q10_srt_rmdup_peaks_peak_27325
chr6 87748334 87748683
P19_NANOG_CNCC_D5_mem_q10_srt_rmdup_peaks_peak_27327
chr6 88154122 88154425
P19_NANOG_CNCC_D5_mem_q10_srt_rmdup_peaks_peak_27332
chr6 88182456 88182854
P19_NANOG_CNCC_D5_mem_q10_srt_rmdup_peaks_peak_27333
chr6 88678001 88678282
P19_NANOG_CNCC_D5_mem_q10_srt_rmdup_peaks_peak_27341
chr6 88893187 88893551
P19_NANOG_CNCC_D5_mem_q10_srt_rmdup_peaks_peak_27343
chr6 89200921 89201374

P19_NANOG_CNCC_D5_mem_q10_srt_rmdup_peaks_peak_27346
chr6 89673067 89673713
P19_NANOG_CNCC_D5_mem_q10_srt_rmdup_peaks_peak_27354
chr6 89844487 89844865
P19_NANOG_CNCC_D5_mem_q10_srt_rmdup_peaks_peak_27364
chr6 90096647 90097004
P19_NANOG_CNCC_D5_mem_q10_srt_rmdup_peaks_peak_27368
chr6 91074263 91074878
P19_NANOG_CNCC_D5_mem_q10_srt_rmdup_peaks_peak_27386
chr6 91199744 91200141
P19_NANOG_CNCC_D5_mem_q10_srt_rmdup_peaks_peak_27394
chr6 92126962 92127458
P19_NANOG_CNCC_D5_mem_q10_srt_rmdup_peaks_peak_27406
chr6 93464345 93464757
P19_NANOG_CNCC_D5_mem_q10_srt_rmdup_peaks_peak_27413
chr6 94163949 94164639
P19_NANOG_CNCC_D5_mem_q10_srt_rmdup_peaks_peak_27420
chr6 94539668 94540013
P19_NANOG_CNCC_D5_mem_q10_srt_rmdup_peaks_peak_27423
chr6 95968416 95968724
P19_NANOG_CNCC_D5_mem_q10_srt_rmdup_peaks_peak_27433
chr6 96969591 96969862
P19_NANOG_CNCC_D5_mem_q10_srt_rmdup_peaks_peak_27441
chr6 97972691 97973171
P19_NANOG_CNCC_D5_mem_q10_srt_rmdup_peaks_peak_27447
chr6 99092008 99092523
P19_NANOG_CNCC_D5_mem_q10_srt_rmdup_peaks_peak_27466
chr6 99120551 99120996
P19_NANOG_CNCC_D5_mem_q10_srt_rmdup_peaks_peak_27467
chr6 99172410 99172720
P19_NANOG_CNCC_D5_mem_q10_srt_rmdup_peaks_peak_27469
chr6 99841739 99842082
P19_NANOG_CNCC_D5_mem_q10_srt_rmdup_peaks_peak_27481
chr6 99936226 99936554
P19_NANOG_CNCC_D5_mem_q10_srt_rmdup_peaks_peak_27483
chr6 100250344 100250627
P19_NANOG_CNCC_D5_mem_q10_srt_rmdup_peaks_peak_27496
chr6 100391662 100392087
P19_NANOG_CNCC_D5_mem_q10_srt_rmdup_peaks_peak_27498
chr6 101289661 101290017
P19_NANOG_CNCC_D5_mem_q10_srt_rmdup_peaks_peak_27515
chr6 101325587 101326158
P19_NANOG_CNCC_D5_mem_q10_srt_rmdup_peaks_peak_27516
chr6 101538050 101538496
P19_NANOG_CNCC_D5_mem_q10_srt_rmdup_peaks_peak_27521
chr6 102617833 102618104
P19_NANOG_CNCC_D5_mem_q10_srt_rmdup_peaks_peak_27531
chr6 104096160 104096431
P19_NANOG_CNCC_D5_mem_q10_srt_rmdup_peaks_peak_27535
chr6 105259509 105259843

P19_NANOG_CNCC_D5_mem_q10_srt_rmdup_peaks_peak_27539
chr6 105901892 105902321
P19_NANOG_CNCC_D5_mem_q10_srt_rmdup_peaks_peak_27548
chr6 106317699 106318065
P19_NANOG_CNCC_D5_mem_q10_srt_rmdup_peaks_peak_27553
chr6 106528389 106529006
P19_NANOG_CNCC_D5_mem_q10_srt_rmdup_peaks_peak_27562
chr6 106535809 106536204
P19_NANOG_CNCC_D5_mem_q10_srt_rmdup_peaks_peak_27564
chr6 106773442 106773738
P19_NANOG_CNCC_D5_mem_q10_srt_rmdup_peaks_peak_27567
chr6 107033374 107033833
P19_NANOG_CNCC_D5_mem_q10_srt_rmdup_peaks_peak_27569
chr6 107230799 107231288
P19_NANOG_CNCC_D5_mem_q10_srt_rmdup_peaks_peak_27577
chr6 107635809 107636254
P19_NANOG_CNCC_D5_mem_q10_srt_rmdup_peaks_peak_27585
chr6 107894102 107894520
P19_NANOG_CNCC_D5_mem_q10_srt_rmdup_peaks_peak_27590
chr6 108324192 108324503
P19_NANOG_CNCC_D5_mem_q10_srt_rmdup_peaks_peak_27597
chr6 108599802 108600117
P19_NANOG_CNCC_D5_mem_q10_srt_rmdup_peaks_peak_27614
chr6 109058062 109058361
P19_NANOG_CNCC_D5_mem_q10_srt_rmdup_peaks_peak_27622
chr6 110177115 110177394
P19_NANOG_CNCC_D5_mem_q10_srt_rmdup_peaks_peak_27635
chr6 110223531 110223824
P19_NANOG_CNCC_D5_mem_q10_srt_rmdup_peaks_peak_27637
chr6 111197429 111197820
P19_NANOG_CNCC_D5_mem_q10_srt_rmdup_peaks_peak_27649
chr6 111408252 111408768
P19_NANOG_CNCC_D5_mem_q10_srt_rmdup_peaks_peak_27650
chr6 112243637 112243978
P19_NANOG_CNCC_D5_mem_q10_srt_rmdup_peaks_peak_27675
chr6 112306440 112306829
P19_NANOG_CNCC_D5_mem_q10_srt_rmdup_peaks_peak_27676
chr6 112520112 112520865
P19_NANOG_CNCC_D5_mem_q10_srt_rmdup_peaks_peak_27680
chr6 112848786 112849066
P19_NANOG_CNCC_D5_mem_q10_srt_rmdup_peaks_peak_27685
chr6 113180533 113180905
P19_NANOG_CNCC_D5_mem_q10_srt_rmdup_peaks_peak_27687
chr6 113964318 113964616
P19_NANOG_CNCC_D5_mem_q10_srt_rmdup_peaks_peak_27697
chr6 114179818 114180365
P19_NANOG_CNCC_D5_mem_q10_srt_rmdup_peaks_peak_27701
chr6 114650605 114650978
P19_NANOG_CNCC_D5_mem_q10_srt_rmdup_peaks_peak_27707
chr6 114898311 114898680

P19_NANOG_CNCC_D5_mem_q10_srt_rmdup_peaks_peak_27715
chr6	115989779	115990067
P19_NANOG_CNCC_D5_mem_q10_srt_rmdup_peaks_peak_27724
chr6	116454587	116455025
P19_NANOG_CNCC_D5_mem_q10_srt_rmdup_peaks_peak_27726
chr6	116592414	116592707
P19_NANOG_CNCC_D5_mem_q10_srt_rmdup_peaks_peak_27728
chr6	117632840	117633275
P19_NANOG_CNCC_D5_mem_q10_srt_rmdup_peaks_peak_27742
chr6	117869061	117869369
P19_NANOG_CNCC_D5_mem_q10_srt_rmdup_peaks_peak_27745
chr6	117996267	117996593
P19_NANOG_CNCC_D5_mem_q10_srt_rmdup_peaks_peak_27746
chr6	118170266	118170664
P19_NANOG_CNCC_D5_mem_q10_srt_rmdup_peaks_peak_27749
chr6	118442649	118443075
P19_NANOG_CNCC_D5_mem_q10_srt_rmdup_peaks_peak_27756
chr6	119090061	119090466
P19_NANOG_CNCC_D5_mem_q10_srt_rmdup_peaks_peak_27759
chr6	119215001	119215340
P19_NANOG_CNCC_D5_mem_q10_srt_rmdup_peaks_peak_27760
chr6	119617100	119617520
P19_NANOG_CNCC_D5_mem_q10_srt_rmdup_peaks_peak_27764
chr6	119630468	119630853
P19_NANOG_CNCC_D5_mem_q10_srt_rmdup_peaks_peak_27765
chr6	121761414	121761772
P19_NANOG_CNCC_D5_mem_q10_srt_rmdup_peaks_peak_27778
chr6	121765078	121765392
P19_NANOG_CNCC_D5_mem_q10_srt_rmdup_peaks_peak_27779
chr6	122931070	122931631
P19_NANOG_CNCC_D5_mem_q10_srt_rmdup_peaks_peak_27793
chr6	123013592	123014171
P19_NANOG_CNCC_D5_mem_q10_srt_rmdup_peaks_peak_27794
chr6	123018637	123018974
P19_NANOG_CNCC_D5_mem_q10_srt_rmdup_peaks_peak_27795
chr6	123154190	123154811
P19_NANOG_CNCC_D5_mem_q10_srt_rmdup_peaks_peak_27798
chr6	124479219	124479490
P19_NANOG_CNCC_D5_mem_q10_srt_rmdup_peaks_peak_27803
chr6	125205734	125206027
P19_NANOG_CNCC_D5_mem_q10_srt_rmdup_peaks_peak_27807
chr6	125323002	125323673
P19_NANOG_CNCC_D5_mem_q10_srt_rmdup_peaks_peak_27808
chr6	125635246	125635563
P19_NANOG_CNCC_D5_mem_q10_srt_rmdup_peaks_peak_27813
chr6	125677988	125678455
P19_NANOG_CNCC_D5_mem_q10_srt_rmdup_peaks_peak_27814
chr6	125932881	125933171
P19_NANOG_CNCC_D5_mem_q10_srt_rmdup_peaks_peak_27820
chr6	126028698	126029090

P19_NANOG_CNCC_D5_mem_q10_srt_rmdup_peaks_peak_27823
chr6 126501715 126502249
P19_NANOG_CNCC_D5_mem_q10_srt_rmdup_peaks_peak_27836
chr6 127297364 127297838
P19_NANOG_CNCC_D5_mem_q10_srt_rmdup_peaks_peak_27841
chr6 127411139 127411540
P19_NANOG_CNCC_D5_mem_q10_srt_rmdup_peaks_peak_27842
chr6 127439848 127440260
P19_NANOG_CNCC_D5_mem_q10_srt_rmdup_peaks_peak_27843
chr6 127587754 127588041
P19_NANOG_CNCC_D5_mem_q10_srt_rmdup_peaks_peak_27844
chr6 128343119 128343510
P19_NANOG_CNCC_D5_mem_q10_srt_rmdup_peaks_peak_27850
chr6 128720729 128721010
P19_NANOG_CNCC_D5_mem_q10_srt_rmdup_peaks_peak_27852
chr6 128890893 128891191
P19_NANOG_CNCC_D5_mem_q10_srt_rmdup_peaks_peak_27855
chr6 129275783 129276256
P19_NANOG_CNCC_D5_mem_q10_srt_rmdup_peaks_peak_27861
chr6 129284905 129285454
P19_NANOG_CNCC_D5_mem_q10_srt_rmdup_peaks_peak_27862
chr6 129516733 129517153
P19_NANOG_CNCC_D5_mem_q10_srt_rmdup_peaks_peak_27865
chr6 130322277 130322741
P19_NANOG_CNCC_D5_mem_q10_srt_rmdup_peaks_peak_27873
chr6 130727943 130728214
P19_NANOG_CNCC_D5_mem_q10_srt_rmdup_peaks_peak_27875
chr6 131189886 131190375
P19_NANOG_CNCC_D5_mem_q10_srt_rmdup_peaks_peak_27878
chr6 131224247 131224581
P19_NANOG_CNCC_D5_mem_q10_srt_rmdup_peaks_peak_27879
chr6 131643253 131643748
P19_NANOG_CNCC_D5_mem_q10_srt_rmdup_peaks_peak_27886
chr6 131665667 131665952
P19_NANOG_CNCC_D5_mem_q10_srt_rmdup_peaks_peak_27888
chr6 131722315 131722586
P19_NANOG_CNCC_D5_mem_q10_srt_rmdup_peaks_peak_27889
chr6 133376744 133377080
P19_NANOG_CNCC_D5_mem_q10_srt_rmdup_peaks_peak_27901
chr6 133634162 133634615
P19_NANOG_CNCC_D5_mem_q10_srt_rmdup_peaks_peak_27904
chr6 134444933 134445204
P19_NANOG_CNCC_D5_mem_q10_srt_rmdup_peaks_peak_27922
chr6 134700235 134700664
P19_NANOG_CNCC_D5_mem_q10_srt_rmdup_peaks_peak_27927
chr6 135095346 135095643
P19_NANOG_CNCC_D5_mem_q10_srt_rmdup_peaks_peak_27934
chr6 135505095 135505366
P19_NANOG_CNCC_D5_mem_q10_srt_rmdup_peaks_peak_27940
chr6 135824958 135825319

P19_NANOG_CNCC_D5_mem_q10_srt_rmdup_peaks_peak_27944
chr6	136823385	136823742
P19_NANOG_CNCC_D5_mem_q10_srt_rmdup_peaks_peak_27949
chr6	137283820	137284176
P19_NANOG_CNCC_D5_mem_q10_srt_rmdup_peaks_peak_27957
chr6	138497575	138498030
P19_NANOG_CNCC_D5_mem_q10_srt_rmdup_peaks_peak_27984
chr6	138807432	138807703
P19_NANOG_CNCC_D5_mem_q10_srt_rmdup_peaks_peak_27989
chr6	138884686	138885150
P19_NANOG_CNCC_D5_mem_q10_srt_rmdup_peaks_peak_27992
chr6	138891806	138892117
P19_NANOG_CNCC_D5_mem_q10_srt_rmdup_peaks_peak_27993
chr6	138911960	138912350
P19_NANOG_CNCC_D5_mem_q10_srt_rmdup_peaks_peak_27995
chr6	139665736	139666020
P19_NANOG_CNCC_D5_mem_q10_srt_rmdup_peaks_peak_28001
chr6	139692781	139693443
P19_NANOG_CNCC_D5_mem_q10_srt_rmdup_peaks_peak_28004
chr6	140285974	140286276
P19_NANOG_CNCC_D5_mem_q10_srt_rmdup_peaks_peak_28015
chr6	140302928	140303272
P19_NANOG_CNCC_D5_mem_q10_srt_rmdup_peaks_peak_28016
chr6	144011108	144011412
P19_NANOG_CNCC_D5_mem_q10_srt_rmdup_peaks_peak_28046
chr6	144651812	144652226
P19_NANOG_CNCC_D5_mem_q10_srt_rmdup_peaks_peak_28068
chr6	144933962	144934248
P19_NANOG_CNCC_D5_mem_q10_srt_rmdup_peaks_peak_28070
chr6	145167678	145168116
P19_NANOG_CNCC_D5_mem_q10_srt_rmdup_peaks_peak_28076
chr6	145460513	145460991
P19_NANOG_CNCC_D5_mem_q10_srt_rmdup_peaks_peak_28084
chr6	146338793	146339186
P19_NANOG_CNCC_D5_mem_q10_srt_rmdup_peaks_peak_28090
chr6	147457189	147457610
P19_NANOG_CNCC_D5_mem_q10_srt_rmdup_peaks_peak_28100
chr6	149310741	149311075
P19_NANOG_CNCC_D5_mem_q10_srt_rmdup_peaks_peak_28123
chr6	150205418	150205689
P19_NANOG_CNCC_D5_mem_q10_srt_rmdup_peaks_peak_28127
chr6	151187386	151187660
P19_NANOG_CNCC_D5_mem_q10_srt_rmdup_peaks_peak_28133
chr6	153174175	153174720
P19_NANOG_CNCC_D5_mem_q10_srt_rmdup_peaks_peak_28148
chr6	153633675	153634085
P19_NANOG_CNCC_D5_mem_q10_srt_rmdup_peaks_peak_28155
chr6	154360529	154360802
P19_NANOG_CNCC_D5_mem_q10_srt_rmdup_peaks_peak_28163
chr6	154779061	154779423

P19_NANOG_CNCC_D5_mem_q10_srt_rmdup_peaks_peak_28168
chr6 154858658 154859034
P19_NANOG_CNCC_D5_mem_q10_srt_rmdup_peaks_peak_28171
chr6 155142872 155143143
P19_NANOG_CNCC_D5_mem_q10_srt_rmdup_peaks_peak_28177
chr6 155200658 155200969
P19_NANOG_CNCC_D5_mem_q10_srt_rmdup_peaks_peak_28180
chr6 155574203 155574908
P19_NANOG_CNCC_D5_mem_q10_srt_rmdup_peaks_peak_28188
chr6 155582714 155583003
P19_NANOG_CNCC_D5_mem_q10_srt_rmdup_peaks_peak_28189
chr6 156718168 156718439
P19_NANOG_CNCC_D5_mem_q10_srt_rmdup_peaks_peak_28199
chr6 156824303 156824660
P19_NANOG_CNCC_D5_mem_q10_srt_rmdup_peaks_peak_28200
chr6 157141278 157141555
P19_NANOG_CNCC_D5_mem_q10_srt_rmdup_peaks_peak_28206
chr6 157358074 157358374
P19_NANOG_CNCC_D5_mem_q10_srt_rmdup_peaks_peak_28210
chr6 157391728 157392097
P19_NANOG_CNCC_D5_mem_q10_srt_rmdup_peaks_peak_28212
chr6 157849603 157849974
P19_NANOG_CNCC_D5_mem_q10_srt_rmdup_peaks_peak_28219
chr6 158764891 158765329
P19_NANOG_CNCC_D5_mem_q10_srt_rmdup_peaks_peak_28235
chr6 159420802 159421247
P19_NANOG_CNCC_D5_mem_q10_srt_rmdup_peaks_peak_28246
chr6 159477469 159477837
P19_NANOG_CNCC_D5_mem_q10_srt_rmdup_peaks_peak_28247
chr6 159673912 159674307
P19_NANOG_CNCC_D5_mem_q10_srt_rmdup_peaks_peak_28248
chr6 160003608 160003996
P19_NANOG_CNCC_D5_mem_q10_srt_rmdup_peaks_peak_28249
chr6 160114605 160114876
P19_NANOG_CNCC_D5_mem_q10_srt_rmdup_peaks_peak_28250
chr6 160400369 160400743
P19_NANOG_CNCC_D5_mem_q10_srt_rmdup_peaks_peak_28255
chr6 161516243 161516784
P19_NANOG_CNCC_D5_mem_q10_srt_rmdup_peaks_peak_28270
chr6 161714222 161714647
P19_NANOG_CNCC_D5_mem_q10_srt_rmdup_peaks_peak_28277
chr6 161774404 161774706
P19_NANOG_CNCC_D5_mem_q10_srt_rmdup_peaks_peak_28278
chr6 162039841 162040112
P19_NANOG_CNCC_D5_mem_q10_srt_rmdup_peaks_peak_28281
chr6 165177326 165177618
P19_NANOG_CNCC_D5_mem_q10_srt_rmdup_peaks_peak_28309
chr6 166903012 166903483
P19_NANOG_CNCC_D5_mem_q10_srt_rmdup_peaks_peak_28320
chr6 167564707 167565095

P19_NANOG_CNCC_D5_mem_q10_srt_rmdup_peaks_peak_28331
chr6 168262044 168262518
P19_NANOG_CNCC_D5_mem_q10_srt_rmdup_peaks_peak_28347
chr6 168280612 168280883
P19_NANOG_CNCC_D5_mem_q10_srt_rmdup_peaks_peak_28348
chr6 168334035 168334317
P19_NANOG_CNCC_D5_mem_q10_srt_rmdup_peaks_peak_28350
chr6 168380450 168380744
P19_NANOG_CNCC_D5_mem_q10_srt_rmdup_peaks_peak_28351
chr6 169083922 169084430
P19_NANOG_CNCC_D5_mem_q10_srt_rmdup_peaks_peak_28366
chr7 436980 437251
P19_NANOG_CNCC_D5_mem_q10_srt_rmdup_peaks_peak_28405
chr7 474853 475225
P19_NANOG_CNCC_D5_mem_q10_srt_rmdup_peaks_peak_28407
chr7 1329590 1329911
P19_NANOG_CNCC_D5_mem_q10_srt_rmdup_peaks_peak_28426
chr7 3967439 3967786
P19_NANOG_CNCC_D5_mem_q10_srt_rmdup_peaks_peak_28461
chr7 4784353 4784791
P19_NANOG_CNCC_D5_mem_q10_srt_rmdup_peaks_peak_28471
chr7 5305938 5306209
P19_NANOG_CNCC_D5_mem_q10_srt_rmdup_peaks_peak_28475
chr7 7100675 7101181
P19_NANOG_CNCC_D5_mem_q10_srt_rmdup_peaks_peak_28500
chr7 7747468 7747806
P19_NANOG_CNCC_D5_mem_q10_srt_rmdup_peaks_peak_28515
chr7 7976132 7976412
P19_NANOG_CNCC_D5_mem_q10_srt_rmdup_peaks_peak_28521
chr7 8225656 8225996
P19_NANOG_CNCC_D5_mem_q10_srt_rmdup_peaks_peak_28524
chr7 10123542 10123910
P19_NANOG_CNCC_D5_mem_q10_srt_rmdup_peaks_peak_28534
chr7 10619150 10619592
P19_NANOG_CNCC_D5_mem_q10_srt_rmdup_peaks_peak_28536
chr7 10658582 10658853
P19_NANOG_CNCC_D5_mem_q10_srt_rmdup_peaks_peak_28537
chr7 14038770 14039244
P19_NANOG_CNCC_D5_mem_q10_srt_rmdup_peaks_peak_28576
chr7 14104913 14105184
P19_NANOG_CNCC_D5_mem_q10_srt_rmdup_peaks_peak_28577
chr7 14311126 14311492
P19_NANOG_CNCC_D5_mem_q10_srt_rmdup_peaks_peak_28579
chr7 14443132 14443565
P19_NANOG_CNCC_D5_mem_q10_srt_rmdup_peaks_peak_28582
chr7 14665748 14666019
P19_NANOG_CNCC_D5_mem_q10_srt_rmdup_peaks_peak_28585
chr7 16106659 16107056
P19_NANOG_CNCC_D5_mem_q10_srt_rmdup_peaks_peak_28592
chr7 17424180 17424548

P19_NANOG_CNCC_D5_mem_q10_srt_rmdup_peaks_peak_28605
chr7 17979673 17980271
P19_NANOG_CNCC_D5_mem_q10_srt_rmdup_peaks_peak_28612
chr7 18447086 18447520
P19_NANOG_CNCC_D5_mem_q10_srt_rmdup_peaks_peak_28617
chr7 18950079 18950498
P19_NANOG_CNCC_D5_mem_q10_srt_rmdup_peaks_peak_28621
chr7 19140157 19140439
P19_NANOG_CNCC_D5_mem_q10_srt_rmdup_peaks_peak_28623
chr7 20165579 20166022
P19_NANOG_CNCC_D5_mem_q10_srt_rmdup_peaks_peak_28637
chr7 20304178 20304725
P19_NANOG_CNCC_D5_mem_q10_srt_rmdup_peaks_peak_28638
chr7 20347392 20347674
P19_NANOG_CNCC_D5_mem_q10_srt_rmdup_peaks_peak_28640
chr7 20369925 20370268
P19_NANOG_CNCC_D5_mem_q10_srt_rmdup_peaks_peak_28642
chr7 20482999 20483490
P19_NANOG_CNCC_D5_mem_q10_srt_rmdup_peaks_peak_28645
chr7 21294596 21295054
P19_NANOG_CNCC_D5_mem_q10_srt_rmdup_peaks_peak_28667
chr7 21644803 21645212
P19_NANOG_CNCC_D5_mem_q10_srt_rmdup_peaks_peak_28676
chr7 22221326 22221846
P19_NANOG_CNCC_D5_mem_q10_srt_rmdup_peaks_peak_28688
chr7 22237232 22237503
P19_NANOG_CNCC_D5_mem_q10_srt_rmdup_peaks_peak_28690
chr7 22290883 22291255
P19_NANOG_CNCC_D5_mem_q10_srt_rmdup_peaks_peak_28691
chr7 22451463 22451904
P19_NANOG_CNCC_D5_mem_q10_srt_rmdup_peaks_peak_28696
chr7 22474707 22475105
P19_NANOG_CNCC_D5_mem_q10_srt_rmdup_peaks_peak_28698
chr7 22882347 22882646
P19_NANOG_CNCC_D5_mem_q10_srt_rmdup_peaks_peak_28704
chr7 22893913 22894227
P19_NANOG_CNCC_D5_mem_q10_srt_rmdup_peaks_peak_28705
chr7 23157492 23157763
P19_NANOG_CNCC_D5_mem_q10_srt_rmdup_peaks_peak_28712
chr7 23327314 23327927
P19_NANOG_CNCC_D5_mem_q10_srt_rmdup_peaks_peak_28714
chr7 24064554 24065053
P19_NANOG_CNCC_D5_mem_q10_srt_rmdup_peaks_peak_28727
chr7 24606813 24607407
P19_NANOG_CNCC_D5_mem_q10_srt_rmdup_peaks_peak_28738
chr7 25343058 25343329
P19_NANOG_CNCC_D5_mem_q10_srt_rmdup_peaks_peak_28750
chr7 25615132 25615649
P19_NANOG_CNCC_D5_mem_q10_srt_rmdup_peaks_peak_28752
chr7 25783668 25783943

P19_NANOG_CNCC_D5_mem_q10_srt_rmdup_peaks_peak_28756
chr7 25905497 25905978
P19_NANOG_CNCC_D5_mem_q10_srt_rmdup_peaks_peak_28761
chr7 25935239 25935706
P19_NANOG_CNCC_D5_mem_q10_srt_rmdup_peaks_peak_28762
chr7 26062074 26062348
P19_NANOG_CNCC_D5_mem_q10_srt_rmdup_peaks_peak_28764
chr7 26188221 26188707
P19_NANOG_CNCC_D5_mem_q10_srt_rmdup_peaks_peak_28767
chr7 26241761 26242063
P19_NANOG_CNCC_D5_mem_q10_srt_rmdup_peaks_peak_28772
chr7 26460167 26460522
P19_NANOG_CNCC_D5_mem_q10_srt_rmdup_peaks_peak_28774
chr7 27286560 27286948
P19_NANOG_CNCC_D5_mem_q10_srt_rmdup_peaks_peak_28815
chr7 27289980 27290414
P19_NANOG_CNCC_D5_mem_q10_srt_rmdup_peaks_peak_28816
chr7 27415674 27416060
P19_NANOG_CNCC_D5_mem_q10_srt_rmdup_peaks_peak_28820
chr7 27780285 27780556
P19_NANOG_CNCC_D5_mem_q10_srt_rmdup_peaks_peak_28827
chr7 28401140 28401617
P19_NANOG_CNCC_D5_mem_q10_srt_rmdup_peaks_peak_28839
chr7 28522745 28523021
P19_NANOG_CNCC_D5_mem_q10_srt_rmdup_peaks_peak_28840
chr7 28556036 28556589
P19_NANOG_CNCC_D5_mem_q10_srt_rmdup_peaks_peak_28841
chr7 28918804 28919159
P19_NANOG_CNCC_D5_mem_q10_srt_rmdup_peaks_peak_28845
chr7 28944004 28944443
P19_NANOG_CNCC_D5_mem_q10_srt_rmdup_peaks_peak_28849
chr7 28971235 28971506
P19_NANOG_CNCC_D5_mem_q10_srt_rmdup_peaks_peak_28850
chr7 29304818 29305383
P19_NANOG_CNCC_D5_mem_q10_srt_rmdup_peaks_peak_28855
chr7 30320826 30321228
P19_NANOG_CNCC_D5_mem_q10_srt_rmdup_peaks_peak_28871
chr7 31336821 31337092
P19_NANOG_CNCC_D5_mem_q10_srt_rmdup_peaks_peak_28890
chr7 31933172 31933574
P19_NANOG_CNCC_D5_mem_q10_srt_rmdup_peaks_peak_28900
chr7 32567596 32567898
P19_NANOG_CNCC_D5_mem_q10_srt_rmdup_peaks_peak_28904
chr7 32903621 32903953
P19_NANOG_CNCC_D5_mem_q10_srt_rmdup_peaks_peak_28910
chr7 33120025 33120333
P19_NANOG_CNCC_D5_mem_q10_srt_rmdup_peaks_peak_28914
chr7 33516143 33516480
P19_NANOG_CNCC_D5_mem_q10_srt_rmdup_peaks_peak_28918
chr7 33672341 33672847

P19_NANOG_CNCC_D5_mem_q10_srt_rmdup_peaks_peak_28922
chr7 34308623 34308985
P19_NANOG_CNCC_D5_mem_q10_srt_rmdup_peaks_peak_28934
chr7 34380926 34381316
P19_NANOG_CNCC_D5_mem_q10_srt_rmdup_peaks_peak_28936
chr7 34694914 34695270
P19_NANOG_CNCC_D5_mem_q10_srt_rmdup_peaks_peak_28940
chr7 36465827 36466098
P19_NANOG_CNCC_D5_mem_q10_srt_rmdup_peaks_peak_28961
chr7 37488526 37488807
P19_NANOG_CNCC_D5_mem_q10_srt_rmdup_peaks_peak_28977
chr7 39082144 39082673
P19_NANOG_CNCC_D5_mem_q10_srt_rmdup_peaks_peak_28987
chr7 39350329 39350752
P19_NANOG_CNCC_D5_mem_q10_srt_rmdup_peaks_peak_28993
chr7 39373406 39373733
P19_NANOG_CNCC_D5_mem_q10_srt_rmdup_peaks_peak_28994
chr7 39802528 39802898
P19_NANOG_CNCC_D5_mem_q10_srt_rmdup_peaks_peak_29001
chr7 39988932 39989297
P19_NANOG_CNCC_D5_mem_q10_srt_rmdup_peaks_peak_29002
chr7 40135274 40135755
P19_NANOG_CNCC_D5_mem_q10_srt_rmdup_peaks_peak_29004
chr7 40364268 40364635
P19_NANOG_CNCC_D5_mem_q10_srt_rmdup_peaks_peak_29007
chr7 40766271 40766621
P19_NANOG_CNCC_D5_mem_q10_srt_rmdup_peaks_peak_29010
chr7 41153319 41153612
P19_NANOG_CNCC_D5_mem_q10_srt_rmdup_peaks_peak_29014
chr7 41611450 41611877
P19_NANOG_CNCC_D5_mem_q10_srt_rmdup_peaks_peak_29018
chr7 41667671 41668113
P19_NANOG_CNCC_D5_mem_q10_srt_rmdup_peaks_peak_29020
chr7 41721961 41722730
P19_NANOG_CNCC_D5_mem_q10_srt_rmdup_peaks_peak_29022
chr7 42927833 42928108
P19_NANOG_CNCC_D5_mem_q10_srt_rmdup_peaks_peak_29042
chr7 44217228 44217518
P19_NANOG_CNCC_D5_mem_q10_srt_rmdup_peaks_peak_29058
chr7 44887505 44887847
P19_NANOG_CNCC_D5_mem_q10_srt_rmdup_peaks_peak_29066
chr7 44925222 44925512
P19_NANOG_CNCC_D5_mem_q10_srt_rmdup_peaks_peak_29067
chr7 46391992 46392318
P19_NANOG_CNCC_D5_mem_q10_srt_rmdup_peaks_peak_29076
chr7 46825854 46826262
P19_NANOG_CNCC_D5_mem_q10_srt_rmdup_peaks_peak_29081
chr7 47482626 47482930
P19_NANOG_CNCC_D5_mem_q10_srt_rmdup_peaks_peak_29084
chr7 48126374 48126763

P19_NANOG_CNCC_D5_mem_q10_srt_rmdup_peaks_peak_29099
chr7 48217657 48218067
P19_NANOG_CNCC_D5_mem_q10_srt_rmdup_peaks_peak_29102
chr7 48528843 48529143
P19_NANOG_CNCC_D5_mem_q10_srt_rmdup_peaks_peak_29106
chr7 48634048 48634357
P19_NANOG_CNCC_D5_mem_q10_srt_rmdup_peaks_peak_29107
chr7 48915000 48915321
P19_NANOG_CNCC_D5_mem_q10_srt_rmdup_peaks_peak_29109
chr7 49064030 49064382
P19_NANOG_CNCC_D5_mem_q10_srt_rmdup_peaks_peak_29111
chr7 50108520 50108808
P19_NANOG_CNCC_D5_mem_q10_srt_rmdup_peaks_peak_29117
chr7 50758170 50758931
P19_NANOG_CNCC_D5_mem_q10_srt_rmdup_peaks_peak_29120
chr7 50932306 50932777
P19_NANOG_CNCC_D5_mem_q10_srt_rmdup_peaks_peak_29123
chr7 52615092 52615449
P19_NANOG_CNCC_D5_mem_q10_srt_rmdup_peaks_peak_29145
chr7 54610884 54611161
P19_NANOG_CNCC_D5_mem_q10_srt_rmdup_peaks_peak_29158
chr7 54796129 54796414
P19_NANOG_CNCC_D5_mem_q10_srt_rmdup_peaks_peak_29162
chr7 65779878 65780460
P19_NANOG_CNCC_D5_mem_q10_srt_rmdup_peaks_peak_29185
chr7 66460373 66460722
P19_NANOG_CNCC_D5_mem_q10_srt_rmdup_peaks_peak_29191
chr7 69284184 69284620
P19_NANOG_CNCC_D5_mem_q10_srt_rmdup_peaks_peak_29220
chr7 69288435 69288753
P19_NANOG_CNCC_D5_mem_q10_srt_rmdup_peaks_peak_29221
chr7 69323465 69323856
P19_NANOG_CNCC_D5_mem_q10_srt_rmdup_peaks_peak_29223
chr7 69820666 69821087
P19_NANOG_CNCC_D5_mem_q10_srt_rmdup_peaks_peak_29230
chr7 70053926 70054281
P19_NANOG_CNCC_D5_mem_q10_srt_rmdup_peaks_peak_29236
chr7 70102791 70103082
P19_NANOG_CNCC_D5_mem_q10_srt_rmdup_peaks_peak_29240
chr7 70125771 70126292
P19_NANOG_CNCC_D5_mem_q10_srt_rmdup_peaks_peak_29241
chr7 71039136 71039436
P19_NANOG_CNCC_D5_mem_q10_srt_rmdup_peaks_peak_29260
chr7 71085909 71086180
P19_NANOG_CNCC_D5_mem_q10_srt_rmdup_peaks_peak_29261
chr7 71217085 71217356
P19_NANOG_CNCC_D5_mem_q10_srt_rmdup_peaks_peak_29266
chr7 71249110 71249397
P19_NANOG_CNCC_D5_mem_q10_srt_rmdup_peaks_peak_29268
chr7 72971378 72971713

P19_NANOG_CNCC_D5_mem_q10_srt_rmdup_peaks_peak_29278
chr7 73256029 73256451
P19_NANOG_CNCC_D5_mem_q10_srt_rmdup_peaks_peak_29288
chr7 75854678 75855066
P19_NANOG_CNCC_D5_mem_q10_srt_rmdup_peaks_peak_29315
chr7 75874157 75874567
P19_NANOG_CNCC_D5_mem_q10_srt_rmdup_peaks_peak_29317
chr7 75948106 75948439
P19_NANOG_CNCC_D5_mem_q10_srt_rmdup_peaks_peak_29322
chr7 76513496 76513870
P19_NANOG_CNCC_D5_mem_q10_srt_rmdup_peaks_peak_29327
chr7 78125564 78125877
P19_NANOG_CNCC_D5_mem_q10_srt_rmdup_peaks_peak_29342
chr7 78378759 78379043
P19_NANOG_CNCC_D5_mem_q10_srt_rmdup_peaks_peak_29347
chr7 81373612 81374223
P19_NANOG_CNCC_D5_mem_q10_srt_rmdup_peaks_peak_29371
chr7 81860516 81860823
P19_NANOG_CNCC_D5_mem_q10_srt_rmdup_peaks_peak_29379
chr7 82160897 82161281
P19_NANOG_CNCC_D5_mem_q10_srt_rmdup_peaks_peak_29388
chr7 82778093 82778532
P19_NANOG_CNCC_D5_mem_q10_srt_rmdup_peaks_peak_29398
chr7 83057064 83057576
P19_NANOG_CNCC_D5_mem_q10_srt_rmdup_peaks_peak_29402
chr7 83360621 83360892
P19_NANOG_CNCC_D5_mem_q10_srt_rmdup_peaks_peak_29410
chr7 83631780 83632100
P19_NANOG_CNCC_D5_mem_q10_srt_rmdup_peaks_peak_29413
chr7 83921057 83921546
P19_NANOG_CNCC_D5_mem_q10_srt_rmdup_peaks_peak_29423
chr7 88720946 88721217
P19_NANOG_CNCC_D5_mem_q10_srt_rmdup_peaks_peak_29450
chr7 89616950 89617221
P19_NANOG_CNCC_D5_mem_q10_srt_rmdup_peaks_peak_29452
chr7 90857920 90858359
P19_NANOG_CNCC_D5_mem_q10_srt_rmdup_peaks_peak_29464
chr7 91569904 91570263
P19_NANOG_CNCC_D5_mem_q10_srt_rmdup_peaks_peak_29477
chr7 92469656 92470004
P19_NANOG_CNCC_D5_mem_q10_srt_rmdup_peaks_peak_29495
chr7 92621482 92621780
P19_NANOG_CNCC_D5_mem_q10_srt_rmdup_peaks_peak_29497
chr7 93198422 93198749
P19_NANOG_CNCC_D5_mem_q10_srt_rmdup_peaks_peak_29503
chr7 93269198 93269514
P19_NANOG_CNCC_D5_mem_q10_srt_rmdup_peaks_peak_29505
chr7 93519766 93520037
P19_NANOG_CNCC_D5_mem_q10_srt_rmdup_peaks_peak_29508
chr7 93605611 93605957

P19_NANOG_CNCC_D5_mem_q10_srt_rmdup_peaks_peak_29509
chr7 94206672 94207220
P19_NANOG_CNCC_D5_mem_q10_srt_rmdup_peaks_peak_29520
chr7 94541494 94541817
P19_NANOG_CNCC_D5_mem_q10_srt_rmdup_peaks_peak_29525
chr7 94676125 94676514
P19_NANOG_CNCC_D5_mem_q10_srt_rmdup_peaks_peak_29527
chr7 94877425 94877709
P19_NANOG_CNCC_D5_mem_q10_srt_rmdup_peaks_peak_29529
chr7 95647025 95647418
P19_NANOG_CNCC_D5_mem_q10_srt_rmdup_peaks_peak_29532
chr7 96614691 96615361
P19_NANOG_CNCC_D5_mem_q10_srt_rmdup_peaks_peak_29548
chr7 97933151 97933435
P19_NANOG_CNCC_D5_mem_q10_srt_rmdup_peaks_peak_29574
chr7 97989793 97990064
P19_NANOG_CNCC_D5_mem_q10_srt_rmdup_peaks_peak_29576
chr7 98248744 98249138
P19_NANOG_CNCC_D5_mem_q10_srt_rmdup_peaks_peak_29578
chr7 99290977 99291383
P19_NANOG_CNCC_D5_mem_q10_srt_rmdup_peaks_peak_29588
chr7 99698114 99698487
P19_NANOG_CNCC_D5_mem_q10_srt_rmdup_peaks_peak_29593
chr7 100916465 100916736
P19_NANOG_CNCC_D5_mem_q10_srt_rmdup_peaks_peak_29629
chr7 101277513 101277905
P19_NANOG_CNCC_D5_mem_q10_srt_rmdup_peaks_peak_29634
chr7 101740940 101741481
P19_NANOG_CNCC_D5_mem_q10_srt_rmdup_peaks_peak_29643
chr7 102061372 102061660
P19_NANOG_CNCC_D5_mem_q10_srt_rmdup_peaks_peak_29647
chr7 102082319 102082597
P19_NANOG_CNCC_D5_mem_q10_srt_rmdup_peaks_peak_29648
chr7 102389144 102389753
P19_NANOG_CNCC_D5_mem_q10_srt_rmdup_peaks_peak_29650
chr7 102967892 102968189
P19_NANOG_CNCC_D5_mem_q10_srt_rmdup_peaks_peak_29658
chr7 104431551 104431889
P19_NANOG_CNCC_D5_mem_q10_srt_rmdup_peaks_peak_29663
chr7 104759528 104760068
P19_NANOG_CNCC_D5_mem_q10_srt_rmdup_peaks_peak_29668
chr7 104863914 104864193
P19_NANOG_CNCC_D5_mem_q10_srt_rmdup_peaks_peak_29670
chr7 105005135 105005517
P19_NANOG_CNCC_D5_mem_q10_srt_rmdup_peaks_peak_29671
chr7 105330937 105331344
P19_NANOG_CNCC_D5_mem_q10_srt_rmdup_peaks_peak_29678
chr7 105470275 105470547
P19_NANOG_CNCC_D5_mem_q10_srt_rmdup_peaks_peak_29684
chr7 106482493 106483101

P19_NANOG_CNCC_D5_mem_q10_srt_rmdup_peaks_peak_29698
chr7 106610787 106611412
P19_NANOG_CNCC_D5_mem_q10_srt_rmdup_peaks_peak_29701
chr7 106805918 106806347
P19_NANOG_CNCC_D5_mem_q10_srt_rmdup_peaks_peak_29703
chr7 107930909 107931574
P19_NANOG_CNCC_D5_mem_q10_srt_rmdup_peaks_peak_29714
chr7 109237494 109237806
P19_NANOG_CNCC_D5_mem_q10_srt_rmdup_peaks_peak_29725
chr7 111276785 111277087
P19_NANOG_CNCC_D5_mem_q10_srt_rmdup_peaks_peak_29742
chr7 113034938 113035213
P19_NANOG_CNCC_D5_mem_q10_srt_rmdup_peaks_peak_29760
chr7 113061716 113062094
P19_NANOG_CNCC_D5_mem_q10_srt_rmdup_peaks_peak_29761
chr7 114270114 114270965
P19_NANOG_CNCC_D5_mem_q10_srt_rmdup_peaks_peak_29766
chr7 114330731 114331135
P19_NANOG_CNCC_D5_mem_q10_srt_rmdup_peaks_peak_29767
chr7 114348748 114349102
P19_NANOG_CNCC_D5_mem_q10_srt_rmdup_peaks_peak_29768
chr7 115452781 115453169
P19_NANOG_CNCC_D5_mem_q10_srt_rmdup_peaks_peak_29783
chr7 115803080 115803438
P19_NANOG_CNCC_D5_mem_q10_srt_rmdup_peaks_peak_29786
chr7 116255614 116256013
P19_NANOG_CNCC_D5_mem_q10_srt_rmdup_peaks_peak_29790
chr7 117515666 117516094
P19_NANOG_CNCC_D5_mem_q10_srt_rmdup_peaks_peak_29805
chr7 117893141 117893517
P19_NANOG_CNCC_D5_mem_q10_srt_rmdup_peaks_peak_29808
chr7 120713989 120714353
P19_NANOG_CNCC_D5_mem_q10_srt_rmdup_peaks_peak_29830
chr7 121951307 121951634
P19_NANOG_CNCC_D5_mem_q10_srt_rmdup_peaks_peak_29848
chr7 121976891 121977442
P19_NANOG_CNCC_D5_mem_q10_srt_rmdup_peaks_peak_29850
chr7 122386848 122387488
P19_NANOG_CNCC_D5_mem_q10_srt_rmdup_peaks_peak_29864
chr7 122944636 122945095
P19_NANOG_CNCC_D5_mem_q10_srt_rmdup_peaks_peak_29871
chr7 124120152 124120516
P19_NANOG_CNCC_D5_mem_q10_srt_rmdup_peaks_peak_29883
chr7 124374374 124374645
P19_NANOG_CNCC_D5_mem_q10_srt_rmdup_peaks_peak_29886
chr7 125868343 125868754
P19_NANOG_CNCC_D5_mem_q10_srt_rmdup_peaks_peak_29899
chr7 126618667 126619018
P19_NANOG_CNCC_D5_mem_q10_srt_rmdup_peaks_peak_29903
chr7 127544326 127544876

P19_NANOG_CNCC_D5_mem_q10_srt_rmdup_peaks_peak_29914
chr7 128339321 128339712
P19_NANOG_CNCC_D5_mem_q10_srt_rmdup_peaks_peak_29927
chr7 128694935 128695243
P19_NANOG_CNCC_D5_mem_q10_srt_rmdup_peaks_peak_29934
chr7 129773899 129774410
P19_NANOG_CNCC_D5_mem_q10_srt_rmdup_peaks_peak_29961
chr7 129867861 129868193
P19_NANOG_CNCC_D5_mem_q10_srt_rmdup_peaks_peak_29962
chr7 129915069 129915433
P19_NANOG_CNCC_D5_mem_q10_srt_rmdup_peaks_peak_29966
chr7 131198311 131198670
P19_NANOG_CNCC_D5_mem_q10_srt_rmdup_peaks_peak_29979
chr7 131434672 131434983
P19_NANOG_CNCC_D5_mem_q10_srt_rmdup_peaks_peak_29989
chr7 131934604 131934950
P19_NANOG_CNCC_D5_mem_q10_srt_rmdup_peaks_peak_29999
chr7 134386124 134386407
P19_NANOG_CNCC_D5_mem_q10_srt_rmdup_peaks_peak_30022
chr7 134429390 134429733
P19_NANOG_CNCC_D5_mem_q10_srt_rmdup_peaks_peak_30023
chr7 134862259 134862608
P19_NANOG_CNCC_D5_mem_q10_srt_rmdup_peaks_peak_30026
chr7 135246215 135246618
P19_NANOG_CNCC_D5_mem_q10_srt_rmdup_peaks_peak_30028
chr7 135413070 135413365
P19_NANOG_CNCC_D5_mem_q10_srt_rmdup_peaks_peak_30030
chr7 135819331 135819739
P19_NANOG_CNCC_D5_mem_q10_srt_rmdup_peaks_peak_30034
chr7 136731749 136732020
P19_NANOG_CNCC_D5_mem_q10_srt_rmdup_peaks_peak_30041
chr7 137359609 137360076
P19_NANOG_CNCC_D5_mem_q10_srt_rmdup_peaks_peak_30047
chr7 138437645 138438022
P19_NANOG_CNCC_D5_mem_q10_srt_rmdup_peaks_peak_30071
chr7 138915446 138915717
P19_NANOG_CNCC_D5_mem_q10_srt_rmdup_peaks_peak_30075
chr7 139150395 139150716
P19_NANOG_CNCC_D5_mem_q10_srt_rmdup_peaks_peak_30079
chr7 139152255 139152865
P19_NANOG_CNCC_D5_mem_q10_srt_rmdup_peaks_peak_30080
chr7 139905139 139905771
P19_NANOG_CNCC_D5_mem_q10_srt_rmdup_peaks_peak_30089
chr7 140262443 140263056
P19_NANOG_CNCC_D5_mem_q10_srt_rmdup_peaks_peak_30099
chr7 140432224 140432495
P19_NANOG_CNCC_D5_mem_q10_srt_rmdup_peaks_peak_30103
chr7 140623971 140624412
P19_NANOG_CNCC_D5_mem_q10_srt_rmdup_peaks_peak_30105
chr7 141059115 141059495

P19_NANOG_CNCC_D5_mem_q10_srt_rmdup_peaks_peak_30109
chr7	142043738	142044009
P19_NANOG_CNCC_D5_mem_q10_srt_rmdup_peaks_peak_30120
chr7	143144705	143144998
P19_NANOG_CNCC_D5_mem_q10_srt_rmdup_peaks_peak_30132
chr7	143434149	143434527
P19_NANOG_CNCC_D5_mem_q10_srt_rmdup_peaks_peak_30134
chr7	144338595	144339050
P19_NANOG_CNCC_D5_mem_q10_srt_rmdup_peaks_peak_30142
chr7	144379634	144380017
P19_NANOG_CNCC_D5_mem_q10_srt_rmdup_peaks_peak_30143
chr7	144532788	144533081
P19_NANOG_CNCC_D5_mem_q10_srt_rmdup_peaks_peak_30145
chr7	146130000	146130288
P19_NANOG_CNCC_D5_mem_q10_srt_rmdup_peaks_peak_30151
chr7	147351545	147351821
P19_NANOG_CNCC_D5_mem_q10_srt_rmdup_peaks_peak_30162
chr7	147355735	147356015
P19_NANOG_CNCC_D5_mem_q10_srt_rmdup_peaks_peak_30163
chr7	147500710	147501114
P19_NANOG_CNCC_D5_mem_q10_srt_rmdup_peaks_peak_30166
chr7	148762787	148763301
P19_NANOG_CNCC_D5_mem_q10_srt_rmdup_peaks_peak_30194
chr7	148902655	148903040
P19_NANOG_CNCC_D5_mem_q10_srt_rmdup_peaks_peak_30196
chr7	149052418	149052807
P19_NANOG_CNCC_D5_mem_q10_srt_rmdup_peaks_peak_30198
chr7	149389830	149390160
P19_NANOG_CNCC_D5_mem_q10_srt_rmdup_peaks_peak_30201
chr7	149973169	149973764
P19_NANOG_CNCC_D5_mem_q10_srt_rmdup_peaks_peak_30208
chr7	150039739	150040072
P19_NANOG_CNCC_D5_mem_q10_srt_rmdup_peaks_peak_30213
chr7	150118094	150118485
P19_NANOG_CNCC_D5_mem_q10_srt_rmdup_peaks_peak_30220
chr7	150801394	150801734
P19_NANOG_CNCC_D5_mem_q10_srt_rmdup_peaks_peak_30229
chr7	151191428	151191852
P19_NANOG_CNCC_D5_mem_q10_srt_rmdup_peaks_peak_30243
chr7	151553163	151553587
P19_NANOG_CNCC_D5_mem_q10_srt_rmdup_peaks_peak_30247
chr7	155058810	155059179
P19_NANOG_CNCC_D5_mem_q10_srt_rmdup_peaks_peak_30260
chr7	156411913	156412263
P19_NANOG_CNCC_D5_mem_q10_srt_rmdup_peaks_peak_30278
chr7	157374765	157375129
P19_NANOG_CNCC_D5_mem_q10_srt_rmdup_peaks_peak_30292
chr7	157634430	157635106
P19_NANOG_CNCC_D5_mem_q10_srt_rmdup_peaks_peak_30293
chr7	157698199	157698606

P19_NANOG_CNCC_D5_mem_q10_srt_rmdup_peaks_peak_30294
chr7 157748010 157748811
P19_NANOG_CNCC_D5_mem_q10_srt_rmdup_peaks_peak_30295
chr7 157754498 157755278
P19_NANOG_CNCC_D5_mem_q10_srt_rmdup_peaks_peak_30296
chr7 157792859 157793218
P19_NANOG_CNCC_D5_mem_q10_srt_rmdup_peaks_peak_30297
chr7 157809099 157810217
P19_NANOG_CNCC_D5_mem_q10_srt_rmdup_peaks_peak_30298
chr7 157953870 157954192
P19_NANOG_CNCC_D5_mem_q10_srt_rmdup_peaks_peak_30299
chr7 158067348 158067934
P19_NANOG_CNCC_D5_mem_q10_srt_rmdup_peaks_peak_30300
chr7 158109983 158110921
P19_NANOG_CNCC_D5_mem_q10_srt_rmdup_peaks_peak_30301
chr7 158118545 158119056
P19_NANOG_CNCC_D5_mem_q10_srt_rmdup_peaks_peak_30302
chr8 521334 521952
P19_NANOG_CNCC_D5_mem_q10_srt_rmdup_peaks_peak_30327
chr8 850540 850964
P19_NANOG_CNCC_D5_mem_q10_srt_rmdup_peaks_peak_30332
chr8 1711087 1711358
P19_NANOG_CNCC_D5_mem_q10_srt_rmdup_peaks_peak_30343
chr8 1845155 1845483
P19_NANOG_CNCC_D5_mem_q10_srt_rmdup_peaks_peak_30348
chr8 5149883 5150349
P19_NANOG_CNCC_D5_mem_q10_srt_rmdup_peaks_peak_30375
chr8 5293936 5294384
P19_NANOG_CNCC_D5_mem_q10_srt_rmdup_peaks_peak_30376
chr8 6433686 6434005
P19_NANOG_CNCC_D5_mem_q10_srt_rmdup_peaks_peak_30382
chr8 7998289 7998571
P19_NANOG_CNCC_D5_mem_q10_srt_rmdup_peaks_peak_30389
chr8 8244576 8244906
P19_NANOG_CNCC_D5_mem_q10_srt_rmdup_peaks_peak_30393
chr8 8353837 8354239
P19_NANOG_CNCC_D5_mem_q10_srt_rmdup_peaks_peak_30395
chr8 8953506 8953807
P19_NANOG_CNCC_D5_mem_q10_srt_rmdup_peaks_peak_30413
chr8 9039078 9039394
P19_NANOG_CNCC_D5_mem_q10_srt_rmdup_peaks_peak_30416
chr8 9041110 9041411
P19_NANOG_CNCC_D5_mem_q10_srt_rmdup_peaks_peak_30417
chr8 9820430 9820741
P19_NANOG_CNCC_D5_mem_q10_srt_rmdup_peaks_peak_30427
chr8 10124418 10124998
P19_NANOG_CNCC_D5_mem_q10_srt_rmdup_peaks_peak_30432
chr8 10222689 10222971
P19_NANOG_CNCC_D5_mem_q10_srt_rmdup_peaks_peak_30437
chr8 11116264 11116600

P19_NANOG_CNCC_D5_mem_q10_srt_rmdup_peaks_peak_30452
chr8 11526203 11526592
P19_NANOG_CNCC_D5_mem_q10_srt_rmdup_peaks_peak_30456
chr8 12698165 12698524
P19_NANOG_CNCC_D5_mem_q10_srt_rmdup_peaks_peak_30465
chr8 12799451 12799820
P19_NANOG_CNCC_D5_mem_q10_srt_rmdup_peaks_peak_30469
chr8 13625887 13626204
P19_NANOG_CNCC_D5_mem_q10_srt_rmdup_peaks_peak_30477
chr8 13641371 13641762
P19_NANOG_CNCC_D5_mem_q10_srt_rmdup_peaks_peak_30478
chr8 15266245 15266773
P19_NANOG_CNCC_D5_mem_q10_srt_rmdup_peaks_peak_30487
chr8 15428159 15428436
P19_NANOG_CNCC_D5_mem_q10_srt_rmdup_peaks_peak_30488
chr8 16651935 16652213
P19_NANOG_CNCC_D5_mem_q10_srt_rmdup_peaks_peak_30498
chr8 18748212 18748774
P19_NANOG_CNCC_D5_mem_q10_srt_rmdup_peaks_peak_30514
chr8 21227529 21227896
P19_NANOG_CNCC_D5_mem_q10_srt_rmdup_peaks_peak_30555
chr8 22102448 22102742
P19_NANOG_CNCC_D5_mem_q10_srt_rmdup_peaks_peak_30566
chr8 22155888 22156303
P19_NANOG_CNCC_D5_mem_q10_srt_rmdup_peaks_peak_30567
chr8 22281978 22282348
P19_NANOG_CNCC_D5_mem_q10_srt_rmdup_peaks_peak_30569
chr8 22799337 22799717
P19_NANOG_CNCC_D5_mem_q10_srt_rmdup_peaks_peak_30578
chr8 22925360 22925791
P19_NANOG_CNCC_D5_mem_q10_srt_rmdup_peaks_peak_30582
chr8 23261299 23261865
P19_NANOG_CNCC_D5_mem_q10_srt_rmdup_peaks_peak_30586
chr8 23386248 23386546
P19_NANOG_CNCC_D5_mem_q10_srt_rmdup_peaks_peak_30589
chr8 24367069 24367431
P19_NANOG_CNCC_D5_mem_q10_srt_rmdup_peaks_peak_30607
chr8 26000774 26001085
P19_NANOG_CNCC_D5_mem_q10_srt_rmdup_peaks_peak_30635
chr8 26166875 26167295
P19_NANOG_CNCC_D5_mem_q10_srt_rmdup_peaks_peak_30638
chr8 26851258 26851532
P19_NANOG_CNCC_D5_mem_q10_srt_rmdup_peaks_peak_30649
chr8 28226746 28227017
P19_NANOG_CNCC_D5_mem_q10_srt_rmdup_peaks_peak_30661
chr8 28571008 28571530
P19_NANOG_CNCC_D5_mem_q10_srt_rmdup_peaks_peak_30666
chr8 28739768 28740052
P19_NANOG_CNCC_D5_mem_q10_srt_rmdup_peaks_peak_30669
chr8 29173194 29173475

P19_NANOG_CNCC_D5_mem_q10_srt_rmdup_peaks_peak_30674
chr8 29210595 29210878
P19_NANOG_CNCC_D5_mem_q10_srt_rmdup_peaks_peak_30677
chr8 29430534 29430896
P19_NANOG_CNCC_D5_mem_q10_srt_rmdup_peaks_peak_30681
chr8 29682814 29683181
P19_NANOG_CNCC_D5_mem_q10_srt_rmdup_peaks_peak_30685
chr8 29710695 29711102
P19_NANOG_CNCC_D5_mem_q10_srt_rmdup_peaks_peak_30687
chr8 29732368 29732802
P19_NANOG_CNCC_D5_mem_q10_srt_rmdup_peaks_peak_30688
chr8 30084817 30085210
P19_NANOG_CNCC_D5_mem_q10_srt_rmdup_peaks_peak_30693
chr8 30283348 30283640
P19_NANOG_CNCC_D5_mem_q10_srt_rmdup_peaks_peak_30699
chr8 30310579 30310850
P19_NANOG_CNCC_D5_mem_q10_srt_rmdup_peaks_peak_30703
chr8 31967933 31968276
P19_NANOG_CNCC_D5_mem_q10_srt_rmdup_peaks_peak_30713
chr8 32265843 32266134
P19_NANOG_CNCC_D5_mem_q10_srt_rmdup_peaks_peak_30715
chr8 32350511 32350932
P19_NANOG_CNCC_D5_mem_q10_srt_rmdup_peaks_peak_30718
chr8 32582713 32582984
P19_NANOG_CNCC_D5_mem_q10_srt_rmdup_peaks_peak_30721
chr8 32823812 32824084
P19_NANOG_CNCC_D5_mem_q10_srt_rmdup_peaks_peak_30723
chr8 33342342 33342649
P19_NANOG_CNCC_D5_mem_q10_srt_rmdup_peaks_peak_30730
chr8 36942436 36943041
P19_NANOG_CNCC_D5_mem_q10_srt_rmdup_peaks_peak_30773
chr8 37871856 37872303
P19_NANOG_CNCC_D5_mem_q10_srt_rmdup_peaks_peak_30783
chr8 38144341 38144677
P19_NANOG_CNCC_D5_mem_q10_srt_rmdup_peaks_peak_30787
chr8 38317622 38317935
P19_NANOG_CNCC_D5_mem_q10_srt_rmdup_peaks_peak_30794
chr8 38483809 38484228
P19_NANOG_CNCC_D5_mem_q10_srt_rmdup_peaks_peak_30804
chr8 38515449 38515929
P19_NANOG_CNCC_D5_mem_q10_srt_rmdup_peaks_peak_30805
chr8 38570214 38570485
P19_NANOG_CNCC_D5_mem_q10_srt_rmdup_peaks_peak_30806
chr8 39130489 39130761
P19_NANOG_CNCC_D5_mem_q10_srt_rmdup_peaks_peak_30811
chr8 40442324 40442907
P19_NANOG_CNCC_D5_mem_q10_srt_rmdup_peaks_peak_30823
chr8 40493962 40494507
P19_NANOG_CNCC_D5_mem_q10_srt_rmdup_peaks_peak_30824
chr8 40782392 40782686

P19_NANOG_CNCC_D5_mem_q10_srt_rmdup_peaks_peak_30829
chr8 41199823 41200116
P19_NANOG_CNCC_D5_mem_q10_srt_rmdup_peaks_peak_30843
chr8 41424989 41425434
P19_NANOG_CNCC_D5_mem_q10_srt_rmdup_peaks_peak_30848
chr8 41452625 41452896
P19_NANOG_CNCC_D5_mem_q10_srt_rmdup_peaks_peak_30849
chr8 41754674 41754945
P19_NANOG_CNCC_D5_mem_q10_srt_rmdup_peaks_peak_30854
chr8 42029034 42029352
P19_NANOG_CNCC_D5_mem_q10_srt_rmdup_peaks_peak_30860
chr8 42698440 42698744
P19_NANOG_CNCC_D5_mem_q10_srt_rmdup_peaks_peak_30863
chr8 49049012 49049391
P19_NANOG_CNCC_D5_mem_q10_srt_rmdup_peaks_peak_30872
chr8 49049627 49049960
P19_NANOG_CNCC_D5_mem_q10_srt_rmdup_peaks_peak_30873
chr8 49582345 49582679
P19_NANOG_CNCC_D5_mem_q10_srt_rmdup_peaks_peak_30881
chr8 49704787 49705118
P19_NANOG_CNCC_D5_mem_q10_srt_rmdup_peaks_peak_30885
chr8 50538140 50538602
P19_NANOG_CNCC_D5_mem_q10_srt_rmdup_peaks_peak_30888
chr8 51552806 51553197
P19_NANOG_CNCC_D5_mem_q10_srt_rmdup_peaks_peak_30897
chr8 53512294 53512592
P19_NANOG_CNCC_D5_mem_q10_srt_rmdup_peaks_peak_30908
chr8 53852353 53852830
P19_NANOG_CNCC_D5_mem_q10_srt_rmdup_peaks_peak_30913
chr8 54462371 54462643
P19_NANOG_CNCC_D5_mem_q10_srt_rmdup_peaks_peak_30919
chr8 54572362 54572692
P19_NANOG_CNCC_D5_mem_q10_srt_rmdup_peaks_peak_30922
chr8 54945007 54945405
P19_NANOG_CNCC_D5_mem_q10_srt_rmdup_peaks_peak_30929
chr8 55624117 55624388
P19_NANOG_CNCC_D5_mem_q10_srt_rmdup_peaks_peak_30937
chr8 57852821 57853349
P19_NANOG_CNCC_D5_mem_q10_srt_rmdup_peaks_peak_30965
chr8 58960568 58960965
P19_NANOG_CNCC_D5_mem_q10_srt_rmdup_peaks_peak_30974
chr8 59210220 59210623
P19_NANOG_CNCC_D5_mem_q10_srt_rmdup_peaks_peak_30978
chr8 59366250 59366598
P19_NANOG_CNCC_D5_mem_q10_srt_rmdup_peaks_peak_30979
chr8 59971394 59971721
P19_NANOG_CNCC_D5_mem_q10_srt_rmdup_peaks_peak_30987
chr8 60023119 60023390
P19_NANOG_CNCC_D5_mem_q10_srt_rmdup_peaks_peak_30992
chr8 61786330 61786716

P19_NANOG_CNCC_D5_mem_q10_srt_rmdup_peaks_peak_31005
chr8 62414170 62414521
P19_NANOG_CNCC_D5_mem_q10_srt_rmdup_peaks_peak_31011
chr8 62475813 62476092
P19_NANOG_CNCC_D5_mem_q10_srt_rmdup_peaks_peak_31012
chr8 63538565 63539018
P19_NANOG_CNCC_D5_mem_q10_srt_rmdup_peaks_peak_31024
chr8 63802741 63803089
P19_NANOG_CNCC_D5_mem_q10_srt_rmdup_peaks_peak_31027
chr8 64409566 64409837
P19_NANOG_CNCC_D5_mem_q10_srt_rmdup_peaks_peak_31033
chr8 67166954 67167273
P19_NANOG_CNCC_D5_mem_q10_srt_rmdup_peaks_peak_31072
chr8 67181811 67182082
P19_NANOG_CNCC_D5_mem_q10_srt_rmdup_peaks_peak_31073
chr8 67813476 67813751
P19_NANOG_CNCC_D5_mem_q10_srt_rmdup_peaks_peak_31082
chr8 68256111 68256382
P19_NANOG_CNCC_D5_mem_q10_srt_rmdup_peaks_peak_31086
chr8 68261220 68261491
P19_NANOG_CNCC_D5_mem_q10_srt_rmdup_peaks_peak_31087
chr8 68796617 68796904
P19_NANOG_CNCC_D5_mem_q10_srt_rmdup_peaks_peak_31095
chr8 69113855 69114168
P19_NANOG_CNCC_D5_mem_q10_srt_rmdup_peaks_peak_31099
chr8 69136588 69136859
P19_NANOG_CNCC_D5_mem_q10_srt_rmdup_peaks_peak_31100
chr8 69505083 69505408
P19_NANOG_CNCC_D5_mem_q10_srt_rmdup_peaks_peak_31103
chr8 70372673 70372974
P19_NANOG_CNCC_D5_mem_q10_srt_rmdup_peaks_peak_31113
chr8 70376519 70376868
P19_NANOG_CNCC_D5_mem_q10_srt_rmdup_peaks_peak_31114
chr8 71251238 71251530
P19_NANOG_CNCC_D5_mem_q10_srt_rmdup_peaks_peak_31130
chr8 71303828 71304101
P19_NANOG_CNCC_D5_mem_q10_srt_rmdup_peaks_peak_31132
chr8 73589403 73589786
P19_NANOG_CNCC_D5_mem_q10_srt_rmdup_peaks_peak_31160
chr8 73920843 73921177
P19_NANOG_CNCC_D5_mem_q10_srt_rmdup_peaks_peak_31165
chr8 75213373 75213713
P19_NANOG_CNCC_D5_mem_q10_srt_rmdup_peaks_peak_31179
chr8 76168595 76168903
P19_NANOG_CNCC_D5_mem_q10_srt_rmdup_peaks_peak_31190
chr8 80789349 80790061
P19_NANOG_CNCC_D5_mem_q10_srt_rmdup_peaks_peak_31215
chr8 81019410 81019690
P19_NANOG_CNCC_D5_mem_q10_srt_rmdup_peaks_peak_31222
chr8 81063894 81064296

P19_NANOG_CNCC_D5_mem_q10_srt_rmdup_peaks_peak_31226
chr8 81523851 81524160
P19_NANOG_CNCC_D5_mem_q10_srt_rmdup_peaks_peak_31236
chr8 82543084 82543471
P19_NANOG_CNCC_D5_mem_q10_srt_rmdup_peaks_peak_31244
chr8 82586504 82586853
P19_NANOG_CNCC_D5_mem_q10_srt_rmdup_peaks_peak_31245
chr8 84718405 84718820
P19_NANOG_CNCC_D5_mem_q10_srt_rmdup_peaks_peak_31252
chr8 85446106 85446383
P19_NANOG_CNCC_D5_mem_q10_srt_rmdup_peaks_peak_31255
chr8 85538447 85538750
P19_NANOG_CNCC_D5_mem_q10_srt_rmdup_peaks_peak_31256
chr8 86871124 86871477
P19_NANOG_CNCC_D5_mem_q10_srt_rmdup_peaks_peak_31264
chr8 87420520 87420934
P19_NANOG_CNCC_D5_mem_q10_srt_rmdup_peaks_peak_31270
chr8 87718533 87719089
P19_NANOG_CNCC_D5_mem_q10_srt_rmdup_peaks_peak_31274
chr8 88985517 88985855
P19_NANOG_CNCC_D5_mem_q10_srt_rmdup_peaks_peak_31285
chr8 89277938 89278414
P19_NANOG_CNCC_D5_mem_q10_srt_rmdup_peaks_peak_31291
chr8 89352303 89352743
P19_NANOG_CNCC_D5_mem_q10_srt_rmdup_peaks_peak_31292
chr8 90264090 90264379
P19_NANOG_CNCC_D5_mem_q10_srt_rmdup_peaks_peak_31301
chr8 90334402 90334837
P19_NANOG_CNCC_D5_mem_q10_srt_rmdup_peaks_peak_31302
chr8 93075695 93076168
P19_NANOG_CNCC_D5_mem_q10_srt_rmdup_peaks_peak_31332
chr8 93102288 93102569
P19_NANOG_CNCC_D5_mem_q10_srt_rmdup_peaks_peak_31334
chr8 93124982 93125292
P19_NANOG_CNCC_D5_mem_q10_srt_rmdup_peaks_peak_31337
chr8 93977666 93978159
P19_NANOG_CNCC_D5_mem_q10_srt_rmdup_peaks_peak_31347
chr8 94468280 94468608
P19_NANOG_CNCC_D5_mem_q10_srt_rmdup_peaks_peak_31353
chr8 95035024 95035436
P19_NANOG_CNCC_D5_mem_q10_srt_rmdup_peaks_peak_31361
chr8 95073096 95073802
P19_NANOG_CNCC_D5_mem_q10_srt_rmdup_peaks_peak_31362
chr8 95359117 95359388
P19_NANOG_CNCC_D5_mem_q10_srt_rmdup_peaks_peak_31365
chr8 95449524 95449807
P19_NANOG_CNCC_D5_mem_q10_srt_rmdup_peaks_peak_31369
chr8 95836119 95836481
P19_NANOG_CNCC_D5_mem_q10_srt_rmdup_peaks_peak_31373
chr8 95964567 95964871

P19_NANOG_CNCC_D5_mem_q10_srt_rmdup_peaks_peak_31376
chr8 96145539 96145826
P19_NANOG_CNCC_D5_mem_q10_srt_rmdup_peaks_peak_31378
chr8 97592291 97592614
P19_NANOG_CNCC_D5_mem_q10_srt_rmdup_peaks_peak_31393
chr8 98099161 98099472
P19_NANOG_CNCC_D5_mem_q10_srt_rmdup_peaks_peak_31396
chr8 98277466 98277815
P19_NANOG_CNCC_D5_mem_q10_srt_rmdup_peaks_peak_31399
chr8 100654837 100655119
P19_NANOG_CNCC_D5_mem_q10_srt_rmdup_peaks_peak_31420
chr8 101427956 101428365
P19_NANOG_CNCC_D5_mem_q10_srt_rmdup_peaks_peak_31427
chr8 101938853 101939134
P19_NANOG_CNCC_D5_mem_q10_srt_rmdup_peaks_peak_31438
chr8 102504280 102504577
P19_NANOG_CNCC_D5_mem_q10_srt_rmdup_peaks_peak_31448
chr8 102903981 102904295
P19_NANOG_CNCC_D5_mem_q10_srt_rmdup_peaks_peak_31459
chr8 104427289 104427715
P19_NANOG_CNCC_D5_mem_q10_srt_rmdup_peaks_peak_31487
chr8 104491715 104491992
P19_NANOG_CNCC_D5_mem_q10_srt_rmdup_peaks_peak_31488
chr8 107282375 107282768
P19_NANOG_CNCC_D5_mem_q10_srt_rmdup_peaks_peak_31519
chr8 107986691 107987048
P19_NANOG_CNCC_D5_mem_q10_srt_rmdup_peaks_peak_31521
chr8 108648000 108648355
P19_NANOG_CNCC_D5_mem_q10_srt_rmdup_peaks_peak_31528
chr8 110296005 110296400
P19_NANOG_CNCC_D5_mem_q10_srt_rmdup_peaks_peak_31541
chr8 113102869 113103142
P19_NANOG_CNCC_D5_mem_q10_srt_rmdup_peaks_peak_31557
chr8 113686458 113687116
P19_NANOG_CNCC_D5_mem_q10_srt_rmdup_peaks_peak_31562
chr8 114214527 114214835
P19_NANOG_CNCC_D5_mem_q10_srt_rmdup_peaks_peak_31571
chr8 116230931 116231315
P19_NANOG_CNCC_D5_mem_q10_srt_rmdup_peaks_peak_31580
chr8 116464723 116464999
P19_NANOG_CNCC_D5_mem_q10_srt_rmdup_peaks_peak_31581
chr8 116503279 116503670
P19_NANOG_CNCC_D5_mem_q10_srt_rmdup_peaks_peak_31583
chr8 116506170 116506482
P19_NANOG_CNCC_D5_mem_q10_srt_rmdup_peaks_peak_31584
chr8 116577262 116577552
P19_NANOG_CNCC_D5_mem_q10_srt_rmdup_peaks_peak_31585
chr8 116645191 116645639
P19_NANOG_CNCC_D5_mem_q10_srt_rmdup_peaks_peak_31586
chr8 116835758 116836371

P19_NANOG_CNCC_D5_mem_q10_srt_rmdup_peaks_peak_31589
chr8	117355973	117356244
P19_NANOG_CNCC_D5_mem_q10_srt_rmdup_peaks_peak_31594
chr8	117698238	117698577
P19_NANOG_CNCC_D5_mem_q10_srt_rmdup_peaks_peak_31596
chr8	118032016	118032395
P19_NANOG_CNCC_D5_mem_q10_srt_rmdup_peaks_peak_31600
chr8	118727549	118727929
P19_NANOG_CNCC_D5_mem_q10_srt_rmdup_peaks_peak_31605
chr8	118859934	118860285
P19_NANOG_CNCC_D5_mem_q10_srt_rmdup_peaks_peak_31607
chr8	119647410	119647705
P19_NANOG_CNCC_D5_mem_q10_srt_rmdup_peaks_peak_31624
chr8	119878548	119878915
P19_NANOG_CNCC_D5_mem_q10_srt_rmdup_peaks_peak_31626
chr8	120220392	120220669
P19_NANOG_CNCC_D5_mem_q10_srt_rmdup_peaks_peak_31629
chr8	120649236	120649618
P19_NANOG_CNCC_D5_mem_q10_srt_rmdup_peaks_peak_31633
chr8	120958148	120958566
P19_NANOG_CNCC_D5_mem_q10_srt_rmdup_peaks_peak_31638
chr8	121697625	121698038
P19_NANOG_CNCC_D5_mem_q10_srt_rmdup_peaks_peak_31646
chr8	121751422	121751709
P19_NANOG_CNCC_D5_mem_q10_srt_rmdup_peaks_peak_31647
chr8	121769270	121769733
P19_NANOG_CNCC_D5_mem_q10_srt_rmdup_peaks_peak_31648
chr8	122843160	122843451
P19_NANOG_CNCC_D5_mem_q10_srt_rmdup_peaks_peak_31657
chr8	124287237	124287672
P19_NANOG_CNCC_D5_mem_q10_srt_rmdup_peaks_peak_31677
chr8	124425113	124425505
P19_NANOG_CNCC_D5_mem_q10_srt_rmdup_peaks_peak_31679
chr8	126346294	126346824
P19_NANOG_CNCC_D5_mem_q10_srt_rmdup_peaks_peak_31700
chr8	127418539	127418841
P19_NANOG_CNCC_D5_mem_q10_srt_rmdup_peaks_peak_31708
chr8	127747965	127748531
P19_NANOG_CNCC_D5_mem_q10_srt_rmdup_peaks_peak_31718
chr8	128755142	128755583
P19_NANOG_CNCC_D5_mem_q10_srt_rmdup_peaks_peak_31729
chr8	129121335	129121645
P19_NANOG_CNCC_D5_mem_q10_srt_rmdup_peaks_peak_31745
chr8	129799929	129800302
P19_NANOG_CNCC_D5_mem_q10_srt_rmdup_peaks_peak_31754
chr8	130214754	130215034
P19_NANOG_CNCC_D5_mem_q10_srt_rmdup_peaks_peak_31761
chr8	130830364	130830838
P19_NANOG_CNCC_D5_mem_q10_srt_rmdup_peaks_peak_31770
chr8	130946328	130946673

P19_NANOG_CNCC_D5_mem_q10_srt_rmdup_peaks_peak_31773
chr8 130985138 130985631
P19_NANOG_CNCC_D5_mem_q10_srt_rmdup_peaks_peak_31774
chr8 131029166 131029512
P19_NANOG_CNCC_D5_mem_q10_srt_rmdup_peaks_peak_31776
chr8 131287254 131287953
P19_NANOG_CNCC_D5_mem_q10_srt_rmdup_peaks_peak_31780
chr8 131516113 131516429
P19_NANOG_CNCC_D5_mem_q10_srt_rmdup_peaks_peak_31785
chr8 132053745 132054022
P19_NANOG_CNCC_D5_mem_q10_srt_rmdup_peaks_peak_31789
chr8 132230852 132231148
P19_NANOG_CNCC_D5_mem_q10_srt_rmdup_peaks_peak_31791
chr8 132749206 132749645
P19_NANOG_CNCC_D5_mem_q10_srt_rmdup_peaks_peak_31795
chr8 136555600 136555966
P19_NANOG_CNCC_D5_mem_q10_srt_rmdup_peaks_peak_31819
chr8 136913145 136913685
P19_NANOG_CNCC_D5_mem_q10_srt_rmdup_peaks_peak_31822
chr8 141185408 141185705
P19_NANOG_CNCC_D5_mem_q10_srt_rmdup_peaks_peak_31835
chr8 141973524 141973795
P19_NANOG_CNCC_D5_mem_q10_srt_rmdup_peaks_peak_31844
chr8 142012659 142013167
P19_NANOG_CNCC_D5_mem_q10_srt_rmdup_peaks_peak_31845
chr8 142554531 142554951
P19_NANOG_CNCC_D5_mem_q10_srt_rmdup_peaks_peak_31847
chr8 143084114 143084417
P19_NANOG_CNCC_D5_mem_q10_srt_rmdup_peaks_peak_31853
chr8 143912661 143913051
P19_NANOG_CNCC_D5_mem_q10_srt_rmdup_peaks_peak_31859
chr8 144072997 144073364
P19_NANOG_CNCC_D5_mem_q10_srt_rmdup_peaks_peak_31860
chr8 145800740 145801030
P19_NANOG_CNCC_D5_mem_q10_srt_rmdup_peaks_peak_31879
chr8 145865073 145865419
P19_NANOG_CNCC_D5_mem_q10_srt_rmdup_peaks_peak_31880
chr8 146021261 146021650
P19_NANOG_CNCC_D5_mem_q10_srt_rmdup_peaks_peak_31883
chr9 442544 442885
P19_NANOG_CNCC_D5_mem_q10_srt_rmdup_peaks_peak_31891
chr9 557881 558214
P19_NANOG_CNCC_D5_mem_q10_srt_rmdup_peaks_peak_31895
chr9 720207 720501
P19_NANOG_CNCC_D5_mem_q10_srt_rmdup_peaks_peak_31899
chr9 1940189 1940491
P19_NANOG_CNCC_D5_mem_q10_srt_rmdup_peaks_peak_31915
chr9 2613579 2614313
P19_NANOG_CNCC_D5_mem_q10_srt_rmdup_peaks_peak_31933
chr9 2621293 2621610

P19_NANOG_CNCC_D5_mem_q10_srt_rmdup_peaks_peak_31934
chr9 2759333 2759688
P19_NANOG_CNCC_D5_mem_q10_srt_rmdup_peaks_peak_31937
chr9 2810953 2811290
P19_NANOG_CNCC_D5_mem_q10_srt_rmdup_peaks_peak_31940
chr9 3401592 3401961
P19_NANOG_CNCC_D5_mem_q10_srt_rmdup_peaks_peak_31946
chr9 3468347 3468926
P19_NANOG_CNCC_D5_mem_q10_srt_rmdup_peaks_peak_31947
chr9 3793991 3794278
P19_NANOG_CNCC_D5_mem_q10_srt_rmdup_peaks_peak_31949
chr9 4238010 4238387
P19_NANOG_CNCC_D5_mem_q10_srt_rmdup_peaks_peak_31954
chr9 4886827 4887236
P19_NANOG_CNCC_D5_mem_q10_srt_rmdup_peaks_peak_31963
chr9 4987824 4988117
P19_NANOG_CNCC_D5_mem_q10_srt_rmdup_peaks_peak_31964
chr9 5095913 5096233
P19_NANOG_CNCC_D5_mem_q10_srt_rmdup_peaks_peak_31965
chr9 5672008 5672407
P19_NANOG_CNCC_D5_mem_q10_srt_rmdup_peaks_peak_31970
chr9 6000807 6001345
P19_NANOG_CNCC_D5_mem_q10_srt_rmdup_peaks_peak_31972
chr9 6412581 6412953
P19_NANOG_CNCC_D5_mem_q10_srt_rmdup_peaks_peak_31976
chr9 7402217 7402652
P19_NANOG_CNCC_D5_mem_q10_srt_rmdup_peaks_peak_31989
chr9 7886520 7886794
P19_NANOG_CNCC_D5_mem_q10_srt_rmdup_peaks_peak_31994
chr9 8491999 8492286
P19_NANOG_CNCC_D5_mem_q10_srt_rmdup_peaks_peak_31999
chr9 8842812 8843267
P19_NANOG_CNCC_D5_mem_q10_srt_rmdup_peaks_peak_32010
chr9 8864028 8864437
P19_NANOG_CNCC_D5_mem_q10_srt_rmdup_peaks_peak_32012
chr9 9341143 9341464
P19_NANOG_CNCC_D5_mem_q10_srt_rmdup_peaks_peak_32016
chr9 12241536 12241931
P19_NANOG_CNCC_D5_mem_q10_srt_rmdup_peaks_peak_32032
chr9 12776258 12776618
P19_NANOG_CNCC_D5_mem_q10_srt_rmdup_peaks_peak_32035
chr9 13954505 13954806
P19_NANOG_CNCC_D5_mem_q10_srt_rmdup_peaks_peak_32044
chr9 14238442 14238870
P19_NANOG_CNCC_D5_mem_q10_srt_rmdup_peaks_peak_32046
chr9 14908952 14909263
P19_NANOG_CNCC_D5_mem_q10_srt_rmdup_peaks_peak_32061
chr9 16795850 16796180
P19_NANOG_CNCC_D5_mem_q10_srt_rmdup_peaks_peak_32089
chr9 18263352 18263735

P19_NANOG_CNCC_D5_mem_q10_srt_rmdup_peaks_peak_32098
chr9 19423103 19423504
P19_NANOG_CNCC_D5_mem_q10_srt_rmdup_peaks_peak_32111
chr9 19935385 19935676
P19_NANOG_CNCC_D5_mem_q10_srt_rmdup_peaks_peak_32120
chr9 20252834 20253234
P19_NANOG_CNCC_D5_mem_q10_srt_rmdup_peaks_peak_32123
chr9 20941170 20941449
P19_NANOG_CNCC_D5_mem_q10_srt_rmdup_peaks_peak_32133
chr9 21933378 21933686
P19_NANOG_CNCC_D5_mem_q10_srt_rmdup_peaks_peak_32143
chr9 22237571 22237892
P19_NANOG_CNCC_D5_mem_q10_srt_rmdup_peaks_peak_32153
chr9 22899083 22899378
P19_NANOG_CNCC_D5_mem_q10_srt_rmdup_peaks_peak_32161
chr9 23594407 23594781
P19_NANOG_CNCC_D5_mem_q10_srt_rmdup_peaks_peak_32168
chr9 25481387 25481768
P19_NANOG_CNCC_D5_mem_q10_srt_rmdup_peaks_peak_32182
chr9 26893205 26893751
P19_NANOG_CNCC_D5_mem_q10_srt_rmdup_peaks_peak_32187
chr9 28774448 28774875
P19_NANOG_CNCC_D5_mem_q10_srt_rmdup_peaks_peak_32205
chr9 28804303 28804594
P19_NANOG_CNCC_D5_mem_q10_srt_rmdup_peaks_peak_32207
chr9 28929143 28929463
P19_NANOG_CNCC_D5_mem_q10_srt_rmdup_peaks_peak_32209
chr9 29251327 29251670
P19_NANOG_CNCC_D5_mem_q10_srt_rmdup_peaks_peak_32213
chr9 33076784 33077074
P19_NANOG_CNCC_D5_mem_q10_srt_rmdup_peaks_peak_32233
chr9 33676597 33676869
P19_NANOG_CNCC_D5_mem_q10_srt_rmdup_peaks_peak_32237
chr9 34651999 34652270
P19_NANOG_CNCC_D5_mem_q10_srt_rmdup_peaks_peak_32248
chr9 34929556 34929865
P19_NANOG_CNCC_D5_mem_q10_srt_rmdup_peaks_peak_32249
chr9 35096198 35096588
P19_NANOG_CNCC_D5_mem_q10_srt_rmdup_peaks_peak_32254
chr9 35603913 35604234
P19_NANOG_CNCC_D5_mem_q10_srt_rmdup_peaks_peak_32262
chr9 35611200 35611615
P19_NANOG_CNCC_D5_mem_q10_srt_rmdup_peaks_peak_32263
chr9 36494704 36495126
P19_NANOG_CNCC_D5_mem_q10_srt_rmdup_peaks_peak_32275
chr9 37184644 37184940
P19_NANOG_CNCC_D5_mem_q10_srt_rmdup_peaks_peak_32286
chr9 38421532 38421838
P19_NANOG_CNCC_D5_mem_q10_srt_rmdup_peaks_peak_32314
chr9 40567846 40568291

P19_NANOG_CNCC_D5_mem_q10_srt_rmdup_peaks_peak_32318
chr9 41957553 41957824
P19_NANOG_CNCC_D5_mem_q10_srt_rmdup_peaks_peak_32319
chr9 71092674 71092956
P19_NANOG_CNCC_D5_mem_q10_srt_rmdup_peaks_peak_32327
chr9 71364577 71364897
P19_NANOG_CNCC_D5_mem_q10_srt_rmdup_peaks_peak_32336
chr9 71650604 71650890
P19_NANOG_CNCC_D5_mem_q10_srt_rmdup_peaks_peak_32342
chr9 72197228 72197499
P19_NANOG_CNCC_D5_mem_q10_srt_rmdup_peaks_peak_32352
chr9 73036286 73036596
P19_NANOG_CNCC_D5_mem_q10_srt_rmdup_peaks_peak_32361
chr9 74073963 74074234
P19_NANOG_CNCC_D5_mem_q10_srt_rmdup_peaks_peak_32373
chr9 74421791 74422179
P19_NANOG_CNCC_D5_mem_q10_srt_rmdup_peaks_peak_32380
chr9 76484167 76484532
P19_NANOG_CNCC_D5_mem_q10_srt_rmdup_peaks_peak_32403
chr9 76705856 76706247
P19_NANOG_CNCC_D5_mem_q10_srt_rmdup_peaks_peak_32406
chr9 76935793 76936152
P19_NANOG_CNCC_D5_mem_q10_srt_rmdup_peaks_peak_32411
chr9 77975159 77975491
P19_NANOG_CNCC_D5_mem_q10_srt_rmdup_peaks_peak_32417
chr9 78743734 78744021
P19_NANOG_CNCC_D5_mem_q10_srt_rmdup_peaks_peak_32423
chr9 78985406 78985766
P19_NANOG_CNCC_D5_mem_q10_srt_rmdup_peaks_peak_32425
chr9 79602481 79602843
P19_NANOG_CNCC_D5_mem_q10_srt_rmdup_peaks_peak_32437
chr9 80646600 80646922
P19_NANOG_CNCC_D5_mem_q10_srt_rmdup_peaks_peak_32455
chr9 81157718 81158163
P19_NANOG_CNCC_D5_mem_q10_srt_rmdup_peaks_peak_32463
chr9 81735418 81735777
P19_NANOG_CNCC_D5_mem_q10_srt_rmdup_peaks_peak_32466
chr9 82591302 82591657
P19_NANOG_CNCC_D5_mem_q10_srt_rmdup_peaks_peak_32483
chr9 83015815 83016233
P19_NANOG_CNCC_D5_mem_q10_srt_rmdup_peaks_peak_32485
chr9 83727799 83728111
P19_NANOG_CNCC_D5_mem_q10_srt_rmdup_peaks_peak_32493
chr9 84697496 84697815
P19_NANOG_CNCC_D5_mem_q10_srt_rmdup_peaks_peak_32507
chr9 86322481 86323153
P19_NANOG_CNCC_D5_mem_q10_srt_rmdup_peaks_peak_32519
chr9 86955195 86955529
P19_NANOG_CNCC_D5_mem_q10_srt_rmdup_peaks_peak_32527
chr9 87434294 87434659

P19_NANOG_CNCC_D5_mem_q10_srt_rmdup_peaks_peak_32536
chr9 88208009 88208296
P19_NANOG_CNCC_D5_mem_q10_srt_rmdup_peaks_peak_32541
chr9 88478815 88479092
P19_NANOG_CNCC_D5_mem_q10_srt_rmdup_peaks_peak_32542
chr9 88749261 88749587
P19_NANOG_CNCC_D5_mem_q10_srt_rmdup_peaks_peak_32545
chr9 88901888 88902276
P19_NANOG_CNCC_D5_mem_q10_srt_rmdup_peaks_peak_32549
chr9 89157317 89157644
P19_NANOG_CNCC_D5_mem_q10_srt_rmdup_peaks_peak_32556
chr9 89226142 89226678
P19_NANOG_CNCC_D5_mem_q10_srt_rmdup_peaks_peak_32558
chr9 89952590 89953057
P19_NANOG_CNCC_D5_mem_q10_srt_rmdup_peaks_peak_32569
chr9 90023067 90023406
P19_NANOG_CNCC_D5_mem_q10_srt_rmdup_peaks_peak_32572
chr9 90134474 90135020
P19_NANOG_CNCC_D5_mem_q10_srt_rmdup_peaks_peak_32574
chr9 90216035 90216362
P19_NANOG_CNCC_D5_mem_q10_srt_rmdup_peaks_peak_32577
chr9 90470414 90470748
P19_NANOG_CNCC_D5_mem_q10_srt_rmdup_peaks_peak_32580
chr9 91397804 91398510
P19_NANOG_CNCC_D5_mem_q10_srt_rmdup_peaks_peak_32597
chr9 92445140 92445476
P19_NANOG_CNCC_D5_mem_q10_srt_rmdup_peaks_peak_32611
chr9 93013602 93013931
P19_NANOG_CNCC_D5_mem_q10_srt_rmdup_peaks_peak_32616
chr9 93222411 93222906
P19_NANOG_CNCC_D5_mem_q10_srt_rmdup_peaks_peak_32620
chr9 93264218 93264558
P19_NANOG_CNCC_D5_mem_q10_srt_rmdup_peaks_peak_32621
chr9 93488603 93488993
P19_NANOG_CNCC_D5_mem_q10_srt_rmdup_peaks_peak_32622
chr9 94402217 94402488
P19_NANOG_CNCC_D5_mem_q10_srt_rmdup_peaks_peak_32636
chr9 94896249 94896704
P19_NANOG_CNCC_D5_mem_q10_srt_rmdup_peaks_peak_32644
chr9 94977054 94977325
P19_NANOG_CNCC_D5_mem_q10_srt_rmdup_peaks_peak_32645
chr9 95667608 95667879
P19_NANOG_CNCC_D5_mem_q10_srt_rmdup_peaks_peak_32652
chr9 96361899 96362353
P19_NANOG_CNCC_D5_mem_q10_srt_rmdup_peaks_peak_32659
chr9 96712797 96713185
P19_NANOG_CNCC_D5_mem_q10_srt_rmdup_peaks_peak_32662
chr9 98343967 98344307
P19_NANOG_CNCC_D5_mem_q10_srt_rmdup_peaks_peak_32688
chr9 99051653 99052020

P19_NANOG_CNCC_D5_mem_q10_srt_rmdup_peaks_peak_32700
chr9 99101940 99102260
P19_NANOG_CNCC_D5_mem_q10_srt_rmdup_peaks_peak_32702
chr9 99808436 99808871
P19_NANOG_CNCC_D5_mem_q10_srt_rmdup_peaks_peak_32712
chr9 100684676 100684971
P19_NANOG_CNCC_D5_mem_q10_srt_rmdup_peaks_peak_32732
chr9 100848210 100848630
P19_NANOG_CNCC_D5_mem_q10_srt_rmdup_peaks_peak_32736
chr9 101440694 101441005
P19_NANOG_CNCC_D5_mem_q10_srt_rmdup_peaks_peak_32743
chr9 101867024 101867408
P19_NANOG_CNCC_D5_mem_q10_srt_rmdup_peaks_peak_32750
chr9 102783584 102783863
P19_NANOG_CNCC_D5_mem_q10_srt_rmdup_peaks_peak_32762
chr9 103537176 103537475
P19_NANOG_CNCC_D5_mem_q10_srt_rmdup_peaks_peak_32767
chr9 103904646 103904976
P19_NANOG_CNCC_D5_mem_q10_srt_rmdup_peaks_peak_32772
chr9 103929589 103929951
P19_NANOG_CNCC_D5_mem_q10_srt_rmdup_peaks_peak_32774
chr9 103997583 103998109
P19_NANOG_CNCC_D5_mem_q10_srt_rmdup_peaks_peak_32775
chr9 104146148 104146488
P19_NANOG_CNCC_D5_mem_q10_srt_rmdup_peaks_peak_32776
chr9 105405065 105405382
P19_NANOG_CNCC_D5_mem_q10_srt_rmdup_peaks_peak_32783
chr9 106107814 106108122
P19_NANOG_CNCC_D5_mem_q10_srt_rmdup_peaks_peak_32786
chr9 106293175 106293651
P19_NANOG_CNCC_D5_mem_q10_srt_rmdup_peaks_peak_32787
chr9 106600169 106600704
P19_NANOG_CNCC_D5_mem_q10_srt_rmdup_peaks_peak_32788
chr9 106615453 106615816
P19_NANOG_CNCC_D5_mem_q10_srt_rmdup_peaks_peak_32789
chr9 107022390 107022707
P19_NANOG_CNCC_D5_mem_q10_srt_rmdup_peaks_peak_32791
chr9 107095835 107096148
P19_NANOG_CNCC_D5_mem_q10_srt_rmdup_peaks_peak_32792
chr9 107857889 107858194
P19_NANOG_CNCC_D5_mem_q10_srt_rmdup_peaks_peak_32803
chr9 107924611 107924884
P19_NANOG_CNCC_D5_mem_q10_srt_rmdup_peaks_peak_32805
chr9 110395545 110395956
P19_NANOG_CNCC_D5_mem_q10_srt_rmdup_peaks_peak_32836
chr9 110513498 110514163
P19_NANOG_CNCC_D5_mem_q10_srt_rmdup_peaks_peak_32838
chr9 110610269 110610557
P19_NANOG_CNCC_D5_mem_q10_srt_rmdup_peaks_peak_32840
chr9 110779953 110780238

P19_NANOG_CNCC_D5_mem_q10_srt_rmdup_peaks_peak_32842
chr9 111785269 111785575
P19_NANOG_CNCC_D5_mem_q10_srt_rmdup_peaks_peak_32850
chr9 111882113 111882496
P19_NANOG_CNCC_D5_mem_q10_srt_rmdup_peaks_peak_32851
chr9 114385417 114385699
P19_NANOG_CNCC_D5_mem_q10_srt_rmdup_peaks_peak_32874
chr9 114800119 114800392
P19_NANOG_CNCC_D5_mem_q10_srt_rmdup_peaks_peak_32879
chr9 114956221 114956639
P19_NANOG_CNCC_D5_mem_q10_srt_rmdup_peaks_peak_32884
chr9 115516861 115517138
P19_NANOG_CNCC_D5_mem_q10_srt_rmdup_peaks_peak_32891
chr9 115634229 115634545
P19_NANOG_CNCC_D5_mem_q10_srt_rmdup_peaks_peak_32893
chr9 116503213 116503611
P19_NANOG_CNCC_D5_mem_q10_srt_rmdup_peaks_peak_32901
chr9 117302013 117302388
P19_NANOG_CNCC_D5_mem_q10_srt_rmdup_peaks_peak_32910
chr9 117670404 117670699
P19_NANOG_CNCC_D5_mem_q10_srt_rmdup_peaks_peak_32915
chr9 117731124 117731521
P19_NANOG_CNCC_D5_mem_q10_srt_rmdup_peaks_peak_32917
chr9 117787357 117787822
P19_NANOG_CNCC_D5_mem_q10_srt_rmdup_peaks_peak_32918
chr9 117855931 117856202
P19_NANOG_CNCC_D5_mem_q10_srt_rmdup_peaks_peak_32920
chr9 117856337 117856618
P19_NANOG_CNCC_D5_mem_q10_srt_rmdup_peaks_peak_32921
chr9 118235318 118235666
P19_NANOG_CNCC_D5_mem_q10_srt_rmdup_peaks_peak_32924
chr9 118919119 118919442
P19_NANOG_CNCC_D5_mem_q10_srt_rmdup_peaks_peak_32928
chr9 119553764 119554056
P19_NANOG_CNCC_D5_mem_q10_srt_rmdup_peaks_peak_32942
chr9 119562288 119562660
P19_NANOG_CNCC_D5_mem_q10_srt_rmdup_peaks_peak_32943
chr9 119580474 119580862
P19_NANOG_CNCC_D5_mem_q10_srt_rmdup_peaks_peak_32945
chr9 119990445 119990966
P19_NANOG_CNCC_D5_mem_q10_srt_rmdup_peaks_peak_32950
chr9 120688351 120688668
P19_NANOG_CNCC_D5_mem_q10_srt_rmdup_peaks_peak_32961
chr9 121244586 121245005
P19_NANOG_CNCC_D5_mem_q10_srt_rmdup_peaks_peak_32965
chr9 121432094 121432818
P19_NANOG_CNCC_D5_mem_q10_srt_rmdup_peaks_peak_32967
chr9 121691026 121691369
P19_NANOG_CNCC_D5_mem_q10_srt_rmdup_peaks_peak_32968
chr9 121981884 121982298

P19_NANOG_CNCC_D5_mem_q10_srt_rmdup_peaks_peak_32975
chr9 122040429 122040849
P19_NANOG_CNCC_D5_mem_q10_srt_rmdup_peaks_peak_32976
chr9 123530492 123530939
P19_NANOG_CNCC_D5_mem_q10_srt_rmdup_peaks_peak_32996
chr9 124582053 124582480
P19_NANOG_CNCC_D5_mem_q10_srt_rmdup_peaks_peak_33010
chr9 124981378 124981906
P19_NANOG_CNCC_D5_mem_q10_srt_rmdup_peaks_peak_33018
chr9 124988553 124988846
P19_NANOG_CNCC_D5_mem_q10_srt_rmdup_peaks_peak_33020
chr9 126147717 126147988
P19_NANOG_CNCC_D5_mem_q10_srt_rmdup_peaks_peak_33029
chr9 126662482 126662852
P19_NANOG_CNCC_D5_mem_q10_srt_rmdup_peaks_peak_33043
chr9 127497387 127497677
P19_NANOG_CNCC_D5_mem_q10_srt_rmdup_peaks_peak_33062
chr9 127527738 127528134
P19_NANOG_CNCC_D5_mem_q10_srt_rmdup_peaks_peak_33065
chr9 128225333 128225720
P19_NANOG_CNCC_D5_mem_q10_srt_rmdup_peaks_peak_33080
chr9 129836927 129837198
P19_NANOG_CNCC_D5_mem_q10_srt_rmdup_peaks_peak_33122
chr9 130696593 130696864
P19_NANOG_CNCC_D5_mem_q10_srt_rmdup_peaks_peak_33138
chr9 131451070 131451418
P19_NANOG_CNCC_D5_mem_q10_srt_rmdup_peaks_peak_33155
chr9 132916037 132916340
P19_NANOG_CNCC_D5_mem_q10_srt_rmdup_peaks_peak_33180
chr9 133474906 133475364
P19_NANOG_CNCC_D5_mem_q10_srt_rmdup_peaks_peak_33190
chr9 134232765 134233436
P19_NANOG_CNCC_D5_mem_q10_srt_rmdup_peaks_peak_33206
chr9 134882301 134882753
P19_NANOG_CNCC_D5_mem_q10_srt_rmdup_peaks_peak_33225
chr9 135352685 135353025
P19_NANOG_CNCC_D5_mem_q10_srt_rmdup_peaks_peak_33233
chr9 135822176 135822476
P19_NANOG_CNCC_D5_mem_q10_srt_rmdup_peaks_peak_33245
chr9 136114472 136114802
P19_NANOG_CNCC_D5_mem_q10_srt_rmdup_peaks_peak_33248
chr9 137420972 137421302
P19_NANOG_CNCC_D5_mem_q10_srt_rmdup_peaks_peak_33254
chr9 138742571 138742900
P19_NANOG_CNCC_D5_mem_q10_srt_rmdup_peaks_peak_33267
chr9 139090599 139090910
P19_NANOG_CNCC_D5_mem_q10_srt_rmdup_peaks_peak_33274
chr9 139233716 139234094
P19_NANOG_CNCC_D5_mem_q10_srt_rmdup_peaks_peak_33275
chrUn_gl000220 41675 42134

P19_NANOG_CNCC_D5_mem_q10_srt_rmdup_peaks_peak_33297
chrUn_gl000220 94404 94786
P19_NANOG_CNCC_D5_mem_q10_srt_rmdup_peaks_peak_33299
chrUn_gl000220 96906 97249
P19_NANOG_CNCC_D5_mem_q10_srt_rmdup_peaks_peak_33300
chrUn_gl000220 131275 131815
P19_NANOG_CNCC_D5_mem_q10_srt_rmdup_peaks_peak_33309
chrUn_gl000220 132096 132706
P19_NANOG_CNCC_D5_mem_q10_srt_rmdup_peaks_peak_33310
chrUn_gl000220 140088 141089
P19_NANOG_CNCC_D5_mem_q10_srt_rmdup_peaks_peak_33313
chrUn_gl000220 141755 143239
P19_NANOG_CNCC_D5_mem_q10_srt_rmdup_peaks_peak_33314
chrUn_gl000220 144126 144562
P19_NANOG_CNCC_D5_mem_q10_srt_rmdup_peaks_peak_33315
chrUn_gl000220 145176 145530
P19_NANOG_CNCC_D5_mem_q10_srt_rmdup_peaks_peak_33316
chrX 5945098 5945482
P19_NANOG_CNCC_D5_mem_q10_srt_rmdup_peaks_peak_33355
chrX 6225073 6225706
P19_NANOG_CNCC_D5_mem_q10_srt_rmdup_peaks_peak_33358
chrX 6301665 6302340
P19_NANOG_CNCC_D5_mem_q10_srt_rmdup_peaks_peak_33359
chrX 6611073 6611404
P19_NANOG_CNCC_D5_mem_q10_srt_rmdup_peaks_peak_33360
chrX 7050536 7050969
P19_NANOG_CNCC_D5_mem_q10_srt_rmdup_peaks_peak_33366
chrX 8073112 8073427
P19_NANOG_CNCC_D5_mem_q10_srt_rmdup_peaks_peak_33372
chrX 13954866 13955226
P19_NANOG_CNCC_D5_mem_q10_srt_rmdup_peaks_peak_33424
chrX 16663717 16664119
P19_NANOG_CNCC_D5_mem_q10_srt_rmdup_peaks_peak_33457
chrX 17404005 17404355
P19_NANOG_CNCC_D5_mem_q10_srt_rmdup_peaks_peak_33463
chrX 18445895 18446370
P19_NANOG_CNCC_D5_mem_q10_srt_rmdup_peaks_peak_33473
chrX 25296430 25296845
P19_NANOG_CNCC_D5_mem_q10_srt_rmdup_peaks_peak_33527
chrX 26394381 26394798
P19_NANOG_CNCC_D5_mem_q10_srt_rmdup_peaks_peak_33530
chrX 29793346 29793736
P19_NANOG_CNCC_D5_mem_q10_srt_rmdup_peaks_peak_33553
chrX 33147793 33148064
P19_NANOG_CNCC_D5_mem_q10_srt_rmdup_peaks_peak_33574
chrX 40002139 40002532
P19_NANOG_CNCC_D5_mem_q10_srt_rmdup_peaks_peak_33618
chrX 40280086 40280507
P19_NANOG_CNCC_D5_mem_q10_srt_rmdup_peaks_peak_33625
chrX 40846666 40846954

P19_NANOG_CNCC_D5_mem_q10_srt_rmdup_peaks_peak_33634
chrX 41376156 41376443
P19_NANOG_CNCC_D5_mem_q10_srt_rmdup_peaks_peak_33646
chrX 41446892 41447209
P19_NANOG_CNCC_D5_mem_q10_srt_rmdup_peaks_peak_33647
chrX 42808033 42808382
P19_NANOG_CNCC_D5_mem_q10_srt_rmdup_peaks_peak_33660
chrX 47243936 47244295
P19_NANOG_CNCC_D5_mem_q10_srt_rmdup_peaks_peak_33699
chrX 49166792 49167193
P19_NANOG_CNCC_D5_mem_q10_srt_rmdup_peaks_peak_33709
chrX 50705146 50705486
P19_NANOG_CNCC_D5_mem_q10_srt_rmdup_peaks_peak_33724
chrX 50846583 50846877
P19_NANOG_CNCC_D5_mem_q10_srt_rmdup_peaks_peak_33725
chrX 53188444 53188780
P19_NANOG_CNCC_D5_mem_q10_srt_rmdup_peaks_peak_33737
chrX 53886292 53886655
P19_NANOG_CNCC_D5_mem_q10_srt_rmdup_peaks_peak_33742
chrX 55899082 55899486
P19_NANOG_CNCC_D5_mem_q10_srt_rmdup_peaks_peak_33747
chrX 57163648 57164217
P19_NANOG_CNCC_D5_mem_q10_srt_rmdup_peaks_peak_33750
chrX 63401946 63402419
P19_NANOG_CNCC_D5_mem_q10_srt_rmdup_peaks_peak_33763
chrX 64486489 64486811
P19_NANOG_CNCC_D5_mem_q10_srt_rmdup_peaks_peak_33768
chrX 67203394 67203902
P19_NANOG_CNCC_D5_mem_q10_srt_rmdup_peaks_peak_33775
chrX 68647359 68647695
P19_NANOG_CNCC_D5_mem_q10_srt_rmdup_peaks_peak_33795
chrX 68721386 68721657
P19_NANOG_CNCC_D5_mem_q10_srt_rmdup_peaks_peak_33797
chrX 69353314 69353682
P19_NANOG_CNCC_D5_mem_q10_srt_rmdup_peaks_peak_33802
chrX 69494545 69494840
P19_NANOG_CNCC_D5_mem_q10_srt_rmdup_peaks_peak_33805
chrX 70411118 70411448
P19_NANOG_CNCC_D5_mem_q10_srt_rmdup_peaks_peak_33815
chrX 71613469 71613750
P19_NANOG_CNCC_D5_mem_q10_srt_rmdup_peaks_peak_33829
chrX 73067703 73068149
P19_NANOG_CNCC_D5_mem_q10_srt_rmdup_peaks_peak_33841
chrX 74813857 74814166
P19_NANOG_CNCC_D5_mem_q10_srt_rmdup_peaks_peak_33862
chrX 76818225 76818496
P19_NANOG_CNCC_D5_mem_q10_srt_rmdup_peaks_peak_33870
chrX 78856887 78857227
P19_NANOG_CNCC_D5_mem_q10_srt_rmdup_peaks_peak_33877
chrX 79378189 79378717

P19_NANOG_CNCC_D5_mem_q10_srt_rmdup_peaks_peak_33880
chrX 80071526 80071883
P19_NANOG_CNCC_D5_mem_q10_srt_rmdup_peaks_peak_33882
chrX 80518415 80518715
P19_NANOG_CNCC_D5_mem_q10_srt_rmdup_peaks_peak_33883
chrX 80783436 80783873
P19_NANOG_CNCC_D5_mem_q10_srt_rmdup_peaks_peak_33884
chrX 80797688 80798311
P19_NANOG_CNCC_D5_mem_q10_srt_rmdup_peaks_peak_33885
chrX 80820290 80820743
P19_NANOG_CNCC_D5_mem_q10_srt_rmdup_peaks_peak_33886
chrX 81668293 81668564
P19_NANOG_CNCC_D5_mem_q10_srt_rmdup_peaks_peak_33887
chrX 84884724 84885180
P19_NANOG_CNCC_D5_mem_q10_srt_rmdup_peaks_peak_33901
chrX 88047224 88047594
P19_NANOG_CNCC_D5_mem_q10_srt_rmdup_peaks_peak_33912
chrX 92269709 92270034
P19_NANOG_CNCC_D5_mem_q10_srt_rmdup_peaks_peak_33931
chrX 92356596 92357024
P19_NANOG_CNCC_D5_mem_q10_srt_rmdup_peaks_peak_33933
chrX 93076614 93077009
P19_NANOG_CNCC_D5_mem_q10_srt_rmdup_peaks_peak_33938
chrX 93445201 93445810
P19_NANOG_CNCC_D5_mem_q10_srt_rmdup_peaks_peak_33941
chrX 93752962 93753361
P19_NANOG_CNCC_D5_mem_q10_srt_rmdup_peaks_peak_33948
chrX 93959415 93960233
P19_NANOG_CNCC_D5_mem_q10_srt_rmdup_peaks_peak_33951
chrX 93961119 93961411
P19_NANOG_CNCC_D5_mem_q10_srt_rmdup_peaks_peak_33952
chrX 94013022 94013698
P19_NANOG_CNCC_D5_mem_q10_srt_rmdup_peaks_peak_33953
chrX 94930024 94930343
P19_NANOG_CNCC_D5_mem_q10_srt_rmdup_peaks_peak_33958
chrX 96849520 96849949
P19_NANOG_CNCC_D5_mem_q10_srt_rmdup_peaks_peak_33982
chrX 97778503 97778913
P19_NANOG_CNCC_D5_mem_q10_srt_rmdup_peaks_peak_33998
chrX 97973049 97973333
P19_NANOG_CNCC_D5_mem_q10_srt_rmdup_peaks_peak_34000
chrX 98157254 98157759
P19_NANOG_CNCC_D5_mem_q10_srt_rmdup_peaks_peak_34001
chrX 98340152 98340598
P19_NANOG_CNCC_D5_mem_q10_srt_rmdup_peaks_peak_34004
chrX 98991872 98992403
P19_NANOG_CNCC_D5_mem_q10_srt_rmdup_peaks_peak_34007
chrX 99658324 99658811
P19_NANOG_CNCC_D5_mem_q10_srt_rmdup_peaks_peak_34012
chrX 99723325 99723619

P19_NANOG_CNCC_D5_mem_q10_srt_rmdup_peaks_peak_34015
chrX 100203473 100203789
P19_NANOG_CNCC_D5_mem_q10_srt_rmdup_peaks_peak_34022
chrX 100940972 100941272
P19_NANOG_CNCC_D5_mem_q10_srt_rmdup_peaks_peak_34025
chrX 102941758 102942029
P19_NANOG_CNCC_D5_mem_q10_srt_rmdup_peaks_peak_34035
chrX 104879894 104880249
P19_NANOG_CNCC_D5_mem_q10_srt_rmdup_peaks_peak_34050
chrX 105297883 105298309
P19_NANOG_CNCC_D5_mem_q10_srt_rmdup_peaks_peak_34051
chrX 106785871 106786163
P19_NANOG_CNCC_D5_mem_q10_srt_rmdup_peaks_peak_34057
chrX 108363970 108364384
P19_NANOG_CNCC_D5_mem_q10_srt_rmdup_peaks_peak_34074
chrX 108975276 108975608
P19_NANOG_CNCC_D5_mem_q10_srt_rmdup_peaks_peak_34083
chrX 109763377 109763776
P19_NANOG_CNCC_D5_mem_q10_srt_rmdup_peaks_peak_34088
chrX 110562827 110563239
P19_NANOG_CNCC_D5_mem_q10_srt_rmdup_peaks_peak_34095
chrX 110565738 110566073
P19_NANOG_CNCC_D5_mem_q10_srt_rmdup_peaks_peak_34096
chrX 110708912 110709197
P19_NANOG_CNCC_D5_mem_q10_srt_rmdup_peaks_peak_34099
chrX 110760265 110760640
P19_NANOG_CNCC_D5_mem_q10_srt_rmdup_peaks_peak_34100
chrX 110792436 110792800
P19_NANOG_CNCC_D5_mem_q10_srt_rmdup_peaks_peak_34103
chrX 112180506 112180777
P19_NANOG_CNCC_D5_mem_q10_srt_rmdup_peaks_peak_34124
chrX 112715447 112715777
P19_NANOG_CNCC_D5_mem_q10_srt_rmdup_peaks_peak_34129
chrX 112851239 112851541
P19_NANOG_CNCC_D5_mem_q10_srt_rmdup_peaks_peak_34134
chrX 113283102 113283602
P19_NANOG_CNCC_D5_mem_q10_srt_rmdup_peaks_peak_34145
chrX 114782919 114783358
P19_NANOG_CNCC_D5_mem_q10_srt_rmdup_peaks_peak_34161
chrX 115029850 115030213
P19_NANOG_CNCC_D5_mem_q10_srt_rmdup_peaks_peak_34172
chrX 122397113 122397384
P19_NANOG_CNCC_D5_mem_q10_srt_rmdup_peaks_peak_34199
chrX 124265778 124266366
P19_NANOG_CNCC_D5_mem_q10_srt_rmdup_peaks_peak_34212
chrX 127300238 127300755
P19_NANOG_CNCC_D5_mem_q10_srt_rmdup_peaks_peak_34224
chrX 128250717 128251007
P19_NANOG_CNCC_D5_mem_q10_srt_rmdup_peaks_peak_34228
chrX 134555686 134555971

P19_NANOG_CNCC_D5_mem_q10_srt_rmdup_peaks_peak_34306
chrX 134701151 134701493
P19_NANOG_CNCC_D5_mem_q10_srt_rmdup_peaks_peak_34310
chrX 135127192 135127467
P19_NANOG_CNCC_D5_mem_q10_srt_rmdup_peaks_peak_34311
chrX 135281564 135281980
P19_NANOG_CNCC_D5_mem_q10_srt_rmdup_peaks_peak_34314
chrX 136640436 136640707
P19_NANOG_CNCC_D5_mem_q10_srt_rmdup_peaks_peak_34322
chrX 136934799 136935276
P19_NANOG_CNCC_D5_mem_q10_srt_rmdup_peaks_peak_34330
chrX 137197029 137197672
P19_NANOG_CNCC_D5_mem_q10_srt_rmdup_peaks_peak_34333
chrX 138936161 138936508
P19_NANOG_CNCC_D5_mem_q10_srt_rmdup_peaks_peak_34347
chrX 146981875 146982237
P19_NANOG_CNCC_D5_mem_q10_srt_rmdup_peaks_peak_34365
chrX 147174492 147174808
P19_NANOG_CNCC_D5_mem_q10_srt_rmdup_peaks_peak_34366
chrX 147946767 147947210
P19_NANOG_CNCC_D5_mem_q10_srt_rmdup_peaks_peak_34376
chrX 150971903 150972232
P19_NANOG_CNCC_D5_mem_q10_srt_rmdup_peaks_peak_34405
chrX 150999292 150999678
P19_NANOG_CNCC_D5_mem_q10_srt_rmdup_peaks_peak_34407
chrX 152070403 152070738
P19_NANOG_CNCC_D5_mem_q10_srt_rmdup_peaks_peak_34410
chrX 153000852 153001185
P19_NANOG_CNCC_D5_mem_q10_srt_rmdup_peaks_peak_34415
chrX 154122062 154122405
P19_NANOG_CNCC_D5_mem_q10_srt_rmdup_peaks_peak_34431
