# Supplementary material for: Inability to switch from ARID1A-BAF to ARID1B-BAF impairs exit from pluripotency and commitment towards neural crest formation in ARID1B-related neurodevelopmental disorders
Source: Nat Commun. 2021 Nov 9;12:6469. doi: 10.1038/s41467-021-26810-x (PMC8578637; doi:10.1038/s41467-021-26810-x)
Supplement: Supplementary file 10 — Supplementary Data 7 [file 41467_2021_26810_MOESM10_ESM.pdf]

Supplementary Data 7 -LIST OF PRIMERS USED IN THIS STUDY			
**TARGET**	**PRIMER NAME**	**SEQUENCE**	**APPLICATION**
18s RNA	18S forward	5’-ATACATGCCGACGGGCGCTG-3’	qRT-PCR
	18S reverse	5’-AGGGGCTGACCGGGTTGGTT-3’	qRT-PCR
SOX2	SOX2 forward	5’-GCCGAGTGGAAACTTTTGTCG-3’	qRT-PCR
	SOX2 reverse	5’-GCAGCGTGTACTTATCCTTCTT-3’	qRT-PCR
OCT4	OCT4 forward	5’-TCGAGAACCGAGTGAGAGG-3’	qRT-PCR
	OCT4 reverse	5’-GAACCACACTCGGACCACA-3’	qRT-PCR
NANOG	NANOG forward	5’-ATGCCTCACACGGAGACTGT-3’	qRT-PCR
	NANOG reverse	5’-AAGTGGGTTGTTTGCCTTTG-3’	qRT-PCR
TFAP2A	TFAP2A forward	5’-AACATGCTCCTGGCTACAAAA-3’	qRT-PCR
	TFAP2A reverse	5’-AGGGGAGATCGGTCCGA-3’	qRT-PCR
NR2F1	NR2F1 forward	5’-ATCGTGCTGTTCACGTCAGA-3’	qRT-PCR
	NR2F1 reverse	5’-GCTCCTCACGTACTCCTCCA-3’	qRT-PCR
SOX9	SOX9 forward	5’-GTACCCGCACTTGCACAAC-3’	qRT-PCR
	SOX9 reverse	5’-TCTCGCTCTCGTTCAGAAGTC-3’	qRT-PCR
chr1:9919431-9919620	Chr1 forward	5’-TGGGAAGGACACAGTGACAA-3’	ChIP-qPCR
	Chr1 reverse	5’-CCTGGGTAAGGAGCTGGCTA-3’	ChIP-qPCR
chr2:1484741-1484873	Chr2 forward	5’-ACTTCTGCTGTGGGCATGTT-3’	ChIP-qPCR
	Chr2 reverse	5’-TGGGATCTCAAGCCAGCAAG-3’	ChIP-qPCR
chr17:853129-853213	Chr17 forward	5’-CCTGGGCACCTTCTGGTAAC-3’	ChIP-qPCR
	Chr17 reverse	5’-GGACCTGGGGCTAATTGCTT-3’	ChIP-qPCR
chr18:37993305-37993425	Chr18 forward	5’-TTCAAGCAACAAGGCACAGG-3’	ChIP-qPCR
	Chr18 reverse	5’-TTAGCTGCTGTGTGGCGTTA-3’	ChIP-qPCR
