# Supplementary material for: Inability to switch from ARID1A-BAF to ARID1B-BAF impairs exit from pluripotency and commitment towards neural crest formation in ARID1B-related neurodevelopmental disorders
Source: Nat Commun. 2021 Nov 9;12:6469. doi: 10.1038/s41467-021-26810-x (PMC8578637; doi:10.1038/s41467-021-26810-x)
Supplement: Supplementary file 11 — Reporting Summary [file 41467_2021_26810_MOESM11_ESM.pdf]

nature research

Corresponding author(s): MARCO TRIZZINO

Last updated by author(s): Oct 8, 2021

Reporting Summary

Nature Research wishes to improve the reproducibility of the work that we publish. This form provides structure for consistency and transparency in reporting. For further information on Nature Research policies, see our Editorial Policies and the Editorial Policy Checklist.

Statistics

For all statistical analyses, confirm that the following items are present in the figure legend, table legend, main text, or Methods section.

n/a	Confirmed	
☐	☒	The exact sample size (*n*) for each experimental group/condition, given as a discrete number and unit of measurement
☐	☒	A statement on whether measurements were taken from distinct samples or whether the same sample was measured repeatedly
☐	☒	The statistical test(s) used AND whether they are one- or two-sided<br>*Only common tests should be described solely by name; describe more complex techniques in the Methods section.*
☒	☐	A description of all covariates tested
☐	☒	A description of any assumptions or corrections, such as tests of normality and adjustment for multiple comparisons
☐	☒	A full description of the statistical parameters including central tendency (e.g. means) or other basic estimates (e.g. regression coefficient) AND variation (e.g. standard deviation) or associated estimates of uncertainty (e.g. confidence intervals)
☐	☒	For null hypothesis testing, the test statistic (e.g. *F*, *t*, *r*) with confidence intervals, effect sizes, degrees of freedom and *P* value noted<br>*Give P values as exact values whenever suitable.*
☒	☐	For Bayesian analysis, information on the choice of priors and Markov chain Monte Carlo settings
☒	☐	For hierarchical and complex designs, identification of the appropriate level for tests and full reporting of outcomes
☒	☐	Estimates of effect sizes (e.g. Cohen's *d*, Pearson's *r*), indicating how they were calculated

Our web collection on statistics for biologists contains articles on many of the points above.

Software and code

Policy information about availability of computer code

Data collection	No software used for data collection
Data analysis	- Adapter Trimming: TrimGalore! v0.6.5<br>- Read mapping (ATAC-seq and CHIP-seq): BWA mem v.0.7.17<br>- Peak calling (ATAC-seq and HISTONE CHIP-seq): Macs2 v2.2.71<br>- Peak calling (other chip-seq): Homer v4<br>- Gene expression quantification: Kallisto v0.46<br>- Differential Gene Expression: DeSeq2 package<br>- Pathway Analysis: Ingenuity Pathway Analysis suite<br>- Motif Analysis: Meme-ChIP (Meme Suite)<br>- Heatmaps and Average Profiles: Deeptools v3.5.0<br>- Flowcytometry: FlowJo v10.7<br>- Bam file filtering for Q=10 (uniquely mapping reads), sorting, and removal of PCR duplicates: Samtools v1.10

For manuscripts utilizing custom algorithms or software that are central to the research but not yet described in published literature, software must be made available to editors and reviewers. We strongly encourage code deposition in a community repository (e.g. GitHub). See the Nature Research guidelines for submitting code & software for further information.

Data

Policy information about availability of data

All manuscripts must include a data availability statement. This statement should provide the following information, where applicable:

- Accession codes, unique identifiers, or web links for publicly available datasets
- A list of figures that have associated raw data
- A description of any restrictions on data availability

All the Genomic Data (raw and processed) are available on GEO database (accession number GSE169654)

Field-specific reporting

Please select the one below that is the best fit for your research. If you are not sure, read the appropriate sections before making your selection.

☒ Life sciences ☐ Behavioural & social sciences ☐ Ecological, evolutionary & environmental sciences

For a reference copy of the document with all sections, see nature.com/documents/nr-reporting-summary-flat.pdf

Life sciences study design

All studies must disclose on these points even when the disclosure is negative.

Sample size	2 biological replicates per condition (2 Coffin-Siris patient iPSC lines vs 2 control lines from healthy donor). Two technical replicates per each biological replicate for the key experiments: ATAC-seq, RNA-seq, ARID1B chip-seq. The 2 biological replicates included a male and a female, comparable age ranges.
Data exclusions	n/a
Replication	2 biological replicates per condition (2 Coffin-Siris patient iPSC lines vs 2 control lines from healthy donor). Two technical replicates per each biological replicate for the key experiments: ATAC-seq, RNA-seq, ARID1B chip-seq. The 2 biological replicates included a male and a female, comparable age ranges.
Randomization	To avoid batch effect, the samples from different conditions (controls, patients) were always processed in the same batch
Blinding	n/a

Reporting for specific materials, systems and methods

We require information from authors about some types of materials, experimental systems and methods used in many studies. Here, indicate whether each material, system or method listed is relevant to your study. If you are not sure if a list item applies to your research, read the appropriate section before selecting a response.

Materials & experimental systems

n/a	Involved in the study
☐	☒ Antibodies
☐	☒ Eukaryotic cell lines
☐	☐ Palaeontology and archaeology
☐	☐ Animals and other organisms
☐	☐ Human research participants
☐	☐ Clinical data
☐	☐ Dual use research of concern

Methods

n/a	Involved in the study
☐	☒ ChIP-seq
☐	☒ Flow cytometry
☐	☐ MRI-based neuroimaging

Antibodies

Antibodies used	AARID1B ChIP-Seq: Abcam ab57461. ARID1B western blot: Santa-Cruz sc-32762 and Abcam ab57461. ARID1A ChIP-Seq: GeneTex GTX129433. ARID1A western blot: Cell Signaling Technologies 12354S. Beta-Actin western blot: Cell Signaling Technologies 8457P. SOX2 ChIP-Seq: Active Motif 39843. NANOG ChIP-Seq: R&D Systems AF1997. H3K27ac ChIP-Seq: Abcam ab4729. GAPDH western blot: Cell Signaling Technologies 5174T. CD10 Flow Cytometry: Miltenyi Biotech 130-124-262. CD99 Flow Cytometry: Miltenyi Biotech 130-121-086. SSEA4 Flow Cytometry: Biolegend 330417. TRA-1-60-R Flow Cytometry: Biolegend 330609. IgG ChIP-qPCR: Cell Signaling Technologies 2729S. Cell Signaling HRP-conjugated anti-rabbit (7074S) and anti-mouse (7076S) were used as secondary antibodies in western blot. Spike-in Antibody: Active Motif 61686. Spike-in Chromatin: Active Motif 53083. Antibodies used in immunofluorescence: Anti-Mouse OCT4 (STEMCELL TECHNOLOGIES, 60059, 1:200); Rabbit Monoclonal Anti-Sox9 (abcam, ab185230,1:250); pPolyclonal Goat Anti-Nanog (R&D System, AF1997-SP,1:20); donkey anti-goat IgG (H+L) Alexa 488 (Jackson ImmunoResearch, 705-545-003, 1:500); donkey anti-mouse IgG (H+L) Alexa 647 (Jackson ImmunoResearch, 715-605-150, 1:500);

Donkey anti-rabbit IgG (H+L) Cy3 (Jackson ImmunoResearch, 11-165-152, 1:500). , Monoclonal mouse anti-human TRA-1-60 Antibody (Millipore MAB4360C3,1:100), Monoclonal mouse Anti-Stage-Specific Embryonic Antigen-4 Antibody (Millipore, MAB4304,1:100), Polyclonal Goat Anti- Human/Mouse Oct-3/4 Antibody (R&D System, AF1759,1:20).

Validation: n/a

Eukaryotic cell lines

Policy information about cell lines

Cell line source(s):
- Control line1 (SV20): University of Pennsylvania iPSC Facility
- Control line2 (GM237): Coriell Institute
- Patient lines: reprogrammed by the University of Leiden (The Netherlands)

Authentication: N/A

Mycoplasma contamination: All the lines tested negative for mycoplasma. Tests were run by the Stem Cell Facility at TJU

Commonly misidentified lines (See ICLAC register): N/A

Palaeontology and Archaeology

Specimen provenance: *Provide provenance information for specimens and describe permits that were obtained for the work (including the name of the issuing authority, the date of issue, and any identifying information).*

Specimen deposition: *Indicate where the specimens have been deposited to permit free access by other researchers.*

Dating methods: *If new dates are provided, describe how they were obtained (e.g. collection, storage, sample pretreatment and measurement), where they were obtained (i.e. lab name), the calibration program and the protocol for quality assurance OR state that no new dates are provided.*

☐ Tick this box to confirm that the raw and calibrated dates are available in the paper or in Supplementary Information.

Ethics oversight: *Identify the organization(s) that approved or provided guidance on the study protocol, OR state that no ethical approval or guidance was required and explain why not.*

Note that full information on the approval of the study protocol must also be provided in the manuscript.

Animals and other organisms

Policy information about studies involving animals; ARRIVE guidelines recommended for reporting animal research

Laboratory animals: *For laboratory animals, report species, strain, sex and age OR state that the study did not involve laboratory animals.*

Wild animals: *Provide details on animals observed in or captured in the field; report species, sex and age where possible. Describe how animals were caught and transported and what happened to captive animals after the study (if killed, explain why and describe method; if released, say where and when) OR state that the study did not involve wild animals.*

Field-collected samples: *For laboratory work with field-collected samples, describe all relevant parameters such as housing, maintenance, temperature, photoperiod and end-of-experiment protocol OR state that the study did not involve samples collected from the field.*

Ethics oversight: *Identify the organization(s) that approved or provided guidance on the study protocol, OR state that no ethical approval or guidance was required and explain why not.*

Note that full information on the approval of the study protocol must also be provided in the manuscript.

Human research participants

Policy information about studies involving human research participants

Population characteristics: Skin Fibroblasts were obtained from two Coffin-Siris syndrome patients, one male and one female.

Recruitment: Patients were recruited based on their willingness to provide a skin biopsy. We do not believe that this process is likely to lead to bias in the study results.

Ethics oversight: Leiden University

Note that full information on the approval of the study protocol must also be provided in the manuscript.

Clinical data

Policy information about clinical studies

All manuscripts should comply with the ICMJEguidelines for publication of clinical research and a completedCONSORT checklist must be included with all submissions.

Clinical trial registration	N/A
Study protocol	N/A
Data collection	N/A
Outcomes	N/A

Dual use research of concern

Policy information about dual use research of concern

Hazards

Could the accidental, deliberate or reckless misuse of agents or technologies generated in the work, or the application of information presented in the manuscript, pose a threat to:

No	Yes	
☒	☐	Public health
☒	☐	National security
☒	☐	Crops and/or livestock
☒	☐	Ecosystems
☒	☐	Any other significant area

Experiments of concern

Does the work involve any of these experiments of concern:

No	Yes	
☒	☐	Demonstrate how to render a vaccine ineffective
☒	☐	Confer resistance to therapeutically useful antibiotics or antiviral agents
☒	☐	Enhance the virulence of a pathogen or render a nonpathogen virulent
☒	☐	Increase transmissibility of a pathogen
☒	☐	Alter the host range of a pathogen
☒	☐	Enable evasion of diagnostic/detection modalities
☒	☐	Enable the weaponization of a biological agent or toxin
☒	☐	Any other potentially harmful combination of experiments and agents

ChIP-seq

Data deposition

☒ Confirm that both raw and final processed data have been deposited in a public database such as GEO.

☒ Confirm that you have deposited or provided access to graph files (e.g. BED files) for the called peaks.

Data access links
May remain private before publication.

https://nam10.safelinks.protection.outlook.com/?url=https%3A%2F%2Fwww.ncbi.nlm.nih.gov%2Fgeo%2Fquery%2Facc.cgi%3Facc%3DGSE169654&data=04%7C01%7Cmarco.trizzino%40jefferson.edu%7C95ccf4a973794d931b2008d8efba8a95%7C55a89906c710436bbc444c590cb67c4a%7C0%7C1%7C637522931757668718%7CUnknown%7CTWFpbGZsb3d8eyJWIjoiMC4wLjAwMDAiLCJQIjoiV2luMzIiLCJBTiI6Ik1haWwiLCJXVCI6Mn0%3D%7C1000&sdata=GoqzcBionXnoX90zxt9u3sk669N8H%2FlCrXOzimh%2Bxl4%3D&reserved=0

Files in database submission

ARID1B_CTRL_LINE1_rep1_peaks.txt
ARID1B_CTRL_LINE1_rep3_peaks.txt
ARID1B_CTRL_LINE2_rep1_peaks.txt
ARID1B_CTRL_LINE2_rep1_peaks.txt
ARID1B_P19_peaks.txt
ARID1B_P26_peaks.txt
ARID1B_KD_ATAC_CNCC5_peaks.bed
ARID1B_KD_CNCC5_H3K27ac_rep1_peaks.bed

ARID1B_WT_ATAC_CNCC5_peaks.bed
ARID1B_WT_CNCC5_H3K27ac_rep1_peaks.bed
CTRL_LINE1_ATAC_CNCC5_rep1.bed
CTRL_LINE1_ATAC_CNCC5_rep2_peaks.bed
CTRL_LINE1_iPSC_H3K27ac_rep1_peaks.bed
CTRL_LINE1_CNCC2_H3K27ac_rep1_peaks.bed
CTRL_LINE1_CNCC3_H3K27ac_rep1_peaks.bed
CTRL_LINE1_CNCC4_H3K27ac_rep1_peaks.bed
CTRL_LINE1_CNCC5_H3K27ac_rep1_peaks.bed
CTRL_LINE1_RNA_iPSC_rep1_counts.xlsx
CTRL_LINE1_RNA_iPSC_rep2_counts.xlsx
CTRL_LINE1_NANOG_CNCC5_rep1_peaks.bed
CTRL_LINE1_RNA_CNCC5_rep1_counts.xlsx
CTRL_LINE1_RNA_CNCC5_rep2_counts.xlsx
CTRL_LINE1_SOX2_CNCC5_rep1_peaks.txt
CTRL_LINE2_ATAC_CNCC5_rep1_peaks.bed
CTRL_LINE2_ATAC_CNCC5_rep2_peaks.bed
CTRL_LINE2_CNCC5_H3K27ac_rep1_peaks.bed
CTRL_LINE2_RNA_iPSC_rep1_counts.xlsx
CTRL_LINE2_RNA_iPSC_rep2_counts.xlsx
CTRL_LINE2_NANOG_CNCC5_rep1_peaks.bed
CTRL_LINE2_RNA_CNCC5_rep1_counts.xlsx
CTRL_LINE2_RNA_CNCC5_rep2_counts.xlsx
CTRL_LINE2_SOX2_CNCC5_rep1_peaks.txt
input_CNCC_mem_srt_q10_rmdup_normalized.bw
P19_ATAC_CNCC5_rep1_peaks.bed
P19_ATAC_CNCC5_rep2_peaks.bed
P19_CNCC5_ARID1A_peaks.txt
P19_CNCC5_H3K27ac_rep1_peaks.bed
P19_RNA_iPSC_rep1_counts.xlsx
P19_RNA_iPSC_rep2_counts.xlsx
P19_NANOG_CNCC5_rep1_peaks.bed
P19_RNA_CNCC5_rep1_counts.xlsx
P19_RNA_CNCC5_rep2_counts.xlsx
P19_SOX2_CNCC5_peaks.txt
P26_ATAC_CNCC5_rep1_peaks.bed
P26_ATAC_CNCC5_rep2_peaks.bed
P26_CNCC5_ARID1A_peaks.txt
P26_CNCC5_H3K27ac_peaks.bed
P26_RNA_iPSC_rep1_counts.xlsx
P26_RNA_iPSC_rep2_counts.xlsx
P26_NANOG_CNCC5_rep1_peaks.bed
P26_RNA_CNCC5_rep1_counts.xlsx
P26_RNA_CNCC5_rep2_counts.xlsx
P26_SOX2_CNCC5_peaks.txt
ARID1B_CTRL_LINE1_rep1.fastq.gz
ARID1B_CTRL_LINE1_rep3.fastq.gz
ARID1B_CTRL_LINE2_rep1.fastq.gz
ARID1B_CTRL_LINE2_rep1.fastq.gz
ARID1B_P19_peaks.fastq.gz
ARID1B_P26_peaks.fastq.gz
ARID1B_KD_ATAC_CNCC5.fastq.gz
ARID1B_KD_CNCC5_H3K27ac_rep1.fastq.gz
ARID1B_WT_ATAC_CNCC5.fastq.gz
ARID1B_WT_CNCC5_H3K27ac_rep1.fastq.gz
CTRL_LINE1_ATAC_CNCC5_rep1.fastq.gz
CTRL_LINE1_ATAC_CNCC5_rep2.fastq.gz
CTRL_LINE1_iPSC_H3K27ac_rep1.fastq.gz
CTRL_LINE1_CNCC2_H3K27ac_rep1.fastq.gz
CTRL_LINE1_CNCC3_H3K27ac_rep1.fastq.gz
CTRL_LINE1_CNCC4_H3K27ac_rep1.fastq.gz
CTRL_LINE1_CNCC5_H3K27ac_rep1.fastq.gz
CTRL_LINE1_iPSC_RNA_rep1.fastq.gz
CTRL_LINE1_iPSC_RNA_rep2.fastq.gz
CTRL_LINE1_NANOG_CNCC5_rep1.fastq.gz
CTRL_LINE1_RNA_CNCC5_rep1.fastq.gz
CTRL_LINE1_RNA_CNCC5_rep2.fastq.gz
CTRL_LINE1_SOX2_CNCC5_rep1.fastq.gz

CTRL_LINE2_ATAC_CNCC5_rep1.fastq.gz
CTRL_LINE2_ATAC_CNCC5_rep2.fastq.gz
CTRL_LINE2_CNCC5_H3K27ac_rep1.fastq.gz
CTRL_LINE2_iPSC_RNA_rep1.fastq.gz
CTRL_LINE2_iPSC_RNA_rep2.fastq.gz
CTRL_LINE2_NANOG_CNCC5_rep1.fastq.gz
CTRL_LINE2_RNA_CNCC5_rep1.fastq.gz
CTRL_LINE2_RNA_CNCC5_rep2.fastq.gz
CTRL_LINE2_SOX2_CNCC5_rep1.fastq.gz
input_CNCC5.fastq.gz
P19_ATAC_CNCC5_rep1.fastq.gz
P19_ATAC_CNCC5_Rep2.fastq.gz
P19_CNCC5_ARID1A.fastq.gz
P19_CNCC5_HK27ac_Rep1.fastq.gz
P19_iPSC_RNA_rep1.fastq.gz
P19_iPSC_RNA_rep2.fastq.gz
P19_NANOG_CNCC5_rep1.fastq.gz
P19_RNA_CNCC5_rep1.fastq.gz
P19_RNA_CNCC5_rep2.fastq.gz
P19_SOX2_CNCC5_rep1.fastq.gz
P26_ATAC_CNCC5_rep1.fastq.gz
P26_ATAC_CNCC5_rep2.fastq.gz
P26_CNCC5_ARID1A.fastq.gz
P26_CNCC5_H3K27ac_rep1.fastq.gz
P26_RNA_iPSC_rep1.fastq.gz
P26_RNA_iPSC_rep2.fastq.gz
P26_NANOG_CNCC5_rep1.fastq.gz
P26_RNA_CNCC5_rep1.fastq.gz
P26_RNA_CNCC5_rep2.fastq.gz
P26_SOX2_CNCC5_rep1.fastq.gz
CTRL_LINE1_CNCC5_ARID1A_peaks.bed
CTRL_LINE1_H3K27ac_CNCC7_rep1_peaks.bed
CTRL_LINE1_H3K27ac_CNCC9_rep1_peaks.bed
CTRL_LINE1_iPSC_ARID1A_normalized.bw
CTRL_LINE1_iPSC_ARID1B_normalized.bw
CTRL_LINE1_iPSC_ATAC_rep1_peaks.bed
CTRL_LINE1_iPSC_ATAC_rep2_peaks.bed
CTRL_LINE2_ARID1B_CNCC5_peaks.bed
CTRL_LINE2_iPSC_ARID1A_normalized.bw
CTRL_LINE2_CNCC5_ARID1A_peaks.bed
CTRL_LINE2_iPSC_ARID1B_normalized.bw
CTRL_LINE2_iPSC_ATAC_rep1_peaks.bed
CTRL_LINE2_iPSC_ATAC_rep2_peaks.bed
P19_ARID1B_CNCC5_peaks.bed
P19_H3K27ac_CNCC7_rep1_peaks.bed
P19_H3K27ac_CNCC9_rep1_peaks.bed
P19_iPSC_ARID1A_normalized.bw
P19_iPSC_ARID1B_normalized.bw
P19_iPSC_ATAC_rep1_peaks.bed
P19_iPSC_ATAC_rep2_peaks.bed
P26_ARID1B_CNCC5_peaks.bed
P26_H3K27ac_CNCC7_rep1_peaks.bed
P26_H3K27ac_CNCC9_rep1_peaks.bed
P26_iPSC_ARID1A_normalized.bw
P26_iPSC_ARID1B_normalized.bw
P26_iPSC_ATAC_rep1_peaks.bed
P26_iPSC_ATAC_rep2_peaks.bed
CTRL_LINE1_CNCC5_ARID1A.fastq.gz
CTRL_LINE1_H3K27ac_CNCC7_rep1.fastq.gz
CTRL_LINE1_H3K27ac_CNCC9_rep1.fastq.gz
CTRL_LINE1_iPSC_ARID1A.fastq.gz
CTRL_LINE1_iPSC_ARID1B.fastq.gz
CTRL_LINE1_iPSC_ATAC_rep1.fastq.gz
CTRL_LINE1_iPSC_ATAC_rep2.fastq.gz
CTRL_LINE2_ARID1B_CNCC5.fastq.gz
CTRL_LINE2_iPSC_ARID1A.fastq.gz
CTRL_LINE2_CNCC5_ARID1A.fastq.gz
CTRL_LINE2_iPSC_ARID1B.fastq.gz

CTRL_LINE2_iPSC_ATAC_rep1.fastq.gz
CTRL_LINE2_iPSC_ATAC_rep2.fastq.gz
P19_ARID1B_CNCC5.fastq.gz
P19_H3K27ac_CNCC7_rep1.fastq.gz
P19_H3K27ac_CNCC9_rep1.fastq.gz
P19_iPSC_ARID1A.fastq.gz
P19_iPSC_ARID1B.fastq.gz
P19_iPSC_ATAC_rep1.fastq.gz
P19_iPSC_ATAC_rep2.fastq.gz
P26_ARID1B_CNCC5.fastq.gz
P26_H3K27ac_CNCC7_rep1.fastq.gz
P26_H3K27ac_CNCC9_rep1.fastq.gz
P26_iPSC_ARID1A.fastq.gz
P26_iPSC_ARID1B.fastq.gz
P26_iPSC_ATAC_rep1.fastq.gz
P26_iPSC_ATAC_rep2.fastq.gz

Genome browser session
(e.g. UCSC)

N/A

Methodology

Replicates

2 biological replicates (2 patients, 2 control lines) per condition. For ATAC-seq, RNA-seq and for select CHIP-seq experiments, 2 technical replicate for each biological replicate were conducted.

Sequencing depth

- Length of reads: ALL 76bp single-End.
- Genome Assembly: hg19
-DEPTH (millions of sequenced reads/uniquely mapped reads):
ARID1B_CTRL_LINE1_rep1.fastq.gz: 31.5/24.7
ARID1B_CTRL_LINE1_rep3.fastq.gz: 83.4/71.1
ARID1B_CTRL_LINE2_rep1.fastq.gz: 25.5/20.4
ARID1B_P19.fastq.gz: 27.0/22.6
ARID1B_P26.fastq.gz: 22.7/18.4
ARID1B_KD_CNCC5_H3K27ac_rep1.fastq.gz: 78.4/67.3
ARID1B_WT_CNCC5_H3K27ac_rep1.fastq.gz: 63.2/52.1
CTRL_LINE1_iPSC_H3K27ac_rep1.fastq.gz: 23.4/18.7
CTRL_LINE1_CNCC2_H3K27ac_rep1.fastq.gz: 26.9/20.3
CTRL_LINE1_CNCC3_H3K27ac_rep1.fastq.gz: 28.9/23.4
CTRL_LINE1_CNCC4_H3K27ac_rep1.fastq.gz: 25.5/19.7
CTRL_LINE1_CNCC5_H3K27ac_rep1.fastq.gz: 41.5/33.2
CTRL_LINE1_NANOG_CNCC5_rep1.fastq.gz: 37.7/30.0
CTRL_LINE1_SOX2_CNCC5_rep1.fastq.gz: 22.7/16.7
CTRL_LINE2_CNCC5_H3K27ac_rep1.fastq.gz: 18.4/14.3
CTRL_LINE2_NANOG_CNCC5_rep1.fastq.gz: 12.7/9.5
CTRL_LINE2_SOX2_CNCC5_rep1.fastq.gz: 20.0/17.8
input_CNCC5.fastq.gz: 22.8/18.5
P19_CNCC5_ARID1A.fastq.gz: 32.0/26.7
P19_CNCC5_HK27ac_Rep1.fastq.gz: 41.2/35.7
P19_NANOG_CNCC5_rep1.fastq.gz: 35.3/29.7
P19_SOX2_CNCC5_rep1.fastq.gz: 15.6/11.4
P26_CNCC5_ARID1A.fastq.gz: 42.7/37.2
P26_CNCC5_H3K27ac_rep1.fastq.gz: 64.0/57.3
P26_NANOG_CNCC5_rep1.fastq.gz: 22.8/18.2
P26_SOX2_CNCC5_rep1.fastq.gz: 19.5/16.2
CTRL_LINE1_H3K27ac_CNCC7_rep1.fastq.gz 27.1/23.2
CTRL_LINE1_H3K27ac_CNCC9_rep1.fastq.gz 23.6/20.8
CTRL_LINE1_iPSC_ARID1A.fastq.gz 89.7/72.5
CTRL_LINE1_iPSC_ATAC_rep1.fastq.gz 77.8/68.7
CTRL_LINE1_iPSC_ATAC_rep2.fastq.gz 34.8/30.3
CTRL_LINE2_iPSC_ARID1A.fastq.gz 74.7/66.2
CTRL_LINE2_iPSC_ATAC_rep1.fastq.gz 41.9/34.6
CTRL_LINE2_iPSC_ATAC_rep2.fastq.gz 54.2/49.2
P19_H3K27ac_CNCC7_rep1.fastq.gz 38.1/32.2
P19_H3K27ac_CNCC9_rep1.fastq.gz 19.6/16.4
P19_iPSC_ARID1A.fastq.gz 16.6/13.5
P19_iPSC_ATAC_rep1.fastq.gz 49.4/45.2
P19_iPSC_ATAC_rep2.fastq.gz 81.4/72.9
P26_H3K27ac_CNCC7_rep1.fastq.gz 31.0/26.6

	P26_H3K27ac_CNCC9_rep1.fastq.gz 18.9/15.5<br>P26_iPSC_ARID1A.fastq.gz 32.1/28.4<br>P26_iPSC_ATAC_rep1.fastq.gz 34.6/28.7<br>P26_iPSC_ATAC_rep2.fastq.gz 19.8/14.5
Antibodies	ARID1B Abcam ab57461; ARID1A GeneTex GTX129433; SOX2 Active Motif 39843; NANOG R&D Systems AF1997; H3K27ac Abcam ab4729
Peak calling parameters	#PEAK CALLING WITH MACS2<br>macs2 callpeak -t CHIP_SAMPLE_mem_srt_q10_rmdup.bam -c input_mem_srt_q10_rmdup.bam -f BAM -g hs -n $2_mem_q10_srt_rmdup_peaks -q 0.05<br><br>#PEAK CALLING WITH HOMER:<br>makeTagDirectory CHIMP_SAMPLE_NAME CHIP_SAMPLE_mem_srt_q10_rmdup.bam<br>findPeaks CHIMP_SAMPLE_NAME-style factor -o auto -i INPUT_NAME -fdr 0.05
Data quality	Peaks were called using FDR <5% and 10 fold enrichment<br>Only replicated peaks across biological replicates were retained. Number of replicated peaks were:<br>H3K27ac_iPSC_CTRL: 18,631<br>H3K27ac_day2_CTRL: 15,059<br>H3K27ac_day3_CTRL: 32,421<br>H3K27ac_day4_CTRL: 30,382<br>H3K27ac_day5_CTRL: 25,844<br>ARID1B_WT_CNCC5_H3K27ac: 24,281<br>ARID1B_KD_CNCC5_H3K27ac: 44,045<br>H3K27ac_CNCC5_CTRL: 17,612<br>H3K27ac_CNCC5_PATIENTS: 17,791<br>NANOG_CTRL: 17,034<br>NANOG_PATIENTS: 21,819<br>SOX2_CTRL: 5,191<br>SOX2_PATIENTS: 4,043<br>ARID1B_CTRL: 6,486<br>ARID1B_CNCC5_PATIENTS: 757 (expected, there is almost total loss of ARID1B protein in patients)<br>H3K27ac-day7-CTRL: 96,600<br>H3K27ac-day7-P19: 115,737<br>H3K27ac-day7-P26: 113,844<br>H3K27ac-day9-CTRL: 111,773<br>H3K27ac-day9-P19: 120,605<br>H3K27ac-day9-P26: 153,134<br>ARID1A-iPSC-CTRL: 11,634<br>ARID1A-iPSC-PATIENTS: 26,434<br>ARID1A-CNCC5-CTRL: 304 (expected, no protein detected in WB)<br>ARID1A-iPSC-PATIENTS: 2,405 in P19, 17,080 in P26.
Software	- Adapter Trimming: TrimGalore! v0.6.5<br>- Read mapping (ATAC-seq and CHIP-seq): BWA mem v.0.7.17<br>- Peak calling (ATAC-seq and HISTONE CHIP-seq): Macs2 v2.2.71<br>- Peak calling (other chip-seq): Homer v4<br>- Gene expression quantification: Kallisto v0.46<br>- Differential Gene Expression: Deseq2 package<br>- Pathway Analysis: Ingenuity Pathway Analysis suite<br>- Motif Analysis: Meme-ChIP (Meme Suite)<br>- Heatmaps and Average Profiles: Deeptools v3.5.0<br>- Bam file filtering for Q=10 (uniquely mapping reads), sorting, and removal of PCR duplicates: Samtools v1.10

Flow Cytometry

Plots

Confirm that:

- [x] The axis labels state the marker and fluorochrome used (e.g. CD4-FITC).
- [x] The axis scales are clearly visible. Include numbers along axes only for bottom left plot of group (a 'group' is an analysis of identical markers).
- [x] All plots are contour plots with outliers or pseudocolor plots.
- [x] A numerical value for number of cells or percentage (with statistics) is provided.

Methodology

Sample preparation	To obtain a single cell suspension for flow cytometry analysis, control and patient cells were treated with Accutase for 5 minutes. Cells were then washed with cold PBS-2% FBS and live cells were counted. 1×106 cells/condition were resuspended in 100L PBS-2% FBS and stained. For pluripotency evaluation, 4 µl of the respective antibodies were used: APC anti-human SSEA-4 antibody (Biolegend, #330417) and PE anti-human TRA-1-60-R antibody (Biolegend, #330609). For analysis of differentiation, 2 µl of the respective antibodies were used: FITC anti-human CD10 (Miltenyi Biotec, #130-124-262) and APC anti-human CD99 (Miltenyi Biotec, #130-121-096). Cells were incubated for 15 min on ice and protected from light, before transferring them into FACS tubes containing additional 300L PBS-2% FBS.
Instrument	Flow cytometry data were acquired using a BD LSR II flow cytometer
Software	FlowJo Software version 10.7.
Cell population abundance	n/a
Gating strategy	Gating Strategy for Analysis of Pluripotency (A) and Differentiation (B): representation of hierarchical gating for control sample. Unstained control sample, top row; stained control sample, bottom row; Forward Scatter Area (FSC-A) vs Side Scatter Area (SSC-A) gating has been used to identify P1 "Live Cells" on the base of cell size and granularity, and also to remove dead cells and debris. Sequential gating has been used to identify single cells (SSC-H vs FSC-W and FSC-H vs SSC-W, "Doublet Discrimination" 1 and 2) by exclusion of doublets/clumps. Pluripotent cells were then identified by surface biomarker expression of SSEA-4 APC vs TRA-1-60-R PE within the population (Marker Expression APC vs PE). Differentiation cells were identified by surface marker expression of CD19-APC vs CD10-FITC within the population (Marker Expression APC vs FITC). Separation between negative (red population) and positive (blue population) cells is presented to the right.

☒ Tick this box to confirm that a figure exemplifying the gating strategy is provided in the Supplementary Information.

Magnetic resonance imaging

Experimental design

Design type	*Indicate task or resting state; event-related or block design.*
Design specifications	*Specify the number of blocks, trials or experimental units per session and/or subject, and specify the length of each trial or block (if trials are blocked) and interval between trials.*
Behavioral performance measures	*State number and/or type of variables recorded (e.g. correct button press, response time) and what statistics were used to establish that the subjects were performing the task as expected (e.g. mean, range, and/or standard deviation across subjects).*

Acquisition

Imaging type(s)	*Specify: functional, structural, diffusion, perfusion.*
Field strength	*Specify in Tesla*
Sequence & imaging parameters	*Specify the pulse sequence type (gradient echo, spin echo, etc.), imaging type (EPI, spiral, etc.), field of view, matrix size, slice thickness, orientation and TE/TR/flip angle.*
Area of acquisition	*State whether a whole brain scan was used OR define the area of acquisition, describing how the region was determined.*

Diffusion MRI ☐ Used ☐ Not used

Preprocessing

Preprocessing software	*Provide detail on software version and revision number and on specific parameters (model/functions, brain extraction, segmentation, smoothing kernel size, etc.).*
Normalization	*If data were normalized/standardized, describe the approach(es): specify linear or non-linear and define image types used for transformation OR indicate that data were not normalized and explain rationale for lack of normalization.*
Normalization template	*Describe the template used for normalization/transformation, specifying subject space or group standardized space (e.g. original Talairach, MNI305, ICBM152) OR indicate that the data were not normalized.*
Noise and artifact removal	*Describe your procedure(s) for artifact and structured noise removal, specifying motion parameters, tissue signals and physiological signals (heart rate, respiration).*
Volume censoring	*Define your software and/or method and criteria for volume censoring, and state the extent of such censoring.*

Statistical modeling & inference

Model type and settings	*Specify type (mass univariate, multivariate, RSA, predictive, etc.) and describe essential details of the model at the first and second levels (e.g. fixed, random or mixed effects; drift or auto-correlation).*
Effect(s) tested	*Define precise effect in terms of the task or stimulus conditions instead of psychological concepts and indicate whether ANOVA or factorial designs were used.*

Specify type of analysis: ☐ Whole brain ☐ ROI-based ☐ Both

Statistic type for inference (See Eklund et al. 2016)	*Specify voxel-wise or cluster-wise and report all relevant parameters for cluster-wise methods.*
Correction	*Describe the type of correction and how it is obtained for multiple comparisons (e.g. FWE, FDR, permutation or Monte Carlo).*

Models & analysis

n/a	Involved in the study
☒	☐ Functional and/or effective connectivity
☒	☐ Graph analysis
☒	☐ Multivariate modeling or predictive analysis
