# Supplementary material for: Inability to switch from ARID1A-BAF to ARID1B-BAF impairs exit from pluripotency and commitment towards neural crest formation in ARID1B-related neurodevelopmental disorders
Source: Nat Commun. 2021 Nov 9;12:6469. doi: 10.1038/s41467-021-26810-x (PMC8578637; doi:10.1038/s41467-021-26810-x)

g 2c, Control Line 1 ARID1B Time Course

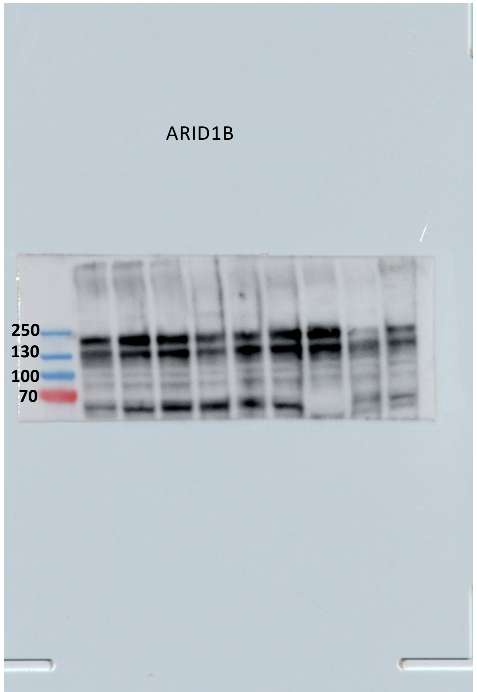

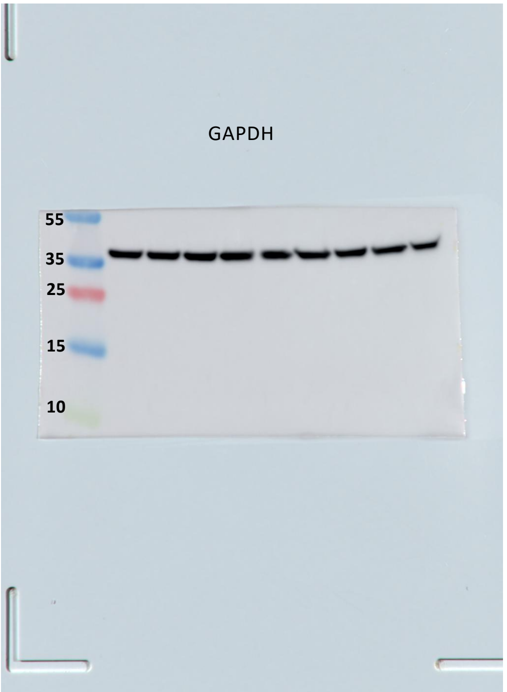

Fig 7a, Control Line 1 ARID1A Time Course

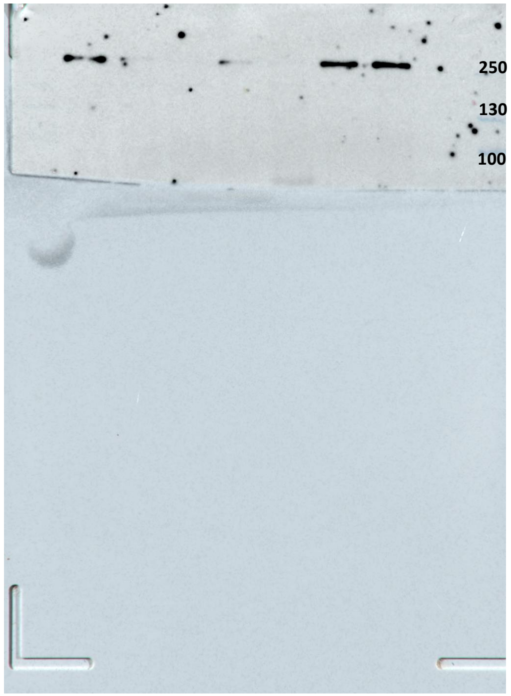

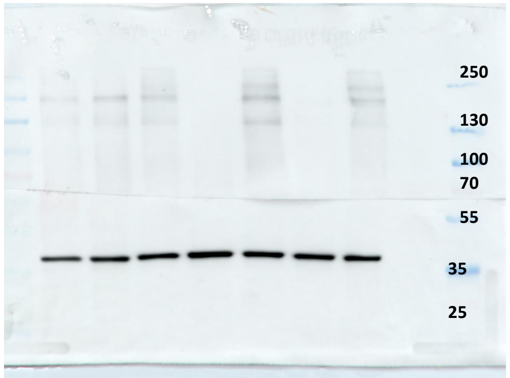

Fig 7a, Control Line 1 ARID1B Time Course

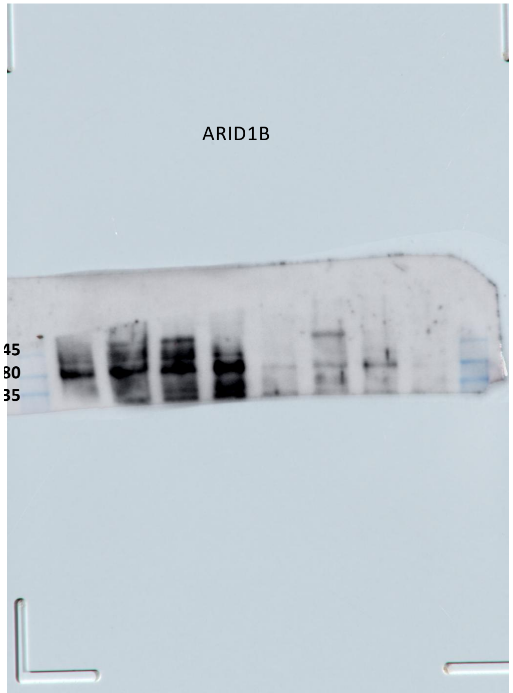

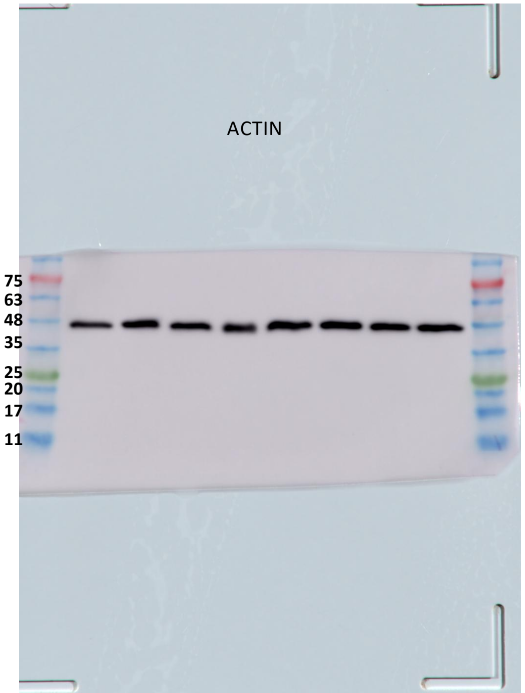

Fig 7b, P26 ARID1A Time Course

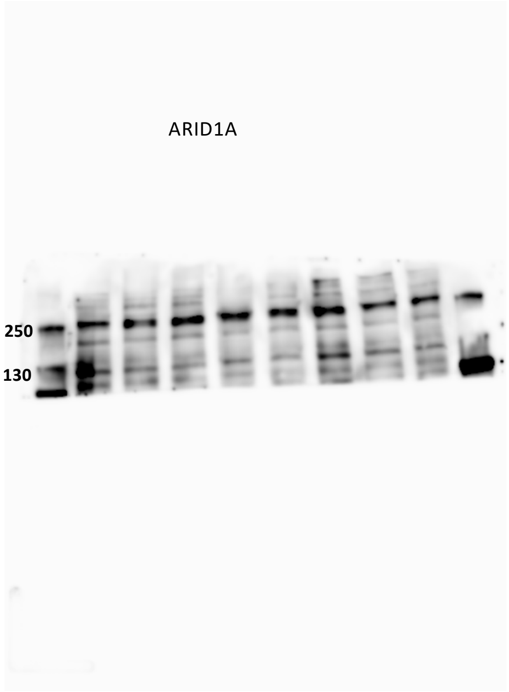

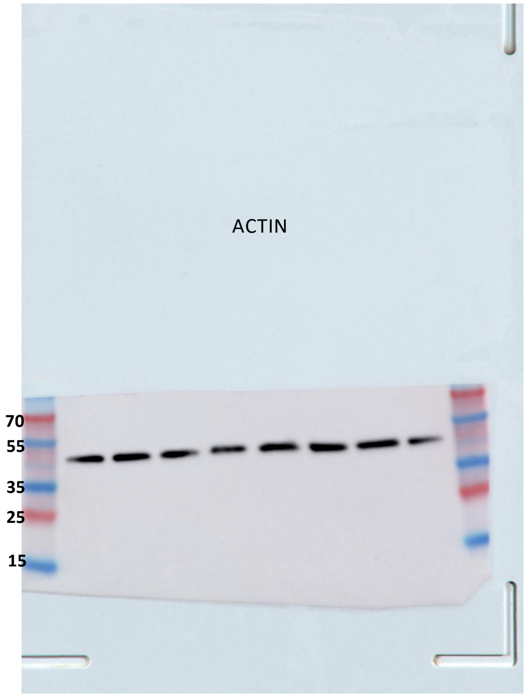

Fig 7b, P19 ARID1A Time Course

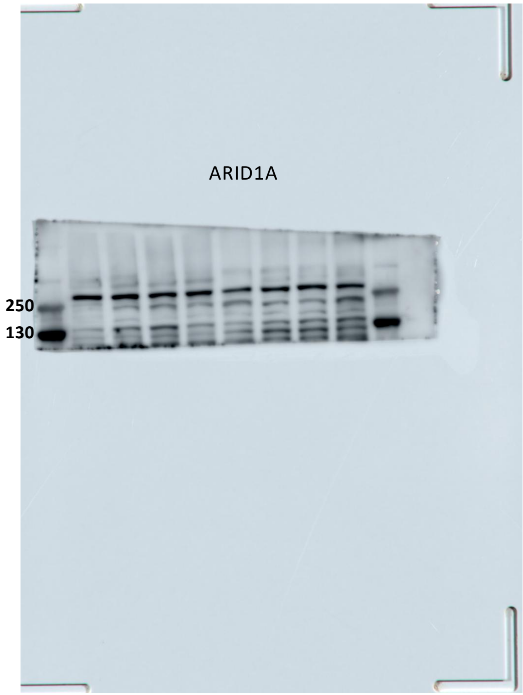

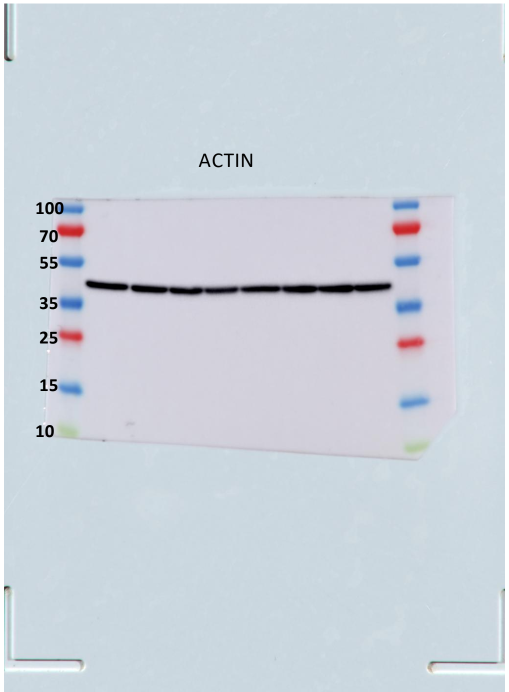

Fig 7c, ARID1A CNCC

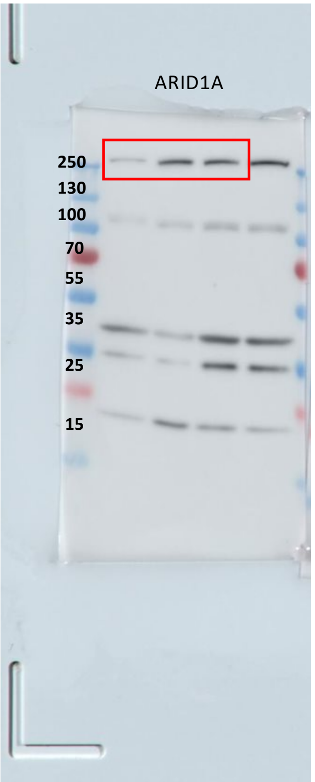

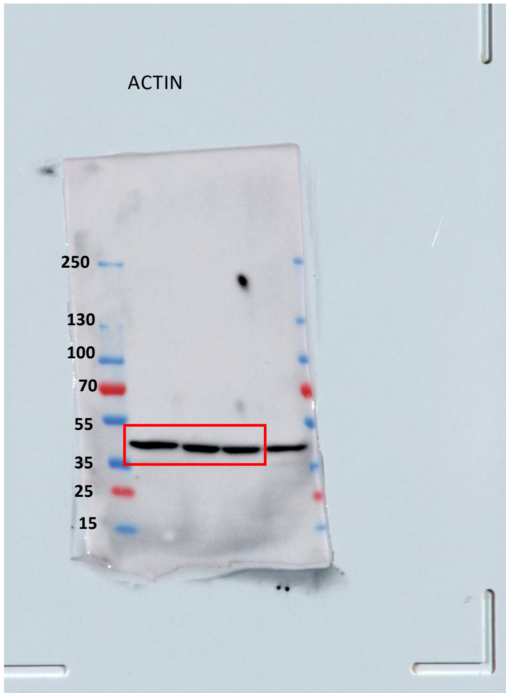

Fig S1a, Cytoplasmic Fraction of ARID1B

Fig S1a, Chromatin Fraction of ARID1B

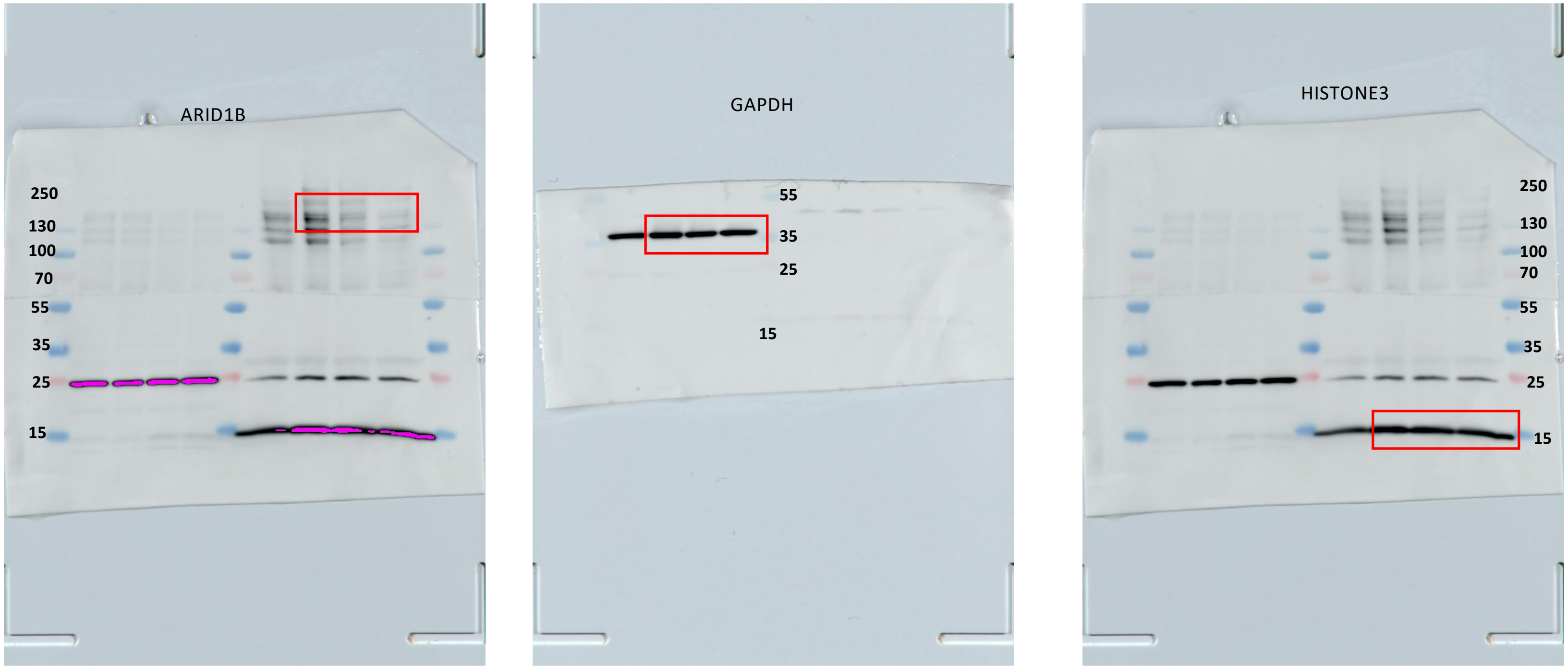

Fig S1a, Nuclear Fraction of ARID1B

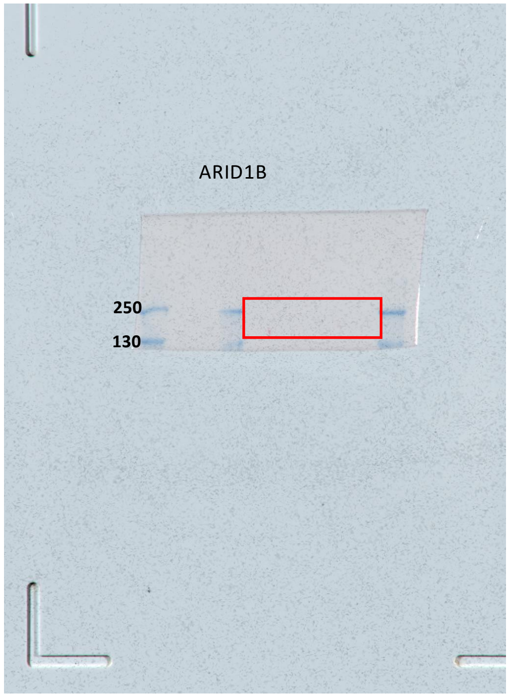

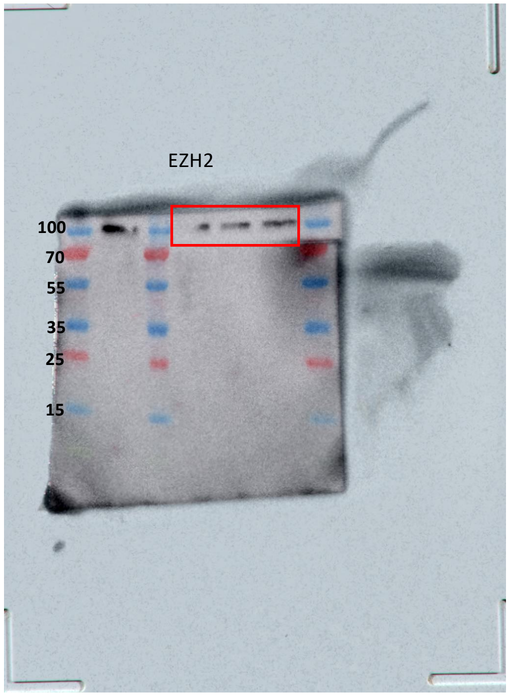

Fig S4c, Quantitative ARID1B

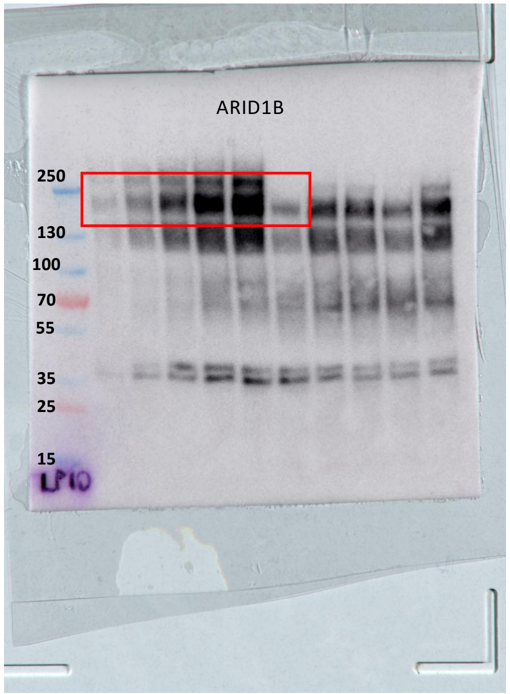

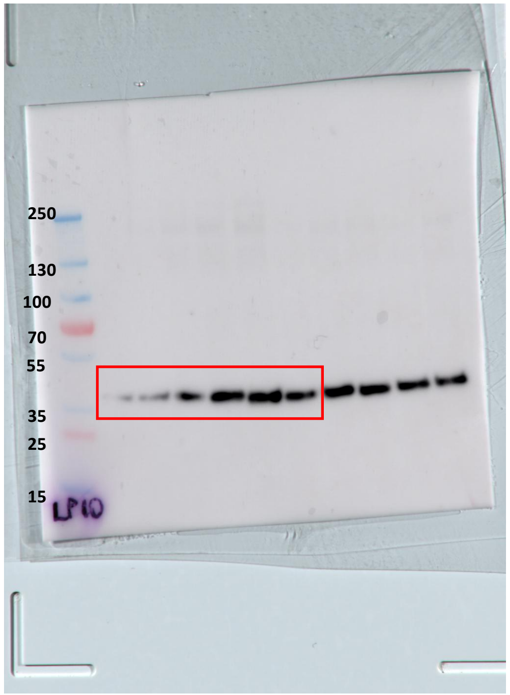

Supplement: Supplementary file 12 — Source data [file 41467_2021_26810_MOESM12_ESM.zip › SOURCE_DATA/Full_membranes_UNCROPPED.pdf]
